# Ubiquitin-dependent proteolysis of CXCL7 leads to posterior longitudinal ligament ossification

**DOI:** 10.1371/journal.pone.0196204

**Published:** 2018-05-21

**Authors:** Michiyo Tsuru, Atsushi Ono, Hideaki Umeyama, Masahiro Takeuchi, Kensei Nagata

**Affiliations:** 1 Clinical Proteomics and Gene Therapy Laboratory, Kurume University, Fukuoka, Japan; 2 Department of Orthopaedic Surgery, Hirosaki Memorial Hospital, Hirosaki, Japan; 3 Department of Biological Science, Chuo University, Tokyo, Japan; 4 Department of Clinical Medicine (Biostatistics), Kitasato University School of Pharmacy, Tokyo, Japan; 5 Department of Orthopaedic Surgery, Kurume University School of Medicine, Fukuoka, Japan; CHA University, REPUBLIC OF KOREA

## Abstract

Ossification of the posterior longitudinal ligament (OPLL), a spinal ligament, reduces the range of motion in limbs. No treatment is currently available for OPLL, which is why therapies are urgently needed. OPLL occurs in obesity, is more common in men, and has an onset after 40 years of age. The mechanisms underlying OPLL remain unclear. In this study, we performed a serum proteomic analysis in both OPLL patients and healthy subjects to identify factors potentially involved in the development of OPLL, and found reduced levels of a protein that might underlie the pathology of OPLL. We isolated the protein, determined its amino acid sequence, and identified it as chemokine (C-X-C motif) ligand 7 (CXCL7). Based on these proteomics findings, we generated a CXCL7 knockout mouse model to study the molecular mechanisms underlying OPLL. *CXCL7*-null mice presented with a phenotype of OPLL, showing motor impairment, heterotopic ossification in the posterior ligament tissue, and osteoporosis in vertebrate tissue. To identify the mechanisms of CXCL7 deficiency in OPLL, we searched for single nucleotide polymorphisms and altered DNA exons, but no abnormalities were found. Although miR-340 levels were found to be high in an miRNA array, they were insufficient to reduce CXCL7 levels. Ubiquitin C-terminal hydrolase1 (UCHL1) was found to be overexpressed in *CXCL7*-null mice and in the sera of patients with OPLL, and was correlated with OPLL severity. Post-translational modifications of proteins with ubiquitin and ubiquitin-like modifiers, orchestrated by a cascade of specialized ubiquitin activating enzyme (E1), ubiquitin conjugating enzyme (E2), and ubiquitin ligase (E3) enzymes, are thought to control a wide range of cellular processes, and alterations in the ubiquitin–proteasome system have been associated with several degenerative disorders. In addition, the OPLL tissue of *CXCL7*-null mouse and its primary cells expressed the antibody to ubiquitin (linkage-specific K48). Our data clearly show decreased CXCL7 levels in patients with OPLL, and that OPLL developed in mice lacking CXCL7. Tumor necrosis factor receptor-associated factor (TRAF)6 expression was decreased in *CXCL7*-null mouse primary cells. Furthermore, K48 polyubiquitination was found in posterior longitudinal ligament ossified tissue and primary cells from *CXCL7*-null mice. We performed a phosphoproteomics analysis in *CXCL7*-deficient mice and identified increased phosphorylation of mitogen-activated protein kinase kinase (ME3K)15, ubiquitin protein ligase E3C (UBE3C) and protein kinase C (PKC) alpha, suggesting that ubiquitin-dependent degradation is involved in CXCL7 deficiency. Future studies in the *CXCL7*-null mouse model are, therefore, warranted to investigate the role of ubiquitination in the onset of OPLL. In conclusion, CXCL7 levels may be useful as a serum marker for the progression of OPLL. This study also suggests that increasing CXCL7 levels in patients can serve as an effective therapeutic strategy for the treatment of OPLL.

## Introduction

Ossification of the posterior longitudinal ligament (OPLL) is associated with limb dysfunction resulting from spinal cord compression. The disease is classified into continuous, segmental, mixed, and circumscribed subtypes, and has a male-to-female ratio of 2–2.5:1 [1, 2]. OPLL typically develops after 40 years of age [3, 4] and is often accompanied by diabetes mellitus, changes in bone mineral density (BMD), diffuse idiopathic skeletal hyperostosis [5, 6], elevated plasma pentosidine levels, hypoparathyroidism [6, 7], ligamentum flavum ossification, increased prostaglandin (PG)E2/cyclic (c)AMP [8] and PGI2/cAMP [9] ratios, and obesity [10, 11]. Elevated serum insulin levels, which protect against high glucose levels, and increased body mass and insulinogenic indices are all correlated with the progression of ossification [12]. In mice deficient in insulin receptor substrate 1, bone formation and resorption in femoral and tibial bony tissues is reduced. Moreover, these mice have low bone metabolism and exhibit osteoporosis [13]. Herein, we performed a proteomics analysis to identify serum factors differentially expressed in patients with OPLL and healthy subjects. Pro-platelet basic protein (also known as chemokine (C-X-C motif) ligand 7; CXCL7) was found to be significantly downregulated in sera of patients with OPLL, and we hypothesized that OPLL pathology may originate from dysregulation of CXCL7 protein levels. To determine the mechanism underlying CXCL7 dysregulation, we generated CXCL7 knockout mice as a model for OPLL, and investigated the expression and activities of proteins in the ubiquitin-proteasome system, such as ubiquitin C-terminal hydrolase1 (UCHL1) [14]. The ubiquitin-proteasome system is a major pathway for protein degradation and involves three enzymes: a ubiquitin activating enzyme (E1), a ubiquitin conjugating enzyme (E2), and a ubiquitin transferase (ubiquitin ligase) (E3) [15, 16].

## Materials and methods

### Ethical statement

All study procedures were performed in accordance with the Declaration of Helsinki and the Good Clinical Practice guidelines. The study protocol was approved by Kurume University Hospital (approval Nos. 8 and 106), and performed in accordance with the experimental guidelines of the Japanese Orthopaedic Association Corporation as part of the research on intractable diseases under the Ministry of Health, Labour and Welfare of Japan. Individual information systems based on Freezer Works (FDA validation) were used. Informed written consent was obtained from all patients before the study, and patient care was unaffected by study participation.

### Animal welfare and management

All animal welfare and management procedures were performed using isoflurane and ketamine anesthesia according to standard protocols and the Guidelines for Proper Conduct of Animal Experiments (Science Council of Japan). Severity assessment in studies conducted in laboratory animals is an important issue with respect to implementing the 3R concept in biomedical research, and is a pivotal feature in current Japanese regulations. This study was therefore conducted in accordance with the Japanese Act on Welfare and Management of Animals protection [https://www.env.go.jp/en/laws/nature/act_wm_animals.pdf]. All experiments were approved and permitted by Kurume University. All procedures, including the euthanasia of morbidly ill mice, were performed under isoflurane and ketamine anesthesia according to standard protocols and the Guidelines for Proper Conduct of Animal Experiments (Science Council of Japan) [http://www.scaw.com/]. Based on the pain classification of the Scientists Center for Animal Welfare (SCAW), euthanasia of all experimental animals in this study was performed using ketamine (narcotics handler license: 230263/2011-2012, 250263/2013-2014, 270263/2015-2016, and 170263/2017) [17].

Action on the conservation and sustainable use of biological diversity was achieved by following regulations on the use of modified living organisms; the protocol was approved under Type 2 Regulations Concerning the Use of Living Modified Organisms of Kurume University (Approval no. 27–2) and was performed following Genetically Modified Animal Experiments Education Training (Approval nos. 136/2010, 92/2011, 191/2012, 166/2013, 55-1/2014, 21-1/2015, 46/2016 and 137/2017) [18, 19]. Routine microbiological monitoring was conducted according to the recommendations of the Federation of International Council for Laboratory Animal Science (ICLAS) and did not show any evidence of infection in mice with common urine pathogens. Mice were maintained in specific-pathogen-free (SPF) Level P1A rooms with a controlled environment: 21 ± 2°C; relative humidity 55 ± 5%; 12:12 h light:dark cycle (lights on at 7:00 a.m.), and 12–14 air changes hourly. Pelleted diet (CE-2, CLEA, Japan) and autoclaved (135°C/60 min) distilled water were provided *ad libitum*.

### Experimental design and statistical rationale

Serum samples were collected from 70 Japanese patients (43 males, 27 females; mean age, 63 years) with OPLL during the follow-up period from 2000 to 2012, at Fukui, Hirosaki, and Kurume Universities. We divided these patients into four groups based on OPLL subtype: continuous, 27 patients (18 males, 9 females; mean age, 67 years); mixed, 34 patients (21 males, 13 females; mean age, 65 years); segmental, 7 patients (3 males, 4 females; mean age, 62 years); and circumscribed, 2 patients (1 male, 1 female; mean age, 58 years). Sera from 57 healthy individuals in the control group were obtained during a health check of university officials and student volunteers. Informed written consent was obtained from all patients before the study, and patient care was unaffected by study participation. Samples were collected using Venoject II 7 mL tubes with a gel and clot activator (VP-P073K; Terumo, Tokyo, Japan), and serum was separated using a centrifuge and preserved at -80°C.

### Proteomics and purification methods for the target peak

Proteomics analysis was performed to identify key proteins involved in the development of OPLL. For surface-enhanced laser desorption/ionization-time of flight-mass spectrometry (SELDI-TOF MS) analysis, we centrifuged the collected human serum (200 μL) at 21,040 × *g* for 10 min to obtain the supernatant. A volume of 1.8 mL of 50 mM Tris buffer (pH 9.0) was then added, followed by cooling on ice for 20 min and centrifugation at 21,040 × *g* for 10 min. The supernatant was applied to an anion-exchange column (Q Sepharose Fast Flow, C/N 17-0510-01; GE Healthcare, Little Chalfont, UK) and washed four times with four column volumes of buffer (pH 9.0) for equilibration. Fractions were eluted at pH 7.0, collected, and then analyzed by SELDI with a cation exchange chip (CM10) to detect the target peaks in the non-adsorbed (pH 9.0) and eluted (pH 7.0) fractions. The purified crude fraction was subjected to further chromatographic fractionation. For the refinement and identification of target peaks, protein samples in the fractions obtained through purification by reverse-phase high-performance liquid chromatography (HPLC) were dried, digestion buffer was added, and the mixture was incubated at 70°C for 3 min. Modified trypsin (Promega, Madison, WI, USA) was then added, followed by incubation at 35°C for 5 h. The digested products were subjected to SELDI analysis (NP20 chip). External calibration was conducted using Arg-8-Vasopressin (1,084.25 Da), porcine dynorphin A 209–225 (2,147.5 Da), human ACTH 1–24 (29,335 Da), and bovine insulin β-chain (3,495.94 Da). The trypsin-digested solution was analyzed using liquid chromatography (LC)–tandem mass spectrometry (MS) (Q-TOF Ultima API LC/MS/MS; Waters Micromass, Manchester, UK), followed by a Mascot search. Non-adsorbed (pH 9.0) and elution (pH 7.0) fractions, obtained from the anion exchange column, were adjusted to pH 6.0 with 10% acetic acid, followed by fractionation using a cation-exchange column (Q Sepharose Fast Flow, C/N 17-0510-01, GE Healthcare), and washing three times with five column volumes of buffer (pH 6.0 + n-octyl-β-D-glucoside; OG). The 0.2 and 0.3 M NaCl eluted fractions obtained from the cation-exchange column were diluted five times with 0.1% TFA, and then subjected to reverse-phase HPLC (2-mm column), followed by elution using an acetonitrile concentration gradient under the following conditions: column: TSK-GEL, SuperODS (Tosoh Corporation, Tokyo, Japan) (2 × 100 mm); flow rate: 200 μL/min; detection: 210 nm; solvent A: 0.1% TFA; solvent B: 90% acetonitrile/0.1% TFA; gradient: 10–50% B/5-40 min; fraction: 200 μL/1 min/Fr and 100 μL/1 min/Fr (second chromatography). Fractions obtained by reverse-phase HPLC were diluted five times with acetonitrile and applied to normal-phase HPLC, followed by elution using an acetonitrile concentration gradient under the following conditions: column: TSK-GEL Amide-80 (Tosoh) (2 × 150 mm); flow rate: 200 μL/min; detection: 210 nm; solvent A: 0.1% TFA; solvent B: 90% acetonitrile/0.1% TFA; gradient: 100%–85%–65% B/5-40 min; fraction: 100 μL/0.5 min/Fr. Fractions obtained by normal-phase HPLC were diluted five times with 0.1% TFA and subjected to micro-reverse-phase HPLC (2-mm column), followed by elution using an acetonitrile concentration gradient under the following conditions: column: TSK-GEL SuperODS (Tosoh) (1 × 50 mm); flow rate: 50 μL/min; detection: 210 nm; solvent A: 0.1% TFA; solvent B: 90% acetonitrile/0.1% TFA; gradient: 10–50% B/5-40 min; fraction: 50 μL/1 min/Fr. Denaturation buffer (9 μL) was added to 1 μL of serum, mixed and incubated on ice for 10 min. Protein chip experiment buffer (90 μL; pH 6.0) was added to the mixture, and a further 150 μL of protein chip experiment buffer was separately added to a cation-exchange protein chip (CM10), followed by equilibration at 25°C for 5 min with stirring. These procedures were repeated twice. The above serum samples (100 μL each) were added to the protein chip, followed by incubation at room temperature for 30 min with stirring. Following this, 150 μL of protein chip experiment buffer (pH 6.0) was added to the protein chip, followed by equilibration at room temperature for 5 min with stirring. These procedures were repeated three times. For rinsing and desalting, 200 μL of Milli-Q water was added to each protein chip, and the procedure was repeated twice. The protein chips were air-dried, and 0.5 μL of saturation energy-absorbing molecule (sinapinic acid) solution was added, followed by air-drying. This was repeated twice, followed by measurement with a protein chip reader. Baseline correction, molecular weight calibration, and normalization were conducted.

DOI; dx.doi.org/10.17504/protocols.io.j9fcr3n

### DNA exon and SNP analyses

Genomic DNA was extracted from white blood cells (PAXgene-RNA tubes) using the PAXgene blood RNA kit (Qiagen, Hilden, Germany) according to the manufacturer's instructions (PAXgene blood RNA kit handbook version 2, April 2008). CXCL7 gene and adjacent DNA exons and SNP analyses were conducted using a PRISM3730 DNA Analyzer (Applied Biosystems, Foster City, CA, USA) and TaqMan SNP Genotyping Assay (Applied Biosystems) according to the manufacturer's instructions. (Hokkaido System Science, Sapporo, Japan) (S1 Data. Supplementary SNP data).

DOI; dx.doi.org/10.17504/protocols.io.j9gcr3w

### *CXCL7*-null mouse model

The *Ppbp (CXCL7)* gene knockout allele was generated by a procedure referred to as reporter-tagged insertion with conditional potential (Transgenic, Kobe, Japan). The targeted allele *Ppbp (CXCL7)*, contained within gene trap and selection cassettes, is a conventional knockout allele, as insertion of the cassettes disrupts the targeted gene by splicing in C57BL/6N embryonic stem (ES) cells. The promoter-driven targeting cassette consists of the gene trap and selection cassettes flanked by flippase (Flp) recognition target (FRT) sites. Deletion of exon 2 and 3 creates a frame-shift mutation in *Ppbp*. A third loxP site was inserted immediately after exon 3 to remove exons 2 and 3, and generate *Ppbp-*inactivated mice in which both alleles of *Ppbp* exon 3 are homozygous null (S2 Data. Supplementary conditional *Ppbp*^*dE2E3/+*^ knockout mice data).

### In situ hybridization

We generated a miR-340 probe by tagging Hsa-miR-340 with Alexa Fluor 488. Locked nucleic acid; LNA-*in situ* hybridization (ISH) was performed according to the manufacturer’s instructions (http://www.exiqon.com/mirna-ish-kit). Sections of FFPE tissues of human OPLL and *CXCL7*-null mice (4-μm-thick) were fixed with 4% PFA in PBS for 20 min at room temperature, washed with PBS (3 × 5 min), treated with 0.5% Triton X-100 (10 min at 4°C), and briefly washed with PBS followed by two washes (10 min each) in saline-sodium citrate buffer (2× SSC, 0.3 M NaCl, 0.03 M Na_3_С_6_Н_5_О_7_, рН 7.0). The sections were then digested with 15 μg/mL proteinase K (Exiqon, Vedbaek, Denmark) at 37°C for 10 min and rinsed for 3 × 5 min in PBS. Hybridization was performed in a humidified chamber for 18 h at 65°C. *In situ* hybridization of miR-340 was conducted using the miRCURY LNA microRNA ISH Optimization kit (Exiqon, Vedbaek, Denmark). miR-340-Alexa Fluor 488 and bone morphogenetic protein 2 (Alexa Fluor 555) were detected in human OPLL tissue by *in situ* hybridization and immunocytochemistry, respectively. Nuclei were counterstained with DAPI. After staining, the tissues were observed by fluorescence microscopy (BZ-X700, Keyence, Osaka, Japan).

DOI; dx.doi.org/10.17504/protocols.io.j9hcr36

### Bone histomorphometric parameters in wild-type and knockout mice

The spinal bones of knockout mice were fixed in ethanol, stained by the Villanueva method, and embedded in methyl methacrylate without decalcification. The administration schedule involved a single subcutaneous injection of the calcium chelating agent tetracycline to *CXCL7*-null and wild-type mice, followed by no administration for the next two days. Thereafter, mice were given a single subcutaneous injection of calcein to double-label the bone, followed by no administration on the next day. The results of the bone histomorphometric analysis are expressed according to the methods of the ASBMR Histomorphometry Nomenclature Committee, excluding the osteocyte parameters.

DOI; dx.doi.org/10.17504/protocols.io.j9icr4e

### Phosphoproteomics

Proteins from the spinal ligament cells of wild-type and *CXCL7*-null mice were separated using two-dimensional gel electrophoresis and stained using the Pro-Q Diamond phosphoprotein gel stain (Thermo Fisher Scientific) followed by SYPRO Ruby protein gel stain (Thermo Fisher Scientific). The gel was visualized using a Molecular Imager FX Pro Plus multi imager system (Bio-Rad, Hercules, CA, USA), and the images were acquired using PDQuest software, version 8.0 (Bio-Rad). The composite images were digitally pseudo-colored and overlaid. The gels were treated with Pro-Q Diamond, and the phosphorylation spots were verified using LC–MS/MS after in-gel digestion using trypsin and peptide extraction [20], both of which were performed according to a previously published protocol [21]. Purified peptides (20–30 pmol) were analyzed using the UltiMate 3000 RSLCnano system (ThermoFisher Scientific) coupled to an Orbitrap Elite linear ion trap mass spectrometer (ThermoFisher Scientific) with an in-house-manufactured nano-electrospray ionization interface. For micro reversed-phase LC-MS/MS analysis, the samples were injected into a trap column (nano; 75 × 280 μm inner × outer diameter; packed with 15 cm Acclaim Pep Map C18). Buffer A (0.1% formamide) and Buffer B (80% acetonitrile and 0.0 8% formic acid) were used to elute the bound peptides with a split flow system (flow rate: 300 nL/min) for 60 min on a linear gradient. In positive ion mode, the spectra were acquired with cycles of one full MS scan in the linear trap quadrupole (*m*/*z* 350–2000) followed by twenty data-dependent MS/MS scans with a normalized collision energy of 35%.

DOI; dx.doi.org/10.17504/protocols.io.j9jcr4n

### Gene expression microarrays

RNA samples (RNA concentration: 100 ng/μL) were prepared from the kidney tissues of both homozygous knockout and wild-type mice and subjected to gene array analyses (Agilent Technologies, Santa Clara, CA, USA) (GEO array GES57590). RNA samples were extracted from the blood of healthy subjects and patients with OPLL, and then subjected to microRNA array analyses (Agilent Technologies) (GEO array GES57592). Total RNA was isolated from frozen whole tissue samples with TRIzol (Invitrogen, Carlsbad, CA, USA), and from blood samples with Blood RNA Tubes (PAXgene; PreAnalytiX, Hombrechtikon, Switzerland). RNA samples with RIN values ≥ 9.0, as determined using a 2100 Bioanalyzer (Agilent, Waldbronn, Germany), were selectively used for these analyses. Pathway analysis was performed based on the results of genomic and miRNA arrays (Agilent Technologies, Santa Clara, CA, USA) of samples from patients with OPLL.

DOI; dx.doi.org/10.17504/protocols.io.j9kcr4w

### Culture of bone marrow stromal cells and mesenchymal stem cells

Bone marrow aspirate was obtained from equine ilium, and mesenchymal stem cells (MSCs) were separated from the aspirate by density-gradient centrifugation (Ficoll-Hypaque gradient centrifugation) (GE Healthcare, Pittsburgh, PA, USA) and further cultured [22].

DOI; dx.doi.org/10.17504/protocols.io.kcicsue

### Ubiquitinylation assays

Human white blood cells were disrupted and ubiquitinylated proteins were recovered using a K48 Linkage Specific UbiTest kit (Life Sensors, Inc., Malvern, PA, USA); in particular, expression of K48 polyubiquitin was verified by performing CXCL7 immunoblot analysis on human white blood cells. As a control with the K48 deubiquitinating enzyme, ubiquitin specific peptidase 2 catalytic domain (USP2CD) was used [23]. Ubiquitinylated proteins were resolved by SDS-PAGE [24].

DOI; dx.doi.org/10.17504/protocols.io.nxcdfiw

### Statistical analyses

Descriptive statistics were calculated and continuous variables are presented as the mean ± standard deviation. Differences between groups under multiple conditions were determined by one-way analysis of variance followed by Bonferroni’s multiple comparison tests. Welch's t-test was performed for between-groups analyses. All statistical analyses were performed using SAS 9.4 software (SAS Institute, Cary, NC, USA).

## Results and discussion

### Discovery of a disease-specific protein through human proteomics

**Fig 1:**
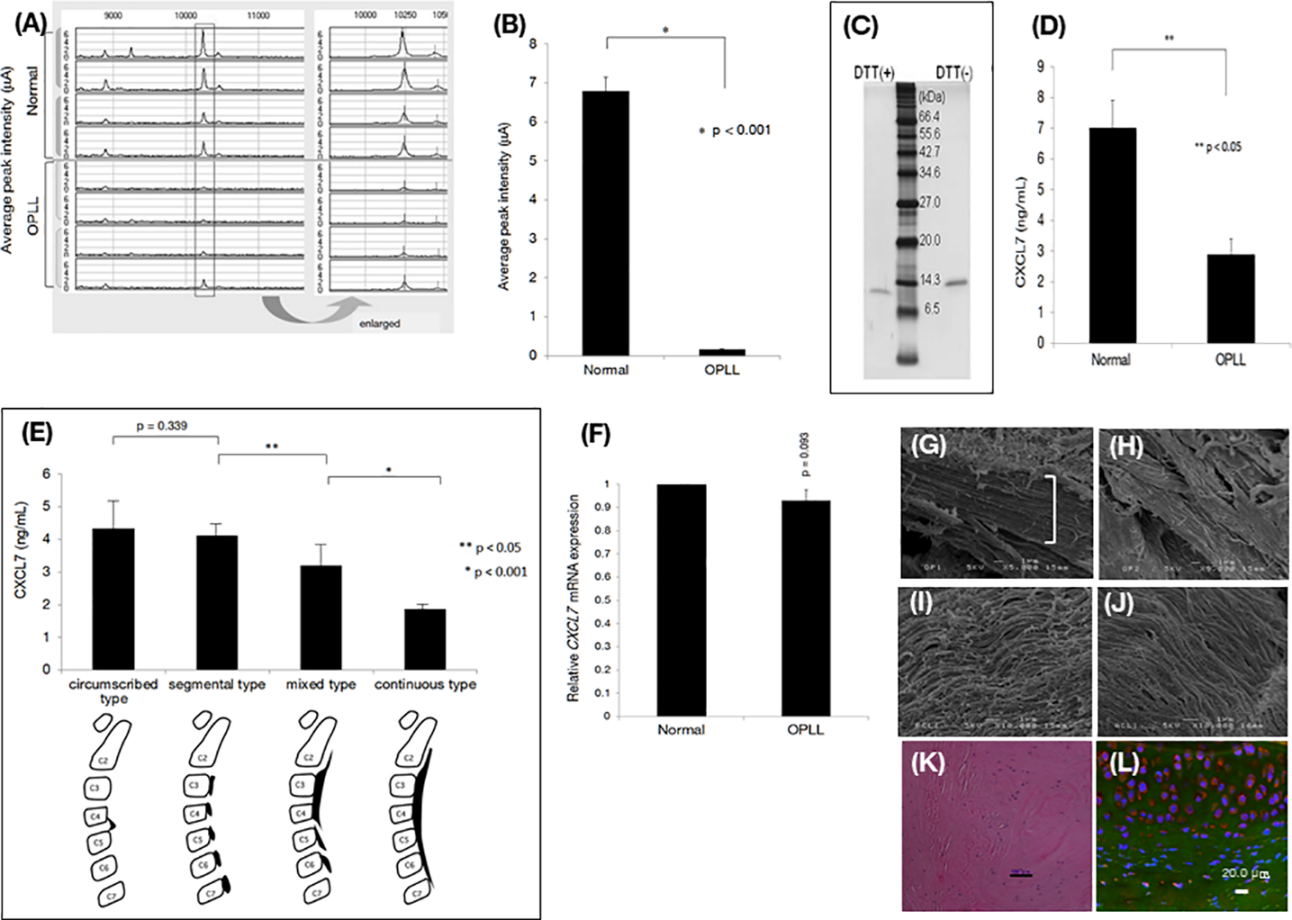
Characterization of human posterior longitudinal ligament ossification (OPLL). (**A)** Serum proteins downregulated in four OPLL patients *vs*. four healthy controls and (**B**) quantitative analysis of one of these proteins. In proteomics analysis, a specific peak of mass 10300 Da was decreased in OPLL patients compared to healthy subjects. (**C**) SDS PAGE and silver staining of a purified sample of the peak of interest with/without dithiothreitol. (**D**) Serum chemokine (C-X-C motif) ligand 7 (CXCL7) concentration determined by ELISA (normal, n = 7; OPLL, n = 13). (**E**) CXCL7 levels measured by ELISA in OPLL subtypes (continuous, n = 17; mixed, n = 15; segmental, n = 7; circumscribed, n = 2). (**F**) Real-time PCR analysis of CXCL7 gene expression in total RNA extracted from human (normal, n = 7; OPLL, n = 13). Human OPLL tissues were also examined by scanning electron microscopy as follows: (**G**) Segmental-type OPLL tissue (bracket) and (**H)** continuous-type OPLL tissue. The presence of OPLL fibers was confirmed, as indicated by the arrows below the fibers. Scanning electron microcopy of **(I**) human PCL tissue and (**J**) human ACL tissue. (**K**) H&E staining of human OPLL tissue (scale bar; 100 μm). (**L**) Immunohistochemical staining and *in situ* hybridization of human OPLL tissue (axial direction) confirmed the expression of Has-miR-340 aatcaG(L)t5(L)aT(L)tG(L)cT(L)ttataa_N(6)_Y with Alexa Fluor 488 fluorescence and BMP-2 (Alexa Fluor 555 labeled). Nuclei were stained with DAPI.

In the proteomic analysis (Fig 1A), the height of one peak was significantly decreased in patients with OPLL (Fig 1B). Purification of the corresponding protein by reverse-phase high performance liquid chromatography (HPLC) followed by LC-MS-MS and a Mascot database v2.2.2 (Matrix Science, UK, http://www.matrixscience.com/) search identified the protein as pro-platelet basic protein (also known as chemokine (C-X-C motif) ligand 7; CXCL7) (S1A–S1E Fig). CXCL7 was purified by HPLC and analyzed by SDS-PAGE in the presence or absence of dithiothreitol (DTT). Silver staining revealed a band of approximately 13 kDa, along with a band of slightly higher molecular weight (Fig 1C), suggesting that the target protein comprised a single polypeptide with intermolecular disulfide bonds. Disruption of the disulfide (S-S) bonds by DTT increased the CXCL7 protein mass and changed the migration of the protein on SDS-PAGE, due to a disruption in molecular structure [25], and thus confirmed the presence of two S-S bridges [26] (S1F Fig). Serum CXCL7 levels, measured by enzyme-linked immunosorbent assay (ELISA), were reduced in patients with continuous OPLL compared to patients with mixed OPLL (Fig 1D). Similarly, in patients with segmental or circumscribed subtypes, CXCL7 levels were reduced compared to those in healthy controls, but were higher than those in mixed-subtype patients (Fig 1E).

**Fig 2:**
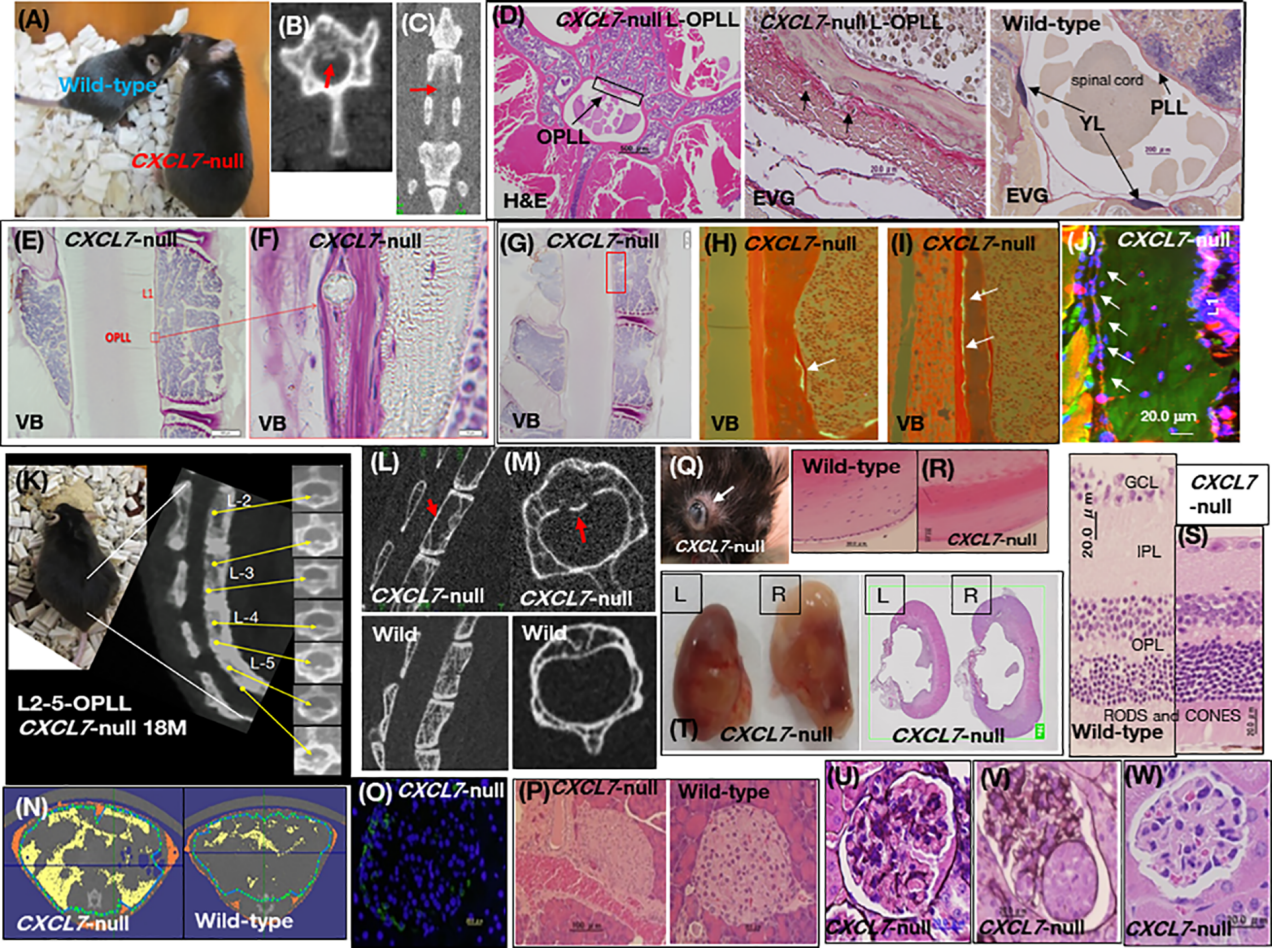
Analysis of chemokine (C-X-C motif) ligand 7 (*CXCL7*)-null mice. (**A**) *CXCL7*-null mouse and wild-type mouse at 8 months of age. (**B**) *CXCL7-*null mouse transverse (arrow; OPLL) and (**C**) vertical image of L1–L3 (arrow; OPLL). (**D**) H&E and EVG staining of the lumbar OPLL tissue in a *CXCL7*-null mouse and EVG staining of lumbar tissue in a wild-type mouse. Histological analysis of the posterior longitudinal ligament (PLL) of the wild-type mouse showed symmetrically arranged elastic fibers intercalated with collagen fibers. Posterior longitudinal ligament ossified tissue in the *CXCL7*-null mouse adheres to the dura, and the fibrous tissue of the ligament tissue is unidentified (arrows). (**E**) Villanueva bone (VB) staining of the first lumbar vertebrae (L1-OPLL) in a *CXCL7*-null mouse at 8 months of age. (**F**) Close-up of ligament ossification (arrow). (**G**) VB staining of L1-level ligament ossification in a *CXCL7*-null mouse at 8 months of age. (**H** and **I**) Fluorescence labeling in Fig 2G. Osteoblasts in the periosteum on the spinal side in a *CXCL7*-null mouse (indicated by arrows). (**J**) Immunohistochemical staining and *in situ* hybridization of OPLL tissue in a 10-month-old *CXCL7*-null mouse confirms the expression of Hsa-miR-340 (Alexa Fluor 488 fluorescence) and BMP-2. Nuclei were stained with DAPI. L1 vertebrae-OPLL (**K**) CT image of a *CXCL7*-null mouse with dysbasia at 18 months of age. The arrows indicate OPLL in L2-L5. Longitudinal (**L**) and cross-sectional (**M)** images. (**N**) Subcutaneous fat (orange) and visceral fat (yellow) analysis of a spinal cross-section at L3 by 3D-CT. (**O**) Immunohistochemical staining for glucagon (Alexa Fluor 488; green) in the pancreatic islet of Langerhans from a *CXCL7*-null mouse. Nuclei were counterstained with DAPI. (**P)** H&E-stained islets of Langerhans in the pancreatic tissue of a *CXCL7*-null T12-OPLL mouse. (**Q**) C3-level ligament ossification in a female *CXCL7*-null mouse showing a bulging eye. (**R)** PAS-stained optic lenses in wild-type and *CXCL7*-null mice. (**S**) H&E-stained retinal tissue from wild-type and *CXCL7*-null mice. GCL; ganglion cell layer, IPL; inner plexiform layer, OPL; outer plexiform layer, RODS and CONES; rod and cone layers. (**T)** L12-OPLL edematous kidney tissue in a *CXCL7*-null mouse. (**U** and **V**) periodic acid-Schiff (PAS)-stained and periodic acid methenamine silver-stained renal glomerulus of a *CXCL7*-null mouse. **(W)** After intravenous injection of human recombinant CXCL7 into a *CXCL7*-null mouse, the glomerular tissue of the kidney was examined by PAS staining after 3 months.

DNA exon and SNP analyses of the *CXCL7* gene revealed no difference between patients with OPLL and healthy subjects (S1 Data. Supplementary SNP data). Additionally, total serum *CXCL7* mRNA levels were similar in both groups (Fig 1F), suggesting a post-translational mechanism. Electron microscopy analysis of ligament tissue revealed ossification in patients with segmental-type OPLL (bracket; Fig 1G) or continuous-type OPLL (Fig 1H), compared to the posterior cruciate ligament (PCL; Fig 1I) and the anterior cruciate ligament (ACL; Fig 1J) in healthy individuals. In human OPLL tissues, chondrocyte morphology was assessed by hematoxylin and eosin (H&E) staining (Fig 1K). In the ligament tissue where ossification occurred, the normal array of collagen and elastic fibers disappeared, and the fibrocartilage layer could be seen. Bone morphogenetic protein (BMP)-2 (Alexa Fluor 555: red) expression was detected by immunohistochemistry (Fig 1L). When the posterior longitudinal ligament ossified tissue site was cross-sectioned and then stained for BMP-2 expression, the ossification site was confirmed to contain prominent ossified lesions expressing chondrocyte BMP-2. Based on these data obtained from patient sera, we generated a *CXCL7* knockout mouse (*CXCL7*-null mice) for use as an OPLL model (S3A–S3C Fig). Starting at 2 months of age, body weight, blood and urinary glucose, and urinary potassium levels were increased in the *CXCL7*-null mice. The *CXCL7*-null mice ingested large volumes of water and exhibited polyuria, hyperglycemia, and diabetic nephropathy. Furthermore, urinary calcium levels decreased, whereas urinary phosphorus and cAMP levels increased, even though cAMP levels were also elevated in the blood. The visceral fat area increased to 28.9%, indicating that the mice were obese (Table 1), and subcutaneous fat tissue could be easily observed during laparotomy (Fig 2A). At four months of age, decreased hind-limb motor function was observed in the knockout mice, resulting in a gait disorder of the hind legs (claudication) (Video1). Wild-type (WT) mice were able to move their limbs during swimming, (Video2), whereas knockout mice were only able to float their non-functional limbs (Video3). This loss of limb function was attributed to spinal column ligament ossification beginning at eight months of age (Fig 2B and 2C). H&E staining of the fifth lumbar spinal vertebra in a *CXCL7*-null mouse showed evidence of OPLL, as indicated by the arrow. In the enlarged photograph of Elastica van Gieson (EVG) staining of the same tissue, adhesion between the posterior longitudinal ligament ossified tissue and the dura was observed, as indicated by the arrow, and furthermore, the structure of the ligament tissue elastic fibers disappeared (Fig 2D) [27]. In addition, EVG staining of elastic fibers, such as elastin and collagen fibers, shown in black-violet [28] and red, respectively, did not show any notable changes in the yellow ligament (YL) or the dura mater in *CXCL7*-null mice (Fig 2D). This finding was confirmed in non-decalcified tissue samples using bone morphometry. Ossification was detected in the PLL at the first lumbar vertebra (L1) (Fig 2E and 2F and S2D Fig) [29], and the low calcification rate in the cancellous bone indicated a low metabolic turnover. Thinning of the bone cortical layer on the lateral and ventral sides of the spinal cord was also observed (Fig 2G–2I and Table 2). These features confirmed that *CXCL7*-null mice exhibited an OPLL phenotype. In addition, the expression of bone markers in the periosteum along the lateral side of the spinal cord suggested membranous ossification, which likely contributed to the pressure on the spinal cord (Fig 2K, 2L and 2M). In *CXCL7*-null mice, miR-340 and bone morphogenetic protein (BMP)-2 were detected by *in situ* hybridization and immunocytochemistry, respectively. The arrow in the figure indicates the chondrocytes located in the lining behind the spine. BMP-2 was expressed in chondrocytes, as shown in a horizontal cross section (Fig 2J and S2F Fig). Calcium and phosphorus are essential minerals in the blood and bone, and their concentrations are regulated by the kidney [30]. In osteoporosis, reduced kidney function results in excess phosphorus accumulating in the blood (Table 1). *CXCL7*-null mice exhibited a phenotype similar to human ectopic ossification (Table 2), showed locomotion abnormalities at 10 months of age, and were unable to eat or drink without assistance at 18 months of age because of their severely restricted mobility (Fig 2K). A 3D micro X-ray CT analysis revealed that the spinal trabeculae were coarser than those of age-matched wild-type mice (Fig 2L and 2M). Islets of Langerhans in *CXCL7-*null mice were negative for insulin and insulin receptor substrate 1 (IRS-1; data not shown), but the α-cells were positive for glucagon expression (Fig 2O). Bleeding, as a result of changes in the capillaries, was detected in *CXCL7*-null mice (Fig 2P), an exophthalmos was also noted in the third cervical vertebra, and OPLL was observed at 18 months of age in two females (Fig 2Q). The crystalline lens (Fig 2R) and the retina (Fig 2S) both had evidence of diabetic cataracts, and there was thinning of the inner plexiform layer (IPL) of the retina. Two of ten mice exhibited bilateral hydro-nephrosis (Fig 2T), and the 3 mL of residual urine in the bladder suggested a urinary disorder caused by OPLL of the twelfth thoracic vertebra (T12) at 18 months of age. In a female *CXCL7*-null mouse, edematous kidney tissues were stained with periodic acid methenamine silver (PAM staining; Fig 2U and 2V). Dark brown-stained mesangial matrix deposits were observed in a wide range of glomeruli. Cell proliferation in the mesangial matrix also increased in the glomeruli of *CXCL7*-null mice (Fig 2U). The glomerular epithelium was stratified, and crescentic glomerulonephritis was present (Fig 2V). Additionally, periodic acid-Schiff (PAS) staining of glomeruli was conducted in a *CXCL7*-null mouse after CXCL7 administration (1 mg/kg/week) (Fig 2W). No significant adverse effects were observed in the knockout mice after administration of CXCL7.

**Table 1:** Body weight, volume ratios of subcutaneous and visceral fat, and biochemical data for wild-type and *CXCL7*-null mice.

			Wild-type	*CXCL7*-null	p-value
Weight ♂ 8 weeks	(WT, n = 6) (CXCL7-/-, n = 10)	g	23.7 ± 0.26	27.8 ± 0.68	< 0.05
Weight ♀ 8 weeks	(WT, n = 12) (CXCL7-/-, n = 9)	g	18.5 ± 0.11	18.8 ± 0.32	0.45
Weight ♂ 8 months	(WT, n = 6) (CXCL7-/-, n = 10)	g	41.4 ± 0.55	47.4 ± 1.14	< 0.05
Weight ♀ 8 months	(WT, n = 12) (CXCL7-/-, n = 9)	g	29.7 ± 0.62	32.9 ± 0.98	< 0.05
Visceral adipose body ♂	(WT, n = 6) (CXCL7-/-, n = 10)	%	17.3 ± 0.38	28.9 ± 0.52	< 0.001
Subcutaneous fat body ♂	(WT, n = 6) (CXCL7-/-, n = 10)	%	9.2 ± 0.13	15.7 ± 0.68	< 0.001
Urinary sugar	(WT, n = 18) (CXCL7-/-, n = 19)	mg/dL	0.025 ± 0.02	81.79 ± 3.35	< 0.001
Blood sugar	(WT, n = 18) (CXCL7-/-, n = 19)	mg/dL	107.3 ± 3.57	411.38 ± 35.04	< 0.001
Urinary potassium	(WT, n = 13) (CXCL7-/-, n = 10)	mEq/L	5.1 ± 0.25	103.3 ± 4.66	< 0.001
Urinary calcium	(WT, n = 13) (CXCL7-/-, n = 10)	mg/dL	7.1 ± 0.35	3.4 ± 0.21	< 0.001
Blood calcium	(WT, n = 3) (CXCL7-/-, n = 5)	mg/dL	8.9 ± 1.3	9.6 ± 0.9	0.73
ALP	(WT, n = 18) (CXCL7-/-, n = 19)	U/L	890.6 ± 61.0	1468.8 ± 93.0	< 0.05
Urinary inorganic phosphorus	(WT, n = 11) (CXCL7-/-, n = 10)	mg/dL	2.7 ± 0.71	70.4 ± 12.9	< 0.001
Blood inorganic phosphorus	(WT, n = 3) (CXCL7-/-, n = 5)	mg/dL	2.7 ± 0.42	10.3 ± 2.18	< 0.001
Urinary cAMP	(WT, n = 2) (CXCL7-/-, n = 4)	pmol/mL	7.3 ± 2.3	5400 ± 84.2	< 0.001
Blood cAMP	(WT, n = 2) (CXCL7-/-, n = 4)	pmol/mL	1.5 ± 0.7	17.3 ± 1.5	< 0.05
Blood glucagon	(WT, n = 3) (CXCL7-/-, n = 2)	pg/mL	275 ± 91	509 ± 12	0.38
Urinary PTH-INTACT ♀	(WT, n = 2) (CXCL7-/-, n = 2)	pg/mL	13.1 ± 1.8	1 >	< 0.001
Urinary NTx	(WT, n = 2) (CXCL7-/-, n = 4)	nmol BCE/L	53 ± 13.7	387 ± 40.1	< 0.001
Blood adiponectin ♂	(WT, n = 2) (CXCL7-/-, n = 3)	μg/mL	5.2 ± 1.9	9.4 ± 0.82	0.07
Blood adiponectin ♀	(WT, n = 2) (CXCL7-/-, n = 3)	μg/mL	6.5 ± 1.1	12.4 ± 3.1	< 0.05

**Table 2:** Bone histomorphometric analysis of wild-type and *CXCL7*-null mice. The parameters measured in cortical bone were determined at the midpoint of the tibia. Data are expressed as the mean ± SEM of eight bones/group. Abbreviations related to osteocyte parameters in Table 2 are described in S1 Table in the Supplement (Histomorphometric parameters are described according to the American Society for Bone and Mineral Research (ASBMR) nomenclature committee).

		Wild-type (n = 3)	*CXCL7*-null (n = 2)	p-value
Cortical bone width				
Tr. Cr. (spinal cord)	μm^2^	111466.71 ± 24586	69265.38 ± 1309	< 0.001
Ps. S. (spinal cord)	μm	1889.21 ± 53.18	1928.72 ± 55.91	0.5361
Ct. Wi (spinal cord)	μm	59.12 ± 13.17	35.96 ± 1.72	< 0.001
Tr. Cr. (abdomen)	μm^2^	124602.45 ± 12588	128278.54 ± 18341	0.344
Ps. S (abdomen)	μm	1658.62 ± 120.91	1708.73 ± 151.11	0.706
Ct. Wi (abdomen)	μm	75.57 ± 8.06	74.71 ± 4.13	0.49
Cancellous bone				
TV	μm^2^	974730.69 ± 138968	1134063.68 ± 237903	0.442
BS	μm	7665.7 ± 875.86	10558.25 ± 1711.30	< 0.05
OS	μm	2433.74 ± 460.05	3307.58 ± 124.04	< 0.001
ES	μm	1878.37 ± 184.84	2368.23 ± 392.61	0.097
QS	μm	3353.6 ± 447.90	4882.45 ± 1442.72	0.1645
Ob. S	μm	1658.48 ± 281.28	2139.07 ± 190.26	0.0827
Oc. S	μm	583.22 ± 21.00	656.79605 ± 123.64	0.3619
BV	μm^2^	135404.33 ± 13835	229285.72 ± 66074	< 0.001
N. Mu. Oc	N	18 ± 1.53	17 ± 5.01	0.7615
N. Mo. Oc	N	6 ± 0.58	5.5 ± 0.50	0.4521
N. Ob	N	145 ± 25.423	183.5 ± 9.50	0.1542
OV	μm^2^	6767.04 ± 880.16	9952.26 ± 634.95	< 0.001
O. Th	μm	2.88 ± 0.22	3.01 ± 0.30	0.554
O. Th (SD)	μm	0.3 ± 0.04	0.16 ± 0.026	< 0.001
O. Th (n)	N	10	10	
dL. S	μm	664.84 ± 196.0	276.45 ± 251.90	0.138
sL. S	μm	120.58 ± 35.28	173.63 ± 114.56	0.462
Vd (d+s) LS	μm	1372.24 ± 478.33	2048.83 ± 194.56	0.1
L. Th	μm	3.46 ± 0.19	2.07 ± 0.25	< 0.001
L. Th (SD)	μm	0.43 ± 0.13	0.39 ± 0.11	0.075
L. Th (n)	N	10	10	
BV/TV	%	14.05 ± 0.77	19.87 ± 1.66	< 0.001
OV/TV	%	0.71 ± 0.10	0.91 ± 0.13	< 0.05
OV/BV	%	4.99 ± 0.44	4.65 ± 1.06	0.6535
Tb. Th	μm	35.59 ± 2.03	42.52 ± 5.62	0.1263
OS/BS	%	31.13 ± 2.76	32.37 ± 6.42	0.7898
Ob. S/ OS	%	68.79 ± 1.94	64.55 ± 3.33	0.1796
Ob. S/BS	%	21.33 ± 1.50	21.11 ± 5.22	0.9477
OV/OS	μm	2.86 ± 0.22	3.02 ± 0.31	0.5826
ES/BS	%	25.28 ± 4.19	22.42 ± 0.085	0.4943
Oc. S/ES	%	31.53 ± 2.53	27.63 ± 0.64	0.1416
Oc. S/BS	%	7.89 ± 1.26	6.19 ± 0.17	< 0.001
N. Mu. Oc/BS	N/mm	2.47 ± 0.53	1.57 ± 0.22	< 0.001
N. Mu. Oc/ES	N/mm	9.7 ± 0.89	7.02 ± 0.95	< 0.05
N. Mo. Oc/BS	N/mm	0.82 ± 0.18	0.54 ± 0.14	0.1646
N. Mo. Oc/ES	N/mm	3.28 ± 0.48	2.42 ± 0.61	0.2124
N. Ob/BS	N/mm	18.66 ± 1.32	18 ± 3.82	0.7986
N. Ob/OS	N/mm	60.38 ± 3.64	55.45 ± 0.79	0.2182
N. Oc/BS	N/mm	3.29 ± 0.71	2.12 ± 0.08	< 0.001
N. Oc/ES	N/mm	12.98 ± 1.36	9.45 ± 0.33	< 0.001
N. Mu. Oc/TV	N/mm^2^	19.28 ± 3.33	14.71 ± 1.32	0.1998
N. Mo. Oc/TV	N/mm^2^	6.52 ± 1.31	5.17 ± 1.53	0.4194
N. Oc/TV	N/mm^2^	25.79 ± 4.60	19.9 ± 0.20	0.2192
N. Ob/TV	N/mm^2^	148.55 ± 16.32	171.09 ± 44.27	0.4783
MAR	μm/day	1.15 ± 0.06	0.69 ± 0.08	< 0.001
MS/OS	%	55.26 ± 9.82	41.55 ± 10.73	0.2702
LS/OS	%	84.21 ± 15.11	75.02 ± 14.15	0.5983
Aj. Ar	μm/day	0.64 ± 0.14	0.3 ± 0.11	< 0.001
Omt	day	2.49 ± 0.16	4.5 ± 0.98	< 0.001
Mlt	day	4.92 ± 1.19	12.23 ± 5.52	< 0.05
BFR/BS	mm^3^/mm^2^/year	0.07 ± 0.02	0.038 ± 0.02	< 0.05
BFR/BV	%/year	435.41 ± 141.51	192.1 ± 118.37	< 0.05
BFR/TV	%/year	62.94 ± 23.71	36.21 ± 13.34	< 0.05
BS/TV	μm/μm^2^	0.008 ± 0.0009	0.009 ± 0.0005	0.1923
BS/BV	μm/μm^2^	0.06 ± 0.003	0.048 ± 0.006	0.1281
dL. S/BS	%	8.36 ± 1.98	3.09 ± 2.89	< 0.05
sL. S/BS	%	18.57 ± 4.59	22.1 ± 6.51	0.5585
Vd (d+s) LS/BS	%	16.86 ± 5.01	20.24 ± 5.12	0.565
LS/BS	%	26.93 ± 6.52	25.19 ± 9.40	0.8385
MS/BS	%	17.64 ± 4.23	14.14 ± 6.14	0.5384
BRs. R	mm^3^/mm^2^/year	0.04 ± 0.016	0.018 ± 0.011	< 0.05
Tb. N	N/mm	4.0 ± 0.45	4.7 ± 0.23	0.1849
Tb. Sp	μm	220.3 ± 23.85	170.59 ± 4.90	< 0.05

### Role of CXCL7 deficiency in OPLL patients

**Fig 3:**
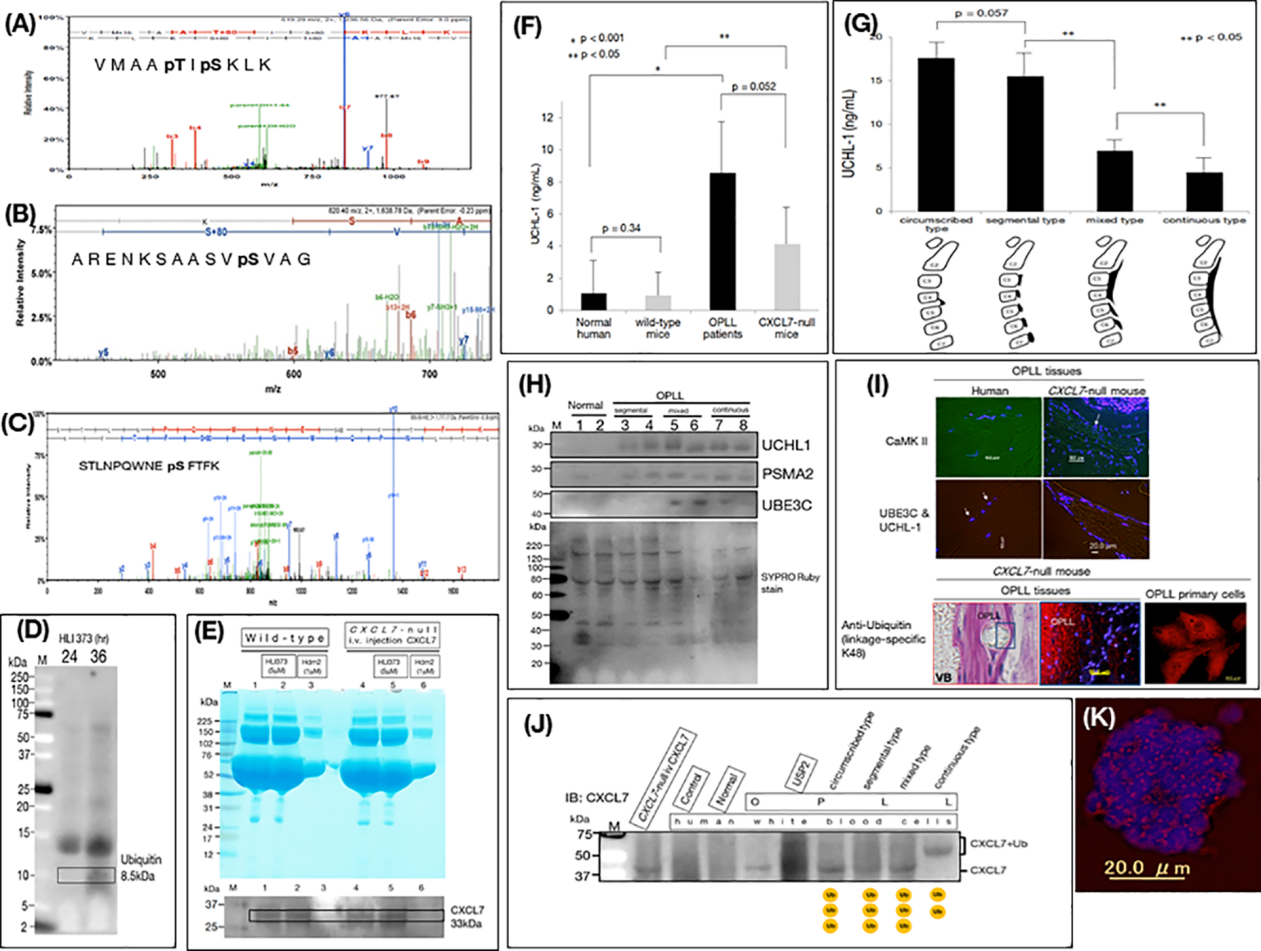
Phosphorylation analysis of posterior longitudinal ligament ossification (OPLL) in mice. Proteomics revealed phosphorylation of **(A)** MAPK kinase kinase (MAP3K)15 at threonine 1076 and serine 1078, **(B)** E3 ubiquitin ligase at serine 419, and **(C)** protein kinase C alpha at serine 235 in OPLL ligament primary cells derived from *CXCL7*-null mice. Function of the ubiquitin proteasome system in *CXCL7*-null mice. (**D**) Ubiquitin levels in primary cells derived from *CXCL7*-null mouse spinal ligaments cultured in the presence of 5 μM HLI 373 for 24 h and 36 h. (**E**) Wild-type spinal ligament primary cells, *CXCL7*-null OPLL ligament primary cells, or *CXCL7*-null OPLL ligament primary cells from CXCL7 animals treated with CXCL7 (i.v.) for 3 months were treated with the ubiquitin E3 ligase inhibitor HLI 373 (5 μM) or recombinant Hdm2 (1 μM). Cell lysates were separated by SDS-PAGE and stained with Coomassie brilliant blue (CBB) or western blotted for CXCL7. (**F**) Serum UCHL1 levels, as determined by ELISA in normal subjects, (n = 7), wild-type mice (n = 7), OPLL patients (n = 13), and *CXCL7*-null mice (n = 7). (**G**) Serum UCHL1 concentrations as determined by ELISA in different human OPLL subtypes (continuous, n = 17; mixed, n = 15; segmental, n = 7; circumscribed, n = 2). (**H**) Western blotting analysis of UCHL1, PSMA2, UBE3C, and CaMK II in the sera of patients with OPLL and normal human subjects. Lanes 1 and 2, normal subjects; lanes 3 and 4, segmental-type OPLL; lanes 5 and 6, mixed-type OPLL; lanes 7 and 8, continuous-type OPLL. The bottom panel shows the SYPRO ruby stain used to normalize protein loading. **(I)** Immunohistochemical staining of OPLL tissues from human OPLL patients, *CXCL7*-null mice and its primary cells, examining the expression of CaMK II (Alexa Fluor 488, left-hand side) and dual staining for UBE3C (Alexa Fluor 488; green), UCHL1 (Alexa Fluor 555; red), and ubiquitin (linkage-specific K48) (Alexa Fluor 555; red). The nuclei were stained with DAPI (blue) as indicated by the arrows. These images were obtained from the *CXCL7*-null L-OPLL tissue section shown in Fig 2D and 2F. (**J**) White blood cells were extracted from patients with OPLL and K48 ubiquitination analysis to examine CXCL7 ubiquitination was performed, because CXCL7 protein was present at low levels or was defective. The standard band of CXCL7 at 33 kDa and the band of CXCL7 in the higher macromolecular region indicates the K48 ubiquitin modification. The ubiquitination band of CXCL7 was not seen in healthy subjects, but was present in OPLL patients of the circumscribed, segmental, mixed, and continuous types. Agarose beads (UM400, Life Sensors, Inc.; Cat. # UM400) were used as a control without conjugation of TUBE. (**K**) Immunohistochemical staining of white blood cells of continuous-type patients with OPLL was performed. The expression of K48 (Alexa Fluor 555: red) is shown; nuclear staining was performed with DAPI.

We investigated phosphorylation by two-dimensional electrophoresis analysis of wild-type mice. Phosphorylation of ME3K15 (Fig 3A and S3A Fig), E3 ubiquitin ligase (Fig 3B and S3B Fig), and PKC alpha (Fig 3C and S3C Fig) was found to occur in *CXCL7*-null mice.

Based on these data, we reasoned that if the deficiency of CXCL7 in OPLL patients was due to an effect of the ubiquitin system, we could examine the expression of CXCL7 by western blotting to determine whether CXCL7 levels could be normalized in the presence of a ubiquitin inhibitor. We investigated the dysregulation in the ubiquitin system in *CXCL7*-null mice in primary cell cultures following treatment with the ubiquitin ligase inhibitor (5-(3- dimethylaminopropylamino)-3,10-dimethyl-10H-pyrimido(4,5-b)quinoline-2,4- dione dihydrochloride (HLI 373; 5 μM; Tocris Bioscience/R&D Systems, Minneapolis, MN, USA) after 24 and 36 hours of incubation. Ubiquitin expression was observed by western blotting 36 h after the addition of HLI 373 (Fig 3D). This result confirms that the ubiquitin inhibitor HLI 373 had a diminished effect at 36 hours. We administered human recombinant CXCL7 (Wako, Osaka, Japan) (1 mg/kg/week) to *CXCL7*-null mice by intravenous injection and prepared primary cell cultures from spinal ligament tissue (Fig 3E). To investigate the expression of CXCL7 in the posterior longitudinal ligament tissue, primary cells were generated from a small amount of spinal ligament tissue, the cells were proliferated, and western blotting was performed. CXCL7 was expressed in the primary cells of the spinal ligament tissues of wild-type mice and CXCL7 was expressed on addition of 1 μM Hdm2 (Enzo Life Sciences, Farmingdale, NY, USA) (Fig 3E), but when 5 μM HLI 373 was added, expression of CXCL7 disappeared. CXCL7 was expressed in early cultured cells of the posterior longitudinal ligament tissue of knockout mice intravenously injected with human recombinant CXCL7, but CXCL7 expression disappeared by HLI 373 [31–34]. In addition, western blotting of proteins from primary cells derived from the spinal ligament tissues of wild-type mice treated with the ubiquitin ligase inhibitor (HLI 373), treated with recombinant UCHL1, or transfected with miR-340 showed that expression of CXCL7 was not completely suppressed by miR-340 transfection. Furthermore, UCHL1-induced de-ubiquitination of CXCL7 or the presence of a polyubiquitin chain, that was not hydrolyzed by UCHL1, in ubiquitinated CXCL7 is suggested by the presence of a CXCL7 band in the macromolecular region (bracket) (S3K Fig). UCHL1 was found to be highly expressed in *CXCL7*-null mice, as well as in the sera of patients with OPLL (Fig 3F–3H). Moreover, western blotting of primary cells derived from the spinal ligament tissues from wild-type and *CXCL7*-null mice showed that UCHL1 and UBE3C were not expressed in wild-type mouse primary cells but were present in the CXCL7 knockout primary cells, and RANKL was expressed in both wild and *CXCL7*-null mouse primary cells but TRAF6 expression was decreased in *CXCL7*-null mouse primary cells. Furthermore, K48 polyubiquitination was found in the posterior longitudinal ligament ossified tissue and primary cells from *CXCL7*-null mice (Fig 3I and S3L Fig; https://www.ebi.ac.uk/interpro/entry/IPR032049, https://www.rcsb.org/structure/2LUA, http://pfam.xfam.org/family/PF16682) [35, 36]. K48 was immunoprecipitated after binding to Dynabeads protein A. The samples used for the immunoprecipitation were primary spinal ligament cells of wild type and *CXCL7*-null mice and sera of healthy subjects and OPLL patients. K48 polyubiquitination was observed in *CXCL7*-null mice and mixed-type and continuation-type OPLL patient sera (S3M Fig). Next, to investigate whether CXCL7 is a target for K48 polyubiquitylation, white blood cells were extracted from a patient with OPLL, and K48 polyubiquitination was examined [37, 38]. To investigate the target protease degradation of CXCL7 in OPLL, the patient's white blood cells were separated with K48 linkage-specific Ubi tandem ubiquitin binding entity (TUBE) beads and K48-bound polyubiquitinated (DUB) protein (Life Sensors, Inc., Malvern, PA, USA; Cat. # UM414), and a de-ubiquitinating enzyme (Ubiquitin Specific Peptidase 2 Catalytic Domain; USP2CD; in Figure USP2) with a wide-range and CXCL7 immunoblot analysis. The results in Fig 3J show that polyubiquitinated CXCL7 bound to K48 was not observed in healthy individuals, whereas it was observed in patients with OPLL, and in the classification, in addition to the K48 modification, presence of 2 to 3 polyubiquitins was suggested (bracket). In addition, administration of human recombinant CXCL7 to *CXCL7*-null mice by intravenous injection suggested degradation by K48, and administration for a long period was performed (Fig 3J and S3H Fig). K48 western blot analysis of white blood cells of healthy subjects and patients with OPLL was performed. K48 ubiquitin was confirmed in white blood cells of patients with OPLL (S3N Fig). Immunohistochemical staining with K48 antibody of white blood cells from a patient with continuous-type OPLL indicated no expression of CXCL7, but K48 expression was observed (Fig 3K). In immunohistochemistry of OPLL tissues obtained from humans, as well as from *CXCL7*-null mice, UCHL1 and UBE3C were detected in the nucleus (arrows in Fig 3I) and CaMK II was also detected. In addition, the OPLL tissue of *CXCL7*-null mouse and its primary cells expressed the antibody to ubiquitin (linkage-specific K48). (Fig 3I) [39–41]. These data suggest that CXCL7 might be continuously degraded by the ubiquitin pathway.

Long-term administration to determine whether exogenous CXCL7 protein can ameliorate OPLL in wild-type and *CXCL7*-null mice was conducted by intravenous injection of human recombinant CXCL7 once daily for 10 months. No specific changes were observed in wild-type mice, whereas there were marked decrease in blood and urinary glucose levels in *CXCL7*-null mice, suggesting amelioration of the disease (S3H Fig).

Gene expression related to MAPK-SAPK/JNK signaling was also determined by real-time PCR (S3E Fig) [42], and expression of CXCL7 itself by western blotting (S3G Fig), in both wild-type and *CXCL7*-null spinal ligament primary cells obtained from animals treated for 10 months with intravenous human recombinant CXCL7. The expression of phospho-SAPK/JNK and of CXCL7 in cells derived from wild-type mice was also examined by immunocytochemistry. MAPK-SAPK/JNK (Alexa Fluor 555: red) and CXCL7 (Alexa Fluor 488: green) expression in cells derived from wild-type mice was observed by immunocytochemistry; in particular, nuclear import of CXCL7 was confirmed in cells derived from *CXCL7*-null mice administered CXCL7, and this effect was blocked by the ubiquitin ligase inhibitor HLI 373 (S3J Fig). In wild-type mouse primary cells, the expression of p38 MAPK and nuclear translocation of CXCL7 were shown by immunocytochemical staining, and were observed in CXCL7 knockout primary cells from animals that had received intravenous administration of human recombinant CXCL7. In addition, no expression of p38 MAPK was observed in the CXCL7 knockout primary cells by western blotting (S3G Fig).

*CXCL7*-null mice showed ligament ossification and expression of BMP-2, whereas bone morphometry measurement suggested that osteoporosis was present; consequently, calcium signaling was investigated. Consistently, higher levels of proteasome subunit alpha type-2 (PSMA2), ubiquitin protein ligase E3C (UBE3C), and Ca^2+^/calmodulin-dependent protein kinase II (CaMK II) were detected in the sera of patients with OPLL (Fig 3H). The levels of CaMK II were examined because bone morphometry measurements suggested that osteoclast activity may be affected and CamK II is known to be involved in osteoclast regulation [43].

RNA and microRNA were obtained from blood obtained from OPLL patients. The data from the microRNA array analysis showed that miR-340 is associated with the CXCL7 deficiency found between OPLL patients and healthy subjects (S3H Fig and S2 Dataset. Supplementary Dataset GSE57592.pdf). miR-340 did not induce the degradation of CXCL7 in primary cells derived from the spine ligament of healthy mice (S3K Fig), suggesting that this mechanism is not responsible for the decreased levels of CXCL7 seen in OPLL. We also performed a kidney tissue RNA array analysis to determine the molecular mechanisms underlying *CXCL7* deficiency-induced OPLL. Factors involved in gluconeogenesis, acquisition of natural immunity, and MAPK signaling were highly represented in the DAVID pathway analysis (v 6.7; https://david.ncifcrf.gov/) (Table 3 and S1 Dataset. Supplementary Dataset GSE57590.pdf). In summary, the gene expression changes showed large changes in cytokine-cytokine receptor interactions involved in various cytokine signaling pathways, MAPK signaling pathways, the p53 signaling pathway, and the regulation of glycolysis and gluconeogenesis.

**Table 3:** Kyoto encyclopedia of genes and genomes pathway analysis. Data from renal tissue genomic arrays of wild-type and *CXCL7*-null mice were analyzed using DAVID software.

Term	Count
NOD-like receptor signaling pathway	11
Cytokine-cytokine receptor interaction	20
Chemokine signaling pathway	14
Intestinal immune network for IgA production	7
Toll-like receptor signaling pathway	9
Arachidonic acid metabolism	8
Hematopoietic cell lineage	8
MAPK signaling pathway	16
Prion diseases	5
Drug metabolism	7
T cell receptor signaling pathway	9
Graft-versus-host disease	6
Glycolysis/gluconeogenesis	6
p53 signaling pathway	6
Primary immunodeficiency	4

### In vitro ossification induced by shRNA-CXCL7 expression increases osteoblast and osteoclast calcification

**Fig 4:**
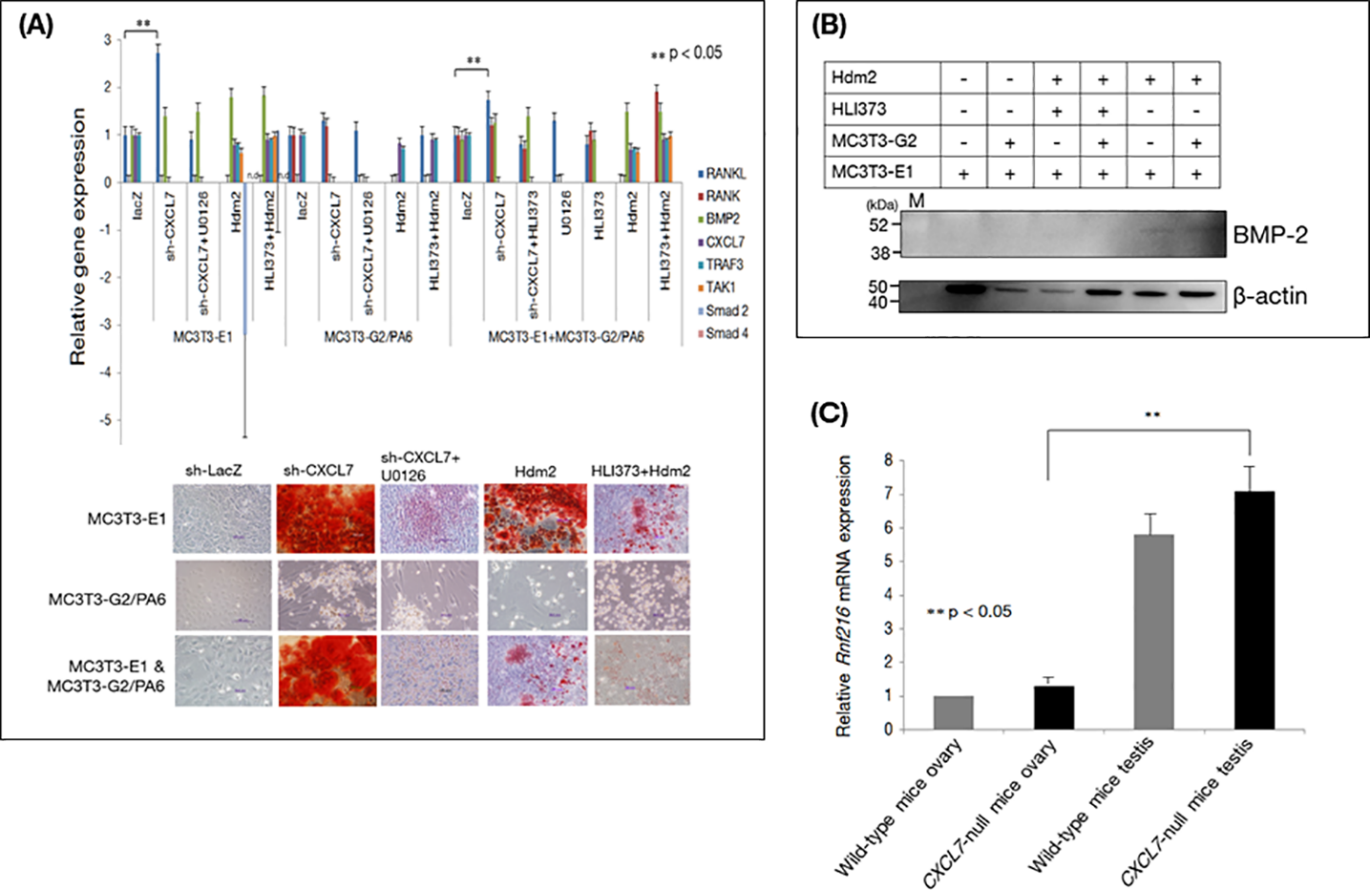
Mechanism underlying the effects of CXCL7 downregulation. (**A)** Upper panel. Real-time PCR analysis of osteogenic genes expressed in the osteoblastic cell line MC3T3-E1, the MC3T3-G2/PA6 preadipocyte cell line, and a co-culture of the two. MC3T3-E1 osteoblasts or MC3T3-G2/PA6 preadipocytes were transfected with shRNA-LacZ or shRNA-CXCL7 after which the shRNA-CXCL7-transfected cells were treated with the MEK inhibitor U0126 (10 μM for 15 min), Hdm2 (1 μM for 30 min), or HLI 373 (5 μM for 8 h). Lower panel. Staining of calcified nodules. Cells from the treatments shown in the upper panel were stained to detect calcification, as shown in red. Calcification was confirmed in MC3T3-E1 osteoblasts expressing sh-*CXCL7* and following Hdm2 administration. (**B**) BMP-2 levels in cultures of MC3T3-E1 osteoblasts, MC3T3-G2/PA6 preadipocytes, or co-cultures of both, treated or not with recombinant Hdm2 (1 μM for 30 min) or HL1 373 (5 μM for 8 h). Similarly, even following western blotting, BMP-2 expression was enhanced following sh-RNA-CXCL7 transfection in osteoblastic MC3T3-E1 cells, and in addition BMP-2 levels were also increased following administration of Hdm2. Sex differences in OPLL. **(C**) Ovarian and testicular expression of ring finger protein (Rnf)216 in wild-type and *CXCL7*-null mice (each, n = 4).

Based on the data obtained from the *CXCL7*-null mice and patient OPLL data, we observed an induction of ligament ossification, as well as evidence for osteoporosis. Consequently, we investigated bone metabolism using MC3T3-E1 cells and MC3T3-G2/PA6 preadipocytes, which have been used extensively for bone metabolism research. Expression of sh-RNA-*CXCL7* in MC3T3-E1 osteoblasts and MC3T3-G2/PA6 showed that CXCL7 depletion resulted in calcification [44, 45], whereas HLI 373 addition reduced calcification (Fig 4A), In addition, BMP-2 levels were increased by sh-RNACXCL7, whereas administration of the MEK inhibitor U0126 decreased BMP-2 levels in MC3T3-E1 osteoblasts and MC3T3-G2/PA6 preadipocytes [46]. Furthermore, BMP-2 expression was induced by addition of recombinant Hdm2 (Fig 4B and S4A Fig). In equine bone marrow-derived stem cells prepared from ligament tissue and human and mouse mesenchymal stem cells [47], CXCL7 knockdown induced BMP-2 expression (S4B and S4C Fig). Knockdown of CXCL7 in a co-culture of MC3T3-E1 osteoblasts and MC3T3-G2/PA6 preadipocytes resulted in up-regulation of RANKL-RANK. When co-cultures expressing sh-CXCL7 were treated with U0126, the osteoblasts were found to express RANKL and BMP-2, and the MC3T3-G2/PA6 preadipocytes were found to express RANKL, and these effects were not blocked in the presence of a MEK inhibitor, indicating that CXCL7 may phosphorylate MAPK without signaling through MEK.

### Influence of sex differences in OPLL

Based on these data, we hypothesized that the ubiquitin-proteasome induces chemokine degradation in OPLL. Given the known sex differences in the development of OPLL, the expression of the ring-finger protein (Rnf)216 (https://www.genenames.org/cgi-bin/genefamilies/set/58), which encodes a ubiquitin E3 ligase (http://www.ncbi.nlm.nih.gov/gene/54476) [35, 48], was examined in the testes and ovaries of *CXCL7*-null mice. A significant increase in *Rnf216* expression was detected in the ovaries and testes of *CXCL7*-null mice compared to that in tissues from wild-type mice (Fig 4C). These data suggest a relationship between sex and OPLL.

## Conclusions

In this study, a proteomics analysis of sera from patients with OPLL revealed that CXCL7 is significantly downregulated, suggesting that CXCL7 loss is involved in the development of OPLL. *CXCL7*-null mice exhibited spinal ligament ossification and many symptomatic features of human OPLL, and presented with diabetes and severe obesity. Continuous and segmental OPLL were observed in most *CXCL7* knockout mice, including one case of OPLL in the cervical vertebra, and seven and 13 cases in the thoracic and lumbar vertebrae, respectively. In rare cases, OPLL has been described as a combination of ossification of the yellow ligament (OYL) and dural ossification (DO) [49]. However, OYL was not observed in *CXCL7*-null mice. This is likely related to the fact that the YL is composed of elastic fibers primarily composed of elastin, whereas the PLL is composed of collagen fibers primarily comprised of collagen [50]. All mutant mice exhibited type I diabetes with insulin deficiency, and some mice experienced other complications depending on the location of the OPLL. Female *CXCL7*-null mice with OPLL in T12 presented with bilateral hydro-nephrosis and exophthalmos, whereas female and male mice with OPLL in C3 showed a thinning of the retinal IPL and lens fiber cell proliferation. Epithelial cells from the kidney glomerulus had proliferated, and there was a lower calcification rate in cancellous bone, similar to what is observed in the human disease. The cortical bone also exhibited thinning along the width of the lateral spinal cord, similar to human ectopic ossification [51]. In summary, these observations demonstrate that *CXCL7*-null mice are an ideal model of human OPLL.

Serum CXCL7 protein levels were lower in patients with continuous OPLL than in patients with mixed subtype OPLL. Given that the former is associated with more severe clinical symptoms, this finding suggests that CXCL7 insufficiency is a causal factor in OPLL. BMD is known to be lower in patients with OPLL than in healthy subjects [5, 6]. Similar features were observed in *CXCL7*-null mice, consistent with high electrolyte levels. *CXCL7*-null mice developed type I diabetes, consistent with the effects of insulin deficiency on bone tissue [52]. Concomitant with decreased insulin secretion, cAMP levels were increased in *CXCL7-*null mice, resulting in high blood inorganic phosphate levels [53]. This effect was associated with OPLL features such as loss of bone mass and decreased cortical bone width. *CXCL7*-null mutants also exhibited elevated glucagon expression in pancreatic α cells, reflective of increased blood glucose levels [54]. An increase in the glomerular mesangial matrix has been observed in patients with type I diabetes mellitus [55, 56]. In *CXCL7*-null mice, this is a symptom of diabetic nephropathy that can potentially progress to glomerulosclerosis. Western blotting of primary cells derived from the spinal ligament tissues of wild-type and CXCL7 knockout mice showed that UCHL1 and UBE3C expression was absent in wild-type, but positive in CXCL7 knockout primary cells (S3G Fig). In wild-type primary cells, expression of MAPK-SAPK/JNK and nuclear translocation of CXCL7 were shown by immunocytochemical staining, and these effects were also observed in CXCL7 knockout primary cells derived from CXCL7 knockout mice that had received an intravenous administration of human recombinant CXCL7. We have previously reported that MAP3K is expressed in mesenchymal stem cell-derived ligament-like tissue [47]. Data obtained from a kidney tissue RNA array analysis of *CXCL7*-mice are consistent with the induction of autophagy genes in response to p53 activation [57]. These data are consistent with the bone morphogenetic and biochemical results, which indicate that PKC phosphorylation and activation allow osteoclasts to be regulated by calcium ion release and subsequent Ca^2+^/CaMK II signaling, resulting in the onset of osteoporosis and generation of cAMP as a result of activation of the Ac complex [58]. PKC has been long known to be able to phosphorylate glucose transporter-1 [59], and BMP-2-induced apoptosis in human osteoblasts is also mediated by PKC activation [60].

*CXCL7*-null mice exhibited heterotopic ossification in posterior ligament tissue and osteoporosis in vertebrate tissue. Regarding the mechanism, CXCL7 is known to promote osteoclast proliferation [61]. During the process of chemokine degradation by the ubiquitin-proteasome in *CXCL7*-null mice, TRAF3 and MEKK3 (https://meshb.nlm.nih.gov/record/ui?name=MAP%20Kinase%20Kinase%20Kinases, http://www.uniprot.org/uniprot/A2AQW0) act on the E3 ubiquitin ligase. When E3 was inhibited by addition of HLI 373 to primary cultures of spinal ligament tissues from *CXCL7*-null mice, the target chemokine was translocated to the nucleus by activation of MAPK-SAPK/JNK. Because osteoblasts transfected with sh-CXCL7 and treated with the MEK inhibitor showed an increase in both RANKL and BMP expression, it is possible that CXCL7 may phosphorylate MAPK without going through MEK. Posterior longitudinal ligament ossification observed in *CXCL7*-null mice is thought to be caused by osteoclast dysfunction owing to a decrease in TRAF6. In addition, complications that occur in diabetes and obesity of OPLL are suggested to be caused by the decreased transmission of TRAF6 to TAK1 [62–65]. Among the DUB enzymes, DUB has been shown to remove the K63 polyubiquitin chain of TRAF6 and inhibit protein degradation from K48 polyubiquitination to suppress NF-κB activity [41, 66–68]. This report suggests that the degradation of CXCL7 in this study is due to K48 polyubiquitination, which is a function of ubiquitinating enzymes (UBE). The deficiency in CXCL7 due to PKC phosphorylation leads to cAMP and CaMK II activation, subsequent Ca^2+^ release, and consequent osteoporosis and osteoclast reduction in spinal ligament tissues owing to the reduction of TRAF6-TAK1 transmission in obesity and diabetes.

In conclusion, CXCL7 deficiency in OPLL is suggestive of target degradation by the ubiquitin-proteasome by phosphorylation of E3 ubiquitin ligase and K48 polyubiquitination.

## Supporting information

S1 File Supplementary protocols and methods. (PDF)

S1 Fig Analysis of serum target proteins in OPLL. (PDF)

S2 Fig Generation of *CXCL7*-null mice and determination of bone histomorphometric values in OPLL. (PDF)

S3 Fig Defective system of target CXCL7 protein. (PDF)

S4 Fig Ossification by CXCL7 knockdown in equine mesenchymal stem cells. Verification of the background for CXCL7 protein deficiency.
(PDF)

S1 Table Abbreviations of the histomorphometric parameters according to the American Society for Bone and Mineral Research: ASBMR nomenclature committee. (PDF)

S2 Table Abbreviations (Fig 2S). (PDF)

S1 Video A four-month-old male *CXCL7*-null mouse walking. DOI; dx.doi.org/10.17504/protocols.io.j9mcr46.
(MP4)

S2 Video A four-month-old male wild-type mouse swimming. DOI; dx.doi.org/10.17504/protocols.io.j9ncr5e.
(MP4)

S3 Video A four-month-old male *CXCL7*-null mouse swimming. DOI; dx.doi.org/10.17504/protocols.io.j9pcr5n.
(MOV)

S1 Dataset Supplementary Dataset GSE57590. (PDF)

S2 Dataset Supplementary Dataset GSE57592. (PDF)

S1 Data Supplementary SNP data. DOI; dx.doi.org/10.17504/protocols.io.j9gcr3w.
(PDF)

S2 Data Supplementary conditional *Ppbp*^*dE2E3/+*^ knockout mouse data. (PDF)
