# Supplementary material for: Ubiquitin-dependent proteolysis of CXCL7 leads to posterior longitudinal ligament ossification
Source: PLoS One. 2018 May 21;13(5):e0196204. doi: 10.1371/journal.pone.0196204 (PMC5962073; doi:10.1371/journal.pone.0196204)
Supplement: S1 File — (PDF) [file pone.0196204.s001.pdf]

Supporting Information

Ubiquitin-dependent proteolysis of CXCL7 leads to posterior longitudinal ligament ossification

Michiyo Tsuru, Atsushi Ono, Hideaki Umeyama, Masahiro Takeuchi and Kensei Nagata

SUPPLEMENTARY PROTOCOLS AND METHODS

S1 File. Supplementary protocols and methods

Supplementary protocols and methods

Primary cell cultures

Primary cell cultures were obtained by treating spinal tissue from *CXCL7*-null and wild type mice (4 males and 3 females; mean age: 1 year for each group) with 0.2% collagenase type X (Wako Pure Chemical Industries, Osaka, Japan) in Hank's balanced salt solution for 60 min. The first two digests were discarded and cells from the final digest were re-suspended in Dulbecco's modified Eagle's medium (Life Technologies) supplemented with 10% heat-inactivated fetal bovine serum (JRH Bioscience, Lenexa, KS, USA), 2 mM L-glutamine, 5% gentamicin, 100 U/mL penicillin, 100 μg/mL streptomycin, and 0.25 μg/mL amphotericin. Cells were cultured for 2–4 days in a humidified atmosphere of 5% CO_2 and 95% air at 37°C in 35-cm^2 flasks until they reached confluence. *CXCL7*-null mice were injected intravenously with human recombinant CXCL7 (1 mg/kg/week) and ossified tissues were excised for preparation of primary cultures. Micro (mi) RNA (1 μg /well) was transfected into primary cells using Lipofectamine RNAiMAX (Thermo Fisher Scientific, Waltham, CA, USA). We generated a miR-340 probe by tagging Has-miR-340 aatcaG(L)t5(L)aT(L)tG(L)cT(L)ttataa_N(6)_Y with Alexa Fluor 488. Fluorescence was visualized in the cells following treatment with 5 μM 1,4-diamino-2,3-dicyano-1,4-bis [2-aminophenylthio]butadiene (U0126, #9903; Cell Signaling Technology, Danvers, MA, USA) for 30 min, 5 μM HLI 373 (Tocris Bioscience/R&D Systems, Minneapolis, MN, USA) for 8 h, or 1 μM E3 ubiquitin ligase Hdm2 (Enzo Life Sciences, Farmingdale, NY, USA) for 30 min.

Cell culture

MC3T3-E1 (RCB1126) osteoblasts, MC3T3-G2/PA6 (RCB1127) preadipocytes, and C2C12 (RCB0987) myoblasts were obtained from the RIKEN Bioresource Center Cell Bank (Wako, Osaka, Japan). Cell counts were performed with an automated cell counter (TC10; Bio-Rad, Hercules, CA, USA).

Immunostaining

We obtained 5-µm-thick sections of ossification ligament, pancreas, fat, and testis from the *CXCL7* knockout mice and performed fixation with 4% paraformaldehyde. Primary antibodies against BMP-2 (GeneTex, Irvine, CA, USA), ubiquitin protein ligase E3C (ab68225), calcium/calmodulin-dependent protein kinase II (ab22609), ubiquitin (linkage-specific K48) (ab140601) (Abcam, Cambridge, MA, USA), insulin (#4590), glucagon (#2760), and ubiquitin C-terminal hydrolase L1 (#3524) (all of the latter from Cell Signaling Technology, Danvers, MA, USA) were detected with secondary antibodies tagged with Alexa Fluor 488 or Alexa Fluor 555, and nuclei were counterstained with DAPI (Dojindo Laboratories, Kumamoto, Japan). After staining, the cells and tissues were observed under a fluorescence microscope (BZ-X700, Keyence, Osaka, Japan).

ELISA

We used ELISA kits to measure the levels of CXCL7 (Adipo Bioscience, Santa Clara, CA, USA) and UCHL1 (Medical & Biological Laboratories, Aichi, Japan) in the sera from healthy subjects and patients with OPLL.

Quantitative real-time PCR (qRT-PCR)

Real-time PCR was performed using a TaqMan Gene Expression Assay kit with a StepOnePlus real-time PCR system. The mRNA expression levels of the treated samples relative to the control were calculated from the difference in threshold cycle (comparative CT [ΔΔCT] method; [1]) and are presented as the average of triplicate experiments with a 95% confidence interval. The resultant cDNAs were diluted 20 × before qRT-PCR. The TaqMan probes used were: MEKK3, Mm00803725_m1; Rnf216, Mm01205634_m1;TRAF3, Mm00495752_m1; TAK1, Mm00554514_m1; NFκB, Mm00477800_s1; RANK, Smad2, Mm00487530_m1; Smad4, Mm03023996_m1; BMP-2, Mm01340178_m1; RANKL, Mm00441906_m1; CXCL7, Mm01347901_g1; TRAF6, Mm; 00493836_m1; mouse collagen, type I, alpha 1 Col1a1: Mm00801666_g1; human collagen, type I, alpha 2: Hs00164099_m1; PRELP, Hs01941580_s1; UCHL1, Mm00495900_m1; and 18S, Mm03928990_g1 or human eukaryotic 18S rRNA Hs99999901_s1.

Flow cytometry

MSCs were separated from equine cerebrospinal fluid and transfected with the pCAGGS-*PRELP* vector [2]; in addition, single cells were obtained from a ligament like tissue sample and transfected with shRNA-*CXCL7* one day later. An antibody targeting BMP-2 (GeneTex) was used to assess BMP-2 expression by fluorescence-activated cell sorting (FACS; FACS Canto II, Becton Dickinson, Franklin Lakes, NJ, USA). Stem cells transfected with the shRNA-*CXCL7* were compared to cells transfected with sh-scramble and sh-LacZ and compared to cells expressing the

pCAGGS-*PRELP* vector. We observed high levels of BMP-2 expression, confirming ossification of the ligament.

Immunoprecipitation

Spinal ligament primary cells of wild type and *CXCL7*-null mice and sera of healthy subjects and OPLL patients were immunoprecipitated with anti-ubiquitin (linkage-specific K48 antibody (ab140601) (Abcam) bound to Dynabeads protein A. A Hula sample mixer (Thermo Fisher Scientific) was used according to the recommended protocol for the Dynabeads Protein A immunoprecipitation kit (Thermo Fisher Scientific). Next, 10 μg of the K48 antibody (1.429 mg/mL) was used for preparing the Dynabeads Protein A 50 µl-bound anti-K48 bound for 30 minutes. The bound antibodies were then mixed with NuPAGE LDS sample buffer/NuPAGE Reducing agent mix (Thermo Fisher Scientific) and incubated at 70°C for 10 minutes. The Dynabeads Protein A-bound K48 antigen-antibody complex was finally extracted using the DynaMag™-2 magnet (Thermo Fisher Scientific), washed 3 times with wash buffer, and then analyzed by western blotting [3]. As a control, PBS was added to the mixture of Dynabeads Protein A magnetic beads and K48 antibodies.

Separation of white blood cells

Blood from healthy volunteers and OPLL patients was collected in PAXgene blood collection tubes (Becton Drive, Franklin Lakes, NJ, USA) and stored frozen at −80°C. Thawed samples were thawed at room temperature for 2 hours at room temperature and centrifuged at 4300 × *g* for 10 min in a swing rotor centrifuge (Kubota 5911, Tokyo, Japan; http://www.centrifuge.jp/products/model-5911/). The supernatant

was discarded, sterilized water was added to the pellets, and the erythrocytes were hemolyzed. The suspension was then centrifuged at 4300 g for 10 minutes, the supernatant was discarded, and white blood cells were extracted into the resuspension solution.

Western blotting

Cell lysates were subjected to SDS-PAGE and proteins were then transferred to a polyvinylidene difluoride membrane (0.4 A for 1.0 h at 10°C), which was then incubated in a blocking solution for 1 h at room temperature and then probed for 16 h at 4°C with antibodies against the following proteins: calmodulin-dependent protein kinase II (ab22609), tumor necrosis factor receptor-associated factor (TRAF)3 (ab110715), Toll-like receptor 3 (ab62566), mitogen-associated protein kinase kinase kinase (MAP3K)3 (ab40750), receptor activator of nuclear factor κB ligand (RANKL) (ab9957), CXCL7 (ab58142), ubiquitin protein ligase E3C (ab68225), ubiquitin (linkage-specific K48) (ab140601) and β-actin (ab8227) (Abcam); RANK (#4845), proteasome 26S subunit, non-ATPase 4 (PSMD4) (#12441), ubiquitin C-terminal hydrolase L1 (#3524), phospho-p44/42 MAPK (#4370), phospho-p38 MAPK (#4511), phospho-stress-associated protein kinase/c-Jun N-terminal kinase MAPK (#4668), TRAF6 (#8028), transforming growth factor β-activated kinase 1 (#9339), ubiquitin (#3933), SQSTM1/p62 (#5114), nuclear factor κB (#8242), and proteasome subunit alpha type-2 (#2455) (Cell Signaling Technology); and bone morphogenetic protein 2 (no. 103626) (GeneTex). After washing with 0.05% Tween-20 in phosphate-buffered saline (PBS), the membrane was probed with the appropriate secondary antibody for 1 h at room temperature, washed with 0.05% Tween-20 in PBS, and processed using the

ECL Advanced WB Analysis System (GE Healthcare Life Sciences, Amersham, UK). The ChemiDoc Touch system (Bio-Rad) was used to visualize protein bands, which were then analyzed with imaging software (Bio-Rad).

3D CT

3D micro X-ray CT analysis was performed per the manufacturer's instructions (CT StellaScan AX; https://www.rigaku.com/ja/products/xrm/stellascan_ax) (Rigaku, Tokyo, Japan).

Supplementary Discussion

Many OPLL patients develop diabetes and their blood sugar must be controlled prior to surgery. A lack of chemokines is associated with the development of OPLL, but the underlying mechanism is unclear, given that no abnormalities have been observed by exon sequencing or SNP analysis. Our microRNA analysis revealed that high miR-340 levels are present in OPLL patients compared to the levels in healthy subjects (Fig. 1L, S2 Fig. E and Supplementary Dataset GSE57592-pone17-25580.pdf). Furthermore, a proteome phosphorylation analysis was performed because of the inability to establish a direct correlation with the lack of the CXCL7 protein by SNP and microRNA. CXC chemokines bearing the glutamic acid-leucine-arginine (ELR) motif are crucial mediators in neutrophil-dependent acute inflammation. NAP-2/CXCL7 is an NH2-terminally processed form of β-thromboglobulin (β-TG), which in turn is a cleavage product of the platelet basic protein (PBP) precursor derived from platelets, along with connective-tissue activating peptide-III (CTAP-III) and PBP [4]. We

performed a kidney tissue RNA array analysis to determine the molecular mechanisms underlying OPLL caused by *CXCL7* deficiency. Factors involved in gluconeogenesis, the acquisition of natural immunity, and MAPK and p53 signaling were highly represented in the DAVID pathway analysis v6.7 (https://david.ncifcrf.gov/) (Table 3). These results are consistent with the notion that induction of autophagy genes in response to p53 activation is associated with enhanced autophagy in diverse settings and depends on p53 transcriptional activity [5]**.** During chemokine degradation by the ubiquitin-proteasome system in *CXCL7*-null mice, the expression of Toll-like receptor 3, signaling through the downstream tumor necrosis factor receptor-associated factor (TRAF)3 and MAPK kinase kinase (MEKK)3, resulted in a modulation of E3 ubiquitin ligase activity.–Posterior longitudinal ligament ossification observed in *CXCL7*-null mice is thought to be caused by osteoclast dysfunction owing to a decrease in TRAF6 [6–8]. Among the de-ubiquitinating (DUB) enzymes, DUB has been shown to removes the K63 polyubiquitin chain of TRAF6 and inhibits protein degradation from K48 polyubiquitination to suppress NF-κB activity [9–11]. This report suggests that the degradation of CXCL7 in this study is due to K48 polyubiquitination, which is a function of deubiquitinating enzymes. Based on the present data, under normal conditions, we suggest that CXCL7 is stabilized in the cell as a result of de-ubiquitination by UCHL1. In the process of chemokine degradation by the ubiquitin-proteasome in *CXCL7*-null mice, TRAF3 and MEKK3 act on the E3 ubiquitin ligase. When the latter was inhibited by addition of HLI 373 to the cells in primary cultures of spinal ligament tissues of *CXCL7*-null mice, the target chemokine was translocated to the nucleus as a result of MAPK-SAPK/JNK signaling, and the expression of the ubiquitin chain-binding PSMD4 of proteasome 26S was increased.

Our findings provide insight into the molecular basis of OPLL and suggest that increasing CXCL7 levels in patients could serve as an effective therapeutic strategy for the treatment of this disease.

DUB UCHL1 is strongly up-regulated, and inhibition of UCHL1 has been found to exacerbate rather than ameliorate the disease in a mouse model of spinal muscular atrophy (SMA) [12]. Although complex interactions of genetic and environmental factors underlie human neurodegenerative diseases, many of these conditions are suggested to share a common molecular signature: collapse of protein homeostasis [13, 14].

Table legends

Table 1. Body weight, volume ratios of subcutaneous and visceral fat, and biochemical data for wild-type and *CXCL7*-null mice.

Table 2. Bone histomorphometric analysis of wild-type and *CXCL7*-null mice. Parameters for cortical bone were measured at the midpoint of the tibia. Data are expressed as the mean ± SEM of eight bones/group. Abbreviations related to osteocyte parameters in Table 2 are described in S1 Table.

Table 3. Kyoto Encyclopedia of Genes and Genomes pathway analysis. Data from renal tissue genomic arrays of wild-type and *CXCL7*-null mice were analyzed using DAVID software.

Video legends

Video 1. A four-month-old male *CXCL7*-null mouse walking.

DOI; dx.doi.org/10.17504/protocols.io.j9mcr46

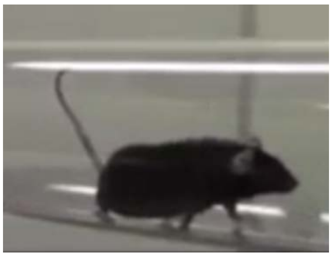

Video 2. A four-month-old male wild-type mouse swimming. The water temperature was 34°C.

DOI; dx.doi.org/10.17504/protocols.io.j9ncr5e

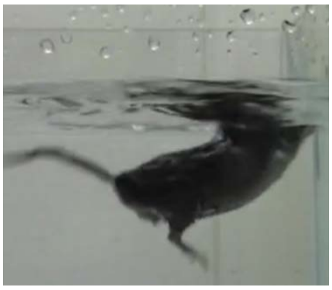

Video 3. A four-month-old male *CXCL7*-null mouse swimming. The water temperature was 34°C.

DOI; dx.doi.org/10.17504/protocols.io.j9pcr5n

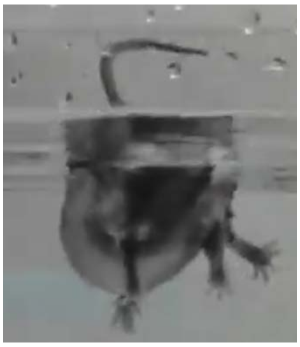

As a functional evaluation of the limbs in *CXCL7* knockout mice, we present here videos of the animals walking and swimming. For the walking analysis, an

appropriately sized transparent cylinder was used, with both ends being sealed with a film containing air holes to prevent the animals from escaping. For the swimming analysis, it was necessary to evaluate limb function to detect any differences from a healthy mouse. A tank with an optimum depth and width so as not to stress the animals, was prepared. The water temperature was kept at 34°C, and the mouse was allowed to float for only 17 seconds. In addition, the laboratory room temperature was maintained at 27°C. After the experiment, a hot air dryer was used to quickly dry the animals to keep them in a good physical state.

References

1. Livak KJ, Schmittgen TD. Analysis of relative gene expression data using real-time quantitative PCR and the 2(-Delta Delta C(T)) Method. Methods. 2001 ;25:402–408.
2. Tsuru M, Soejima T, Shiba N, Kimura K, Sato K, Toyama Y, et al. Proline/arginine-rich end leucine-rich repeat protein converts stem cells to ligament tissue and Zn(II) influences its nuclear expression. Stem Cells Dev. 2013;22: 2057–2070.
3. Ansell SM, Hodge LS, Secreto FJ, Manske M, Braggio E, Price-Troska T, et al. Activation of TAK1 by MYD88 L265P drives malignant B-cell growth in non-Hodgkin lymphoma. Blood Cancer J. 2014; 4:e183.
4. Brandt E, Petersen F, Ludwig A, Ehlert JE, Bock L, Flad HD. The beta-thromboglobulins and platelet factor 4: blood platelet-derived CXC chemokines with divergent roles in early neutrophil regulation. J Leukoc Biol. 2000; 67: 471–478.
5. Rosenfeldt MT, O'Prey J, Morton JP, Nixon C, MacKay G, Mrowinska A, et al. p53 status determines the role of autophagy in pancreatic tumour development. Nature. 2013;504: 296–300.
6. Naito A, Azuma S, Tanaka S, Miyazaki T, Takaki S, Takatsu K, et al. Severe osteopetrosis, defective interleukin-1 signalling and lymph node organogenesis in TRAF6-deficient mice. Genes Cells. 1999; 4:353–362.
7. Paul PK, Kumar A. TRAF6 coordinates the activation of autophagy and ubiquitin-proteasome systems in atrophying skeletal muscle. Autophagy. 2011;7:

555–556.

8. Mao AP, Li S, Zhong B, Li Y, Yan J, Li Q, et al. Virus-triggered ubiquitination of TRAF3/6 by cIAP1/2 is essential for induction of interferon-beta (IFN-beta) and cellular antiviral response. J Biol Chem. 2010;285: 9470–9476.
9. Chen ZJ, Sun LJ. Nonproteolytic functions of ubiquitin in cell signaling. Mol Cell. 2009; 33:275–286.
10. Wertz IE, O'Rourke KM, Zhou H, Eby M, Aravind L, Seshagiri S, et al. De-ubiquitination and ubiquitin ligase domains of A20 downregulate NF-kappaB signalling. Nature. 2004; 430: 694–699.
11. Kayagaki N, Phung Q, Chan S, Chaudhari R, Quan C, O'Rourke KM, et al. DUBA: a deubiquitinase that regulates type I interferon production. Science. 2007; 318:1628–1632.
12. Powis RA, Mutsaers CA, Wishart TM, Hunter G, Wirth B, Gillingwater TH. Increased levels of UCHL1 are a compensatory response to disrupted ubiquitin homeostasis in spinal muscular atrophy and do not represent a viable therapeutic target. Neuropathol. Appl. Neurobiol. 2014; 40: 873–887.
13. Rubinsztein DC. The roles of intracellular protein-degradation pathways in neurodegeneration. Nature. 2006; 443:780–786.
14. Groen EJN, Gillingwater TH. UBA1: At the crossroads of ubiquitin homeostasis and neurodegeneration. Trends Mol Med. 2015;21:622–632.
