# Supplementary material for: Ubiquitin-dependent proteolysis of CXCL7 leads to posterior longitudinal ligament ossification
Source: PLoS One. 2018 May 21;13(5):e0196204. doi: 10.1371/journal.pone.0196204 (PMC5962073; doi:10.1371/journal.pone.0196204)
Supplement: S1 Fig — (PDF) [file pone.0196204.s002.pdf]

Supporting Information

Ubiquitin-dependent proteolysis of CXCL7 leads to posterior longitudinal ligament ossification

Michiyo Tsuru, Atsushi Ono, Hideaki Umeyama, Masahiro Takeuchi and Kensei Nagata

SUPPLEMENTAL FIGURES

S1 Fig. Analysis of serum target proteins in OPLL.

S1 Fig.

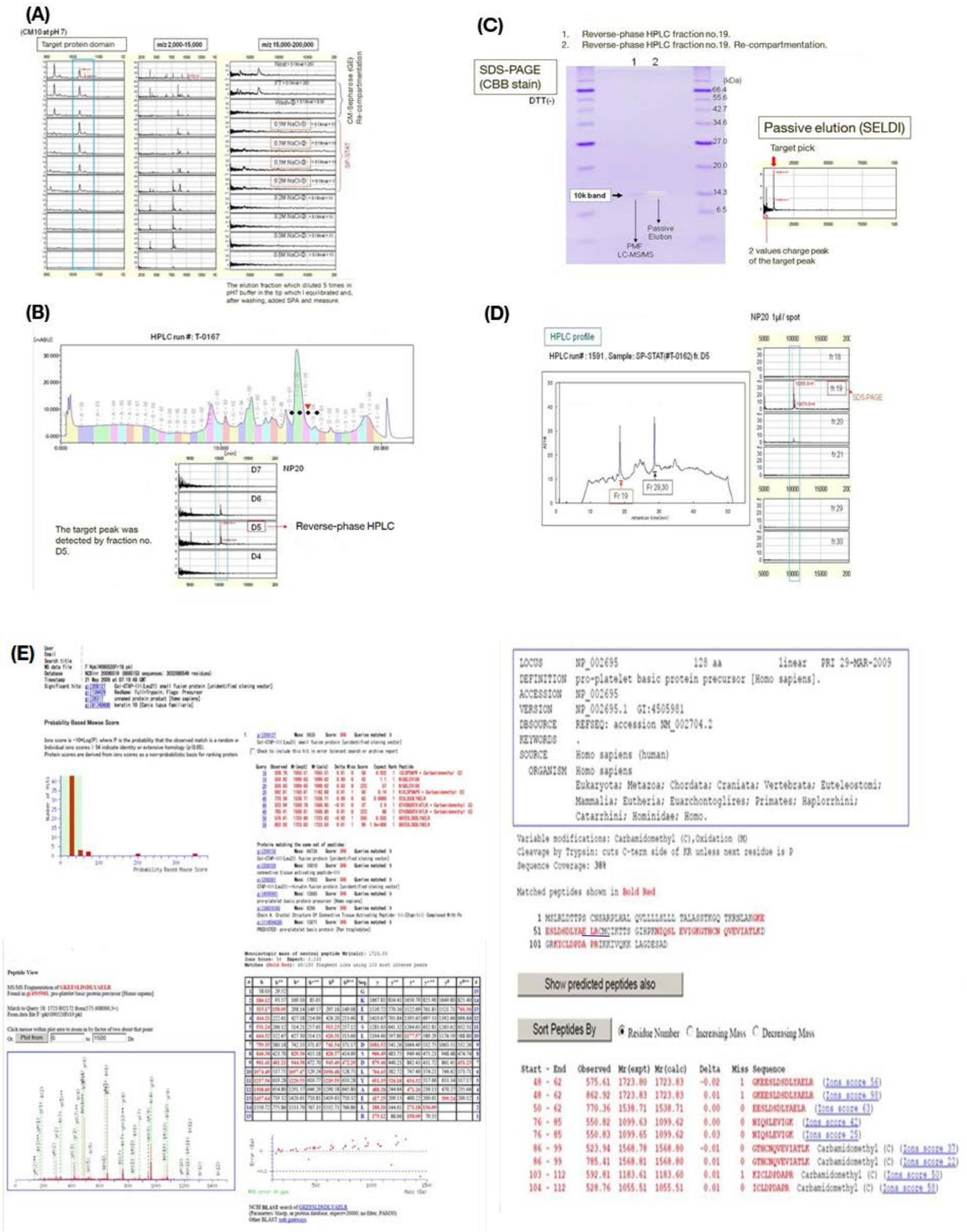

(**F**) >Kurume_Sequence_2_1 #gi 4505981|ref|NP_002695.1| platelet basic protein preproprotein [Homo sapiens]

MSLRLDTTPSCNSARPLHALQVLLLLSLLLTALASSTKGQTKRNLAKGKEESLDS
DLYAELRCMCIKTTSGIHPKNIQSLEVIGKGTHCNQVEVIATLKDGRKICLDPDAP
RIKKIVQKKLAGDESAD

A model of the sequence of “NLAK GKEESLDSDLYAELR CMCIKTTSGIHPK NIQSLEVIGKGTHCNQVEVIATLKDGRKICLDPDAPR IKKIVQKKLAG” was prepared with reference to the A chain of PDB code 1F9P.

1F9P_A
AUTHOR
J.YANG,T.FAULK,R.ASTER,G.VISENTIN,B.EDWARDS,C.CASTOR
REVDAT 1 26-AUG-03 1F9P 0
JRNL AUTH J.YANG,T.FAULK,R.ASTER,G.VISENTIN,B.EDWARDS,
JRNL AUTH 2 C.CASTOR
JRNL TITL STRUCTURE OF THE CXC CHEMOKINE, CONNECTIVE
TISSUE
JRNL TITL 2 ACTIVATING PEPTIDE-III, COMPLEXED WITH THE
HEPARIN
JRNL TITL 3 ANALOGUE, POLYVINYLSULFONIC ACID
JRNL REF TO BE PUBLISHED
JRNL REFN

81 amino acid residues homology 100% based on 1 F 9 P A chain

NLAK
GKEESLDSDLYAELR Gly5-Arg19
CMCIKTTSGIHPK
NIQSLEVIGKGTHCNQVEVIATLKDGRKICLDPDAPR ASN33-ARG69
IKKIVQKKLAG

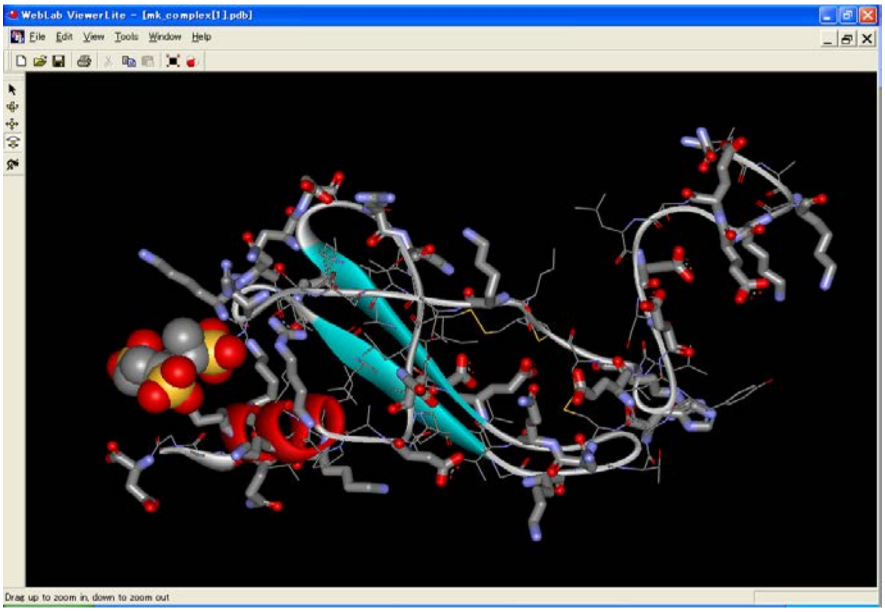

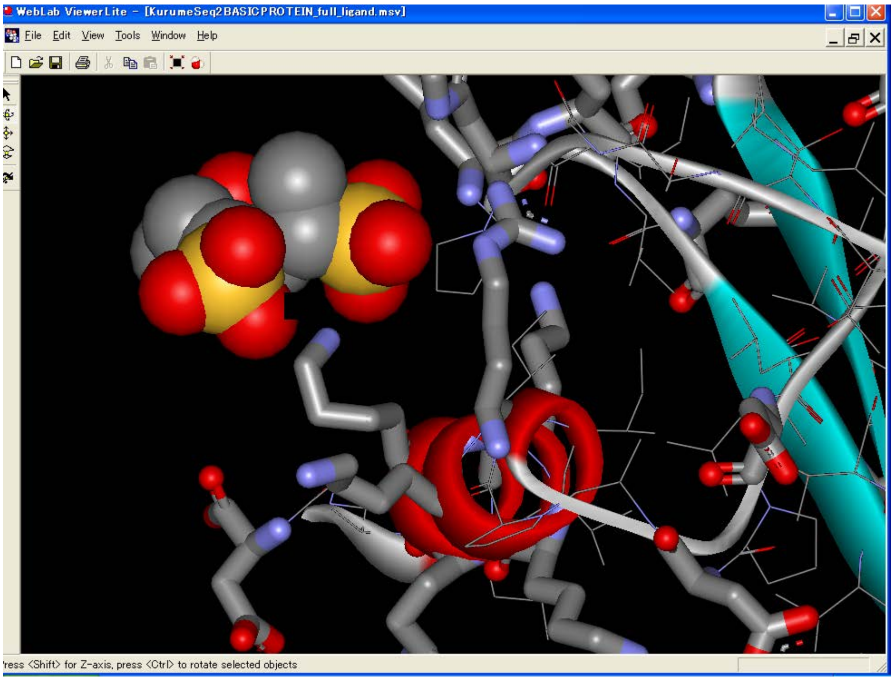

The binding pocket of HEPARIN ANALOGUE, POLYVINYLSULFONIC ACID, is indicated.

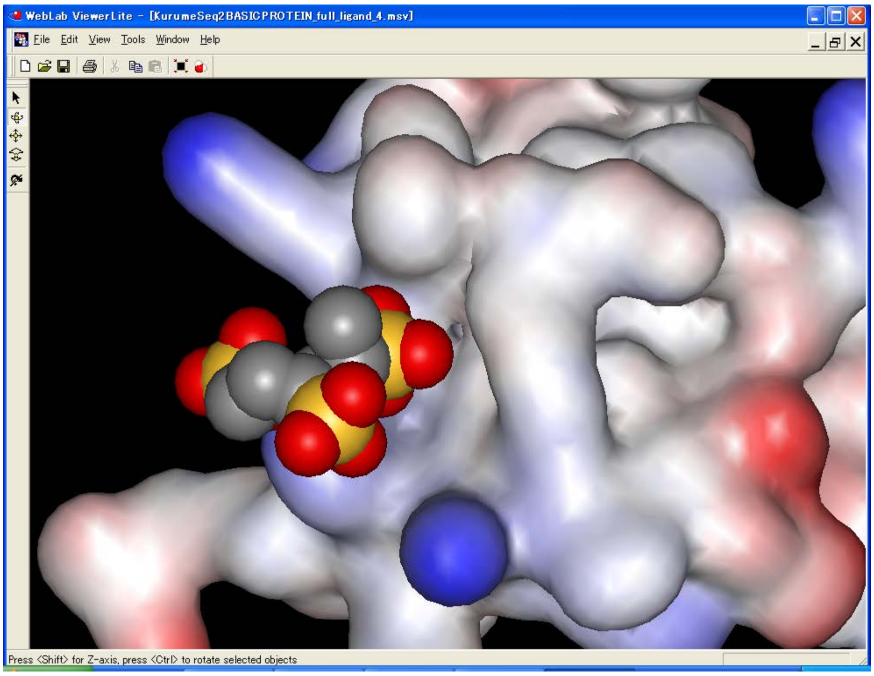
WebLab ViewerLite - [KurumeSeq2BASICPROTEIN_full_ligand_4.msv]
File Edit View Tools Window Help
Press <Shift> for Z-axis, press <Ctrl> to rotate selected objects

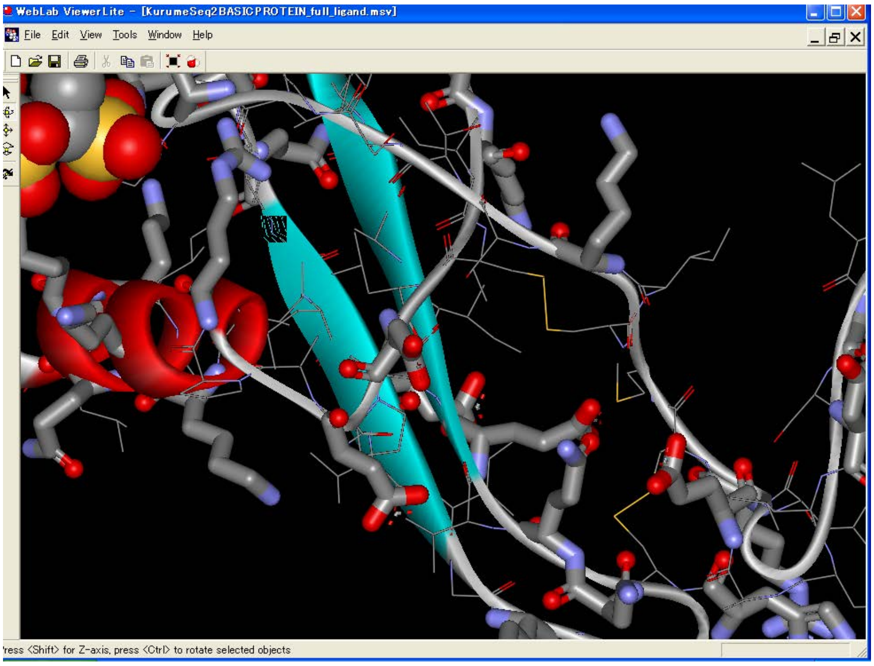
WebLab ViewerLite - [KurumeSeq2BASICPROTEIN_full_ligand.msv]
File Edit View Tools Window Help
ress <Shift> for Z-axis, press <Ctrl> to rotate selected objects

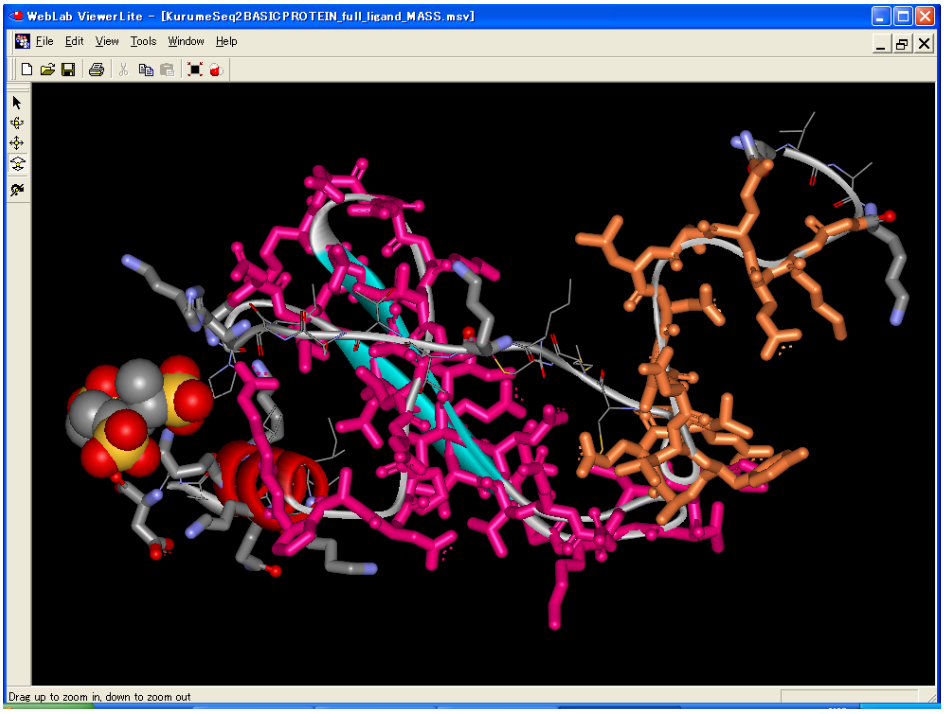

NLAK

GKEESLDSDLYAELR Gly5-Arg19: Orange

CMCIKTTSGIHPK

NIQSLEVIGKGTHCNQVEVIATLKDGRKICLDPDAPR ASN33-ARG69: Red purple

IKKIVQKKLAG

Mass data

Database	NCBInr_20090519 database<br>Proteome Discoverer v1.4<br>Mascot database search v2.2
Taxonomy	Homo sapiens (human)
Type of search	MS/MS Ion Search
Enzyme	Trypsin
Fixed modifications	Carbamidomethyl (C)
Variable modifications	Carbamidomethyl (C), Oxidation (M)
Mass values	Monoisotopic
Peptide mass tolerance	± 1 Da
Fragment mass tolerance	± 0.9 Da
Max missed cleavages	2
Instrument type	ESI-TRAP
Number of queries	16,195
Significance threshold p<	0.014651
Max. number of families	AUTO
Ions score or expect cut-off	0
Preferred taxonomy	All entries
Show Percolator scores?	no
Accession# (NCBInr)	gi|4505981
Protein name	pro-platelet basic protein precursor
pI theoretical value	9.04
Molecular weight (Da)	13,894
Mascot Search Total Score	348

S1 Fig. Analysis of serum target proteins in OPLL. (**A**) SELDI analysis by CM10. (**B**) Proteins were separated by cation exchange HPLC using a SP-STAT column, and the D5 fraction was further separated using SuperODS reverse-phase HPLC (**C**). (**D**) Fraction No. 19, from the final reverse phase HPLC step, was subjected to SDS-PAGE and CBB staining, and the protein was excised from the gel, purified, and identified using the Mascot search engine (Matrix Science) (**E**). (**F**) Protein identity was

confirmed by prediction of *in silico* three-dimensional structure based on the amino acid sequence using the Full Automatic Modeling System (FAMS; http://www.pharm.kitasato-u.ac.jp/fams/). Two S-S bridges were identified.
