# Supplementary material for: Ubiquitin-dependent proteolysis of CXCL7 leads to posterior longitudinal ligament ossification
Source: PLoS One. 2018 May 21;13(5):e0196204. doi: 10.1371/journal.pone.0196204 (PMC5962073; doi:10.1371/journal.pone.0196204)
Supplement: S2 Fig — (PDF) [file pone.0196204.s003.pdf]

Supporting Information

Ubiquitin-dependent proteolysis of CXCL7 leads to posterior longitudinal ligament ossification

Michiyo Tsuru, Atsushi Ono, Hideaki Umeyama, Masahiro Takeuchi and Kensei Nagata

SUPPLEMENTAL FIGURES

S2 Fig. Generation of *CXCL7*-null mice and determination of bone histomorphometric values in OPLL.

S2 Fig.

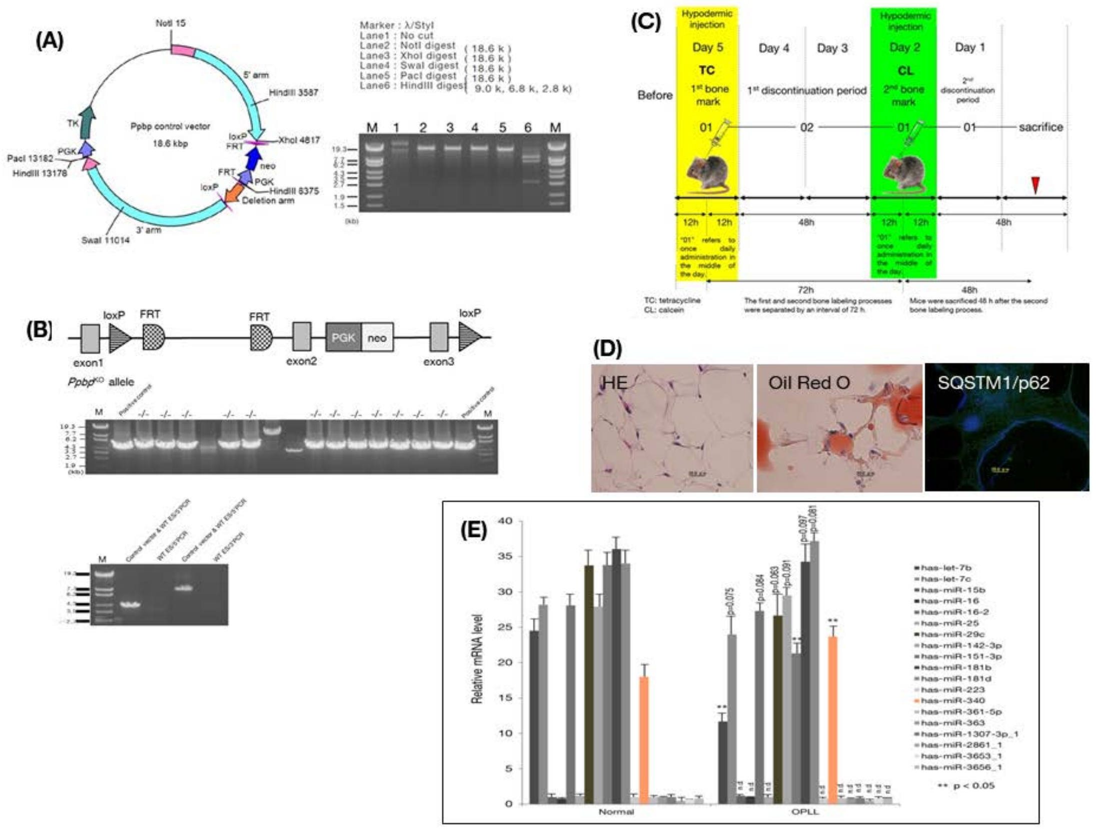

S2 Fig. Generation of *CXCL7*-null mice and determination of bone histomorphometric values in OPLL. (**A**) Construction of the *CXCL7*-targeting vector. (**B**) PCR analysis of CXCL7 conditional targeting vectors. Deletion of exons 2 and 3 causes a frame shift, confirmed by PCR analysis using the 3' primer (AAATTAATTAAGTGCTTTGAGAACAACATTGATAGC) and the 5' primer (AAAGCGGCCGCAGAGAAATAATGACAGAGGGAGACC). (**C**) Bone histomorphometric analysis of wild-type and *CXCL7*-null mice. The spinal bones of knockout mice were fixed in ethanol, stained by the Villanueva method, and embedded in methyl methacrylate without decalcification. The administration schedule involved a single subcutaneous injection of tetracycline (TC) administered to wild-type and *CXCL7*-null mice, followed by no administration on the next two days. Thereafter, the

mice were given a single subcutaneous injection of calcein (CL), followed by no administration on the next day. The results of the bone histomorphometric analysis are expressed according to the methods of the ASBMR Histomorphometry Nomenclature Committee, excluding the osteocyte parameters. (**D**) Frozen sections derived from tissues not obviously corresponding to the seminal vesicle were shown to contain adipose tissue by staining with Oil Red O. The same tissues were also subjected to immunohistochemical staining for adiponectin, SQSTM1/p62 (Alexa Fluor 488: green) and DAPI. (**E**) Determination of micro (mi)RNA expression levels using microarray analysis. miRNA and gene expression profiles in OPLL patients are shown. miRNA was extracted using the miRCURY RNA Isolation kit (Exiqon, Vedbaek, Denmark) per the manufacturer's protocol {GEO database (http://www.ncbi.nlm.nih.gov/geo/) GSE57592}. Upregulation of miR-340 was detected by real-time PCR (normal, n = 7; OPLL, n = 13). miRNA array analysis was performed to elucidate the cause of CXCL7 deficiency *in vivo*. In addition, E3 ubiquitin ligase was determined from phosphoproteomics analysis of wild-type and *CXCL7*-null OPLL model mice. miR-340 was upregulated in OPLL patients relative to healthy subjects, as determined by microarray analysis {GEO database (http://www.ncbi.nlm.nih.gov/geo/) GSE57592}. RNA samples (100 ng/μL) were extracted from kidney tissues of both wild-type and homozygous *CXCL7* knockout mice and then subjected to gene array analyses (Agilent Technologies, Santa Clara, CA) (GEO database GES57590) (Supplementary Dataset).
