# Supplementary material for: Ubiquitin-dependent proteolysis of CXCL7 leads to posterior longitudinal ligament ossification
Source: PLoS One. 2018 May 21;13(5):e0196204. doi: 10.1371/journal.pone.0196204 (PMC5962073; doi:10.1371/journal.pone.0196204)
Supplement: S3 Fig — (PDF) [file pone.0196204.s004.pdf]

Supporting Information

Ubiquitin-dependent proteolysis of CXCL7 leads to posterior longitudinal ligament ossification

Michiyo Tsuru, Atsushi Ono, Hideaki Umeyama, Masahiro Takeuchi and Kensei Nagata

SUPPLEMENTAL FIGURES

S3 Fig. Defective system of target CXCL7 protein.

S3 Fig.

(A)

B	B Ions	B+2H	B-NH3	B-H2O	AA	Y Ions	Y+2H	Y-NH3	Y-H2O	Y
1	100.1				V	1,237.6	619.3	1,220.5	1,219.6	10
2	247.1				M+16	1,138.5	569.8	1,121.5	1,120.5	9
3	318.1				A	991.5	496.2	974.4	973.5	8
4	389.2				A	920.4	460.7	903.4	902.4	7
5	570.2			552.2	T+80	849.4	425.2	832.4	831.4	6
6	683.3	342.1		665.3	I	668.4	334.7	651.3	650.4	5
7	850.3	425.6		832.3	S+80	555.3	278.1	538.3	537.3	4
8	978.4	489.7	961.4	960.4	K	388.3	194.6	371.3		3
9	1,091.5	546.2	1,074.4	1,073.5	L	260.2		243.2		2
10	1,237.6	619.3	1,220.5	1,219.6	K	147.1		130.1		1

(B)

B	B Ions	B+2H	B-NH3	B-H2O	AA	Y Ions	Y+2H	Y-NH3	Y-H2O	Y
1	72.0				A	1,639.8	820.4	1,622.8	1,621.8	16
2	228.1	114.6	211.1		R	1,568.8	784.9	1,551.7	1,550.7	15
3	357.2	179.1	340.2	339.2	E	1,412.7	706.8	1,395.6	1,394.6	14
4	471.2	236.1	454.2	453.2	N	1,283.6	642.3	1,266.6	1,265.6	13
5	599.3	300.2	582.3	581.3	K	1,169.6	585.3	1,152.5	1,151.6	12
6	686.4	343.7	669.3	668.3	S	1,041.5	521.2	1,024.4	1,023.5	11
7	757.4	379.2	740.4	739.4	A	954.4	477.7	937.4	936.4	10
8	828.4	414.7	811.4	810.4	A	883.4	442.2	866.4	865.4	9
9	915.5	458.2	898.4	897.5	S	812.4	406.7	795.3	794.4	8
10	1,014.5	507.8	997.5	996.5	V	725.3	363.2	708.3	707.3	7
11	1,181.5	591.3	1,164.5	1,163.5	S+80	626.3	313.6	609.2	608.3	6
12	1,280.6	640.8	1,263.6	1,262.6	V	459.3		442.2		5
13	1,351.6	676.3	1,334.6	1,333.6	A	360.2		343.2		4
14	1,408.7	704.8	1,391.6	1,390.6	G	289.2		272.1		3
15	1,465.7	733.3	1,448.7	1,447.7	G	232.1		215.1		2
16	1,639.8	820.4	1,622.8	1,621.8	R	175.1		158.1		1

(C)

B	B Ions	B+2H	B-NH3	B-H2O	AA	Y Ions	Y+2H	Y-NH3	Y-H2O	Y
1	88.0			70.0	S	1,778.8	889.9	1,761.8	1,760.8	14
2	189.1			171.1	T	1,691.7	846.4	1,674.7	1,673.7	13
3	302.2			284.2	L	1,590.7	795.9	1,573.7	1,572.7	12
4	416.2		399.2	398.2	N	1,477.6	739.3	1,460.6	1,459.6	11
5	513.3		496.2	495.3	P	1,363.6	682.3	1,346.5	1,345.6	10
6	641.3	321.2	624.3	623.3	Q	1,266.5	633.8	1,249.5	1,248.5	9
7	827.4	414.2	810.4	809.4	W	1,138.5	569.7	1,121.4	1,120.4	8
8	941.4	471.2	924.4	923.4	N	952.4	476.7	935.4	934.4	7
9	1,070.5	535.7	1,053.5	1,052.5	E	838.3	419.7	821.3	820.3	6
10	1,237.5	619.2	1,220.5	1,219.5	S+80	709.3		692.3	691.3	5
11	1,384.6	692.8	1,367.5	1,366.5	F	542.3		525.3	524.3	4
12	1,485.6	743.3	1,468.6	1,467.6	T	395.2		378.2	377.2	3
13	1,632.7	816.8	1,615.6	1,614.7	F	294.2		277.2		2
14	1,778.8	889.9	1,761.8	1,760.8	K	147.1		130.1		1

(D)

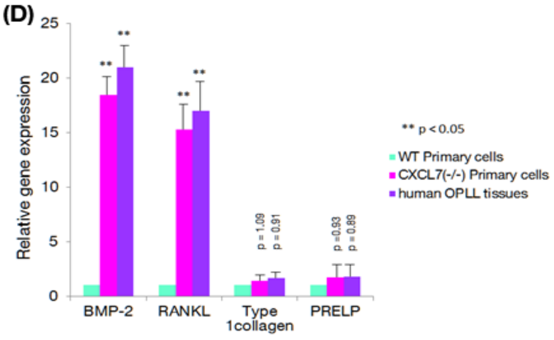

(F)

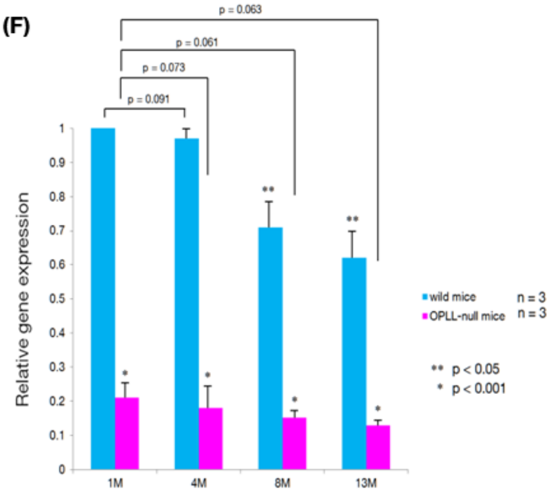

(E)

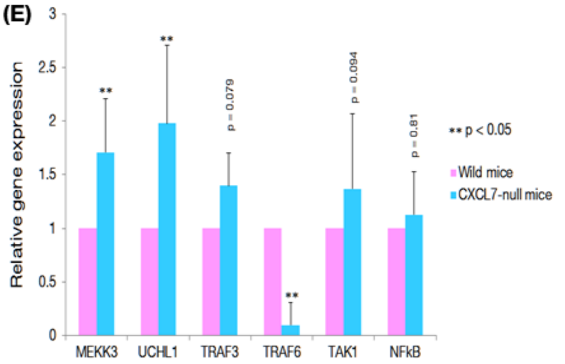

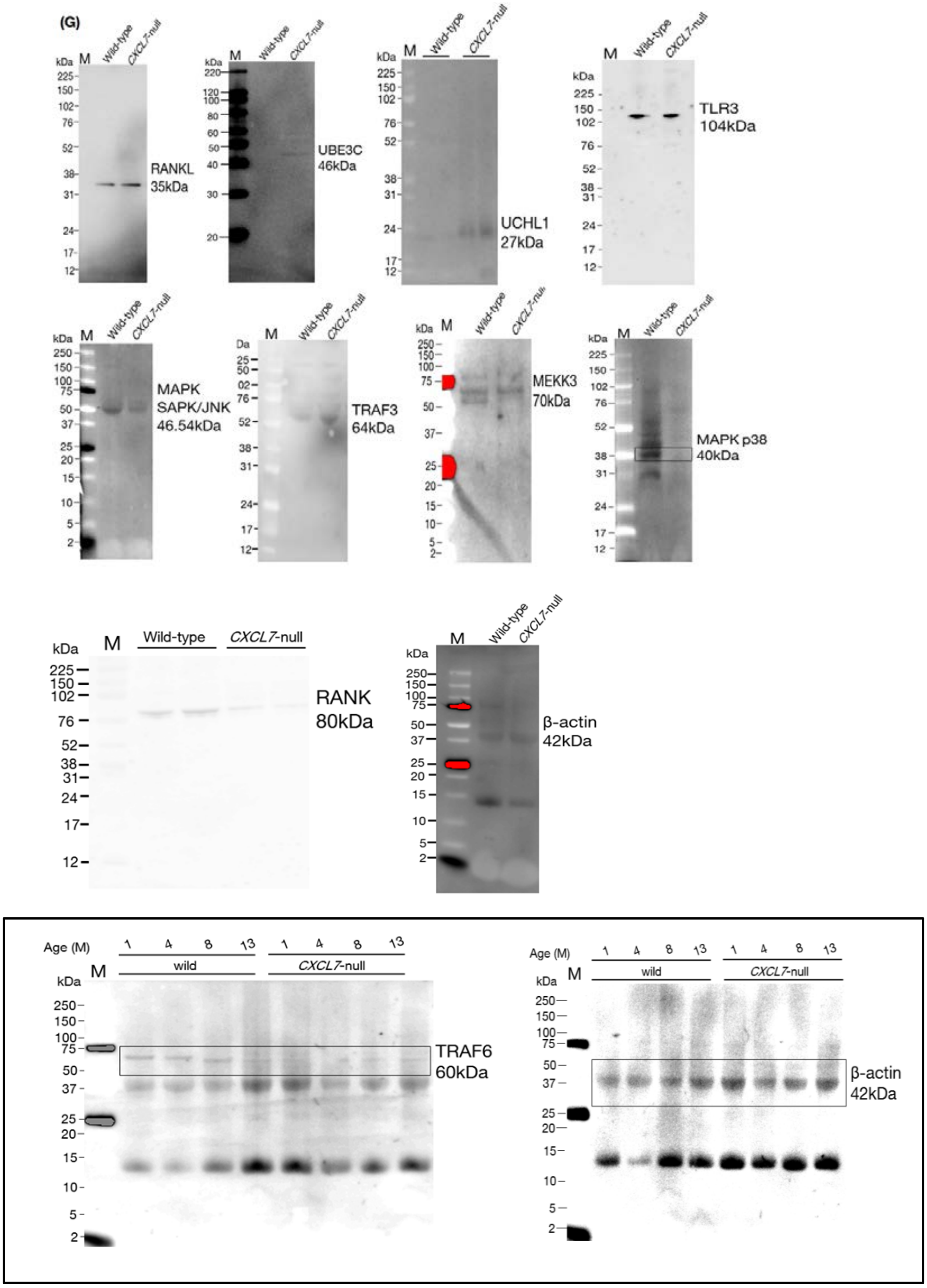
(G)
M
Wild-type
CXCL7-null
kDa
RANKL
35kDa
UBE3C
46kDa
UCHL1
27kDa
TLR3
104kDa
MAPK
SAPK/JNK
46.54kDa
TRAF3
64kDa
MEKK3
70kDa
MAPK p38
40kDa
RANK
80kDa
β-actin
42kDa
Age (M)
1 4 8 13 1 4 8 13
wild
CXCL7-null
TRAF6
60kDa
β-actin
42kDa

(H)

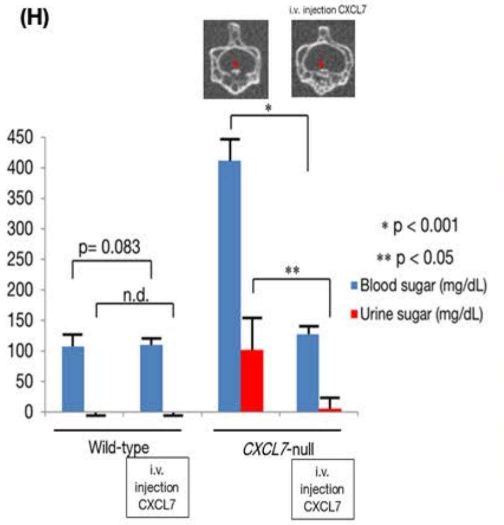
i.v. injection CXCL7
450
400
350
300
250
200
150
100
50
0
p= 0.083
n.d.
*
**
* p < 0.001
** p < 0.05
Blood sugar (mg/dL)
Urine sugar (mg/dL)
Wild-type
CXCL7-null
i.v. injection CXCL7
i.v. injection CXCL7

(J)

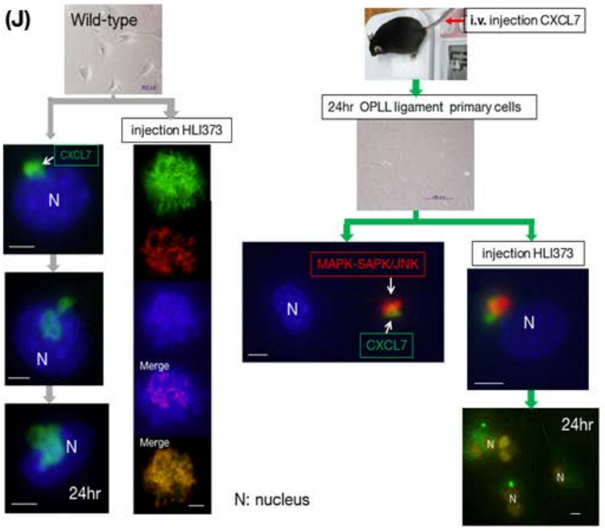
Wild-type
injection HLI373
CXCL7
N
Merge
Merge
24hr
N: nucleus
i.v. injection CXCL7
24hr OPLL ligament primary cells
MAPK-SAPK/JNK
CXCL7
injection HLI373
24hr

(I)

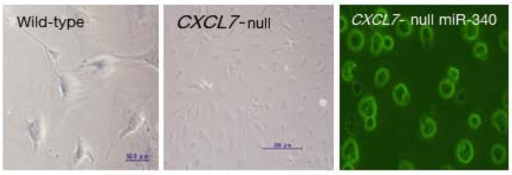
Wild-type
CXCL7-null
CXCL7- null miR-340

(K)

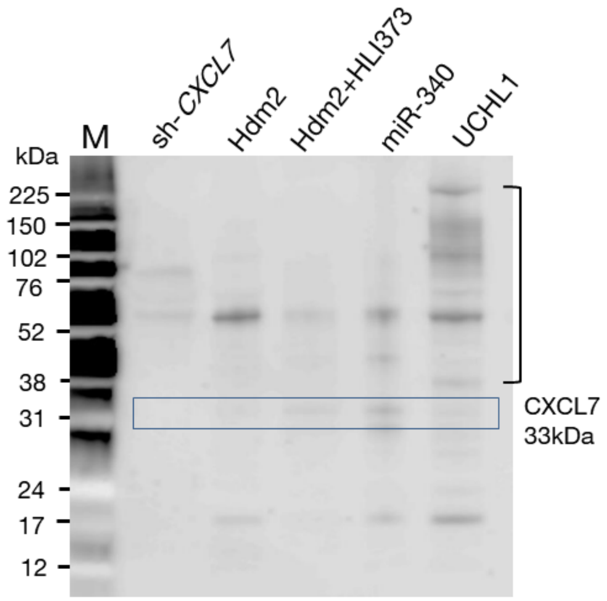
M
sh-CXCL7
Hdm2
Hdm2+HLI373
miR-340
UCHL1
kDa
225
150
102
76
52
38
31
24
17
12
CXCL7
33kDa

(L) Western blot of ubiquitin (linkage-specific K48)

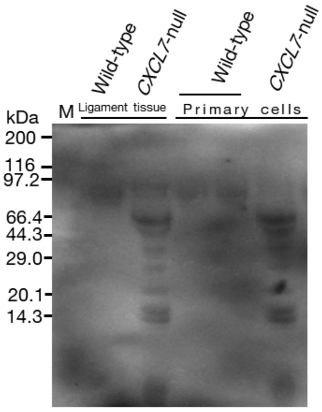

(M) Immunoprecipitation analysis of K48

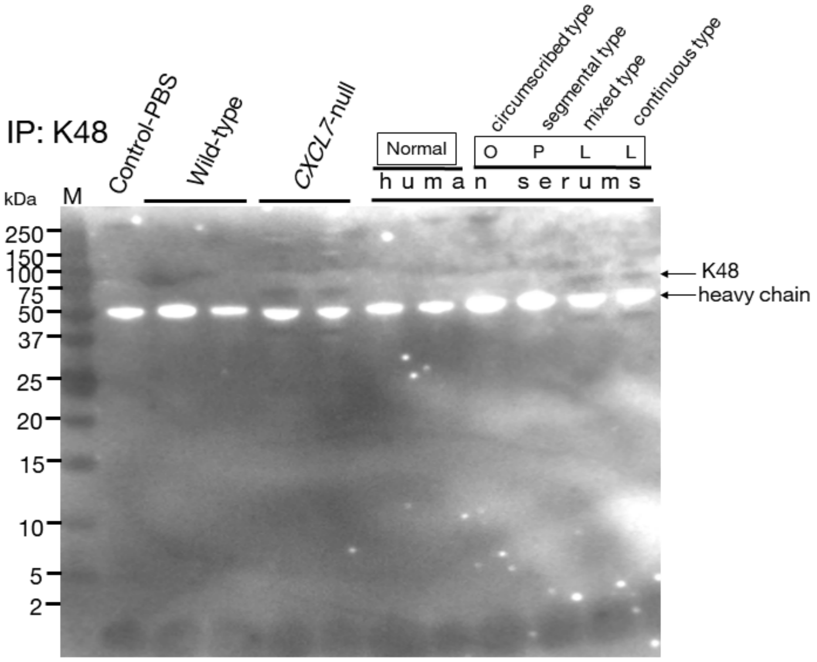

(N) Western blot of K48 in patient's white blood cells

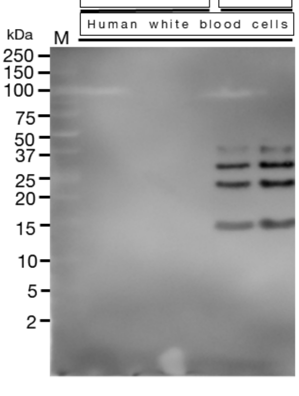

Mass data

Database	NCBInr 20150523<br>Mascot database search v2.5.1<br>Scaffold_4.4.3
Taxonomy	Mus. (176,196 sequences)
Type of search	MS/MS Ion Search
Enzyme	Trypsin
Fixed modifications	Carbamidomethyl (C)
Variable modifications	Oxidation (M), Phospho (S), Phospho (T), Phospho (Y)
Mass values	Monoisotopic
Peptide mass tolerance	10 ppm
Fragment mass tolerance	0.6 Da
Max missed cleavages	2
Instrument type	ESI-TRAP
Number of queries	16,195
Significance threshold p<	0.014651
Max. number of families	AUTO
Ions score or expect cut-off	0
Preferred taxonomy	All entries
Show Percolator scores?	No
Accession# (NCBInr)	gi|282847404
Protein name	mitogen-activated protein kinase kinase kinase 15
Molecular weight (Da)	149,331.2
pI theoretical value	6.8
Accession# (NCBInr)	gi|164663791
Protein name	protein kinase C alpha type
Molecular weight (Da)	76,825.3
pI theoretical value	5.4
Accession# (NCBInr)	gi|568932043
Protein name	E3 ubiquitin-protein ligase MIB2 isoform X1
Molecular weight (Da)	106,823.1
pI theoretical value	8.9

S3 Fig. Defective system of target CXCL7 protein. Analysis of protein phosphorylation. Phosphorylation proteomics analysis was conducted via a two-dimensional electrophoresis analysis of wild-type (SYPRO Ruby; excitation at 488 nm and band pass (BP) filter of 640 nm) and *CXCL7*-null mice (Pro-Q Diamond; excitation at 532 nm and long pass (LP) filter of 555 nm). (**A**) Liquid chromatography-tandem mass spectrometry analysis. A scaffold analysis was performed to examine the expression of phosphorylated mitogen-associated protein kinase kinase kinase 15 (Proteome Software, Portland, OR, USA). Proteomics analysis revealed phosphorylation of MAPK kinase kinase (MAP3K)15 at threonine 1076 and serine 1078 in primary cells derived from *CXCL7*-null mice. (**B**) Proteomic analysis of E3 ubiquitin ligase serine phosphorylation. The scaffold fragmentation table is shown. Phosphorylation of E3 ubiquitin ligase was observed at serine 419 in primary cells derived from *CXCL7*-null mice. (**C**) Proteomic analysis of protein kinase C alpha serine phosphorylation. The scaffold fragmentation table is shown. Phosphorylation of protein kinase C alpha was observed at serine 235 in primary cells derived from *CXCL7*-null mice. Ubiquitin function in *CXCL-7*-null mice. (**D**) Analysis of primary cultures of mouse spinal ligament cells and human OPLL tissues by real-time PCR. Wild-type and *CXCL7*-null mouse spinal ligament tissues were excised and subjected to primary culture followed by real-time PCR to examine the expression of type I collagen and PRELP [1], which are highly expressed in ligamentous tissues, to elucidate the mechanisms underlying ubiquitin-induced suppression of CXCL7, thus confirming the equivalence to human spinal ligament tissue. The expression of BMP-2 and RANKL was also analyzed in human OPLL tissues and *CXCL7*-null mouse spinal ligament tissues compared to wild-type mouse spinal ligament tissues. (**E**) Real-time PCR

analysis of primary cultured cells from wild-type (n = 5) and *CXCL7*-null mice (n = 5) was performed to examine the expression of MEKK3, UCHL1, TRAF3, TRAF6, TAK1, and NF-κB. (**F**) Furthermore, TRAF6 expression in wild-type (n = 3) and *CXCL7*-null mice (n = 3) from the age of 1 month through 13 months were examined using real-time PCR. (**G**) Western blot analysis of RANKL, UBE3C, TLR3, MAPK SAPK/JNK, TRAF3, TRAF6, MEKK3, MAPK p38, UCHL1, RANK and β-actin levels in wild-type and *CXCL7*-null spinal ligament tissues. Western blot was used to examine TRAF6 expression by age (1, 4, 8 and 13 M) in wild and *CXCL7*-null mice. (**H**) Blood and urine glucose levels in wild-type (n = 5) and *CXCL7*-null (n = 5) mice were measured 30 min after intravenous administration of human recombinant CXCL7. (**I**) Primary cell cultures were obtained by treating spinal tissue obtained from *CXCL7*-null and wild-type mice. (**J**) Left hand-side: Effect of treatment with the ubiquitin ligase E3 5 μM inhibitor (HLI 373) for 30 min on CXCL7 expression (Alexa Fluor 488) and MAPK-SAPK/JNK activation (Alexa Fluor 555) in primary cells derived from spinal ligaments of wild-type mice. Right hand-side: *CXCL7*-null mice were treated with human recombinant CXCL7 (1 mg/kg/week for 3 weeks), and primary cells derived from spinal ligaments were examined for the effect of HLI 373. Scale bar, 10 μm. (**K**) Primary cells obtained from the spinal ligament of wild-type mice were transfected with sh-RNA-CXCL7, treated with recombinant E3 ubiquitin ligase Hdm2 (1 μM for 30 min) in the presence or absence of HLI 373 (5 μM for 30 min), transfected with miR-340, or treated with 50 nM recombinant UCHL1 (R&D) for 15 min, and the expression of CXCL7 was determined using western blotting. In control cells (sh-*CXCL7*), the expression of CXCL7 was suppressed, whereas in cells expressing miR-340, CXCL7 expression was still observed. Based on these data, we consider that

the reduction in CXCL7 levels in OPLL patients was not induced by miR-340. (**L**) Western blot analysis of ubiquitin (linkage-specific K48) in wild-type and *CXCL7*-null spinal ligament tissues. (**M**) K48 was immunoprecipitated using spinal ligament primary cells of wild-type *CXCL7*-null mice and sera of healthy subjects and OPLL patients. IP; immunoorecipitation. (**N**) Western blot of K48 of white blood cells of healthy subjects and OPLL patients was performed. In the patients with OPLL, K48 bands were confirmed.

Full-length western blot images for Fig. 3E, 3H and 3J.

Fig. 3E

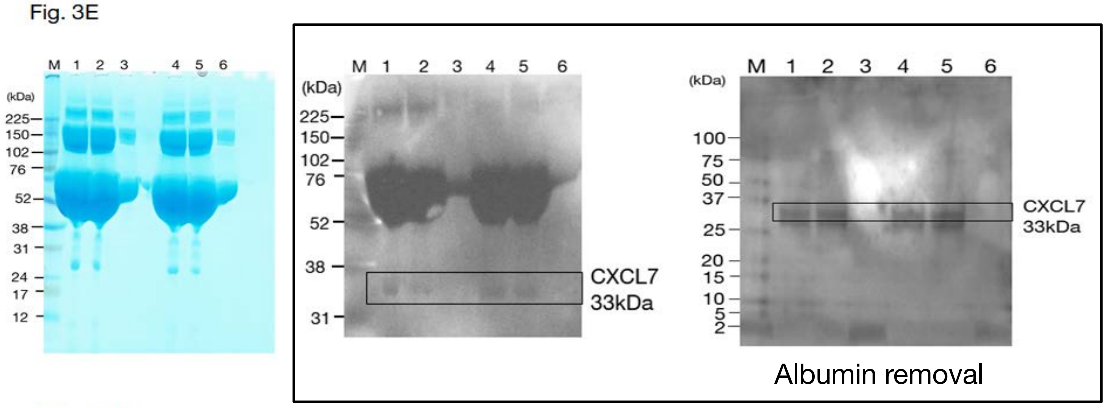

Fig. 3H

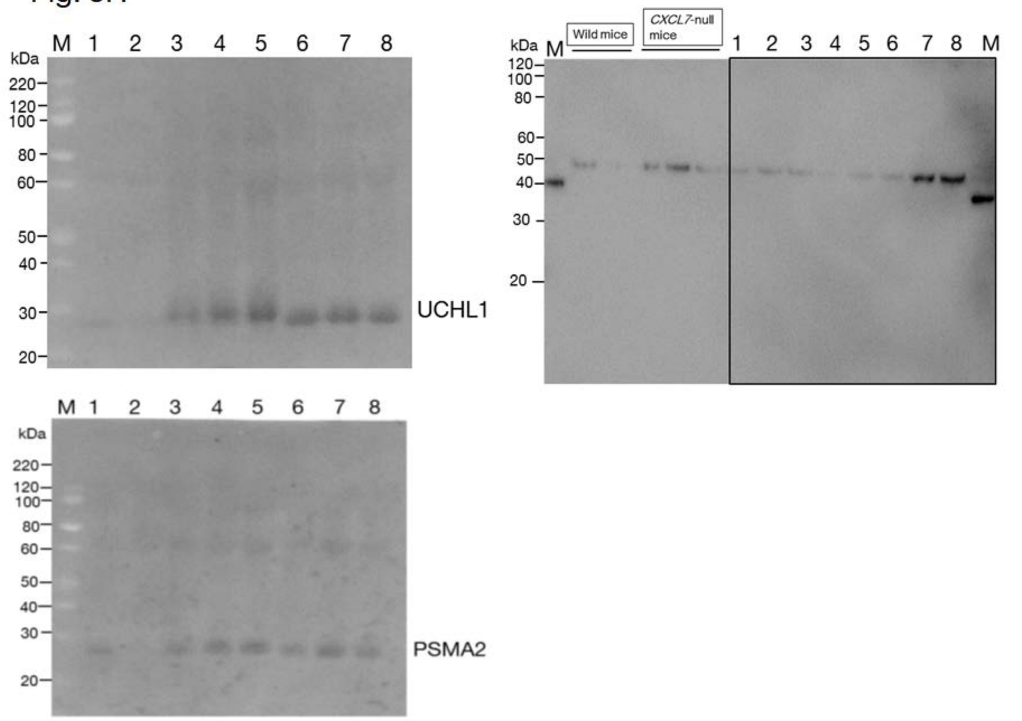

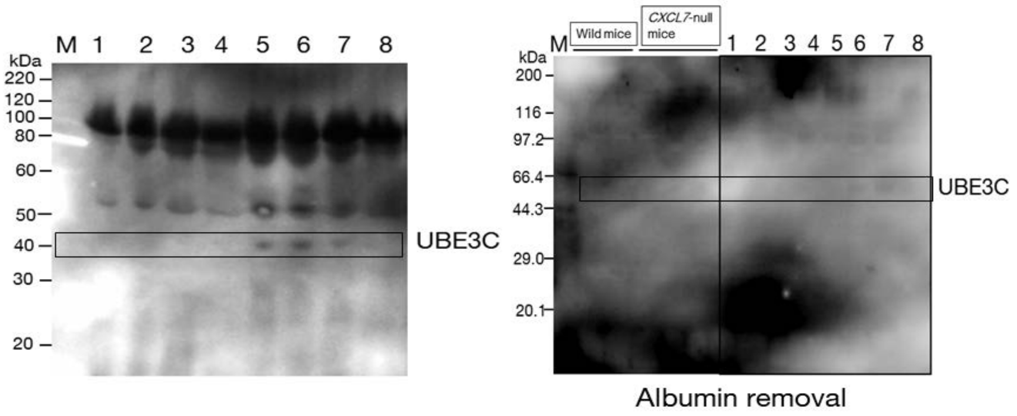

Fig. 3J

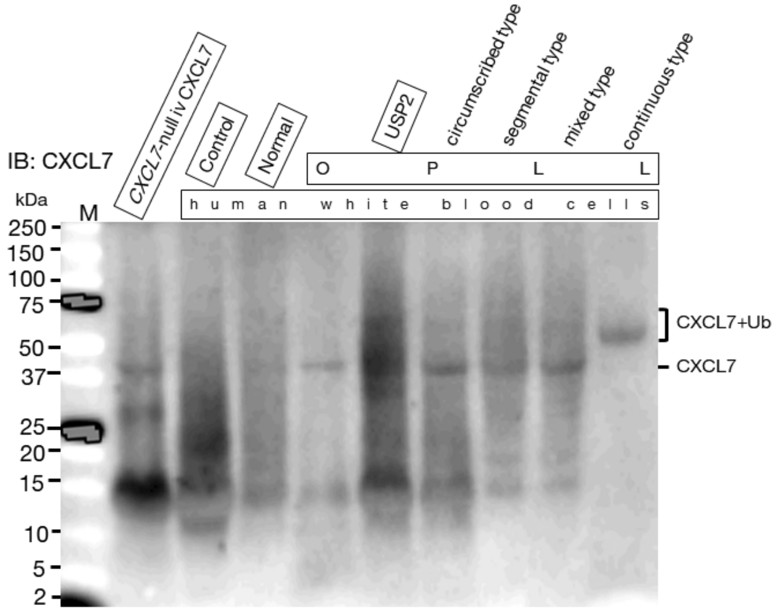

intravenous; iv

References

1. Tsuru M, Soejima T, Shiba N, Kimura K, Sato K, Toyama Y, et al. Proline/arginine-rich end leucine-rich repeat protein converts stem cells to ligament tissue and Zn(II) influences its nuclear expression. Stem Cells Dev. 2013;22: 2057–2070.
