# Supplementary material for: Ubiquitin-dependent proteolysis of CXCL7 leads to posterior longitudinal ligament ossification
Source: PLoS One. 2018 May 21;13(5):e0196204. doi: 10.1371/journal.pone.0196204 (PMC5962073; doi:10.1371/journal.pone.0196204)

# Supporting Information

**Ubiquitin-dependent proteolysis of CXCL7 leads to posterior longitudinal ligament ossification**

Michiyo Tsuru, Atsushi Ono, Hideaki Umeyama, Masahiro Takeuchi and Kensei Nagata

**SUPPLEMENTAL FIGURES**

**S4 Fig. Ossification by CXCL7 knockdown in equine mesenchymal stem cells. Verification of the background for CXCL7 protein deficiency.**

**S4 Fig.**

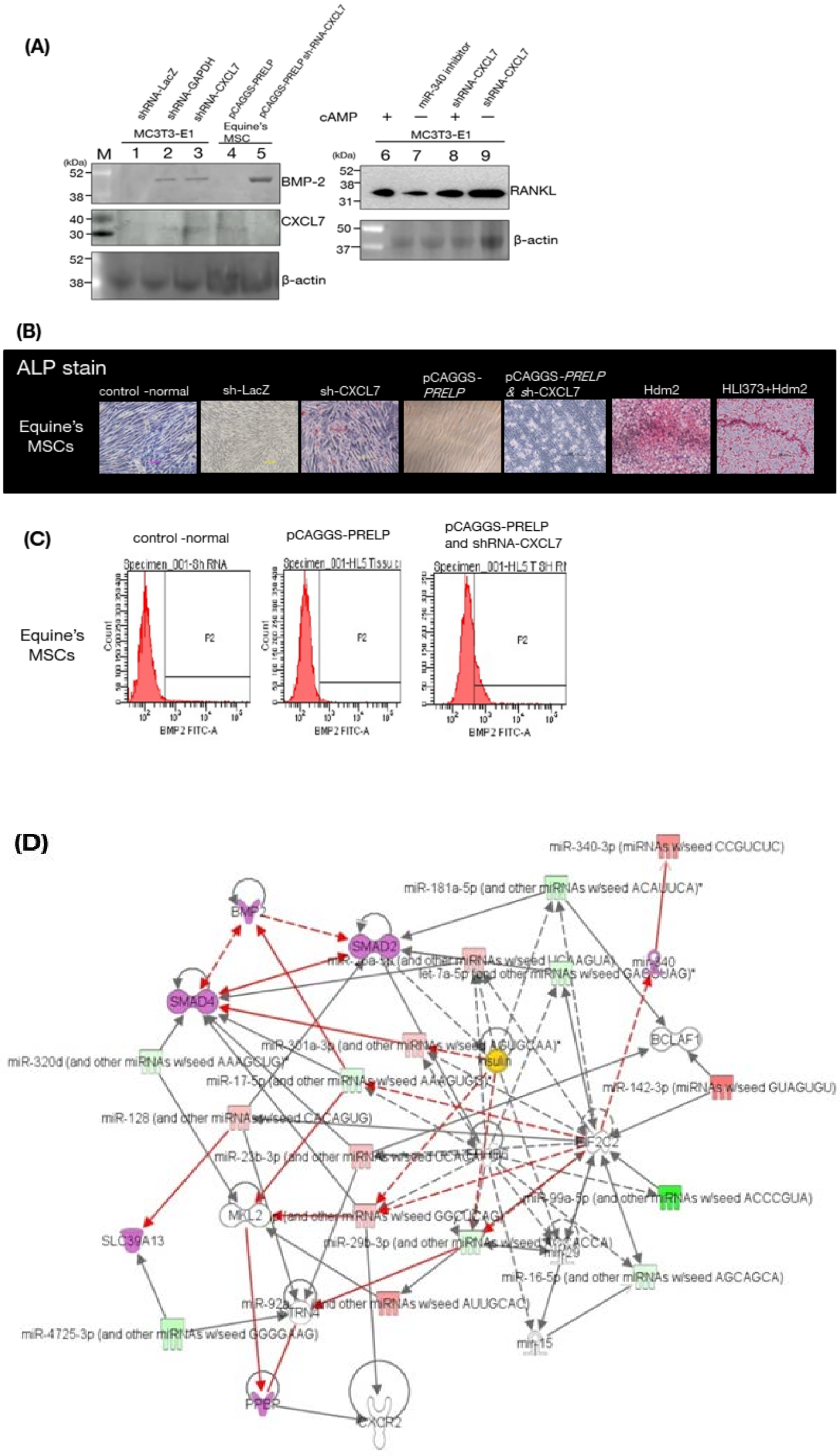
(A)
shRNA-LacZ
shRNA-GAPDH
shRNA-CXCL7
pCAGGS-PRELP
pCAGGS-PRELP sh-RNA-CXCL7
MC3T3-E1
Equine's MSC
BMP-2
CXCL7
β-actin
cAMP
miR-340 inhibitor
shRNA-CXCL7
RANKL
(B)
ALP stain
control -normal
sh-LacZ
sh-CXCL7
pCAGGS-PRELP
pCAGGS-PRELP & sh-CXCL7
Hdm2
HLI373+Hdm2
Equine's MSCs
(C)
control -normal
pCAGGS-PRELP
pCAGGS-PRELP and shRNA-CXCL7
Equine's MSCs
BMP2 FITC-A
(D)

**S4 Fig. Ossification by CXCL7 knockdown in equine mesenchymal stem cells. Verification of the background for CXCL7 protein deficiency.** (**A**) BMP-2, CXCL7, RANKL, and β-actin expression were assessed by western blotting. Cells expressing shRNA-LacZ were used as the negative control (lane 1) and shRNA-*GAPDH* was used as the positive control (lane 2). CXCL7 was suppressed by shRNA-*CXCL7* (lane 3). Overexpression of proline/arginine-rich end leucine-rich repeat protein (PRELP) by *pCAGGS-PRELP* in equine mesenchymal stem cells (lane 4) drives them toward a ligament-like tissue lineage. Similar to the observations in lane 4, CXCL7 was suppressed by shRNA in lane 5. Lane 6 shows the effect of treatment with cAMP (150 μM) (Merck, Darmstadt, Germany) in MC3T3-E1 cells, lane 7 shows the effect of treatment with an miR-340 inhibitor, lane 8 shows treatment with cAMP & shRNA-*CXCL7*, and lane 9 shows treatment with shRNA-*CXCL7* alone. (**B**) Alkaline phosphatase staining was performed on marrow-derived equine mesenchymal stem cells (MSCs). Alkaline phosphatase was no longer present when cells were driven to a ligament-like tissue lineage by PRELP.

In contrast, overexpression of PRELP drives marrow-derived equine ligament-like tissue after expression of shRNA-*CXCL7*. The data for MSCs, shRNA-LacZ–treated cells, and shRNA-*CXCL7*–treated cells are shown. (**C**) MSCs were isolated from equine cerebrospinal fluid and transfected with the pCAGGS-*PRELP* vector. One day later, single cells were obtained from a ligament like tissue sample and transfected with shRNA-*CXCL7*. The expression of BMP-2 was assessed using an anti BMP-2 antibody (GeneTex, Irvine, CA) using FACS analysis (FACS Canto II, Becton Dickinson, Franklin Lakes, NJ) of stem cells derived from ligament-like tissue and transfected with either shRNA-LacZ or the pCAGGS-*PRELP* plasmid. MSCs were transfected with the

*pCAGGS-PRELP* plasmid and then 24 h later transfected with shRNA-*CXCL7*. The tissue was converted to single cells for FACS analysis. We observed high levels of BMP-2 expression, thus confirming ossification of the ligament-like cells. (**D**) Pathway analysis based on data from genomic and miRNA array analysis (Agilent Technologies, Santa Clara, CA, USA) of OPLL patient samples.

**Full-length western blot images for Fig. 4B, and S4 Fig. A.**

Fig. 4B

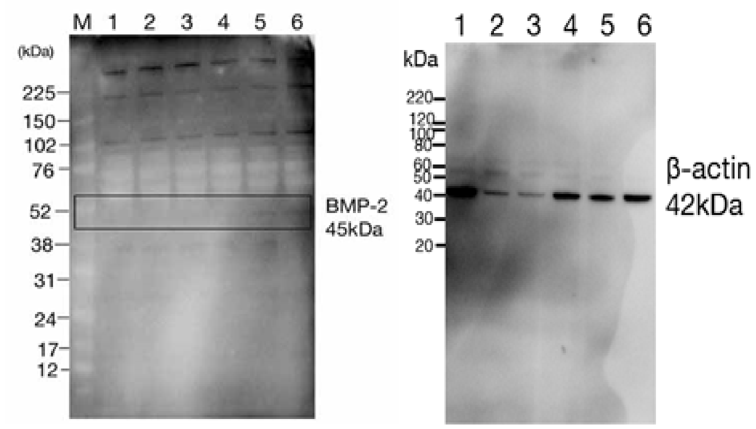


S4 Fig. A.

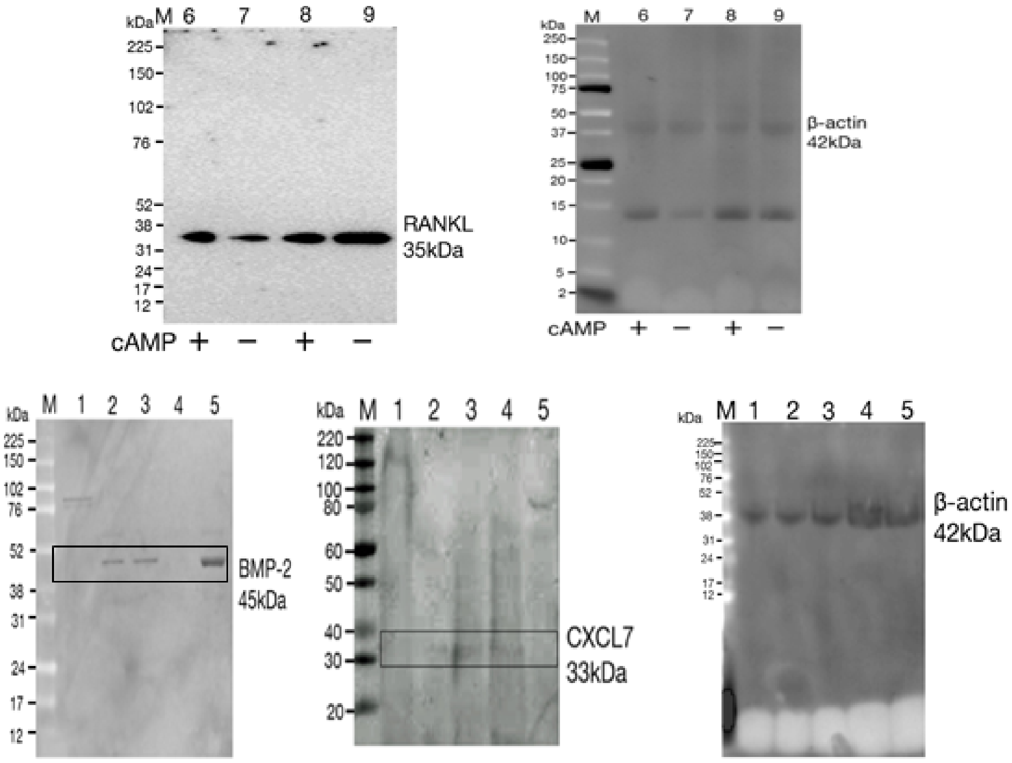

Supplement: S4 Fig — Verification of the background for CXCL7 protein deficiency. (PDF) [file pone.0196204.s005.pdf]
