# Supplementary material for: Ubiquitin-dependent proteolysis of CXCL7 leads to posterior longitudinal ligament ossification
Source: PLoS One. 2018 May 21;13(5):e0196204. doi: 10.1371/journal.pone.0196204 (PMC5962073; doi:10.1371/journal.pone.0196204)
Supplement: S1 Table — (PDF) [file pone.0196204.s006.pdf]

Supporting Information

Ubiquitin-dependent proteolysis of CXCL7 leads to posterior longitudinal ligament ossification

Michiyo Tsuru, Atsushi Ono, Hideaki Umeyama, Masahiro Takeuchi and Kensei Nagata

SUPPLEMENTAL TABLE

S1 Table. Abbreviations of the histomorphometric parameters according to the American Society for Bone and Mineral Research: ASBMR nomenclature committee.

S1 Table. Abbreviations of the histomorphometric parameters according to the American Society for Bone and Mineral Research: ASBMR nomenclature committee.

Bone histomorphometry			
Tr. Cr.	Total cortical bone area	N. Mu. Oc/BS	Multinuclear osteoclast Number
Ps. S.	Periosteal surface	N. Mu. Oc/ES	Multinuclear osteoclast Number
Ct. Wi.	Cortical width	N. Mo. Oc/BS	Mononuclear osteoclast Number
TV	Tissue volume	N. Mo. Oc/ES	Mononuclear osteoclast Number
BS	Bone surface	N. Ob/BS	Osteoblast number
OS	Osteoid surface	N. Ob/OS	Osteoblast number
ES	Eroded surface	N. Oc/BS	Osteoclast number
QS	Quiescent surface	N. Oc/ES	Osteoclast number
Ob. S.	Osteoblast surface	N. Mu.Oc/TV	Multinuclear Osteoclast Number
Oc. S.	Osteoclast surface	N. Mo. Oc/TV	Mononuclear osteoclast Number
BV	Bone volume	N. Oc/TV	Osteoclast number
N. Mu. Oc	Multinuclear osteoclast number	N. Ob/TV	Osteoblast number
N. Mo. Oc	Mononuclear osteoclast number	MAR	Mineral apposition rate
N. Ob	Osteoblast number	MS/OS	Mineralizing surface
OV	Osteoid volume	LS/OS	Labeled surface
O. Th	Osteoid thickness	Aj. Ar	Adjusted appositional rate
dL. S	Double labeled surface	Omt	Osteoid maturation time
sL. S	Single labeled surface	Mlt	Mineralization lag time
Vd (d+s) LS	Void labeled surface	BFR/BS	Bone formation rate
L. Th	Labeled thickness	BFR/BV	Bone formation rate
BV/TV	Bone volume	BFR/TV	Bone formation rate
OV/TV	Osteoid volume	BS/TV	Bone surface
OV/BV	Osteoid volume	BS/BV	Bone surface
Tb. Th	Trabecular thickness	dL. S/BS	Double labeled surface
OS/BS	Osteoid surface	sL. S/BS	Single labeled surface
Ob. S/OS	Osteoblast surface	Vd (d+s) LS/BS	Void labeled surface
Ob. S/BS	Osteoblast surface	LS/BS	Labeled surface
OV/OS	Osteoid volume	MS/BS	Mineralizing surfaces
ES/BS	Eroded surface	BRs. R	Bone resorption rate
Oc. S/ES	Osteoclast surface	Tb. N	Trabecular number
Oc. S/BS	Osteoclast surface	Tb. Sp	Trabecular separation
