# Supplementary material for: Ubiquitin-dependent proteolysis of CXCL7 leads to posterior longitudinal ligament ossification
Source: PLoS One. 2018 May 21;13(5):e0196204. doi: 10.1371/journal.pone.0196204 (PMC5962073; doi:10.1371/journal.pone.0196204)
Supplement: S2 Table — (PDF) [file pone.0196204.s007.pdf]

Supporting Information

Ubiquitin-dependent proteolysis of CXCL7 leads to posterior longitudinal ligament ossification

Michiyo Tsuru, Atsushi Ono, Hideaki Umeyama, Masahiro Takeuchi and Kensei Nagata

SUPPLEMENTAL TABLE

S2 Table. Abbreviations (Fig. 2S).

S2 Table. Abbreviations (Fig. 2S)

Retina tissue	
RODS and CONES	rod and cone layer
ONL	outer nuclear layer
OPL	outer plexiform layer
INL	inner nuclear layer
IPL	inner plexiform layer
GCL	ganglion cell layer
