# Supplementary material for: Ubiquitin-dependent proteolysis of CXCL7 leads to posterior longitudinal ligament ossification
Source: PLoS One. 2018 May 21;13(5):e0196204. doi: 10.1371/journal.pone.0196204 (PMC5962073; doi:10.1371/journal.pone.0196204)
Supplement: S1 Dataset — (PDF) [file pone.0196204.s011.pdf]

Supporting Information

Ubiquitin-dependent proteolysis of CXCL7 leads to posterior longitudinal ligament ossification

Michiyo Tsuru, Atsushi Ono, Hideaki Umeyama, Masahiro Takeuchi and Kensei Nagata

Supplementary dataset

Microarray data are available in the Gene Expression Omnibus database (http://www.ncbi.nlm.nih.gov/geo/) under the accession number GSE57590.

Supplementary Dataset GSE57590

ProbeID	GeneSymbol	Description	wild54_signal	homo4_signal	wild54_flag	homo4_flag	compare1 Zscore	compare1 ratio	PrimaryAccession	RefSeqAccession	GenbankAccession	UniGeneID	EntrezGeneID	EnsemblID	TIGRID	GO	GenomicCoordinates	Cytoband
A_52_P270429	Fam25c	Mus musculus family with sequ	30.179735	2829.726	P	P	16.86039	93.762454	NM_183278	NM_183278	NM_183278	Mm.27156	69134	ENSMUST00000052126	TC1582983	GO:0003674(molecular function)|GO:	chr14:35166894-35165215	mm|14qB
A_55_P2011101	Gsdmc3	Mus musculus gasdermin C3 (G	17.786105	3031.035	P	P	14.257874	170.41589	NM_183194	NM_183194	NM_183194	Mm.483471	270328	ENSMUST00000089900	TC1584383	GO:0003674(molecular function)|GO:	chr15:63689366-63689307	mm|15qD1
A_55_P2085142	Spp1	Mus musculus secreted phosph	62.99476	2170.4595	P	P	13.142738	34.454604	NM_001204233	NM_001204233	NM_001204233	Mm.288474	20750	ENSMUST00000112747	TC1681616	GO:0001503(ossification)|GO:000512	chr5:104868502-104868561	mm|5qE5
A_52_P461343	Upk3a	Mus musculus uroplakin 3A (Up	66.33886	2189.2875	P	P	12.982735	33.001585	NM_023478	NM_023478	NM_023478	Mm.46214	22270	ENSMUST00000023070	TC1600934	GO:0000902(cell morphogenesis)|GO	chr15:84851819-84852720	mm|15qE2
A_55_P2156425	Upk1a	Mus musculus uroplakin 1A (Up	160.48845	5048.56	P	P	12.804787	31.457466	NM_026815	NM_026815	NM_026815	Mm.25471	109637	ENSMUST00000006476	TC1586520	GO:0005515(protein binding)|GO:000	chr7:31388170-31388111	mm|7qB1
A_51_P324814	Krt18	Mus musculus keratin 18 (Krt1	500.89095	9026.744	P	P	12.796215	18.021376	NM_010664	NM_010664	NM_010664	Mm.22479	16668	ENSMUST00000023803	TC1674411	GO:0005198(structural molecule acti	chr15:101862341-101862400	mm|15qF3
A_51_P363187	Cxcl1	Mus musculus chemokine (C-X	10.983585	1037.1658	A	P	12.619515	94.428709	NM_008176	NM_008176	NM_008176	Mm.21013	14825	ENSMUST00000031327	TC1585292	GO:0002237(response to molecule of	chr5:91321845-91321904	mm|5qE1
A_51_P239654	Nr4a1	Mus musculus nuclear recepto	1339.3695	25365.13	P	P	12.436445	18.938112	NM_010444	NM_010444	NM_010444	Mm.119	15370	ENSMUST00000023779	TC1779970	GO:0001077(RNA polymerase II core	chr15:101105071-101105130	mm|15qF2
A_55_P2048855	Sprr2a2	Mus musculus small proline-ric	87.07726	2232.4545	P	P	12.0451	25.637629	NM_001164787	NM_001164787	NM_001164787	Mm.429851	100303744	ENSMUST00000090871	TC1734054	GO:0003674(molecular function)|GO:	chr3:92058417-92058476	mm|3qF1
A_65_P07626		Mus musculus anti-DNA antibo	2.7531955	1105.8555	A	P	11.794616	401.66254	U30234		U30234	Mm.333124			NP866517		chr6:70648995-70649054	mm|6qC1
A_55_P2325663	Cers3	Mus musculus ceramide synth	8.390256	516.998	A	P	11.434925	61.618859	NM_001164201	NM_001164201	NM_001164201	Mm.380104	545975	ENSMUST00000066475	TC1682009	GO:0003677(DNA binding)|GO:00037	chr7:73968501-73968560	mm|7qC
A_52_P452689	Atf3	Mus musculus activating trans	97.210535	1865.9565	P	P	10.970376	19.195003	NM_007498	NM_007498	NM_007498	Mm.2706	11910	ENSMUST00000027941	TC1582352	GO:0003677(DNA binding)|GO:00037	chr1:192994606-192994547	mm|1qH6
A_51_P436652	Ccl7	Mus musculus chemokine (C-C	100.42312	1828.979	P	P	10.775309	18.212729	NM_013654	NM_013654	NM_013654	Mm.341574	20306	ENSMUST00000021011	TC1632498	GO:0005125(cytokine activity)|GO:00	chr11:81860656-81860715	mm|11qC
A_51_P351384	Upk2	Mus musculus uroplakin 2 (Upk	14.09252	662.0167	P	P	10.682004	46.97646	NM_009476	NM_009476	NM_009476	Mm.4453	22269	ENSMUST00000047740	TC1592071	GO:0005886(plasma membrane)|GO	chr9:44260881-44260822	mm|9qA5.2
A_66_P128079	Krt8	Mus musculus blastocyst blast	421.9288	4703.6815	P	P	10.673571	11.148046	AK166854		AK166854	Mm.358618	16691		TC1635855	GO:0000904(cell morphogenesis invo	chr15:101827197-101827138	mm|15qF3
A_51_P286737	Ccl2	Mus musculus chemokine (C-C	221.10014	3897.6844	P	P	10.654255	17.628593	NM_011333	NM_011333	NM_011333	Mm.290320	20296	ENSMUST00000124479	TC1580703	GO:0001525(angiogenesis)|GO:00016	chr11:81850650-81850709	mm|11qC
A_51_P395899		Mus musculus monoclonal anti	14.11553	610.3441	P	P	10.451957	43.239191	AF433163		AF433163	Mm.333124			TC1700736		chr6:70557780-70557839	mm|6qC1
A_51_P367866	Egr1	Mus musculus early growth res	561.5163	5488.2145	P	P	10.092205	9.7739184	NM_007913	NM_007913	NM_007913	Mm.181959	13653	ENSMUST00000165033	TC1730587	GO:0000122(negative regulation of tr	chr18:35024395-35024454	mm|18qB1
A_55_P2033362	Egr2	Mus musculus early growth res	27.86311	948.0315	P	P	9.7868888	34.024612	NM_010118	NM_010118	NM_010118	Mm.290421	13654	ENSMUST00000048289	TC1578922	GO:0001102(RNA polymerase II activ	chr10:67004798-67004857	mm|10qB5.1
A_55_P2001281	Igh-VJ558	Mus musculus clone J558.B11	13.85686	423.7741	P	P	9.4908946	30.58226	AF296428		AF296428	Mm.335723	16061		NP614370		chr12:116396793-116396734	mm|12qF2
A_51_P139678	Sprr1a	Mus musculus small proline-ric	177.28925	2243.305	P	P	9.4228653	12.653362	NM_009264	NM_009264	NM_009264	Mm.331191	20753	ENSMUST00000054599	TC1583348	GO:0005737(cytoplasm)|GO:0018149	chr3:92288014-92287955	mm|3qF1
A_55_P2119257	Serpine1	Mus musculus serine (or cyste	332.34525	3753.663	P	P	9.0009768	11.294469	NM_008871	NM_008871	NM_008871	Mm.250422	18787	ENSMUST00000077523	NP822114	GO:0002020(protease binding)|GO:00	chr5:137537500-137537441	mm|5qG2
A_55_P2065562	Aldh3a1	Mus musculus aldehyde dehyd	85.746335	964.72965	P	P	8.9866482	11.250972	NM_007436	NM_007436	NM_007436	Mm.4257	11670	ENSMUST00000108716		GO:0001666(response to hypoxia)|G	chr11:61031864-61031920	mm|11qB2
A_55_P2164932	Gsdmc4	Mus musculus gasdermin C4 (G	2.621035	226.7328	A	P	8.7749113	86.505064	NM_028992	NM_028992	NM_028992	Mm.273185	74548	ENSMUST00000063530	NP1487828	GO:0003674(molecular function)|GO:	chr15:63723337-63723278	mm|15qD1
A_55_P1953377			292.3254	2958.9886	P	P	8.5940609	10.122243									chr8:074048674-074048733	mm|8qB3.3
A_66_P103124		Mus musculus hybridoma 4D8	2.3272395	175.30965	A	P	8.5028525	75.329441	AF113109		AF113109	Mm.453038			NP045610		chr12:115538364-115538305	mm|12qF2
A_55_P1993001		Mus musculus clone J558.37 i	27.86443	595.8297	P	P	8.4979497	21.383165	AF303868		AF303868	Mm.489023			NP647897		chr12:116446492-116446433	mm|12qF2
A_55_P2053933	Foxa1	Mus musculus forkhead box A1	2.3919945	177.89265	A	P	8.4776427	74.370008	NM_008259	NM_008259	NM_008259	Mm.4578	15375	ENSMUST00000044380	TC1573059	GO:0000122(negative regulation of tr	chr12:58641823-58641764	mm|12qC1
A_55_P2113051	Fosb	Mus musculus FBJ osteosarco	24.701405	517.2992	P	P	8.4401111	20.942096	NM_008036	NM_008036	NM_008036	Mm.248335	14282	ENSMUST00000003640		GO:0003677(DNA binding)|GO:00036	chr7:19888227-19888168	mm|7qA3
A_55_P1997756	Il6	Mus musculus interleukin 6 (Il6	2.8344825	200.48055	A	P	8.3789239	70.729154	NM_031168	NM_031168	NM_031168	Mm.1019	16193	ENSMUST00000026845	TC1586583	GO:0001781(neutrophil apoptotic pro	chr5:30346367-30346426	mm|5qB1
A_52_P502849	Igkv3-4	Mus musculus immunoglobulin	26.92367	550.74135	P	P	8.3748931	20.455657	BC108385		BC108385	Mm.333124	626347		NP059707		chr6:70622214-70622273	mm|6qC1
A_52_P614207		Mus musculus immunoglobulin	28.29647	553.8913	P	P	8.2527147	19.574572	BC080787		BC080787	Mm.333124			TC1585461		chr6:70557842-70557901	mm|6qC1
A_52_P358406		Mus musculus anti-DNA immu	21.8852	403.61965	P	P	8.0874098	18.442584	U55531		U55531	Mm.422810			NP058789		chr12:116229549-116229490	mm|12qF2
A_55_P2071064	Fer1l4	Mus musculus fer-1-like 4 (C.	3.111919	178.21295	A	P	7.9637155	57.267863	NM_001136556	NM_001136556	NM_001136556	Mm.159241	74562	ENSMUST00000150538	TC1629306	GO:0003674(molecular function)|GO:	chr2:155844936-155844877	mm|2qH1
A_55_P2006261	Krt15	Mus musculus keratin 15 (Krt1	6.020365	319.99575	A	P	7.8170387	53.152218	NM_008469	NM_008469	NM_008469	Mm.440010	16665	ENSMUST00000107411	TC1580208	GO:0005198(structural molecule acti	chr11:99993390-99993331	mm|11qD
A_55_P2046877	Foxq1	Mus musculus forkhead box Q	28.74352	456.79135	P	P	7.6743545	15.891977	NM_008239	NM_008239	NM_008239	Mm.44235	15220	ENSMUST00000042118	TC1585447	GO:0000122(negative regulation of tr	chr13:31652206-31652265	mm|13qA3.2
A_52_P423810	Mt1	Mus musculus NOD-derived C	26.277825	410.05155	P	P	7.6236913	15.604471	AK154954		AK154954	Mm.192991	17748		TC1631988	GO:0005507(copper ion binding)|GO:	chr8:96703844-96703903	mm|8qC5
A_55_P2001274		Mus musculus isolate IgMus11	28.022535	412.46985	P	P	7.4616212	14.71922	AY114394		AY114394	Mm.304472			NP164116		chr12:116996378-116996319	mm|12qF2
A_51_P409349	Trim29	Mus musculus tripartite motif-	4.465912	187.80015	A	P	7.3563272	42.051915	NM_023655	NM_023655	NM_023655	Mm.273277	72169	ENSMUST00000034511	TC1580544	GO:0000122(negative regulation of tr	chr9:43144032-43144092	mm|9qA5.1
A_52_P213483	Ighv1-77	Mus musculus 6E6 monoclonal	66.06171	921.70805	P	P	7.3131166	13.952228	AF045501		AF045501	Mm.313480	619994		NP049389		chr12:117100268-117100209	mm|12qF2
A_55_P2011950		Mus musculus mRNA for immu	44.11904	599.8105	P	P	7.2411985	13.595283	AB159872		AB159872	Mm.357651			NP614310		chr12:117238485-117238426	mm|12qF2
A_55_P2001267		Mus musculus isolate 311 imm	5.558199	206.7643	A	P	7.1152094	37.199874	AY208318		AY208318	Mm.460983			NP590658			
A_51_P513776	Igh-VJ558	Mus musculus clone J558.B10	71.356955	860.00325	P	P	6.9068604	12.052129	AF296427		AF296427	Mm.313465	16061		NP921411		chr12:116745995-116745936	mm|12qF2
A_55_P2138627		Mouse Ig active H-chain (GAC	9.70559	315.6163	A	P	6.850726	32.519023	M32051		M32051	Mm.422809			NP051972		chr12:115630402-115629983	mm|12qF2
A_51_P186856	Krt5	Mus musculus keratin 5 (Krt5).	20.91601	246.28595	P	P	6.8423053	11.774997	NM_027011	NM_027011	NM_027011	Mm.451847	110308	ENSMUST00000023709	TC1575544	GO:0005198(structural molecule acti	chr15:101537573-101537514	mm|15qF2
A_51_P308796	Fosl1	Mus musculus fos-like antigen	4.08847	125.64865	A	P	6.739599	30.732438	NM_010235	NM_010235	NM_010235	Mm.6215	14283	ENSMUST00000025850	TC1580200	GO:0000977(RNA polymerase II regu	chr19:5455496-5455555	mm|19qA

A_52_P489295	Adamts1	Mus musculus a disintegrin-lik	524.9148	1186.328	P	P	3.0262334	2.2600392	NM_009621
A_51_P353252	Mal2	Mus musculus mal, T cell differ	196.4334	443.48485	P	P	3.0223642	2.2576856	NM_178920
A_55_P2061278	Sik1	Mus musculus salt inducible ki	228.13735	513.13215	P	P	3.0084214	2.2492246	NM_010831
A_51_P436817	Dos	Mus musculus downstream of	99.38549	292.93295	P	P	2.9987977	2.9474418	NM_001195268
A_51_P137336	Cdh1	Mus musculus cadherin 1 (Cdh	60.565582	178.35917	P	P	2.9963971	2.9448932	NM_009864
A_55_P2108837	Tuba1c	Mus musculus tubulin, alpha 1	2192.2195	4298.6315	P	P	2.993066	1.9608582	NM_009448
A_55_P2064547	Tuba1c	Mus musculus tubulin, alpha 1	1781.108	3483.8815	P	P	2.9821463	1.9560192	BC022182
A_55_P2089913	Cd6	Mus musculus CD6 antigen (C	99.430135	291.1743	P	P	2.9808412	2.9284311	NM_009852
A_51_P113195	Upk1b	Mus musculus uroplakin 1B (U	129.03607	377.64708	P	P	2.9791798	2.9266784	NM_178924
A_55_P1983508	Nr4a2	Mus musculus nuclear recepto	29.02469	131.72475	P	P	2.9777672	4.5383689	NM_013613
A_55_P2057528	Arl4d	Mus musculus ADP-ribosylatic	395.9022	881.4661	P	P	2.9706693	2.2264744	NM_025404
A_55_P2075459	Dtnb	dystrobrevin, beta [Source:MG	10.844585	48.749455	A	P	2.9590058	4.4952808	ENSMUST00000101637
A_55_P1959308	Rbm41	Mus musculus RNA binding mo	21.90496	98.18188	P	P	2.9532638	4.4821757	NM_001172147
A_51_P295192	Nfkbia	Mus musculus nuclear factor o	3823.575	7704.3905	P	P	2.9496684	2.0149704	NM_010907
A_55_P1984556	Ccl12	Mus musculus chemokine (C-C	126.4574	365.3203	P	P	2.9431071	2.8888804	NM_011331
A_55_P2158018	Rnf39	Mus musculus ring finger prote	37.921185	109.4673	P	P	2.9410173	2.8867057	NM_001099632
A_52_P547612	Tmem30b	Mus musculus transmembrane	11.61295	51.71852	P	P	2.9406503	4.4535213	NM_178715
A_55_P2096762	Arhgdig	Mus musculus Rho GDP dissoc	13.895745	61.81952	P	P	2.9385684	4.4488093	NM_008113
A_51_P254656	Hdc	Mus musculus histidine decarb	93.430465	268.43255	P	P	2.9278811	2.873073	NM_008230
A_55_P2063654	Mup20	Mus musculus major urinary pr	16.09968	71.16764	P	P	2.925986	4.4204382	NM_001012323
A_55_P2105454		M.musculus mRNA for immuno	8.0348945	35.37211	A	P	2.9179048	4.4023117	X87746
A_55_P2007088	Rnf128	Mus musculus ring finger prote	610.3441	1331.7975	P	P	2.895815	2.1820437	NM_023270
A_55_P2020612	Arl4d	Mus musculus ADP-ribosylatic	393.55135	856.3021	P	P	2.8852306	2.1758332	NM_025404
A_52_P108346	Myc	Mus musculus myelocytomato	24.614225	106.50505	P	P	2.8839556	4.3269715	NM_010849
A_51_P300960	Tti2	Mus musculus adult male corp	9.6291045	41.322165	A	P	2.8677124	4.2913819	AK045392
A_55_P1973848	Sema4b	Mus musculus sema domain, ir	49.478265	138.93385	P	P	2.864284	2.8079774	NM_013659
A_55_P2130497		Mus musculus isolate M14H im	30.41934	130.2851	P	P	2.8638532	4.2829693	GQ856057
A_55_P2005549	Tnn	Mus musculus tenascin N (Tnr	15.08939	64.468445	P	P	2.8590101	4.2724355	NM_177839
A_52_P1037027	Gm10664	Mus musculus predicted gene	9.5249	40.670635	A	P	2.8578554	4.2699278	NR_040692
A_51_P367060	Ifrd1	Mus musculus interferon-relat	775.1406	1673.663	P	P	2.8566881	2.1591734	NM_013562
A_55_P2056846	Olfr525	Mus musculus olfactory recept	2.3474975	78.992675	A	P	2.8526489	33.649738	NM_146956
A_52_P638459	Ccl5	Mus musculus chemokine (C-C	2093.2795	3969.601	P	P	2.8452411	1.896355	NM_013653
A_55_P2138306			990.59445	1873.9125	P	P	2.834391	1.891705	
A_51_P487178	Megf11	Mus musculus multiple EGF-lik	7.346578	30.98814	A	P	2.8338085	4.2180373	NM_172522
A_55_P1974014		Mus musculus monoclonal anti	45.126395	125.2001	P	P	2.8309324	2.7744317	AF466768
A_55_P2140751			2156.863	4071.321	P	P	2.824819	1.8876122	NAP112112-1
A_55_P2133907		Mouse immunoglobulin variable	2.3622255	76.39651	A	P	2.8208345	32.340905	X59198
A_55_P2046198	Slc25a25	Mus musculus solute carrier fa	77.53593	213.93885	P	P	2.8156778	2.7592221	NM_146118
A_52_P124173	Zdhhc8	Mus musculus zinc finger, DHH	8.586132	35.830535	A	P	2.8127297	4.1730706	NM_172151
A_51_P258768	Paqr5	Mus musculus progestin and ad	8.978909	37.34324	A	P	2.806085	4.1589953	NM_028748
A_55_P2092296	Fbxo2	Mus musculus F-box protein 2	112.94025	307.30855	P	P	2.7769513	2.7209834	NM_176848
A_55_P2135064	H2-M2	Mus musculus histocompatibili	104.71977	283.28715	P	P	2.7608	2.7051926	NM_008204
A_55_P2065510		Mus musculus clone LBTR5Pa	24.73254	100.39679	P	P	2.7583665	4.0592996	KF208101
A_55_P2051229	Gpr132	Mus musculus G protein-coupl	192.54085	403.6727	P	P	2.7474023	2.0965561	NM_019925
A_52_P77227	Rnf212	PREDICTED: Mus musculus rir	10.395083	41.86971	A	P	2.7430641	4.027838	XM_001476621
A_51_P472932	Atp8b1	Mus musculus ATPase, class I	8.801503	35.09122	A	P	2.7230012	3.9869577	NM_001001488
A_51_P255271	Gltp	Mus musculus glycolipid transf	47.872435	127.6873	P	P	2.7215927	2.6672406	NM_019821
A_55_P1972381	Gjb4	Mus musculus gap junction pro	2.424001	68.659225	A	P	2.7145014	28.324751	NM_008127
A_55_P2103452	Npy1r	Mus musculus neuropeptide Y	298.1236	619.329	P	P	2.7133586	2.0774236	NM_010934
A_51_P469443	8030474K03Rik	RIKEN cDNA 8030474K03 gen	2.4502985	68.78348	A	P	2.7072983	28.07147	ENSMUST00000151231
A_52_P343306	H2-Aa	Mus musculus histocompatibili	4507.2105	8571.6005	P	P	2.7048175	1.9017529	NM_010378
A_55_P2024625	Gm1987	Mus musculus predicted gene	8982.112	17047.485	P	P	2.6963138	1.8979373	NM_001193667
A_52_P93933	Mcl1	Mus musculus myeloid cell leu	387.25975	799.40115	P	P	2.6897362	2.0642505	NM_008562
A_51_P296249	Foxs1	Mus musculus forkhead box S1	194.0746	398.92105	P	P	2.6739675	2.0555037	NM_010226
A_55_P2056236	Pdlim5	Mus musculus PDZ and LIM do	7.59608	29.464375	A	P	2.6689582	3.8788921	NM_019809
A_51_P480241	Elf3	Mus musculus E74-like factor	2.382298	62.475635	A	P	2.652733	26.224945	NM_007921
A_51_P103541	Cacna1s	Mus musculus calcium channe	55.47085	144.16935	P	P	2.6496826	2.599011	NM_001081023
A_55_P1994694	Serinc2	Mus musculus serine incorpora	2.3949285	62.3379	A	P	2.6467227	26.029128	NM_001253386
A_55_P2128511	Ociad2	Mus musculus OCIA domain co	69.193735	179.56275	P	P	2.645474	2.5950724	NM_026950
A_55_P2053838	Tnfaip3	Mus musculus tumor necrosis	161.44155	418.8023	P	P	2.6444789	2.594142	NM_009397
A_52_P286098	Epb4.1l4b	Mus musculus erythrocyte pro	101.32138	262.5001	P	P	2.6408666	2.5907672	NM_019427
A_51_P172853	Cd14	Mus musculus CD14 antigen ((	550.69835	1121.5865	P	P	2.6397714	2.0366622	NM_009841
A_52_P196161	Sh3gl2	Mus musculus SH3-domain GF	52.235055	135.1706	P	P	2.6376192	2.5877373	NM_019535
A_52_P585124	Cxcr4	Mus musculus chemokine (C-X	41.12575	106.20745	P	P	2.6320024	2.5825049	NM_009911
A_66_P106497		Mus musculus anti-DNA antib	27.28252	103.62691	P	P	2.6276597	3.7982893	U29575
A_52_P366525	Coq10b	Mus musculus coenzyme Q10	122.4581	315.64415	P	P	2.6266931	2.5775686	NM_001039710
A_51_P181565	Hbegf	Mus musculus heparin-binding	272.98455	552.4513	P	P	2.6161455	2.0237457	NM_010415
A_55_P2114995			6100.469	11359.025	P	P	2.6153551	1.8619921	NAP111417-1
A_55_P2416534	5033428I22Rik	Mus musculus 13 days embryo	2.398674	59.434845	A	P	2.6072264	24.778209	AK142377
A_52_P1172945	Ankrd34b	Mus musculus ankyrin repeat c	2.3914695	59.12898	A	P	2.6055011	24.724957	NM_175455
A_51_P317941	Perp	Mus musculus PERP, TP53 ap	552.32135	1112.822	P	P	2.5997107	2.014809	NM_022032
A_55_P2131964	Pdia2	Mus musculus protein disulfide	2.494574	60.71756	A	P	2.5929123	24.339851	NM_001081070
A_55_P2025829			8652.4185	16025.15	P	P	2.5928025	1.8521007	
A_51_P390937		Mus musculus clone SMN-1-2	142.89305	363.82935	P	P	2.5926768	2.5461655	JX420695
A_66_P105596		DNA encoding V region of L ch	19.70421	73.44725	P	P	2.5906547	3.7274902	E05351
A_55_P2086079			9.6537255	35.96427	A	P	2.5895669	3.7254291	
A_52_P663904	Lhfpl1	Mus musculus lipoma HMGIC f	2.3746135	56.09563	A	P	2.5689413	23.623057	NM_178358
A_51_P397296	Marveld3	Mus musculus MARVEL (memb	10.470153	38.45857	A	P	2.5617791	3.6731622	NM_028584
A_51_P386870	Sprr2f	Mus musculus small proline-ric	2.522744	58.148175	A	P	2.5492332	23.049574	NM_011472
A_55_P1999162		PREDICTED: Mus musculus pr	66.351395	166.2935	P	P	2.5488345	2.5062548	ENSMUST00000111423
A_55_P1988994		Mus musculus clone 3D1J-05L	43.121515	108.03155	P	P	2.5477569	2.5052818	HQ446714
A_52_P621617	Dnajb4	Mus musculus DnaJ (Hsp40) h	127.2159	317.9159	P	P	2.5408194	2.4990265	NM_027287
A_55_P2136501	Midn	Mus musculus midnolin (Midn),	1240.232	2195.1975	P	P	2.5404747	1.7699894	NM_021565
A_55_P2166618			53.459845	133.40635	P	P	2.5368447	2.4954496	
A_55_P2158990	Jun	Mus musculus Jun oncogene (	2472.8575	4371.794	P	P	2.5352841	1.7679118	NM_010591
A_55_P2071059	Nab2	Mus musculus Ngfi-A binding p	580.11545	1148.0325	P	P	2.5330661	1.9789725	NM_008668
A_55_P2144150	Olfr218	Mus musculus olfactory recept	2.563572	57.672835	A	P	2.5297763	22.497061	NM_001001809
A_55_P2041325	Cep350	Mus musculus centrosomal pro	24.138505	87.216385	P	P	2.5293893	3.6131643	NM_001039184
A_51_P458707	Olfr299	Mus musculus olfactory recept	2.37549	53.37681	A	P	2.5288044	22.46981	NM_001011767
A_52_P165654	Star	Mus musculus steroidogenic ac	88.21345	219.4492	P	P	2.5282211	2.4877068	NM_011485
A_55_P2070992	Aldoc	Mus musculus aldolase C, fruc	208.7274	412.14475	P	P	2.5247768	1.9745599	NM_009657
A_51_P151732	Pkp1	Mus musculus plakophilin 1 (Pl	3.113534	69.584795	A	P	2.524486	22.349136	NM_019645
A_66_P106789		predicted gene 6970 [Source:M	55.91231	138.7745	P	P	2.5218509	2.4820026	ENSMUST00000169692
A_66_P113466		Mus musculus clone AiDWTimi	44.36349	109.9144	P	P	2.5169094	2.4775869	DQ372793
A_55_P2023542	Ccr7	Mus musculus chemokine (C-C	309.56255	609.54705	P	P	2.5144178	1.9690594	NM_007719
A_55_P2086978	Col23a1	Mus musculus collagen, type X	7.9891555	28.59246	A	P	2.5106546	3.5789089	NM_153393
A_51_P383032	Clec4d	Mus musculus C-type lectin do	78.22791	193.09335	P	P	2.506537	2.4683435	NM_010819
A_55_P2186005	Sphk1	Mus musculus sphingosine kina	154.46665	381.04595	P	P	2.5048567	2.4668493	NM_001172472
A_55_P2133057	Pdia2	Mus musculus protein disulfide	2.383154	51.94563	A	P	2.5044259	21.797009	NM_001081070
A_55_P2001262		Mus musculus strain C57BL/6	22.448725	79.989765	P	P	2.5020146	3.5632209	JX120220
A_52_P355084	Metrnl	Mus musculus meteorin, glial c	609.94555	1196.51	P	P	2.5004499	1.9616669	NM_144797
A_66_P126153	1700011M02Rik	Mus musculus RIKEN cDNA 17	9.5015115	33.80483	A	P	2.4990408	3.5578371	NR_073044
A_55_P2052240	Crlf1	Mus musculus cytokine recept	62.00075	152.6224	P	P	2.49897	2.4616218	NM_018827
A_51_P114693	Parm1	Mus musculus prostate androg	209.32068	410.31093	P	P	2.4976769	1.9602026	NM_145562
A_55_P2067505	Slc16a3	Mus musculus solute carrier fa	216.51015	424.17285	P	P	2.4956559	1.9591361	NM_030696
A_55_P1990121	Aqp5	Mus musculus aquaporin 5 (Aq	29.26051	103.47269	P	P	2.4870755	3.5362572	NM_009701
A_55_P2060897		immunoglobulin kappa constan	6276.8715	11329.445	P	P	2.4836177	1.8049509	ENSMUST00000103410
A_55_P1979828	Sprr2g	Mus musculus small proline-ric	2.375746	50.316755	A	P	2.4813735	21.17935	NR_003548
A_55_P2150962	Cpne7	Mus musculus copine VII (Cpne	511.37805	995.801	P	P	2.4731325	1.9472893	NM_170684
A_55_P1967196	Lamb3	Mus musculus laminin, beta 3 (	118.9551	289.4509	P	P	2.4668329	2.4332786	NM_008484
A_55_P2125491	Tnfrsf4	Mus musculus tumor necrosis	75.299565	183.00015	P	P	2.4634281	2.4302949	NM_011659
A_55_P1962937	Trem2	Mus musculus triggering recep	421.1311	817.57935	P	P	2.4618638	1.9413892	NM_001272078
A_55_P2183035	Exoc7	exocyst complex component 7	2.3394355	48.13038	A	P	2.4580994	20.573502	ENSMUST00000124281
A_55_P2029051	Fgd3	FYVE, RhoGEF and PH domain	296.9218	575.8525	P	P	2.4580723	1.939408	ENSMUST00000110087
A_52_P578436		immunoglobulin kappa chain va	343.96865	667.04245	P	P	2.4577767	1.9392536	ENSMUST00000101325
A_55_P2004867	Serpinb2	Mus musculus serine (or cyste	8.4719575	29.47968	A	P	2.4553539	3.4796775	NM_011111
A_51_P371750	Marco	Mus musculus macrophage rec	28.42895	98.682965	P	P	2.4505644	3.4712139	NM_010766
A_52_P652336	Lhx1	LIM homeobox protein 1 [Sour	16.75238	58.01992	P	P	2.446123	3.4633837	ENSMUST00000018842
A_55_P2025687	Muc4	Mus musculus mucin 4 (Muc4),	2.4286215	49.17091	A	P	2.4452481	20.246428	NM_080457
A_52_P30641	Igkv4-86	Mus musculus clone BL6immB	12.706385	43.94485	P	P	2.4433396	3.4584856	DQ372749
A_51_P424338	Nqo1	Mus musculus NAD(P)H dehyd	72.773615	175.51585	P	P	2.4422361	2.4118061	NM_008706
A_55_P2003513	Hsph1	Mus musculus heat shock 105	121.2375	292.1345	P	P	2.4397025	2.4096051	NM_013559
A_51_P497255	Defa4	Mus musculus defensin, alpha,	2.3945545	47.549215	A	P	2.4296824	19.857228	NM_010039
A_51_P285206	Cd3d	Mus musculus CD3 antigen, de	328.20485	631.2503	P	P	2.4271826	1.9233424	NM_013487
A_66_P135452	Clcnka	Mus musculus chloride channe	2.36802	46.832155	A	P	2.4264329	19.776925	NM_024412
A_55_P2133337	March9	membrane-associated ring fing	147.41805	353.2806	P	P	2.4245158	2.3964542	ENSMUST00000040307
A_52_P532227	S1pr1	Mus musculus sphingosine-1-p	401.54595	770.201	P	P	2.4170264	1.9180893	NM_007901
A_55_P1970274	Zscan10	Mus musculus zinc finger and S	8.9903245	30.57074	A	P	2.4100306	3.4004045	NM_001033425
A_55_P1989061	Tsc22d3	Mus musculus TSC22 domain	3378.3845	5801.913	P	P	2.4070798	1.7173631	NM_001077364
A_55_P2035400	Ripk4	Mus musculus receptor-intera	406.21515	776.45135	P	P	2.404109	1.9114288	NM_023663
A_55_P2030899			199.20065	380.4784	P	P	2.4013824	1.9100259	NAP111930-1
A_55_P2136249			2.39332	45.756455	A	P	2.399276	19.118402	NAP061506-1
A_55_P2034227			4798.861	8477.971	P	P	2.3928353	1.7666632	
A_52_P148514	Hpse	Mus musculus heparanase (Hp	19.75812	66.4737	P	P	2.3890801	3.3643737	NM_152803
A_51_P455866	Elf5	Mus musculus E74-like factor	25.202025	84.584265	P	P	2.3843247	3.3562488	NM_010125
A_55_P2078716	Morc4	Mus musculus microrchidia 4 (	8.405875	28.21098	A	P	2.3842392	3.3561027	NM_029413
A_55_P2070869	Lcn2	Mus musculus lipocalin 2 (Lcn2	287.5783	546.75145	P	P	2.3842348	1.9012264	NM_008491
A_51_P418116	Tmem119	Mus musculus transmembrane	1490.13	2544.3675	P	P	2.3815736	1.7074802	NM_146162
A_55_P1985850	Timp1	Mus musculus tissue inhibitor	284.01215	538.7524	P	P	2.375842	1.8969343	NM_001044384
A_55_P2102973			2.3603615	43.76357	A	P	2.3746853	18.541046	
A_55_P2136880	Ppp1r15a	Mus musculus protein phospha	640.10565	1213.5885	P	P	2.373854	1.8959191	NM_008654
A_55_P2127973	Gm9112	Mus musculus predicted gene	25.286035	84.28304	P	P	2.3707633	3.3331853	NM_001177365
A_51_P354126	Reg3g	Mus musculus regenerating isl	2.807848	51.7389	A	P	2.3697171	18.426532	NM_011260
A_55_P2025248	Mxd1	Mus musculus MAX dimerizatio	285.3548	540.24595	P	P	2.3686086	1.8932429	NM_010751
A_51_P326191	Serpina3g	Mus musculus serine (or cyste	489.09735	925.73615	P	P	2.3676302	1.8927442	NM_009251
A_51_P187602	Serpinb5	Mus musculus serine (or cyste	2.637546	48.40458	A	P	2.3664724	18.352127	NM_009257
A_51_P412270	Rapgef6	Mus musculus Rap guanine nu	47.83803	112.2497	P	P	2.3660038	2.3464532	NM_175258
A_55_P2122035	Olfr735	Mus musculus olfactory recept	2.3745435	43.54893	A	P	2.3659387	18.339917	NM_001011754
A_52_P529650	Drd2	Mus musculus dopamine recep	2.3963115	43.89964	A	P	2.365053	18.319672	NM_010077
A_55_P2089488	Coq10b	Mus musculus coenzyme Q10	208.9245	395.0038	P	P	2.3635258	1.8906533	NM_001039710
A_51_P363749	Irf6	Mus musculus interferon regula	44.321585	103.71245	P	P	2.3583598	2.3399987	NM_016851
A_51_P286488	Ier3	Mus musculus immediate early	284.7	537.20985	P	P	2.3562115	1.8869331	NM_133662
A_55_P2098530	Krt40	Mus musculus keratin 40 (Krt4	2.360606	42.660655	A	P	2.3541333	18.071908	NM_001039666
A_55_P2116059	Aim1	Mus musculus absent in melan	259.87665	489.7132	P	P	2.3512355	1.8844063	NM_172393
A_55_P2168383	Myo18b	Mus musculus myosin XVIIIb (M	2.4058565	43.236715	A	P	2.3496629	17.971444	NM_028901
A_51_P383530	Mpzl3	myelin protein zero-like 3 [Sou	2.4043985	43.11169	A	P	2.3478267	17.930343	ENSMUST00000093856
A_55_P2062802		Mus musculus partial mRNA fo	26.66309	87.7495	P	P	2.3457419	3.2910477	AJ277812
A_52_P93467	Sdc4	Mus musculus syndecan 4 (Sd	64.71198	150.71285	P	P	2.3452608	2.3289791	NM_011521
A_55_P1989653	Slco4a1	Mus musculus solute carrier or	256.7659	482.91875	P	P	2.3440716	1.8807745	NM_148933
A_51_P391805	Dkkl1	Mus musculus dickkopf-like 1	43.52112	101.2989	P	P	2.3435937	2.3275803	NM_015789
A_55_P2185860	Prg4	Mus musculus proteoglycan 4	8.927055	29.34089	A	P	2.3431647	3.2867379	NM_021400
A_66_P118124	Fxyd3	Mus musculus FXYD domain-c	132.544	308.0007	P	P	2.3390375	2.3237619	NM_008557
A_55_P2149540	Srf	Mus musculus serum response	142.21945	330.4155	P	P	2.3384611	2.3232793	NM_020493
A_52_P410685	Krt7	Mus musculus keratin 7 (Krt7),	142.6777	331.40655	P	P	2.3378449	2.3227635	NM_033073
A_51_P380078	Fcgbp	Mus musculus Fc fragment of I	8.6381705	28.31017	A	P	2.3375296	3.277334	NM_001122603
A_51_P476820	Calr3	Mus musculus calreticulin 3 (C	8.3837825	27.44598	A	P	2.335347	3.273699	NM_028500
A_51_P378789	Cxcl13	Mus musculus chemokine (C-X	116.27755	269.8193	P	P	2.3351111	2.3204763	NM_018866
A_55_P2171116	Lgals3	Mus musculus lectin, galactose	2207.8065	3729.996	P	P	2.3346784	1.6894578	NM_001145953
A_52_P597461	Skil	Mus musculus SKI-like (Skil), t	45.799595	106.24315	P	P	2.3342303	2.3197399	NM_011386
A_55_P2151178	Krtap19-1	Mus musculus keratin associat	3.3263505	58.57207	A	P	2.3333021	17.608508	NM_130876
A_55_P1971318		AV259773 RIKEN full-length e	2.421212	42.610155	A	P	2.3328547	17.598688	ENSMUST00000181081
A_55_P2143693	Sytl1	Mus musculus synaptotagmin-	135.06405	312.2782	P	P	2.3250458	2.3120749	NM_031393
A_52_P530291	Pim1	Mus musculus proviral integrat	19.102435	62.124965	P	P	2.3223895	3.2522014	NM_008842
A_52_P269482	Prss47	PREDICTED: Mus musculus pr	2.364431	40.70644	A	P	2.315232	17.216167	XM_138671
A_51_P506417	Krt14	Mus musculus keratin 14 (Krt1	16.561795	53.53413	P	P	2.3103704	3.2323869	NM_016958
A_52_P207303	Bmp3	Mus musculus bone morphoger	30.51069	98.32174	P	P	2.3043665	3.2225341	NM_173404
A_51_P501550	Uxs1	UDP-glucuronate decarboxyla	10.784356	34.70592	A	P	2.3017029	3.2181728	ENSMUST00000076997
A_55_P2066180	Hid1	Mus musculus HID1 domain co	290.552	540.0626	P	P	2.3003224	1.8587468	NM_175454
A_51_P153683	Lrrc26	Mus musculus leucine rich rep	2.811752	47.48865	A	P	2.2998623	16.889345	NM_146117
A_55_P2179463	Tnfsf8	Mus musculus tumor necrosis	13.16362	42.296005	P	P	2.2985993	3.2130983	NM_009403
A_55_P2001397			2.3258665	39.18183	A	P	2.2978073	16.846122	
A_66_P124724	Rnf19b	ring finger protein 19B [Source	21.00509	67.37733	P	P	2.2952719	3.2076668	ENSMUST00000030584
A_55_P2135551	Dnase1l2	Mus musculus deoxyribonuclea	30.059105	96.33196	P	P	2.2934836	3.2047514	NM_025718
A_66_P136569		Mus musculus partial mRNA fo	34.905005	111.84275	P	P	2.2931475	3.2042038	AJ277813
A_55_P1980204			10.289947	32.85686	A	P	2.2863222	3.1931031	
A_55_P2039284	Hspb1	Mus musculus heat shock prot	14135.245	24344.175	P	P	2.2849877	1.7222323	NM_013560
A_55_P1969861	Zap70	Mus musculus zeta-chain (TCI	232.26445	429.76218	P	P	2.2834368	1.8503141	NM_009539
A_55_P2176535	Cyp2j5	Mus musculus cytochrome P45	94.93892	215.74805	P	P	2.2771274	2.2724932	NM_010007
A_55_P2162035	Smr2	Mus musculus MSG2alpha sali	2.3672035	38.49546	A	P	2.2695078	16.261999	U82380
A_55_P2122339			2.558357	41.601075	A	P	2.2694514	16.260856	
A_55_P2181888	Tmem54	Mus musculus transmembrane	208.69705	384.3878	P	P	2.2664026	1.8418459	NM_133881
A_55_P2078246	Ppp1r10	Mus musculus protein phospha	14.550015	45.963995	P	P	2.2652256	3.1590342	NM_001163818
A_55_P1978465	H2-Q5	Mus musculus histocompatibili	927.58495	1542.493	P	P	2.2646881	1.6629129	NR_051981
A_55_P2019890	Rasef	Mus musculus RAS and EF har	10.380346	32.717115	A	P	2.2607372	3.151833	NM_001017427
A_55_P2008907	Actg1	Mus musculus actin, gamma, c	10629.115	18191.595	P	P	2.2584885	1.7114873	NM_009609
A_55_P2012899	Vsig4	Mus musculus V-set and immu	98.47279	222.02815	P	P	2.2553334	2.2547157	NM_177789
A_55_P2168321			20.036475	62.972815	P	P	2.2551608	3.1429089	NAP114507-1
A_52_P681310	Plaur	Mus musculus plasminogen act	536.35645	983.97815	P	P	2.2516841	1.8345601	NM_011113
A_55_P2185100	Cage1	Mus musculus cancer antigen	9.3498335	29.3044	A	P	2.2497137	3.1342162	BC082568
A_55_P2034300	Tmem40	Mus musculus transmembrane	278.83495	511.21265	P	P	2.2493107	1.833388	NM_001168256
A_55_P1958165	Ms4a7	Mus musculus membrane-span	1382.24	2288.321	P	P	2.2449868	1.6555164	NM_001276398
A_51_P254646	Jdp2	Mus musculus Jun dimerizatio	663.75035	1215.1915	P	P	2.244057	1.830796	NM_030887
A_52_P115579	Fbxo45	Mus musculus F-box protein 4	29.52827	92.266635	P	P	2.2437258	3.1246881	NM_173439
A_66_P125185	Gm13749	Mus musculus predicted gene	2.377235	37.127305	A	P	2.2370967	15.617852	NR_027824
A_55_P1959425	Slc16a3	Mus musculus solute carrier fa	210.96805	384.7645	P	P	2.2298489	1.8238046	NM_030696
A_51_P247614	6330419J24Rik	Mus musculus RIKEN cDNA 63	43.27004	96.59129	P	P	2.2275949	2.2322903	NR_028086
A_55_P2124976	Grhl1	Mus musculus grainyhead-like	15.70909	48.680975	P	P	2.2274302	3.0989048	NM_145890
A_55_P2075553	Tnfsf18	Mus musculus tumor necrosis	2.3697205	36.542155	A	P	2.2268961	15.420449	NM_183391
A_55_P2117342		Ig V kappa =anti-Sm autoantib	33.45656	103.62918	P	P	2.2264906	3.0974247	S74547
A_55_P2166799			2.3670155	36.4244	A	P	2.2252237	15.388323	
A_55_P1981464	5430410E06Rik	Mus musculus 6 days neonate	13.20377	40.8291	P	P	2.2231897	3.0922305	AK017289
A_55_P1969306	Ranbp3l	Mus musculus RAN binding pro	3.7931675	57.69958	A	P	2.215953	15.211451	NM_198024
A_51_P234253	Sdcbp2	Mus musculus syndecan bindir	44.450845	98.78491	P	P	2.2151983	2.2223404	NM_145535
A_55_P2012006	Zfp735	Mus musculus zinc finger prote	2.458823	37.21236	A	P	2.2118709	15.134217	NM_001126489
A_52_P345237	Olfr564	Mus musculus olfactory recept	2.4833055	37.57556	A	P	2.2117147	15.131268	NM_146359
A_55_P1979973	Nuak2	Mus musculus NUAK family, S	71.932915	159.3207	P	P	2.2058307	2.2148512	NM_001195025
A_51_P521176	Rassf7	Mus musculus Ras association	248.06955	449.37255	P	P	2.2046654	1.8114781	NM_025886
A_55_P1983488	Lsr	Mus musculus lipolysis stimula	472.80845	855.12285	P	P	2.1987671	1.8086031	NM_017405
A_55_P2184841	Olfr828	Mus musculus olfactory recept	2.419238	35.9073	A	P	2.1962573	14.842401	NM_146605
A_55_P1966608			56.616405	124.76985	P	P	2.1919192	2.2037756	NAP059589-1
A_55_P2116650		Mus musculus lung RCB-0558	2554.691	4175.788	P	P	2.1886776	1.634557	AK144717
A_52_P145539	Tshr	Mus musculus thyroid stimulat	10.499915	31.839675	A	P	2.1847471	3.0323745	NM_011648
A_55_P1994132	Tmem184a	transmembrane protein 184a [	51.88979	113.95915	P	P	2.1823342	2.1961767	ENSMUST00000110832
A_51_P304490	Olfr12	Mus musculus olfactory recept	2.368217	34.36558	A	P	2.1781579	14.511162	NM_206896
A_51_P101460	Dsp	Mus musculus desmoplakin (Ds	3.6283828	52.649174	A	P	2.1781141	14.510369	NM_023842
A_55_P1964408	Cryab	Mus musculus crystallin, alpha	2488.5415	4057.946	P	P	2.1781078	1.6306523	NM_009964
A_55_P2162945	Nit1	nitrilase 1 [Source:MGI Symbo	10.14316	30.6394	A	P	2.177158	3.0206959	ENSMUST00000146428
A_52_P108243	Pde1c	Mus musculus phosphodiestera	2.5181615	36.46969	A	P	2.1765815	14.482665	NM_011054
A_66_P131433		Q7M6U4 MOUSE (Q7M6U4) G	59.466305	130.207	P	P	2.1740026	2.1895929	TC1600187
A_55_P2044518	Gm3961	Mus musculus adult male hypo	2.6306945	37.76814	A	P	2.1695771	14.356718	AK038723
A_55_P1964598	Hsd17b7	hydroxysteroid (17-beta) dehy	9.5319265	28.67014	A	P	2.1687446	3.0078012	ENSMUST00000111353
A_55_P2097264	Pth2	Mus musculus parathyroid hor	2.9889005	42.85115	A	P	2.1684616	14.33676	NM_053256
A_55_P2397999	4930465M20Rik	Mus musculus RIKEN cDNA 49	2.4480965	34.94062	A	P	2.1648628	14.272566	NR_045973
A_52_P12877	Hspa8	Mus musculus heat shock prot	1444.8175	2348.7395	P	P	2.1644766	1.6256306	NM_031165
A_51_P367780	Adamtsl2	Mus musculus ADAMTS-like 2	215.63805	386.3771	P	P	2.1640747	1.7917854	NM_029981
A_51_P167263	Cd5	Mus musculus CD5 antigen (C	97.053935	211.72505	P	P	2.1637517	2.1815195	NM_007650
A_55_P2078680	Tpm1	Mus musculus tropomyosin 1,	95.19668	207.55485	P	P	2.1621669	2.180274	NM_001164251
A_55_P1983448	S100a4	Mus musculus S100 calcium bi	1921.034	3118.9955	P	P	2.1589594	1.6236024	NM_011311
A_51_P212782	Il1b	Mus musculus interleukin 1 bet	103.6002	225.34802	P	P	2.1556627	2.1751698	NM_008361
A_55_P2025820	Plcb4	Mus musculus adult female va	46.650355	101.47134	P	P	2.1556326	2.1751461	AK036896
A_52_P587738	P2ry2	Mus musculus purinergic recep	55.38068	120.25485	P	P	2.1508778	2.1714224	NM_008773
A_55_P2129449	Sult3a1	Mus musculus sulfotransferase	2.6749705	37.32603	A	P	2.1467499	13.95381	NM_020565
A_55_P2206605	5830444B04Rik	Mus musculus RIKEN cDNA 58	15.844705	47.07231	P	P	2.1444366	2.9708543	NR_102283
A_55_P2282176	4933434P08Rik	Mus musculus adult male testi	2.4613195	34.19422	A	P	2.1432266	13.892638	AK017062
A_55_P2128443	Gm10673	Mus musculus 16 days neonat	14.353245	42.58467	P	P	2.1418183	2.9669019	AK137946
A_51_P307964	Krt13	Mus musculus keratin 13 (Krt1	10.526588	31.18828	A	P	2.139104	2.96281	NM_010662
A_55_P2095171			2.331385	32.19807	A	P	2.1384832	13.810705	
A_55_P2427085		RIKEN cDNA C030005K15 ger	3.6095685	49.714865	A	P	2.1362953	13.773077	ENSMUST00000166373
A_52_P481957	Grem1	Mus musculus gremlin 1 (Grem	3.0691068	42.161885	A	P	2.1342218	13.73751	NM_011824
A_55_P2096674	Ranbp3l	Mus musculus RAN binding pro	3.5053215	48.04991	A	P	2.13248	13.707704	NM_198024
A_52_P642239	Pik3ap1	Mus musculus phosphoinositid	14.604879	43.096685	P	P	2.1311433	2.9508417	NM_031376
A_55_P2053439	Ivl	Mus musculus involucrin (Ivl),	2.8582955	38.96035	A	P	2.1279579	13.630624	NM_008412
A_55_P2077098	Pof1b	Mus musculus premature ovari	2.640142	35.97628	A	P	2.1277238	13.626646	NM_181579
A_51_P111164	Rnd1	Mus musculus Rho family GTP	184.7409	397.5879	P	P	2.1261227	2.1521379	NM_172612
A_55_P1981401			3.63417	49.27972	A	P	2.1237982	13.560103	
A_51_P475748	Ivl	Mus musculus involucrin (Ivl),	3.942478	53.44972	A	P	2.1236379	13.557392	NM_008412
A_55_P2066440	Myh3	Mus musculus myosin, heavy p	2.522774	34.086276	A	P	2.1209144	13.511427	NM_001099635
A_55_P1994962	B230209K01Rik	Mus musculus RIKEN cDNA B2	2.390439	32.284565	A	P	2.1205747	13.505705	NR_038008
A_55_P2011570			2.485505	33.511935	A	P	2.1192223	13.482948	
A_51_P239737	Pigr	Mus musculus polymeric immu	16.7986	49.23309	P	P	2.1177303	2.9307853	NM_011082
A_55_P2079535	Gm10839	PREDICTED: Mus musculus pr	2.356446	31.693655	A	P	2.1172465	13.449769	XR_141261
A_55_P2105507	Gm4681	PREDICTED: Mus musculus pr	3.04329	40.92652	A	P	2.117148	13.448117	XR_168533
A_55_P2013612	Gm11677	PREDICTED: Mus musculus pr	16.062795	46.969375	P	P	2.1132455	2.9241097	XM_894989
A_55_P2136289	Ripk4	receptor-interacting serine-th	428.2894	756.8712	P	P	2.1127597	1.7671957	ENSMUST00000019386
A_55_P1953788	Itk	Mus musculus IL2 inducible T	94.79636	202.9906	P	P	2.1121559	2.1413333	NM_001281965
A_55_P2395179	AA408954	EST03446 Mouse 7.5 dpc emb	2.366693	31.49521	A	P	2.1087299	13.307687	AA408954
A_52_P512575	Hopx	Mus musculus HOP homeobox	226.4948	399.63915	P	P	2.1069892	1.7644518	NM_175606
A_52_P382676	Ighv6-6	Mouse Ig heavy chain mRNA V	2.5022495	33.189495	A	P	2.1060847	13.263863	M59922
A_55_P2011286	Hopx	Mus musculus HOP homeobox	144.88445	309.46655	P	P	2.1051762	2.1359542	NM_175606
A_55_P2095047	Mat2a	Mus musculus methionine ade	207.8835	366.58585	P	P	2.1048163	1.7634197	NM_145569
A_51_P450788		M.musculus mRNA for 11E7-1	3.246323	42.96779	A	P	2.1043883	13.235833	X05391
A_55_P1986608	D830036C21Rik	PREDICTED: Mus musculus RI	2.385185	31.527355	A	P	2.1033065	13.217991	XM_003945770
A_66_P118165			2416.117	3873.534	P	P	2.1030892	1.6032063	NAP112345-1
A_55_P2008538	Chmp4b	Mus musculus charged multive	303.0484	533.72755	P	P	2.1001301	1.7611957	NM_029362
A_55_P2012704	Kcnh7	Mus musculus potassium volta	2.4817085	32.55055	A	P	2.0971061	13.116186	NM_133207
A_51_P104418	Dusp10	Mus musculus dual specificity	11.31373	32.806515	A	P	2.0967646	2.8997081	NM_022019
A_55_P2071921	LOC101056369	PREDICTED: Mus musculus ol	2.4611825	32.228235	A	P	2.0957862	13.094614	XM_003946032
A_52_P668686	A530088E08Rik	Mus musculus RIKEN cDNA A5	2.813398	36.68223	A	P	2.0923366	13.038408	NR_029458
A_51_P423976	Crem	Mus musculus cAMP responsiv	81.36936	172.7156	P	P	2.0877881	2.1226122	NM_013498
A_55_P1973588	Slc9a4	Mus musculus solute carrier fa	2.347005	30.30975	A	P	2.0846621	12.914225	NM_177084
A_51_P323812	Slc6a12	Mus musculus solute carrier fa	18.893175	54.421545	P	P	2.0836845	2.880487	NM_133661
A_55_P2023391	Grhl3	Mus musculus grainyhead-like	2.8832985	37.16535	A	P	2.0831485	12.889872	NM_001013756
A_51_P115666	Fam83h	Mus musculus family with sequ	154.14855	326.1683	P	P	2.0790447	2.1159349	NM_134087
A_55_P2192729	5830469G19Rik	Mus musculus adult male thym	69.37575	146.6322	P	P	2.0759735	2.1135944	AK018045
A_55_P2025490	Tnfrsf18	Mus musculus tumor necrosis	111.09435	234.8046	P	P	2.0759286	2.1135602	NM_009400
A_52_P452667	Prom2	Mus musculus prominin 2 (Pro	2.819145	35.92865	A	P	2.0740542	12.74452	NM_178047
A_52_P546162		Mus musculus clone nat28L im	63.34468	133.669	P	P	2.0714936	2.1101851	HQ446585
A_51_P155313	Gsto1	Mus musculus glutathione S-tr	1643.6645	2615.4935	P	P	2.0700274	1.5912575	NM_010362
A_55_P2146947	Olfr693	Mus musculus olfactory recept	2.3758085	30.11637	A	P	2.0697476	12.676262	NM_146453
A_55_P2017681	C5ar1	Mus musculus complement cor	48.466125	102.19272	P	P	2.0693281	2.108539	NM_001173550

A_51_P139108	Cpxm1	Mus musculus carboxypeptidas	1301.694	2070.6165	P	P	2.068504	1.5907091	NM_019696	NM_019696	NM_019696	Mm.112701	56264	ENSMUST00000130533	TC1579793	GO:0004180(carboxypeptidase activ	chr2:130216670-130216611	mm	2qF1
A_55_P2129975	Rabgef1	Mus musculus RAB guanine nu	299.00405	521.5114	P	P	2.0640389	1.7441617	NM_019983	NM_019983	NM_019983	Mm.288639	56715	ENSMUST00000026390	TC1578024	GO:0001933(negative regulation of p	chr5:130690147-130690206	mm	5qG1.3
A_52_P520495	Vcam1	Mus musculus vascular cell ad	86.42993	181.68165	P	P	2.0607995	2.1020687	NM_011693	NM_011693	NM_011693	Mm.76649	22329	ENSMUST00000029574	TC1582765	GO:0002102(podosome)|GO:0005178	chr3:115829012-115828953	mm	3qG1
A_55_P1959218	Usp51	Mus musculus ubiquitin specifi	2.5124275	31.439075	A	P	2.0593795	12.513426	NM_001137547	NM_001137547	NM_001137547	Mm.426169	635253	ENSMUST00000095755	TC1688399	GO:0003674(molecular function)|GO:	chrX:149443874-149443933	mm	XqF3
A_55_P2107154	Olfr623	Mus musculus olfactory recept	2.328474	29.07601	A	P	2.057694	12.487153	NM_147122	NM_147122	NM_147122	Mm.377863	259126	ENSMUST00000068531	TC1599974	GO:0004871(signal transducer activi	chr7:110809187-110809128	mm	7qE3
A_55_P2157809	Gm12	Mus musculus predicted gene	2.343759	29.26051	A	P	2.0575196	12.484436	NM_001195544	NM_001195544	NM_001195544	Mm.342821	192852				chr11:98460767-98460826	mm	11qD
A_55_P2176792	Sh3gl3	Mus musculus SH3-domain GF	10.164411	28.873005	A	P	2.0562591	2.8405979	NM_017400	NM_017400	NM_017400	Mm.736	20408	ENSMUST00000032874	TC1595258	GO:0005515(protein binding)|GO:000	chr7:89455864-89455923	mm	7qD3
A_55_P2038187	BC051019	Mus musculus cDNA sequence	2182.36	3461.549	P	P	2.0558176	1.5861494	NM_001040700	NM_001040700	NM_001040700	Mm.358682	57355	ENSMUST00000033334	NP377898	GO:0003674(molecular function)|GO:	chr7:116859573-116859514	mm	7qE3
A_52_P454921		maestro heat-like repeat famil	2.8881875	35.96126	A	P	2.0553787	12.451151	ENSMUST00000133030	XR_033650	XR_033650	Mm.159735		ENSMUST00000133030	TC1648540		chr1:138078493-138078434	mm	1qE4
A_51_P477121	Pmaip1	Mus musculus phorbol-12-myr	93.2704	195.6572	P	P	2.0550813	2.0977416	NM_021451	NM_021451	NM_021451	Mm.271878	58801	ENSMUST00000025399	TC1597591	GO:0001836(release of cytochrome c	chr18:66625088-66625147	mm	18qE1
A_52_P322141	Ccdc88b	Mus musculus coiled-coil dom	992.2817	1573.243	P	P	2.0539526	1.5854802	NM_001081291	NM_001081291	NM_001081291	Mm.329596	78317	ENSMUST00000113440	TC1586524	GO:0003674(molecular function)|GO:	chr19:6919178-6919119	mm	19qA
A_55_P2112302			4250.8805	6928.8305	P	P	2.0518742	1.6299754									chr17:054080232-054080173	mm	17qC
A_51_P357744	Jund	Mus musculus Jun proto-onco	3739.569	5921.6935	P	P	2.0484936	1.583523	NM_010592	NM_010592	NM_010592	Mm.419851	16478	ENSMUST00000095267	TC1575686	GO:0002076(osteoblast development	chr8:73224436-73224495	mm	8qB3.3
A_55_P2009787	Atp1a4	Mus musculus ATPase, Na+/K	9.0328835	25.55334	A	P	2.0481599	2.8289239	NM_013734	NM_013734	NM_013734	Mm.482392	27222	ENSMUST00000111243	TC1638247	GO:0000166(nucleotide binding)|GO:0	chr1:174153709-174153650	mm	1qH3
A_51_P233797	Adh7	Mus musculus alcohol dehydro	2.4256705	29.906035	A	P	2.0474711	12.328977	NM_009626	NM_009626	NM_009626	Mm.8473	11529	ENSMUST00000090171	TC1583015	GO:0001523(retinoid metabolic proce	chr3:137894495-137894554	mm	3qG3
A_55_P2039532	Pax8	Mus musculus paired box gene	26.034355	73.48279	P	P	2.0437108	2.8225316	NM_011040	NM_011040	NM_011040	Mm.2533	18510	ENSMUST00000028355	TC1582701	GO:0000979(RNA polymerase II core	chr2:24276206-24276147	mm	2qA3
A_51_P256246	Tspan13	Mus musculus tetraspanin 13 (	775.57675	1343.2915	P	P	2.0380344	1.7319904	NM_025359	NM_025359	NM_025359	Mm.254663	66109	ENSMUST00000020896	TC1572186	GO:0003674(molecular function)|GO:	chr12:36741672-36741613	mm	12qA3
A_55_P2161278	Spata2l	Mus musculus 10 days neonat	10.731785	30.179735	A	P	2.0364862	2.8121822	AK139339		AK139339	Mm.41459	78779			GO:0003674(molecular function)|GO:	chr8:125754482-125754423	mm	8qE1
A_55_P2114994			14874.975	24157.635	P	P	2.0364423	1.6240454	NAP111417-1								chr8:047316001-047316060	mm	8qB1.1
A_51_P153734	Kif23	Mus musculus 13 days embryo	2.51365	30.49765	A	P	2.0346093	12.132815	AK013939		AK013939		71819			GO:0000166(nucleotide binding)|GO:0	chr9:61775155-61775096	mm	9qB
A_51_P112223	Gsta4	Mus musculus glutathione S-tr	312.1238	539.82526	P	P	2.0327401	1.7295229	NM_010357	NM_010357	NM_010357	Mm.2662	14860	ENSMUST00000034903	TC1574931	GO:0004364(glutathione transferase	chr9:78046172-78047344	mm	9qE1
A_55_P2121240	Syt6	Mus musculus synaptotagmin V	2.6664315	32.22374	A	P	2.0314406	12.084968	NM_018800	NM_018800	NM_018800	Mm.88818	54524	ENSMUST00000151985	NP378651	GO:0005215(transporter activity)|GC	chr3:103424769-103424828	mm	3qF2.2
A_55_P1979978			2.3417295	28.283895	A	P	2.0309918	12.078208	NAP071037-1								chrX:089485536-089485595	mm	XqC2
A_55_P2017054		immunoglobulin heavy variable	2.9630525	35.69023	A	P	2.0287899	12.045089	ENSMUST00000177949					ENSMUST00000177949			chr12:115644933-115644874	mm	12qF2
A_55_P1969775	Pdhb	Mus musculus 13 days embryo	3.73993	44.997405	A	P	2.0278925	12.031617	AK084507		AK084507		68263		TC1655926	GO:0003824(catalytic activity)|GO:0	chr14:9003664-9003605	mm	14qA1
A_52_P476731	Fam110c	Mus musculus family with sequ	46.246925	96.02496	P	P	2.0266425	2.0763534	NM_027828	NM_027828	NM_027828	Mm.248938	104943	ENSMUST00000041133	TC1581438	GO:0005634(nucleus)|GO:0005737(c	chr12:31764490-31764549	mm	12qA2
A_55_P1999033		Mus musculus CB17 SCID imm	2.353536	28.086775	A	P	2.0213504	11.933862	U22990		U22990	Mm.423574			NP054145		chr12:116886282-116886223	mm	12qF2
A_55_P1976743	Gm15753	PREDICTED: Mus musculus pr	62.61191	129.7174	P	P	2.0205084	2.0717688	XR_142153	XR_142153	XR_142153	Mm.394746	624083		TC1672344		chr5:138161120-138161061	mm	5qG2
A_55_P2031021	Bex1	Mus musculus brain expressed	13.41549	37.417825	P	P	2.0203128	2.7891508	NM_009052	NM_009052	NM_009052	Mm.422943	19716	ENSMUST00000113118	TC1626207	GO:0002052(positive regulation of ne	chrX:132674650-132674709	mm	XqF1
A_51_P270949	Hist1h1b	Mus musculus histone cluster	130.06365	269.43835	P	P	2.0202668	2.0715884	NM_020034	NM_020034	NM_020034	Mm.221314	56702	ENSMUST00000080511	TC1610412	GO:0000786(nucleosome)|GO:00079	chr13:21871982-21871923	mm	13qA3.1
A_55_P2035018	Clec4n	Mus musculus C-type lectin d	319.10895	549.9746	P	P	2.0197203	1.7234697	NM_020001	NM_020001	NM_020001	Mm.271782	56620	ENSMUST00000151714	NP211293	GO:0005515(protein binding)|GO:001	chr6:123196969-123197028	mm	6qF2
A_55_P1969176			2.562524	30.50858	A	P	2.0194541	11.905676									chr7:149473931-149473990	mm	7qF5
A_55_P2065506		Mus musculus strain C57BL/6	11.26815	31.397205	A	P	2.0183491	2.7863673	JX120262		JX120262	Mm.425757			NP614286		chr12:116118334-116118275	mm	12qF2
A_55_P2019601	Csprs	Mus musculus component of S	569.7543	981.1453	P	P	2.0166598	1.7220498	NM_033616	NM_033616	NM_033616	Mm.362644	114564	ENSMUST00000078827	TC1582841	GO:0005634(nucleus)	chr8_random:111631-111572		
A_55_P1984981		AGENCOURT 10726009 NIH M	3.570788	42.10468	A	P	2.0117214	11.791425	CA458452		CA458452	Mm.332806					chr5:143659536-143659595	mm	5qG2
A_51_P220993	Pfn1	Mus musculus profilin 1 (Pfn1)	632.7856	1087.6405	P	P	2.0096745	1.7188136	NM_011072	NM_011072	NM_011072	Mm.2647	18643	ENSMUST00000178254	TC1663639	GO:0001843(neural tube closure)|GO	chr11:70466312-70465688	mm	11qB3
A_55_P2010557			2.4284355	28.5396	A	P	2.0090532	11.752258									chr7:023771002-023770943	mm	7qA3
A_55_P2073617	Fgf6	Mus musculus fibroblast growt	1051.6152	1650.3	P	P	2.0086199	1.5693003	M92416		M92416	Mm.3403	14177			GO:0001502(cartilage condensation)	chr6:126978138-126978197	mm	6qF3
A_66_P124052	Bok	Mus musculus BCL2-related o	3886.933	6099.045	P	P	2.0080983	1.569115	NM_016778	NM_016778	NM_016778	Mm.3295	51800	ENSMUST00000027499	TC1577014	GO:0005634(nucleus)|GO:0005737(c	chr1:095592276-095592335	mm	1qD
A_55_P2072284	Dnaaf3	Mus musculus dynein, axonem	105.56591	217.63	P	P	2.0067949	2.0615558	NM_001033548	NM_001033548	NM_001033548	Mm.425359	436022	ENSMUST00000094897	TC1587494	GO:0003674(molecular function)|GO:	chr7:4474635-4474576	mm	7qA1
A_55_P2041910			1592.7645	2497.3605	P	P	2.0047899	1.5679408	NAP029396-1								chr15:073911512-073911453	mm	15qD3
A_55_P2059288			2.4686695	28.830245	A	P	2.0040013	11.678455									chr14:053507821-053507880	mm	14qC2
A_51_P239203	Mapk13	Mus musculus mitogen-activat	59.59344	122.7132	P	P	2.0035856	2.059173	NM_011950	NM_011950	NM_011950	Mm.27970	26415	ENSMUST00000129096	TC1581117	GO:0000165(MAPK cascade)|GO:000	chr17:28915417-28915476	mm	17qA3.3
A_51_P249118	Abcb4	Mus musculus ATP-binding ca	250.23295	429.17645	P	P	2.0016592	1.7151077	NM_008830	NM_008830	NM_008830	Mm.297825	18670	ENSMUST00000003717	TC1585725	GO:0000139(Golgi membrane)|GO:00	chr5:8959108-8959167	mm	5qA1
A_65_P13270	Csnk1g1	Mus musculus casein kinase 1	40.86879	14.749615	P	P	-2.001387	0.3609017	NM_173185	NM_173185	NM_173185	Mm.325816	214897	ENSMUST00000034949	TC1603373	GO:0000166(nucleotide binding)|GO:0	chr9:65890804-65890863	mm	9qC
A_55_P2159850		603275854F1 NCI_CGAP_Mam	4559.974	2887.512	P	P	-2.002271	0.6332299	BI648893		BI648893	Mm.355478					chr6:22135266-22135344	mm	6qA3.1
A_55_P1958344	Harbi1	harbinger transposase derived	41.322165	14.895235	P	P	-2.003763	0.360466	ENSMUST00000111322		AK041747	Mm.130331	241547	ENSMUST00000111322	TC1629698	GO:0003674(molecular function)|GO:	chr2:91558805-91558864	mm	2qE1
A_55_P2394490	D630004K10Rik	Mus musculus 0 day neonate h	300.0436	145.6985	P	P	-2.005457	0.4855911	AK052609		AK052609	Mm.458462	103210		TC1609665		chr10:124656416-124656357	mm	10qD3
A_55_P1979743	Uqcr11	Mus musculus ubiquinol-cytoc	23429.055	14645.885	P	P	-2.006	0.6251163	BC034408		BC034408	Mm.379119	66594		TC1702062	GO:0005739(mitochondrion)|GO:0005	chr10:79865796-79865737	mm	10qC1
A_55_P2284560		predicted gene 14296 [Source	33.91971	12.189455	P	A	-2.009795	0.359362	ENSMUST00000134982		BC090643	Mm.436749		ENSMUST00000134982	TC1650809		chr2:175433018-175432959	mm	2qH4
A_55_P2080880	Clcnkb	Mus musculus chloride channe	2060.4695	1302.212	P	P	-2.010879	0.6319977	NM_019701	NM_019701	NM_019701	Mm.24882	56365	ENSMUST00000105788	NP424864	GO:0005216(ion channel activity)|GC	chr4:140960334-140960275	mm	4qE1
A_55_P2164849		predicted gene 6811 [Source:M	139.7009	67.451815	P	P	-2.02128	0.4828302	ENSMUST00000169389					ENSMUST00000169389			chr17:21231478-21231537	mm	17qA3.2
A_55_P2179299	Ceacam13	Mus musculus carcinoembryor	30.957405	2.3839685	P	A	-2.022952	0.077008	NM_027210	NM_027210	NM_027210	Mm.46086	69785	ENSMUST00000108488	TC1585315	GO:0003674(molecular function)|GO:	chr7:18604511-18604569	mm	7qA2
A_55_P2139087	Slc22a28	Mus musculus solute carrier fa	1994.5995	1256.6935	P	P	-2.024534	0.630048	NM_001013820	NM_001013820	NM_001013820	Mm.24053	434674	ENSMUST00000065651	TC1588077	GO:0003674(molecular function)|GO:	chr19:8136808-8136749	mm	19qA
A_51_P250123	Zfp763	Mus musculus zinc finger prote	42.75079	15.247245	P	P	-2.024671	0.3566541	NM_028543	NM_028543	NM_028543	Mm.129453	73451	ENSMUST00000087654	TC1581512	GO:0003674(molecular function)|GO:	chr17:33154243-33154184	mm	17qB1
A_51_P161308	Slc22a2	Mus musculus solute carrier fa	93.188755	44.861565	P	P	-2.029481	0.4814053	NM_013667	NM_013667	NM_013667	Mm.17322	20518	ENSMUST00000046959	TC1583143	GO:0005215(transporter activity)|GC	chr17:12821245-12821304	mm	17qA1
A_52_P59681	Hrsp12	Mus musculus heat-responsive	590.38115	341.88175	P	P	-2.030367	0.5790865	NM_008287	NM_008287	NM_008287	Mm.491154	15473	ENSMUST00000125286	TC1741899	GO:0004518(nuclease activity)|GO:0	chr15:34414139-34414080	mm	15qB3.1
A_51_P455647	Car2	Mus musculus carbonic anhydr	3242.021	2038.7655	P	P	-2.032901	0.6288564	NM_009801	NM_009801	NM_009801	Mm.1186	12349	ENSMUST00000029078	TC1714886	GO:0002009(morphogenesis of an ep	chr3:14900206-14900265	mm	3qA1
A_55_P1952658	Gm3772	Mus musculus in vitro fertilizec	37.85233	2.8610273	P	A	-2.037921	0.0755839	AK135955		AK135955	Mm.412604	100042291			GO:0003674(molecular function)|GO:	chr13:70258755-70258814	mm	13qC1
A_51_P302856	Ugt2b37	Mus musculus UDP glucuronos	606.5893	350.42545	P	P	-2.039282	0.577698	NM_053215	NM_053215	NM_053215	Mm.160362	112417	ENSMUST00000075858	TC1606243	GO:0005743(mitochondrial inner mer	chr5:87669766-87669707	mm	5qE1
A_51_P264495	Pgam2	Mus musculus phosphoglycera	185.3947	88.88466	P	P	-2.040864	0.4794347	NM_018870	NM_018870	NM_018870	Mm.219627	56012	ENSMUST00000020768	TC1578210	GO:0003824(catalytic activity)|GO:0	chr11:5703040-5701822	mm	11qA1
A_55_P2179246			238.19655	114.1043	P	P	-2.043183	0.4790342									chr14:099671720-099671661	mm	14qE2.2
A_55_P2073248	Slc25a34	Mus musculus solute carrier fa	109.846	52.59753	P	P	-2.044368	0.4788297	NM_001013780	NM_001013780	NM_001013780	Mm.295682	384071	ENSMUST00000038661	TC1585177	GO:0003674(molecular function)|GO:	chr4:141174806-141174747	mm	4qE1
A_55_P2063785	Ank1	Mus musculus ankyrin 1, eryth	29.962115	10.574895	P	A	-2.045247	0.3529422	NM_001110783	NM_001110783	NM_001110783	Mm.334444	11733	ENSMUST00000033947	TC1582131	GO:0005634(nucleus)|GO:0005737(c	chr8:24259068-24259127	mm	8qA2
A_66_P131235	Gucy2g	Mus musculus guanylate cycla	36.93092	2.7501685	P	A	-2.049849	0.0744679	NM_001081076	NM_001081076	NM_001081076	Mm.489987	73707		TC1598443	GO:0000166(nucleotide binding)|GO:0	chr19:55272682-55272623	mm	19qD2
A_55_P2248330		RIKEN cDNA 2700068H02 gen	98.854145	34.77703	P	P	-2.051614	0.3518014	ENSMUST00000137882		AK012503	Mm.396500		ENSMUST00000137882	TC1748971		chr4:106227360-106227301	mm	4qC7
A_55_P2358027			31.83593	2.3588615	P	A	-2.053883	0.0740943									chr6:049078702-049078761	mm	6qB2.3
A_52_P578085	Zfp708	Mus musculus zinc finger prote	27.476415	9.6195435	P	A	-2.061138	0.3501018	NM_001012325	NM_001012325	NM_001012325	Mm.458849	432769	ENSMUST00000109743	TC1602461	GO:0003674(molecular function)|GO:	chr13:67170739-67170680	mm	13qB3
A_55_P2169099	Hoxc12	Mus musculus homeobox C12	56.760573	19.822743	P	P	-2.066018	0.3492344	NM_010463	NM_010463	NM_010463	Mm.221179	15421	ENSMUST00000055562	TC1768816	GO:0003674(molecular function)|GO:	chr15:102768889-102768948	mm	15qF3
A_52_P325527	Edil3	Mus musculus EGF-like repeat	95.6083	45.37305	P	P	-2.069152	0.4745723	NM_010103	NM_010103	NM_010103	Mm.125580	13612	ENSMUST00000081769		GO:0005509(calcium ion binding)|GO	chr13:89461799-89461858	mm	13qC3
A_51_P356967	Lgi3	Mus musculus leucine-rich rep	39.481155	2.8526815	P	A	-2.074049	0.0722543	NM_145219	NM_145219	NM_145219	Mm.41708	213469	ENSMUST00000047331	TC1618809	GO:0003824(catalytic activity)|GO:0	chr14:70937619-70937678	mm	14qD2
A_55_P1959973	Dcaf12l2	Mus musculus DDB1 and CUL	3156.507	1966.2815	P	P	-2.07475	0.6229296	NM_175539	NM_175539	NM_175539	Mm.55270	245403	ENSMUST00000115057	TC1588022	GO:0003674(molecular function)|GO:	chrX:41719518-41719459	mm	XqA4
A_51_P290685	Ppp6r3	Mus musculus protein phospha	96.175375	45.54475	P	P	-2.075081	0.4735594	NM_029456	NM_029456	NM_029456	Mm.284686	52036	ENSMUST00000113997	TC1572454	GO:0005575(cellular component)|GO	chr19:3455909-3455850	mm	19qA
A_52_P484956	Nnat	Mus musculus neuronatin (Nna	567.3111	324.4876	P	P	-2.076255	0.5719747	NM_010923	NM_010923	NM_010923	Mm.34330	18111	ENSMUST00000153739	TC1582095	GO:0005737(cytoplasm)|GO:0007275	chr2:157387978-157388037	mm	2qH1
A_55_P2019214		Q91X50_MOUSE (Q91X50) Glu	119.18935	56.370625	P	P	-2.078653	0.4729502	TC1640169						TC1640169	GO:0004364(glutathione transferase	chr10:075259923-075259864	mm	10qC1
A_55_P1997110	Tmprss12	Mus musculus adult male testi	33.60348	2.410383	P	A	-2.079887	0.0717302	AK015583		AK015583	Mm.261400	75002		TC1593098	GO:0003824(catalytic activity)|GO:0	chr15:100115870-100115929	mm	15qF1
A_66_P136354	Lcp2	Mus musculus lymphocyte cyt	46.132345	15.984655	P	P	-2.081502	0.3464956	NM_010696	NM_010696	NM_010696	Mm.265350	16822	ENSMUST00000169878	TC1595244	GO:0005623(cell)|GO:0005737(cytop	chr11:33992133-33992192	mm	11qA4
A_55_P2037081	2610305D13Rik	Mus musculus RIKEN cDNA 26	366.8462	209.4741	P	P	-2.082502	0.5710134	NM_145078	NM_145078	NM_145078	Mm.327194	112422	ENSMUST00000037565	TC1578546	GO:0003674(molecular function)|GO:	chr4:146986112-146986053	mm	4qE1
A_55_P2078670	Cbs	Mus musculus cystathionine b	126.427	59.702105	P	P	-2.082906	0.4722259	NM_144855	NM_144855	NM_144855	Mm.206417	12411	ENSMUST00000078509	TC1589821	GO:0001958(endochondral ossificatio	chr17:31749652-31749593	mm	17qB1
A_55_P2179711			33.03415	2.3557475	P	A	-2.08457	0.0713125									chr11:097716305-097716876	mm	11qD
A_55_P1994284	Catsper4	Mus musculus cation channel	116.5757	54.996995	P	P	-2.085583	0.4717707	NM_177866	NM_177866	NM_177866	Mm.79072	329954	ENSMUST00000055892	TC1588008	GO:0001539(ciliary or flagellar motili	chr4:133767944-133767885	mm	4qD3
A_55_P2334058	4930401C15Rik	Mus musculus RIKEN cDNA 49	33.53833	2.384601	P	A	-2.086955	0.0711008	NR_045349	NR_045349	NR_045349	Mm.258921	73803				chr10:25499178-25499120	mm	10qA4
A_52_P345078	Wdr25	Mus musculus WD repeat dom	53.615325	18.511115	P	P	-2.088539	0.3452579	NM_177602	NM_177602	NM_177602	Mm.342082	212198	ENSMUST00000047115	TC1586985	GO:0003674(molecular function)|GO:	chr12:110266322-110266381	mm	12qF1
A_55_P2136348	Ccr8	Mus musculus chemokine (C-C	33.50282	2.3702625	P	A	-2.090942	0.0707481	NM_007720	NM_007720	NM_007720	Mm.442098	12776	ENSMUST00000048777	NP1489265	GO:0004871(signal transducer activi	chr9:120003293-120003352	mm	9qF4
A_51_P185693	Slc2a2	Mus musculus solute carrier fa	64.556535	22.184645	P	P	-2.097738	0.3436468	NM_031197	NM_031197	NM_031197	Mm.18443	20526	ENSMUST00000169047	TC1580021	GO:0005158(insulin receptor binding	chr3:28627016-28627075	mm	3qA3
A_55_P2000964	Akr1c21	Mus musculus aldo-keto reduc	1048.8169	596.3365	P	P	-2.098359	0.5685802	NM_029901	NM_029901	NM_029901	Mm.27085	77337	ENSMUST00000021628	TC1575431	GO:0004032(alditol:NADP+ 1-oxidor	chr13:4585696-4585755	mm	13qA1
A_55_P2073498		calcium homeostasis modulato	35.96126	2.511924	P	A	-2.101178	0.0698508	ENSMUST00000178630					ENSMUST00000178630			chr19:47226478-47226419	mm	19qC3
A_51_P409311	Bpifb6	Mus musculus BPI fold contain	37.61527	12.902445	P	P	-2.101381	0.3430108	NM_199303	NM_199303	NM_199303	Mm.137570	228796	ENSMUST00000088955	TC1590005	GO:0005575(cellular component)|GO	chr2:153738365-153738424	mm	2qH1
A_55_P2184831	Olfr728	Mus musculus olfactory recept	55.029605	18.873825	P	P	-2.101582	0.3429758	NM_001011809	NM_001011809	NM_001011809	Mm.377335	258039	ENSMUST00000051563	NP1398950	GO:0004871(signal transducer activi	chr14:50759436-50759377	mm	14qC1
A_52_P369123	Fam189a2	Mus musculus family with sequ	61.281965	21.01045	P	P	-2.10231	0.3428488	NM_001114174	NM_001114174	NM_001114174	Mm.426078	381217	ENSMUST00000096164	TC1690186	GO:0003674(molecular function)|GO:	chr19:24049950-24049891	mm	19qB
A_55_P2317864	C230021G24Rik	PREDICTED: Mus musculus RI	33.441145	2.330728	P	A	-2.102953	0.0696964	XR_105380	XR_105380	XR_105380	Mm.125555	321001		TC1597466	GO:0003674(molecular function)|GO:	chr10:105904119-105904060	mm	10qD1
A_66_P138210		RIKEN cDNA 4930512M02 gen	88.555525	30.29041	P	P	-2.106899	0.3420499	ENSMUST00000149910		AK131682	Mm.159430		ENSMUST00000149910	TC1591881		chr11:11527678-11527619	mm	11qA1
A_55_P1978696	Abcb4	Mus musculus adult male kidn	42.571545	14.550015	P	P	-2.108463	0.3417779	AK165484		AK165484	Mm.297825	18670		TC1607885	GO:0000139(Golgi membrane)|GO:00	chr5:8899682-8899741	mm	5qA1
A_55_P2032548	Slc4a5	Mus musculus solute carrier fa	36.638635	2.5320535	P	A	-2.109742	0.0691088	NM_001166067	NM_001166067	NM_001166067	Mm.333189	232156	ENSMUST00000039212	TC1621002	GO:0002064(epithelial cell developm	chr6:83249444-83249503	mm	6qC3
A_55_P2128238			38.604875	2.665659	P	A	-2.110427	0.0690498									chr17:018844198-018844256	mm	17qA3.2
A_52_P576150	Thap1	Mus musculus THAP domain c	40.030145	13.638245	P	P	-2.114679	0.3406994	NM_199042	NM_199042	NM_199042	Mm.383241	73754	ENSMUST00000036807	TC1582842	GO:0001935(endothelial cell prolifera	chr8:27271300-27271359	mm	8qA2
A_55_P2173952	Myh6	Mus musculus myosin, heavy c	36.54852	12.44396	P	A	-2.115959	0.3404778	NM_001164171	NM_001164171	NM_001164171	Mm.290003	17888	ENSMUST00000081857	TC1577524	GO:0000146(microfilament motor act	chr14:55560833-55560774	mm	14qC3
A_51_P126626	Zfp503	Mus musculus zinc finger prote	661.72326	373.80561	P	P	-2.122492	0.5648972	NM_145459	NM_145459	NM_145459	Mm.292401	218820	ENSMUST00000043409	TC1575977	GO:0003676(nucleic acid binding)|GO	chr14:22803318-22803259	mm	14qA3
A_55_P2262272	4930443O20Rik	Mus musculus RIKEN cDNA 49	27.584685	9.357982	P	A	-2.12309	0.3392456	NR_040504	NR_040504	NR_040504	Mm.66996	74687	ENSMUST00000135659	TC1610134		chr2:84913833-84913774	mm	2qD
A_55_P2002572	Ephx2	Mus musculus epoxide hydrola	2144.8755	1321.553	P	P	-2.123153	0.6161444	NM_001271402	NM_001271402	NM_001271402	Mm.15295	13850	ENSMUST00000070515	TC1575768	GO:0000287(magnesium ion binding)|	chr14:66703273-66703214	mm	14qD1
A_55_P2196417		Mus musculus 16 days neonat	50.92679	3.4469925	P	A	-2.126434	0.0676852	AK035992		AK035992	Mm.447152			TC1631023		chr17:86577310-86577369	mm	17qE4
A_55_P2022444	Pdss2	Mus musculus prenyl (solanesy	95.476045	44.342035	P	P	-2.129095	0.464431	NM_027772	NM_027772	NM_027772	Mm.490430	71365	ENSMUST00000162008	NP1489309	GO:0000010(trans-hexaprenyltranst	chr10:43159704-43159763	mm	10qB2
A_52_P531731	Grin3a	Mus musculus glutamate recep	36.577405	2.462643	P	A	-2.130691	0.0673269	NM_001276355	NM_001276355	NM_001276355	Mm.440095	242443	ENSMUST00000093859	TC1716671	GO:0004970(ionotropic glutamate re	chr4:49674747-49674688	mm	4qB1
A_51_P335993	Plac8l1	Mus musculus PLAC8-like 1 (F	35.232795	2.3606708	P	A	-2.134569	0.0670021	NM_027072	NM_027072	NM_027072	Mm.34841	69401	ENSMUST00000081374	TC1589659	GO:0003674(molecular function)|GO:	chr18:42338529-42338470	mm	18qB3
A_51_P458451	Adipoq	Mus musculus adiponectin, C1	17360.815	10527.616	P	P	-2.134701	0.606401	NM_009605	NM_009605	NM_009605	Mm.3969	11450	ENSMUST00000023593	TC1578928	GO:0001934(positive regulation of pr	chr16:23157576-23157635	mm	16qB1
A_55_P1964332			981.27605	552.0436	P	P	-2.137774	0.5625773									chr17:089675670-089675729	mm	17qE5
A_55_P2397090	4930480K15Rik	Mus musculus RIKEN cDNA 49	35.359165	2.3587018	P	A	-2.138109	0.0667069	NR_045463	NR_045463	NR_045463	Mm.410772	78800	ENSMUST00000162121	TC1608515		chr17:91266316-91266375	mm	17qE5
A_52_P426503	Abhd14a	Mus musculus abhydrolase dor	101.47134	34.155675	P	P	-2.138462	0.3366042	NM_145919	NM_145919	NM_145919	Mm.268925	68644	ENSMUST00000171678	TC1641947	GO:0003674(molecular function)|GO:	chr9:106346520-106346461	mm	9qF1
A_55_P2177741	Gm7166	PREDICTED: Mus musculus pr	25.98617	8.733616	P	A	-2.141486	0.3360871	XM_910904	XM_910904	XM_910904		635756			GO:0003674(molecular function)|GO:	chr3:90843719-90843778	mm	3qF1
A_55_P2052380	Gbp6	Mus musculus guanylate bindir	785.36235	441.3285	P	P	-2.141966	0.5619425	NM_194336	NM_194336	NM_194336	Mm.491210	100702	ENSMUST00000050011	TC1577091	GO:0003674(molecular function)|GO:	chr5:105700115-105700056	mm	5qE5
A_51_P437135	Dab1	Mus musculus disabled 1 (Dab	36.49612	2.414953	P	A	-2.144589	0.0661701	NM_010014	NM_010014	NM_010014	Mm.289682	13131	ENSMUST00000106826	NP358233	GO:0001764(neuron migration)|GO:0	chr4:104352164-104352720	mm	4qC6
A_55_P2161923	Rabgap1	Mus musculus RAB GTPase ac	1415.436	793.67535	P	P	-2.149997	0.5607285	NM_001033960	NM_001033960	NM_001033960	Mm.383192	227800	ENSMUST00000130601	TC1584107	GO:0003674(molecular function)|GO:	chr2:37351140-37351199	mm	2qB
A_55_P2072915			31.176497	10.419961	P	A	-2.152414	0.3342249									chrX:154587994-154588053	mm	XqF4
A_55_P1989951			36.37179	2.3760785	P	A	-2.154866	0.0653275									chr10:093094256-093094315	mm	10qC2
A_52_P229972	Slc22a1	Mus musculus solute carrier fa	294.7026	135.57065	P	P	-2.155545	0.4600253	NM_009202	NM_009202	NM_009202	Mm.594	20517	ENSMUST00000024596	TC1577730	GO:0005215(transporter activity)|GC	chr17:12845321-12845262	mm	17qA1
A_55_P2318934	1200007C13Rik	Mus musculus adult male lung	35.391065	11.76645	P	A	-2.16277	0.3324695	AK004627		AK004627	Mm.410869	74119		NP749416		chr2:167653261-167653202	mm	2qH3
A_55_P1989362			37.290765	2.3886765	P	A	-2.170635	0.0640554	NAP070114-1								chr2:148757323-148757264	mm	2qG3
A_51_P171936	A630033H20Rik	Mus musculus RIKEN cDNA A6	57.432125	18.94921	P	P	-2.177785	0.329941	NM_175442	NM_175442	NM_175442	Mm.265244	213438	ENSMUST00000067249	TC1589795	GO:0003674(molecular function)|GO:	chrX:104367504-104367563	mm	XqD
A_51_P470715	Cish	Mus musculus cytokine inducit	1197.6665	666.3355	P	P	-2.179032	0.5563615	NM_009895	NM_009895	NM_009895	Mm.4592	12700	ENSMUST00000165664	TC1575404	GO:0005515(protein binding)|GO:000	chr9:107204196-107204255	mm	9qF1
A_55_P2327853	4931431B13Rik	Mus musculus adult male testi	147.36365	67.198505	P	P	-2.179906	0.4560046	AK016494		AK016494	Mm.441564	70973				chr7:135345676-135345617	mm	7qF3
A_52_P52910	Fam151a	Mus musculus family with sequ	40.877895	13.45556	P	P	-2.182417	0.3291647	NM_146149	NM_146149	NM_146149	Mm.476048	230579	ENSMUST00000047620	TC1580087	GO:0003674(molecular function)|GO:	chr4:106420510-106420569	mm	4qC7
A_52_P40293	Hgd	Mus musculus homogentisate	33.511935	11.026855	P	P	-2.183147	0.3290426	NM_013547	NM_013547	NM_013547	Mm.157442	15233	ENSMUST00000160847	TC1598675	GO:0004411(homogentisate 1,2-diox	chr16:37621972-37622031	mm	16qB3
A_55_P2161242			37.06118	2.3268065	P	A	-2.186728	0.0627828									chr16:015083968-015083909	mm	16qA1
A_55_P1999862	Rad51ap2	Mus musculus RAD51 associat	40.80174	2.5392765	P	A	-2.193762	0.0622345	NM_001111118	NM_001111118	NM_001111118	Mm.341257	209550	ENSMUST00000124065	TC1622685	GO:0003674(molecular function)|GO:	chr12:11469547-11469606	mm	12qA1.1
A_51_P462862	9430015G10Rik	Mus musculus RIKEN cDNA 94	29.62794	9.679286	P	A	-2.197232	0.3266945	NM_145557	NM_145557	NM_145557	Mm.318925	230996	ENSMUST00000169550	TC1610184	GO:0003674(molecular function)|GO:	chr4:155500803-155500862	mm	4qE2
A_55_P2033215	Cabp5	Mus musculus calcium binding	10196.145	6091.488	P	P	-2.197804	0.5974305	NM_013877	NM_013877	NM_013877	Mm.103669	29865	ENSMUST00000005791	TC1588736	GO:0005509(calcium ion binding)|GO	chr7:13993821-13993880	mm	7qA1
A_55_P2183055	E4f1	Mus musculus E4F transcriptic	2611.8555	1582.189	P	P	-2.198185	0.605772	NM_007893	NM_007893	NM_007893	Mm.163132	13560	ENSMUST00000056032	NP712417	GO:0000122(negative regulation of tr	chr17:24580800-24580741	mm	17qA3.3
A_55_P2106358			43.67786	14.254715	P	P	-2.199246	0.3263602									chr6:071440095-071440036	mm	6qC1
A_55_P1952314	Olfr1415	Mus musculus olfactory recept	44.35545	2.7311385	P	A	-2.20232	0.0615739	NM_001011525	NM_001011525	NM_001011525	Mm.377386	258228	ENSMUST00000071521	TC1711489	GO:0004871(signal transducer activi	chr1:94387454-94387395	mm	1qD
A_52_P10037	Asf1a	Mus musculus ASF1 anti-silen	34.666515	11.259945	P	P	-2.208625	0.3248075	NM_025541	NM_025541	NM_025541	Mm.272989	66403	ENSMUST00000020004	TC1577919	GO:0001649(osteoblast differentiatio	chr10:53328392-53328451	mm	10qB3
A_55_P2111083	Tas2r126	Mus musculus taste receptor	39.10122	2.3879225	P	A	-2.208906	0.0610703	NM_207028	NM_207028	NM_207028	Mm.377914	387353	ENSMUST00000059534	NP955578	GO:0001580(detection of chemical s	chr6:42385354-42385413	mm	6qB2.1
A_55_P2121760			375.86975	169.54825	P	P	-2.210022	0.4510825									chr11:112241546-112241487	mm	11qE2
A_55_P2118857		RIKEN cDNA 4930423O20 gen	1873.9125	1131.9765	P	P	-2.210611	0.6040712	ENSMUST00000072455					ENSMUST00000072455			chr16:70382352-70382293	mm	16qC2
A_55_P2148402	Cplx2	Mus musculus complexin 2 (Cp	3923.5165	2369.651	P	P	-2.211417	0.603961	NM_009946	NM_009946	NM_009946	Mm.268902	12890	ENSMUST00000026985	TC1587818	GO:0000149(SNARE binding)|GO:000	chr13:54481316-54481375	mm	13qB1
A_55_P2126815			39.988375	2.411071	P	A	-2.219161	0.0602943									chr14:104022576-104022517	mm	14qE2.3
A_55_P2025468	Tst	Mus musculus thiosulfate sulfu	932.5827	513.0522	P	P	-2.220783	0.5501412	NM_009437	NM_009437	NM_009437	Mm.15312	22117	ENSMUST00000058659	TC1575910	GO:0003723(RNA binding)|GO:00047	chr15:78230271-78230212	mm	15qE1
A_55_P2151388	Sostdc1	Mus musculus sclerostin doma	45.5382	14.693688	P	P	-2.221627	0.3226673	NM_025312	NM_025312	NM_025312	Mm.43375	66042	ENSMUST00000041407	TC1579772	GO:0005515(protein binding)|GO:000	chr12:37044197-37044256	mm	12qA3
A_55_P2321185	4930488B22Rik	Mus musculus RIKEN cDNA 49	39.592575	2.3565405	P	A	-2.229529	0.0595198	NR_040627	NR_040627	NR_040627	Mm.410816	74971	ENSMUST00000172500	TC1726966		chr1:186542809-186542868	mm	1qH5
A_55_P2123012	AA667203	PREDICTED: Mus musculus ex	162.1083	72.433395	P	P	-2.236363	0.446821	XR_141351	XR_141351	XR_141351	Mm.482595	100503467				chr18:65055717-65055776	mm	18qE1
A_55_P2065754	Krtap19-5	Mus musculus keratin associat	1148.798	628.8164	P	P	-2.239543	0.547369	NM_010676	NM_010676	NM_010676	Mm.3503	16704	ENSMUST00000053460	TC1595960	GO:0003674(molecular function)|GO:	chr16:88896284-88896225	mm	16qC3.3
A_55_P2011111	Cyp4a10	Mus musculus cytochrome P45	59.54782	19.02962	P	P	-2.240604	0.3195687	NM_010011	NM_010011	NM_010011	Mm.10742	13117	ENSMUST00000094886	TC1579925	GO:0004497(monooxygenase activity	chr4:115206150-115206209	mm	4qD1
A_51_P472274	Sox18	Mus musculus SRY-box conta	396.163	216.4893	P	P	-2.24568	0.5464652	NM_009236	NM_009236	NM_009236	Mm.264904	20672	ENSMUST00000054491	TC1581181	GO:0000122(negative regulation of tr	chr2:181404848-181404789	mm	2qH4
A_55_P2257306	4930402D18Rik	Mus musculus adult male testi	42.296005	2.465646	P	A	-2.246203	0.058295	AK015045		AK015045	Mm.159336	74852		TC1649531		chr15:60798840-60798899	mm	15qD1
A_51_P391727	Slc3a1	Mus musculus solute carrier fa	109.24705	48.458395	P	P	-2.256646	0.4435671	NM_009205	NM_009205	NM_009205	Mm.227176	20532	ENSMUST00000024944	TC1633886	GO:0005743(mitochondrial inner mer	chr17:85463142-85463201	mm	17qE4
A_55_P2112459	Cldn22	Mus musculus claudin 22 (Cldn	384.72095	208.79825	P	P	-2.271174	0.5427265	NM_029383	NM_029383	NM_029383	Mm.390033	75677	ENSMUST00000038693		GO:0003674(molecular function)|GO:	chr8:48910761-48910820	mm	8qB1.1
A_55_P2398788	Rnf112	Mus musculus ring finger prote	78.19419	24.53783	P	P	-2.276391	0.3138063	NM_009548	NM_009548	NM_009548	Mm.89201	22671	ENSMUST00000060255	TC1580331	GO:0003674(molecular function)|GO:	chr11:61264390-61264331	mm	11qB2
A_66_P126104	4933407E24Rik	Mus musculus RIKEN cDNA 49	42.076485	2.352485	P	A	-2.279706	0.0559097	NR_045819	NR_045819	NR_045819	Mm.250313	108809		TC1739163		chr4:124252516-124252575	mm	4qD2.2
A_51_P176086	Ffar2	Mus musculus free fatty acid r	122.8052	53.990625	P	P	-2.281295	0.4396445	NM_146187	NM_146187	NM_146187	Mm.97338	233079	ENSMUST00000053156	TC1584889	GO:0004871(signal transducer activi	chr7:31604208-31604149	mm	7qB1
A_51_P167292	Chi3l3	Mus musculus chitinase 3-like	50.34615	15.737635	P	P	-2.284037	0.3125886	NM_009892	NM_009892	NM_009892	Mm.387173	12655	ENSMUST00000063062	TC1581278	GO:0004553(hydrolase activity, hydr	chr3:105950544-105950485	mm	3qF2.2
A_55_P2111212	9130209A04Rik	Mus musculus RIKEN cDNA 91	43.91102	2.4385405	P	A	-2.285118	0.0555337	NR_033453	NR_033453	NR_033453	Mm.98433	77668		TC1614970		chr18:47630411-47630470	mm	18qC
A_55_P2382741	AI314604	vd44a05.r1 Barstead MPLRB1	193.11015	84.67999	P	P	-2.288489	0.4385062	AA473072		AA473072	Mm.324125	102027		TC1680442		chr8:48181864-48181923	mm	8qB1.1
A_52_P71624	Olfr1410	Mus musculus olfactory recept	29.941455	9.33328	P	A	-2.289525	0.3117177	NM_146491	NM_146491	NM_146491	Mm.377528	258484	ENSMUST00000079790	NP830808	GO:0004871(signal transducer activi	chr1:94505050-94505109	mm	1qD
A_55_P2027083	Kcnj10	Mus musculus potassium inwai	628.6442	339.28235	P	P	-2.291906	0.5397049	NM_001039484	NM_001039484	NM_001039484	Mm.254563	16513	ENSMUST00000056136	TC1598226	GO:0000166(nucleotide binding)|GO:0	chr1:174304062-174304121	mm	1qH3
A_55_P2142668			216.1368	94.48597	P	P	-2.297033	0.4371582									chr14:102293160-102293101	mm	14qE2.3
A_55_P2167585	Ube2cbp	ubiquitin-conjugating enzyme	44.5491	2.433257	P	A	-2.298426	0.0546197	ENSMUST00000121189				70348	ENSMUST00000121189		GO:0003674(molecular function)|GO:	chr9:86202478-86202419	mm	9qE3.1
A_55_P2011031	Cdk5rap1	Mus musculus CDK5 regulator	77.568645	24.042585	P	P	-2.300694	0.3099524	NM_025876	NM_025876	NM_025876	Mm.289427	66971	ENSMUST00000148289	TC1665648	GO:0000079(regulation of cyclin-dep	chr2:154191758-154191699	mm	2qH1
A_55_P2004551	Klra1	Mus musculus killer cell lectin-	1546.4935	832.5229	P	P	-2.301382	0.5383294	NM_016659	NM_016659	NM_016659	Mm.24399	16627	ENSMUST00000032288	TC1586502	GO:0004871(signal transducer activi	chr6:130314129-130314070	mm	6qF3
A_55_P2005720			784.09775	421.6456	P	P	-2.305407	0.5377462									chr14:071974463-071974404	mm	14qD2
A_55_P2280868		RIKEN cDNA 0610005C13 gen	49.07122	15.165115	P	P	-2.306473	0.309043	ENSMUST00000142260		AK085337	Mm.439894		ENSMUST00000142260	TC1604629		chr7:52824667-52824608	mm	7qB4
A_55_P1964009	Zfp217	Mus musculus zinc finger prote	123.59595	53.71266	P	P	-2.31343	0.4345827	NM_001033299	NM_001033299	NM_001033299	Mm.22858	228913	ENSMUST00000063710	TC1584840	GO:0000118(histone deacetylase co	chr2:169935647-169935588	mm	2qH3
A_55_P2341890		Mus musculus 16 days neonat	32.3391	9.9512425	P	A	-2.314939	0.3077155	AK137931		AK137931						chr3:79056191-79056132	mm	3qE3
A_51_P500882	Ncr1	Mus musculus natural cytotoxi	44.373965	2.368282	P	A	-2.316973	0.053371	NM_010746	NM_010746	NM_010746	Mm.240231	17086	ENSMUST00000126417	TC1586822	GO:0005886(plasma membrane)|GO:	chr7:4296176-4296235	mm	7qA1
A_51_P198675	Ttc36	Mus musculus tetratricopeptid	62.888905	19.31853	P	P	-2.318333	0.307185	NM_138951	NM_138951	NM_138951	Mm.325487	192653	ENSMUST00000044694	TC1579287	GO:0003674(molecular function)|GO:	chr9:44610918-44610859	mm	9qA5.2
A_55_P2084332	Pigp	Mus musculus phosphatidylino	62.197445	19.054545	P	P	-2.323649	0.3063558	NM_001159620	NM_001159620	NM_001159620	Mm.474449	56176	ENSMUST00000071991	TC1595073	GO:0000506(glycosylphosphatidylino	chr16:94580704-94580645	mm	16qC4
A_55_P2014274			160.8953	69.49445	P	P	-2.330463	0.4319234									chr19:035673846-035673787	mm	19qC2
A_55_P2127243	Tpsb2	Mus musculus tryptase beta 2	678.46	361.51527	P	P	-2.339396	0.5328468	NM_010781	NM_010781	NM_010781	Mm.7409	17229	ENSMUST00000069616	TC1585442	GO:0003824(catalytic activity)|GO:0	chr17:25504819-25504878	mm	17qA3.3
A_55_P2020361	Lzts3	Mus musculus leucine zipper, p	8412.975	4857.336	P	P	-2.342473	0.5773625	NM_197945	NM_197945	NM_197945	Mm.440130	241638	ENSMUST00000045761	TC1643921	GO:0005737(cytoplasm)|GO:0005856	chr2:130459382-130459323	mm	2qF1
A_55_P2044627	Rnase10	Mus musculus ribonuclease, RI	462.96005	246.3386	P	P	-2.344641	0.5320947	NM_029145	NM_029145	NM_029145	Mm.318775	75019	ENSMUST00000022424		GO:0003674(molecular function)|GO:	chr14:51630373-51630432	mm	14qC1
A_55_P2155216	Ankrd34a	Mus musculus ankyrin repeat c	29.51821	8.936503	P	A	-2.346964	0.3027454	NM_001024851	NM_001024851	NM_001024851	Mm.267664	545554	ENSMUST00000058943	TC1585347	GO:0003674(molecular function)|GO:	chr3:96403642-96403701	mm	3qF2.1
A_51_P430082	Tst	Mus musculus thiosulfate sulfu	1411.4235	750.36205	P	P	-2.347851	0.5316349	NM_009437	NM_009437	NM_009437	Mm.15312	22117	ENSMUST00000058659	TC1744660	GO:0003723(RNA binding)|GO:00047	chr15:78230117-78230058	mm	15qE1
A_51_P318856	Glyat	Mus musculus glycine-N-acylt	94.24313	28.50574	P	P	-2.348753	0.3024702	NM_145935	NM_145935	NM_145935	Mm.39974	107146	ENSMUST00000044976	TC1579726	GO:0005739(mitochondrion)|GO:0008	chr19:12724890-12725749	mm	19qA
A_55_P2021476		Q6TXJ2_RAT (Q6TXJ2) LRRG	10467.37	6022.6765	P	P	-2.357063	0.5753763	TC1636493						TC1636493	GO:0000786(nucleosome)|GO:00036	chr14:041958055-041958114	mm	14qB
A_52_P624599		RIKEN cDNA 4930451E10 gen	46.593125	2.364819	P	A	-2.35728	0.0507547	ENSMUST00000152292		AK019623	Mm.243826		ENSMUST00000152292	TC1596769		chr13:55877412-55877353	mm	13qB1
A_51_P369784	Ces1e	Mus musculus carboxylesteras	112.3617	48.005885	P	P	-2.36069	0.4272442	NM_133660	NM_133660	NM_133660	Mm.63490	13897	ENSMUST00000034173	TC1583299	GO:0005783(endoplasmic reticulum)|	chr8:95725266-95725207	mm	8qC5
A_55_P2007170	Slc5a2	Mus musculus solute carrier fa	1946.231	1134.4565	P	P	-2.368287	0.5828992	NM_133254	NM_133254	NM_133254	Mm.38870	246787	ENSMUST00000118169	TC1709307	GO:0005215(transporter activity)|GC	chr7:135415884-135415943	mm	7qF3
A_52_P187909	Slco1a6	Mus musculus solute carrier or	113.4754	33.939575	P	P	-2.370842	0.2990919	NM_023718	NM_023718	NM_023718	Mm.38953	28254	ENSMUST00000111827	TC1583013	GO:0003674(molecular function)|GO:	chr6:142034766-142034707	mm	6qG2
A_55_P2146185	Rs1	Mus musculus retinoschisis (X	13638.755	7816.06	P	P	-2.374016	0.5730772	NM_011302	NM_011302	NM_011302	Mm.41982	20147	ENSMUST00000150170	TC1584989	GO:0001786(phosphatidylserine bind	chrX:157232740-157232799	mm	XqF4
A_55_P1972973			64.22081	19.1298	P	P	-2.378858	0.2978754									chrY_random:053407004-053406945		
A_55_P2121456	Fam43a	Mus musculus family with sequ	485.8359	256.05875	P	P	-2.380032	0.5270478	NM_177632	NM_177632	NM_177632	Mm.28244	224093	ENSMUST00000059078	TC1576754	GO:0003674(molecular function)|GO:	chr16:30602655-30602714	mm	16qB2
A_55_P1987847			154.6258	65.55349	P	P	-2.382174	0.4239492									chr2:029906910-029906969	mm	2qB
A_55_P2165929	Olfr3	Mus musculus olfactory recept	48.954805	2.401874	P	A	-2.384463	0.0490631	NM_206903	NM_206903	NM_206903	Mm.377104	18328	ENSMUST00000100151	TC1696786	GO:0004871(signal transducer activi	chr2:36667822-36667763	mm	2qB
A_55_P1966383			980.40275	516.09385	P	P	-2.384528	0.52641									chr5:045701575-045701516	mm	5qB3
A_55_P2004536	Klra4	Mus musculus killer cell lectin-	38.70391	11.47814	P	A	-2.387544	0.2965628	NM_010649	NM_010649	NM_010649	Mm.333431	16635	ENSMUST00000119096	TC1594090	GO:0007155(cell adhesion)|GO:0009E	chr6:129994094-129994035	mm	6qF3
A_55_P2377156			48.59077	2.3651985	P	A	-2.390817	0.0486759	TC1674879						TC1674879		chr10:030519394-030519335	mm	10qA4
A_55_P1977792	Folr1	Mus musculus folate receptor	2584.5065	1498.186	P	P	-2.392765	0.5796797	NM_001252552	NM_001252552	NM_001252552	Mm.2135	14275	ENSMUST00000001882	TC1577359	GO:0004872(receptor activity)|GO:00	chr7:109006905-109006846	mm	7qE3
A_51_P224164	Slc26a4	Mus musculus solute carrier fa	308.6244	130.30145	P	P	-2.393643	0.4222007	NM_011867	NM_011867	NM_011867	Mm.100187	23985	ENSMUST00000001253	TC1585767	GO:0005886(plasma membrane)|GO:	chr12:32204825-32204766	mm	12qA3
A_55_P1968763	Shc4	Mus musculus SHC (Src homo	7704.3905	4393.6255	P	P	-2.394766	0.5702755	NM_199022	NM_199022	NM_199022	Mm.425101	271849	ENSMUST00000110480	TC1602549	GO:0005575(cellular component)|GO	chr2:125453990-125453931	mm	2qF1
A_55_P1960738	Gm4470	602334681F2 NCI_CGAP_Mam	1415.6535	742.8238	P	P	-2.396459	0.5247215	BG175686		BG175686	Mm.410724	100043484				chr19:56156181-56156239	mm	19qD2
A_55_P2370250	Syn3	Mus musculus 16 days neonat	384.0001	201.33255	P	P	-2.399419	0.5243034	AK036325		AK036325	Mm.444790	27204			GO:0000166(nucleotide binding)|GO:0	chr10:85586627-85586568	mm	10qC1
A_66_P134704	Gm4952	Mus musculus predicted gene	33.019675	9.715663	P	A	-2.403018	0.2942386	NM_001013762	NM_001013762	NM_001013762	Mm.302458	240549	ENSMUST00000092931	TC1589512	GO:0003674(molecular function)|GO:	chr19:12702020-12702079	mm	19qA
A_51_P263302	Rnf24	Mus musculus ring finger prote	113.68915	47.783235	P	P	-2.406183	0.4202972	NM_178607	NM_178607	NM_178607	Mm.477523	51902	ENSMUST00000010194		GO:0003674(molecular function)|GO:	chr2:131124480-131124421	mm	2qF1
A_55_P2358256	Nr1i3	Mus musculus nuclear recepto	150.21805	62.985785	P	P	-2.412803	0.4192957	NM_009803	NM_009803	NM_009803	Mm.486506	12355	ENSMUST00000149404	TC1593157	GO:0003677(DNA binding)|GO:00037	chr1:173147776-173147835	mm	1qH3
A_55_P1975927			51.11188	2.398887	P	A	-2.420039	0.046934									chr2:119303803-119303744	mm	2qE5
A_55_P2335648	D630045M09Rik	Mus musculus RIKEN cDNA D6	43.064885	12.544	P	A	-2.422884	0.2912814	NR_045293	NR_045293	NR_045293	Mm.415319	432777		TC1619023		chr13:73482340-73482281	mm	13qC1
A_55_P2280237	Zfp697	Mus musculus zinc finger prote	95.769965	27.86724	P	P	-2.424913	0.290981	NM_172863	NM_172863	NM_172863	Mm.358911	242109	ENSMUST00000178372	TC1590850	GO:0003676(nucleic acid binding)|GO	chr3:98233867-98233926	mm	3qF2.2
A_55_P2057112	Prl8a2	Mus musculus prolactin family	51.123675	2.3787855	P	A	-2.426973	0.04653	NM_010088	NM_010088	NM_010088	Mm.291450	13529	ENSMUST00000018403	TC1576306	GO:0001666(response to hypoxia)|GO	chr13:27445965-27446024	mm	13qA3.1
A_55_P1968085			421.9813	175.686	P	P	-2.432461	0.416336											
A_55_P1999301	Cyp2e1	Mus musculus cytochrome P45	17437.47	9828.3615	P	P	-2.444362	0.5636346	NM_021282	NM_021282	NM_021282	Mm.21758	13106	ENSMUST00000026552	TC1715724	GO:0000139(Golgi membrane)|GO:00	chr7:147960628-147960687	mm	7qF5
A_51_P209327	Apln	Mus musculus apelin (Apln), ml	325.73245	134.95975	P	P	-2.445884	0.414327	NM_013912	NM_013912	NM_013912	Mm.29262	30878	ENSMUST00000039026		GO:0002026(regulation of the force c	chrX:45378652-45378593	mm	XqA4
A_55_P2017769	B020031M17Rik	Mus musculus RIKEN cDNA B0	288.2378	119.39975	P	P	-2.446464	0.4142404	NM_001033769	NM_001033769	NM_001033769	Mm.387257	333467	ENSMUST00000180768	TC1596142	GO:0003674(molecular function)|GO:	chrUn_random:2889834-2889775		
A_55_P2004132	Gm853	Mus musculus predicted gene	39.22333	11.27616	P	A	-2.448678	0.287486	NM_001034872	NM_001034872	NM_001034872	Mm.387701	332942	ENSMUST00000023884	TC1590687	GO:0003674(molecular function)|GO:	chr4:129886459-129886400	mm	4qD2.2
A_55_P1997184			53.60751	2.4061435	P	A	-2.455847	0.0448844									chr13:081100031-081099972	mm	13qC3
A_55_P2173039		predicted gene 6802 [Source:M	47.310625	13.53342	P	P	-2.458496	0.2860546	ENSMUST00000167843	XR_168483	XR_168483			ENSMUST00000167843			chr12:19497973-19498032	mm	12qA1.2
A_51_P130727	Fkbp11	Mus musculus FK506 binding p	43.91956	12.512025	P	A	-2.466553	0.284885	NM_024169	NM_024169	NM_024169	Mm.30729	66120	ENSMUST00000003445	TC1596505	GO:0000413(protein peptidyl-prolyl i	chr15:98554868-98554809	mm	15qF1
A_55_P2106429	Herc2	Mus musculus hect (homologou	4170.217	2376.7485	P	P	-2.467698	0.569934	NM_010418	NM_010418	NM_010418	Mm.20929	15204	ENSMUST00000164095	TC1574713	GO:0004842(ubiquitin-protein ligase	chr7:63486603-63486662	mm	7qB5
A_55_P2108275			192.92195	78.979055	P	P	-2.479193	0.4093835									chr17:073802434-073802375	mm	17qE2
A_51_P291749	Pecr	Mus musculus peroxisomal tra	1497.7515	767.39525	P	P	-2.484953	0.5123649	NM_023523	NM_023523	NM_023523	Mm.281738	111175	ENSMUST00000027381	TC1576943	GO:0005102(receptor binding)|GO:00	chr1:72305898-72305839	mm	1qC3
A_55_P2118609	St6galnac1	Mus musculus ST6 (alpha-N-a	3186.7065	1809.045	P	P	-2.485173	0.5676848	NM_011371	NM_011371	NM_011371	Mm.436887	20445	ENSMUST00000009732	TC1592196	GO:0001574(ganglioside biosynthetic	chr11:116626625-116626566	mm	11qE2
A_55_P2117894	Tas2r130	Mus musculus taste receptor,	54.28364	2.348349	P	A	-2.485395	0.0432607	NM_199156	NM_199156	NM_199156	Mm.377916	387355	ENSMUST00000067597	NP920320	GO:0001580(detection of chemical s	chr6:131579976-131579917	mm	6qF3
A_55_P2318584	Aqp8	Mus musculus aquaporin 8 (Aq	28.54488	8.052461	P	A	-2.485886	0.2820983	NM_007474	NM_007474	NM_007474	Mm.273175	11833	ENSMUST00000098056		GO:0005215(transporter activity)|GC	chr7:130611453-130611512	mm	7qF3
A_55_P2046504		RIKEN cDNA 1700055D18 gen	32.84879	9.265724	P	A	-2.486069	0.282072	ENSMUST00000095105	XR_035212	XR_035212	Mm.82696		ENSMUST00000095105	TC1589883		chr4:45027087-45027146	mm	4qB1
A_55_P2066976	Acsm1	Mus musculus acyl-CoA synth	1185.8845	607.2551	P	P	-2.487096	0.5120693	NM_054094	NM_054094	NM_054094	Mm.427560	117147	ENSMUST00000047929		GO:0000166(nucleotide binding)|GO:0	chr7:126805969-126806028	mm	7qF2
A_55_P2129407		RIKEN cDNA 4932431P20 gen	194.82135	79.497985	P	P	-2.488207	0.4080558	ENSMUST00000141713					ENSMUST00000141713			chr7:30317242-30317301	mm	7qB1
A_55_P2047620			9187.6675	5122.944	P	P	-2.490021	0.5575892	NAP072242-1								chr10:038991620-038991679	mm	10qB1
A_55_P2095325	AI182371	Mus musculus expressed sequ	57.059565	2.3756695	P	A	-2.516114	0.0416349	NM_178885	NM_178885	NM_178885	Mm.415380	98870	ENSMUST00000045776	TC1596208	GO:0003674(molecular function)|GO:	chr2:34939932-34939873	mm	2qB
A_55_P2014326	Gm8457	PREDICTED: Mus musculus pr	348.63355	140.69845	P	P	-2.518874	0.4035712	XM_003085120	XM_003085120	XM_003085120		667102				chr17:8094983-8094971	mm	17qA1
A_55_P2053923	Rab17	Mus musculus RAB17, member	102.29365	41.259575	P	P	-2.520433	0.4033444	NM_008998	NM_008998	NM_008998	Mm.279780	19329	ENSMUST00000131428	NP355944	GO:0000166(nucleotide binding)|GO:0	chr1:92854959-92854900	mm	1qD
A_51_P178772	Ces1f	Mus musculus carboxylesteras	5006.515	2818.348	P	P	-2.522298	0.5629361	NM_144930	NM_144930	NM_144930	Mm.29110	234564	ENSMUST00000034178	TC1578464	GO:0005811(lipid particle)|GO:00058	chr8:95780284-95780225	mm	8qC5
A_55_P2361927	4930480G23Rik	Mus musculus RIKEN cDNA 49	57.876315	2.3782745	P	A	-2.526632	0.0410924	NR_040768	NR_040768	NR_040768	Mm.445292	74988	ENSMUST00000125909	TC1600640		chr4:19906651-19906592	mm	4qA3
A_52_P973575	Hoxb9	Mus musculus homeobox B9 (H	75.68886	20.815535	P	P	-2.535903	0.2750145	NM_008270	NM_008270	NM_008270	Mm.258271	15417	ENSMUST00000000010	TC1687897	GO:0003677(DNA binding)|GO:00037	chr11:96137748-96137807	mm	11qD
A_51_P358574	Pdzd3	Mus musculus PDZ domain co	37.700765	10.367804	P	A	-2.535989	0.2750025	NM_133226	NM_133226	NM_133226	Mm.29872	170761	ENSMUST00000034618	TC1583795	GO:0005515(protein binding)|GO:000	chr9:44055701-44055642	mm	9qA5.2
A_51_P326229	Ddx25	Mus musculus DEAD (Asp-Glu	2561.4055	1437.2105	P	P	-2.536718	0.5611023	NM_013932	NM_013932	NM_013932	Mm.291723	30959	ENSMUST00000034612	TC1579436	GO:0000166(nucleotide binding)|GO:0	chr9:35349594-35349535	mm	9qA4
A_55_P2179676	Spp2	Mus musculus secreted phosp	3843.51	2153.0405	P	P	-2.544023	0.5601756	NM_029269	NM_029269	NM_029269	Mm.173058	75396	ENSMUST00000027518	TC1580745	GO:0003674(molecular function)|GO:	chr1:90322944-90323003	mm	1qD
A_55_P2131766	D4Wsu53e	Mus musculus DNA segment, (	5244.881	2937.322	P	P	-2.545125	0.560036	NM_023665	NM_023665	NM_023665	Mm.331964	27981	ENSMUST00000145364	TC1643383	GO:0003674(molecular function)|GO:	chr4:134483142-134483201	mm	4qD3
A_52_P285470	Lrp2	Mus musculus low density lipo	337.0052	134.69155	P	P	-2.545815	0.399672	NM_001081088	NM_001081088	NM_001081088	Mm.23847	14725	ENSMUST00000080953	TC1608935	GO:0004872(receptor activity)|GO:00	chr2:69262625-69262566	mm	2qC2
A_55_P2177336	Lipi	Mus musculus lipase, member	60.86372	2.4061835	P	A	-2.557637	0.039534	NM_001252513	NM_001252513	NM_001252513	Mm.99517	320355	ENSMUST00000062721	TC1591250	GO:0004620(phospholipase activity)|	chr16:75540848-75540789	mm	16qC3.1
A_55_P2183715			32.81977	8.880446	P	A	-2.567858	0.2705822									chr3:014377530-014377589	mm	3qA1
A_51_P392967	Zmynd10	Mus musculus zinc finger, MYN	28.869625	7.801696	P	A	-2.570354	0.2702389	NM_053253	NM_053253	NM_053253	Mm.28331	114602	ENSMUST00000010188	TC1675672	GO:0003674(molecular function)|GO:	chr9:107453306-107453365	mm	9qF1
A_55_P1989765			121.4702	48.099575	P	P	-2.57158	0.3959784									chr7:118277000-118276941	mm	7qF1
A_55_P2003813	Scn3b	Mus musculus sodium channel	2254.4845	1253.912	P	P	-2.575614	0.5561857	NM_178227	NM_178227	NM_178227	Mm.290083	235281	ENSMUST00000049941	TC1597113	GO:0001518(voltage-gated sodium c	chr9:40098873-40098932	mm	9qA5.1
A_55_P2089565	Tbx22	Mus musculus T-box 22 (Tbx2	1343.2915	671.58155	P	P	-2.576025	0.4999522	NM_181319	NM_181319	NM_181319	Mm.137011	245572	ENSMUST00000118986	NP717470	GO:0000122(negative regulation of tr	chrX:104881102-104881161	mm	XqD
A_55_P1980139	Prdm12	Mus musculus PR domain cont	61.180155	2.3627785	P	A	-2.576393	0.03862	NM_001123362	NM_001123362	NM_001123362	Mm.379512	381359	ENSMUST00000113470		GO:0003674(molecular function)|GO:	chr2:31511125-31511184	mm	2qB
A_55_P2059040	Pvalb	Mus musculus parvalbumin (Pv	105.33513	28.356503	P	P	-2.57791	0.2692027	NM_013645	NM_013645	NM_013645	Mm.2766	19293	ENSMUST00000120592	TC1577974	GO:0005509(calcium ion binding)|GO	chr15:78021608-78021549	mm	15qE1
A_55_P2009449	Pnma2	Mus musculus paraneoplastic a	399.28975	157.63175	P	P	-2.579889	0.3947804	NM_175498	NM_175498	NM_175498	Mm.44358	239157	ENSMUST00000122431	TC1582114	GO:0003674(molecular function)|GO:	chr14:67535939-67535998	mm	14qD1
A_66_P102374	Calb1	calbindin 1 [Source:MGI Symb	74.74997	20.10094	P	P	-2.580057	0.268909	ENSMUST00000029876		AK038856	Mm.277665	12307	ENSMUST00000029876	TC1599454	GO:0005499(vitamin D binding)|GO:0	chr4:15835078-15835137	mm	4qA2
A_55_P2039354			840.83135	419.9065	P	P	-2.58017	0.4993944									chr3:051324995-051325054	mm	3qC
A_55_P2177697	Ccdc24	coiled-coil domain containing	35.282245	9.4133445	P	A	-2.595534	0.2668012	ENSMUST00000154439		BC026478	Mm.440118	381546	ENSMUST00000154439		GO:0003674(molecular function)|GO:	chr4:117541416-117541357	mm	4qD2.1
A_55_P2012123	Gm16432	Mus musculus predicted gene	63.129975	2.361649	P	A	-2.601935	0.0374093	NM_001034899	NM_001034899	NM_001034899	Mm.442974	545391	ENSMUST00000094273	TC1591941	GO:0003674(molecular function)|GO:	chr1:179978355-179978414	mm	1qH4
A_52_P257625	Esm1	Mus musculus endothelial cell-	22069.945	11975.31	P	P	-2.605345	0.5426072	NM_023612	NM_023612	NM_023612	Mm.38929	71690	ENSMUST00000038144	NP424788	GO:0001525(angiogenesis)|GO:00015	chr13:114008204-114008263	mm	13qD2.2
A_55_P2077153	Slc27a2	Mus musculus solute carrier fa	13662.11	7412.672	P	P	-2.605623	0.5425715	NM_011978	NM_011978	NM_011978	Mm.290044	26458	ENSMUST00000061491	TC1572822	GO:0000038(very long-chain fatty ac	chr2:126413890-126413949	mm	2qF1
A_66_P137255	Olfr310	Mus musculus olfactory recept	65.55349	2.439379	P	A	-2.606176	0.037212	NM_001011520	NM_001011520	NM_001011520	Mm.377384	258222	ENSMUST00000081918	NP1399042	GO:0004871(signal transducer activi	chr7:93417426-93417367	mm	7qD3
A_55_P2180854	Mrgprg	Mus musculus MAS-related GI	1070.5275	530.50665	P	P	-2.608821	0.4955563	NM_203492	NM_203492	NM_203492	Mm.297839	381974	ENSMUST00000058092	TC1589858	GO:0003674(molecular function)|GO:	chr7:150949710-150949651	mm	7qF5
A_52_P221776	Kif12	Mus musculus kinesin family m	430.22855	167.90015	P	P	-2.611961	0.390258	NM_010616	NM_010616	NM_010616	Mm.216057	16552	ENSMUST00000030042	TC1584697	GO:0000166(nucleotide binding)|GO:0	chr4:62826770-62826711	mm	4qB3
A_51_P518600	Atp6v1c2	Mus musculus ATPase, H+ tra	37.7238	9.923063	P	A	-2.623418	0.2630452	NM_133699	NM_133699	NM_133699	Mm.178798	68775	ENSMUST00000140751	TC1588297	GO:0005765(lysosomal membrane)|G	chr12:17291625-17291566	mm	12qA1.1
A_51_P483544	Aass	Mus musculus aminoadipate-s	461.4984	227.7379	P	P	-2.62445	0.493475	NM_013930	NM_013930	NM_013930	Mm.18651	30956	ENSMUST00000031707	TC1778940	GO:0003824(catalytic activity)|GO:0	chr6:23022727-23022668	mm	6qA3.1
A_51_P431329	Car3	Mus musculus carbonic anhydr	43367.82	27607.35	P	P	-2.629236	0.6365861	NM_007606	NM_007606	NM_007606	Mm.300	12350	ENSMUST00000029076	TC1573384	GO:0004089(carbonate dehydratase	chr3:14871781-14871840	mm	3qA1
A_55_P2317435	AI594674	BX519376 Barstead mouse irr	35.58182	9.3316705	P	A	-2.629301	0.2622595	BX519376		BX519376	Mm.441861	98401		TC1663876		chr1:78312491-78312550	mm	1qC4
A_66_P138999	Plxna2	plexin A2 [Source:MGI Symbol	101.6198	26.606755	P	P	-2.63255	0.2618265	ENSMUST00000125381		AK138854		18845	ENSMUST00000125381	TC1668116	GO:0004872(receptor activity)|GO:00	chr14:108739666-108739725	mm	14qE3
A_55_P1985778			67.37733	2.4212955	P	A	-2.634149	0.0359364	NAP014256-001								chr12:115272033-115271974	mm	12qF1
A_55_P2117623	Tnfrsf13c	Mus musculus tumor necrosis	34.239545	8.9369925	P	A	-2.638665	0.2610138	NM_028075	NM_028075	NM_028075	Mm.240047	72049	ENSMUST00000089161	TC1596344	GO:0001782(B cell homeostasis)|GO	chr15:82054675-82054616	mm	15qE1
A_55_P1966690	Cyp2e1	Mus musculus cytochrome P45	6055.5911	3314.1075	P	P	-2.646946	0.5472806	NM_021282	NM_021282	NM_021282	Mm.21758	13106	ENSMUST00000026552	TC1741326	GO:0000139(Golgi membrane)|GO:00	chr7:147956922-147956981	mm	7qF5
A_55_P2103541	Mrpl9	Mus musculus TIB-55 BB88 cD	65.755785	2.3252045	P	A	-2.647087	0.0353612	AK146237		AK146237	Mm.218515	78523			GO:0003674(molecular function)|GO:	chr3:94254157-94254216	mm	3qF2.1
A_55_P1987406		Mus musculus cell-line 4H3 im	414.0424	158.75315	P	P	-2.660998	0.3834224	AY679096		AY679096	Mm.326349			NP238059		chr16:19085170-19085111	mm	16qA3
A_55_P2186450			72.12613	2.4996095	P	A	-2.66324	0.0346561									chr18:010931562-010931621	mm	18qA1
A_51_P245102	4930591A17Rik	Mus musculus RIKEN cDNA 49	32.428965	8.349432	P	A	-2.665562	0.2574683	NM_026596	NM_026596	NM_026596	Mm.158187	68175		TC1592317	GO:0003674(molecular function)|GO:	chr2:179149723-179149782	mm	2qH4
A_51_P468073	Ggt1	Mus musculus gamma-glutamy	67.83685	17.359555	P	P	-2.677567	0.2559015	NM_008116	NM_008116	NM_008116	Mm.4559	14598	ENSMUST00000006508	TC1575925	GO:0002682(regulation of immune sy	chr10:75048435-75048670	mm	10qC1
A_51_P267063	Ugt3a2	Mus musculus UDP glycosyltra	201.01965	76.59231	P	P	-2.678447	0.381019	NM_144845	NM_144845	NM_144845	Mm.422853	223337	ENSMUST00000072403	TC1576229	GO:0005575(cellular component)|GO	chr15:9300203-9300262	mm	15qA1
A_55_P1956302	H2-Q4	Mus musculus histocompatibili	4564.0895	2479.3735	P	P	-2.679736	0.5432351	NM_001143689	NM_001143689	NM_001143689	Mm.464284	15015	ENSMUST00000081435	TC1582197	GO:0002474(antigen processing and	chr17:35521294-35521353	mm	17qB1
A_55_P1987077			34.48188	8.783628	P	A	-2.686578	0.2547317									chr4:136215475-136215416	mm	4qD3
A_55_P2114437	Pvalb	Mus musculus parvalbumin (Pv	405.1401	153.31905	P	P	-2.697334	0.3784346	NM_013645	NM_013645	NM_013645	Mm.2766	19293	ENSMUST00000120592	TC1577974	GO:0005509(calcium ion binding)|GO	chr15:78022010-78021951	mm	15qE1
A_55_P2071779	1700020N01Rik	Mus musculus RIKEN cDNA 17	72.96069	2.4206855	P	A	-2.698194	0.0331779	NR_027968	NR_027968	NR_027968	Mm.54306	67692	ENSMUST00000057341	TC1589933	GO:0003674(molecular function)|GO:	chr10:21341511-21341570	mm	10qA3
A_55_P2172934	F10	Mus musculus coagulation fact	13445.735	7118.7505	P	P	-2.709334	0.529443	NM_001242368	NM_001242368	NM_001242368	Mm.262589	14058	ENSMUST00000160662	NP744990	GO:0003824(catalytic activity)|GO:0	chr8:13056223-13056290	mm	8qA1.1
A_55_P1968028	Tdgf1	Mus musculus teratocarcinoma	1934.016	1043.1848	P	P	-2.711147	0.5393879	NM_011562	NM_011562	NM_011562	Mm.5090	21667	ENSMUST00000035075	TC1580939	GO:0000187(activation of MAPK act	chr9:110842781-110842722	mm	9qF3
A_55_P2133943	LOC101056066	PREDICTED: Mus musculus ur	129.59695	48.298865	P	P	-2.739817	0.3726852	XM_003945408	XM_003945408	XM_003945408		101056066				chr6:89143133-89143074	mm	6qD1
A_55_P1961983	Uqcr11	Mus musculus ubiquinol-cytoc	35193.23	21971.21	P	P	-2.743056	0.6243022	NM_025650	NM_025650	NM_025650	Mm.379119	66594	ENSMUST00000141683	TC1572384	GO:0005739(mitochondrion)|GO:0005	chr10:79865804-79865745	mm	10qC1
A_55_P2038972	Erich1	Mus musculus glutamate rich 1	131.0406	48.7214	P	P	-2.746387	0.3718039	NM_001034862	NM_001034862	NM_001034862	Mm.268896	234086	ENSMUST00000110813	TC1585841	GO:0003674(molecular function)|GO:	chr8:14027715-14027656	mm	8qA1.1
A_55_P1985835	Dnase1	Mus musculus deoxyribonuclea	3557.3395	1902.981	P	P	-2.7477	0.534945	NM_010061	NM_010061	NM_010061	Mm.440565	13419	ENSMUST00000012009	TC1769749	GO:0003779(actin binding)|GO:00045	chr16:4039965-4040024	mm	16qA1
A_52_P212806	Umod	Mus musculus uromodulin (Um	5906.3795	3155.029	P	P	-2.754082	0.5341731	NM_009470	NM_009470	NM_009470	Mm.10826	22242	ENSMUST00000033169	TC1566331	GO:0000922(spindle pole)|GO:000550	chr7:126606588-126606529	mm	7qF2
A_51_P501844	Cyp26b1	Mus musculus cytochrome P45	1041.0591	494.78155	P	P	-2.764056	0.4752675	NM_175475	NM_175475	NM_175475	Mm.255246	232174	ENSMUST00000077705	TC1583864	GO:0001709(cell fate determination)|	chr6:84522011-84521952	mm	6qC3
A_51_P400366	Rhbg	Mus musculus Rhesus blood gr	306.74915	113.12515	P	P	-2.768995	0.3687872	NM_021375	NM_021375	NM_021375	Mm.103777	58176	ENSMUST00000171887	TC1584625	GO:0005886(plasma membrane)|GO:	chr3:88047324-88047265	mm	3qF1
A_52_P425839	Retnlg	Mus musculus resistin like gam	125.1677	46.160205	P	P	-2.768997	0.3687869	NM_181596	NM_181596	NM_181596	Mm.231288	245195	ENSMUST00000065666	TC1584209	GO:0002408(myeloid dendritic cell ch	chr16:48874468-48874527	mm	16qB5
A_55_P1953003	Atp6v0a4	Mus musculus ATPase, H+ tra	801.77625	379.36525	P	P	-2.780591	0.473156	NM_080467	NM_080467	NM_080467	Mm.462308	140494	ENSMUST00000114908	NP850297	GO:0000220(vacuolar proton-transpo	chr6:37998549-37998490	mm	6qB1
A_52_P229981	Slc51a	Mus musculus solute carrier fa	162.9525	59.8136	P	P	-2.78201	0.3670616	NM_145932	NM_145932	NM_145932	Mm.476915	106407	ENSMUST00000042042	TC1582847	GO:0005215(transporter activity)|GC	chr16:32475930-32475871	mm	16qB3
A_55_P1989813	Gcm1	Mus musculus glial cells missin	605.0471	285.90645	P	P	-2.785461	0.4725359	NM_008103	NM_008103	NM_008103	Mm.1400	14531	ENSMUST00000024104	TC1593178	GO:0003677(DNA binding)|GO:00037	chr5:017758450-017758509	mm	5qA3
A_55_P2015555			86.332465	2.5271295	P	A	-2.798637	0.0292721									chrX:131656264-131656205	mm	XqF1
A_55_P2177726			35.408675	8.509879	P	A	-2.801011	0.2403332	NAP102419-1								chr14:006548593-006548534	mm	14qA1
A_52_P676819	Bnc1	Mus musculus basonuclin 1 (Bi	56.582035	13.59278	P	P	-2.801844	0.2402314	NM_007562	NM_007562	NM_007562	Mm.243802	12173	ENSMUST00000026096	TC1584392	GO:0003677(DNA binding)|GO:00055	chr7:89112037-89111978	mm	7qD3
A_55_P2102738		Mus musculus adult female va	7424.9425	3842.02	P	P	-2.806366	0.5174478	AK036976		AK036976	Mm.30136			TC1717985		chr18:67701336-67701277	mm	18qE1
A_66_P109731		Mus musculus clone 17 immun	60.91211	14.561975	P	P	-2.811413	0.2390654	AY731702		AY731702	Mm.429458			NP048607		chr6:67892296-67892237	mm	6qC1
A_55_P2175579	Olfr1466	Mus musculus olfactory recept	418.72265	151.8702	P	P	-2.81519	0.3626958	NM_146694	NM_146694	NM_146694	Mm.271530	258689	ENSMUST00000075868	TC1636706	GO:0004871(signal transducer activi	chr19:13416809-13416868	mm	19qA
A_55_P2034481	Erdr1	Mus musculus erythroid differe	2197.4575	1155.1345	P	P	-2.825009	0.5256686	NM_133362	NM_133362	NM_133362	Mm.391385	170942	ENSMUST00000180251	NP598082	GO:0003674(molecular function)|GO:0005575(cellular component)|GO:0008285(negative regulation of cell proliferation)|GO:0030336(negative regulation of cell migration)|GO:0035019(somatic stem cell maintenance)			
A_55_P2413458	Tfcp2l1	Mus musculus transcription fac	114.5924	41.22786	P	P	-2.837625	0.3597783	NM_023755	NM_023755	NM_023755	Mm.24621	81879	ENSMUST00000027629	TC1656256	GO:0000122(negative regulation of tr	chr1:120577637-120577696	mm	1qE2.3
A_52_P356093	B3galt2	Mus musculus UDP-Gal:betaG	116.895	41.91055	P	P	-2.847258	0.3585316	NM_020025	NM_020025	NM_020025	Mm.285580	26878	ENSMUST00000038252	TC1577996	GO:0005575(cellular component)|GO	chr1:145496531-145496590	mm	1qF
A_55_P2166486	Cd44	Mus musculus CD44 antigen (C	86.58375	2.363011	P	A	-2.854815	0.0272916	NM_009851	NM_009851	NM_009851	Mm.423621	12505	ENSMUST00000111198	TC1596221	GO:0001558(regulation of cell growth	chr2:102672704-102672645	mm	2qE2
A_51_P321734	Bhmt2	Mus musculus betaine-homocy	37.553325	8.765048	P	A	-2.858558	0.2334027	NM_022884	NM_022884	NM_022884	Mm.29981	64918	ENSMUST00000015941	TC1581292	GO:0005575(cellular component)|GO	chr13:94426438-94426379	mm	13qC3
A_52_P439358	Kl	Mus musculus klotho (Kl), mRN	40.49947	9.42576	P	A	-2.864168	0.2327379	NM_013823	NM_013823	NM_013823	Mm.6500	16591	ENSMUST00000078856	TC1582269	GO:0002526(acute inflammatory resp	chr5:151795712-151795771	mm	5qG3
A_55_P1985422		Q811B0_MOUSE (Q811B0) Fru	1772.624	922.9813	P	P	-2.867096	0.5206865	TC1700876						TC1700876				
A_51_P323770	Wfdc15a	Mus musculus WAP four-disulf	452.50435	208.58805	P	P	-2.877535	0.4609636	NM_183271	NM_183271	NM_183271	Mm.142735	68221	ENSMUST00000063251	TC1586601	GO:0003674(molecular function)|GO:	chr2:164025466-164025407	mm	2qH3
A_51_P395856	Slc22a7	Mus musculus solute carrier fa	118.67145	27.21627	P	P	-2.893082	0.2293413	NM_144856	NM_144856	NM_144856	Mm.387538	108114	ENSMUST00000087012	TC1580408	GO:0005886(plasma membrane)|GO:	chr17:46569331-46569272	mm	17qC
A_66_P118316	1700047G07Rik	PREDICTED: Mus musculus RI	109.00025	37.91534	P	P	-2.931219	0.3478464	XM_003945638	XM_003945638	XM_003945638		73323		TC1657259	GO:0003674(molecular function)|GO:	chr8:99036348-99036407	mm	8qD1

A_52_P90363	Ifi27l2a	Mus musculus interferon, alpha	91171.67	55075.045	P	P	-2.935393	0.6040807	NM_029803	NM_029803	NM_029803	NM_029803	Mm.271275	76933	ENSMUST00000055071	TC1582985	GO:0003674(molecular function)|GO:	chr12:104680453-104680394	mm	12qE
A_51_P192089	Pinx1	Mus musculus PIN2/TERF1 int	521.9381	236.1811	P	P	-2.946287	0.4525079	NM_028228	NM_028228	NM_028228	NM_028228	Mm.379214	72400	ENSMUST00000022528	TC1781755	GO:0000228(nuclear chromosome)|G	chr14:64538435-64538494	mm	14qD1
A_55_P1967002			6741.9645	3442.809	P	P	-2.953082	0.5106537										chr7:151963159-151963218	mm	7qF5
A_55_P2148400			6626.024	3380.651	P	P	-2.956941	0.5102081										chr5:119436656-119436597	mm	5qF
A_55_P2019312	Car12	Mus musculus carbonic anhydr	574.7552	259.11785	P	P	-2.960068	0.4508317	NM_178396	NM_178396	NM_178396	NM_178396	Mm.277921	76459	ENSMUST00000071889	NP515859	GO:0004089(carbonate dehydratase	chr9:66614542-66614601	mm	9qC
A_55_P2069550			58.404075	12.92071	P	P	-2.963904	0.2212296										chr12:117744959-117744900	mm	12qF2
A_55_P2004511	Cd300lf	Mus musculus CD300 antigen	1745.2885	887.42825	P	P	-2.972015	0.5084708	NM_001169153	NM_001169153	NM_001169153	NM_001169153	Mm.277387	246746	ENSMUST00000106561	TC1583435	GO:0005515(protein binding)|GO:000	chr11:114977855-114977796	mm	11qE2
A_55_P1962901	Osr1	Mus musculus odd-skipped rel	196.711	66.978975	P	P	-2.9905	0.3404943	NM_011859	NM_011859	NM_011859	NM_011859	Mm.45055	23967	ENSMUST00000057021	TC1583262	GO:0000122(negative regulation of tr	chr12:9587390-9587449	mm	12qA1.1
A_55_P2143516			560.7519	250.54825	P	P	-2.993362	0.4468077										chr14:027097030-027096971	mm	14qA3
A_51_P472249	Slc7a7	Mus musculus solute carrier fa	10172.8	5025.195	P	P	-3.002856	0.4939835	NM_011405	NM_011405	NM_011405	NM_011405	Mm.142455	20540	ENSMUST00000000984	TC1644314	GO:0003333(amino acid transmembr	chr14:54989225-54989166	mm	14qC2
A_55_P2087746	Ppp6r2	Mus musculus protein phospha	36.971265	8.003566	P	A	-3.00658	0.2164807	NM_026813	NM_026813	NM_026813	NM_026813	Mm.184055	71474	ENSMUST00000088788	TC1586669	GO:0005737(cytoplasm)|GO:0019903	chr15:89112375-89112434	mm	15qE3
A_51_P244950	Dpys	Mus musculus dihydropyrimidin	58.720715	12.69863	P	P	-3.008635	0.2162547	NM_001164466	NM_001164466	NM_001164466	NM_001164466	Mm.275974	64705	ENSMUST00000110306	TC1583458	GO:0002058(uracil binding)|GO:0002	chr15:39614608-39614549	mm	15qB3.1
A_55_P2131428	Tlr5	Mus musculus toll-like recepto	50.164765	10.814785	P	A	-3.014732	0.2155853	NM_016928	NM_016928	NM_016928	NM_016928	Mm.116894	53791	ENSMUST00000110997		GO:0001819(positive regulation of cy	chr1:184906031-184906090	mm	1qH5
A_55_P2005005	Cdk5rap1	Mus musculus CDK5 regulator	329.3191	110.97725	P	P	-3.019208	0.33699	NM_025876	NM_025876	NM_025876	NM_025876	Mm.289427	66971	ENSMUST00000028990	TC1580425	GO:0000079(regulation of cyclin-dep	chr2:154161196-154161137	mm	2qH1
A_51_P232399	Acy3	Mus musculus aspartoacylase	1837.596	922.9393	P	P	-3.026385	0.5022537	NM_027857	NM_027857	NM_027857	NM_027857	Mm.29735	71670	ENSMUST00000054030	TC1592659	GO:0004046(aminoacylase activity)|(	chr19:3989878-3989937	mm	19qA
A_55_P2004205		Mus musculus activated splee	346.3699	116.34845	P	P	-3.028131	0.3359081	AK157330		AK157330	AK157330						chr14:048706339-048706398	mm	14qC1
A_55_P1974780			1653.4635	727.8804	P	P	-3.048558	0.4402156										chr5:058899406-058899465	mm	5qC2
A_55_P2136752	Ermn	Mus musculus ermin, ERM-like	1614.1315	710.21575	P	P	-3.050388	0.4399987	NM_029972	NM_029972	NM_029972	NM_029972	Mm.91712	77767	ENSMUST00000090940	TC1584254	GO:0001763(morphogenesis of a brai	chr2:57900529-57900470	mm	2qC1.1
A_52_P408736	Slc16a7	solute carrier family 16 (monoc	59.927243	12.647815	P	A	-3.056521	0.2110528	ENSMUST00000063318		AK085398	AK085398	Mm.29161	20503	ENSMUST00000063318	TC1623955	GO:0005737(cytoplasm)|GO:0005886	chr10:124663635-124663576	mm	10qD3
A_55_P2092831		Mus musculus 17 days embryo	11631.99	5642.138	P	P	-3.080097	0.4850535	AK146780		AK146780	AK146780				TC1626172		chr14:094684384-094684325	mm	14qE2.1
A_55_P1962602		Mus musculus 2 days neonate	4299.351	2128.74	P	P	-3.089512	0.4951305	AK148702		AK148702	AK148702						chr9:10377916-10377857	mm	9qA1
A_55_P1992884			77.76054	15.887255	P	P	-3.12038	0.20431	NAP114441-1									chr5:136686914-136686855	mm	5qG2
A_52_P150236	Apom	Mus musculus apolipoprotein M	137.94855	44.61489	P	P	-3.133292	0.3234169	NM_018816	NM_018816	NM_018816	NM_018816	Mm.2161	55938	ENSMUST00000025249	TC1579902	GO:0005319(lipid transporter activity	chr17:35267106-35267047	mm	17qB1
A_55_P2120141			7402.056	3542.469	P	P	-3.136994	0.4785791										chr6:126244172-126244231	mm	6qF3
A_52_P321140	Defb1	Mus musculus defensin beta 1	779.98115	334.8786	P	P	-3.141436	0.4293419	NM_007843	NM_007843	NM_007843	NM_007843	Mm.431316	13214	ENSMUST00000051017	TC1581749	GO:0005576(extracellular region)|GO	chr8:22905041-22905100	mm	8qA2
A_55_P2116149			6099.045	2981.6995	P	P	-3.145661	0.4888797										chr7:097951864-097951805	mm	7qE1
A_51_P360655	Slc22a6	Mus musculus solute carrier fa	1235.723	528.70885	P	P	-3.154329	0.4278539	NM_008766	NM_008766	NM_008766	NM_008766	Mm.30090	18399	ENSMUST00000010250	TC1577678	GO:0005452(inorganic anion exchang	chr19:8700868-8700927	mm	19qA
A_55_P1991783			7719.4425	3678.1375	P	P	-3.155632	0.4764771										chr5:071567078-071567019	mm	5qC3.1
A_55_P2108933	Dao	Mus musculus D-amino acid o	423.14325	135.01695	P	P	-3.170747	0.3190809	NM_001286396	NM_001286396	NM_001286396	NM_001286396	Mm.20115	13142		TC1716036	GO:0003884(D-amino-acid oxidase a	chr5:114475410-114475469	mm	5qF
A_51_P499816	Pbld1	Mus musculus phenazine biosy	39.474195	7.8130165	P	A	-3.182802	0.1979272	NM_026701	NM_026701	NM_026701	NM_026701	Mm.261389	68371	ENSMUST00000178684	TC1582405	GO:0003674(molecular function)|GO:	chr10:62540038-62540097	mm	10qB4
A_52_P320193	Clec2h	Mus musculus C-type lectin do	71.12807	13.993115	P	A	-3.194721	0.1967313	NM_053165	NM_053165	NM_053165	NM_053165	Mm.197689	94071	ENSMUST00000032518	TC1582395	GO:0004888(transmembrane signalin	chr6:128626525-128626584	mm	6qF3
A_55_P2028847			5698.7575	2755.0465	P	P	-3.195049	0.4834469										chr9:032481344-032481285	mm	9qA4
A_55_P2004168			1201.9075	508.25025	P	P	-3.197842	0.4228697										chr18:040845710-040845651	mm	18qB3
A_66_P131389	Ugt2b38	Mus musculus UDP glucuronos	453.7628	191.41465	P	P	-3.206908	0.4218386	NM_133894	NM_133894	NM_133894	NM_133894	Mm.212589	100559	ENSMUST00000072818	TC1575532	GO:0005575(cellular component)|GO	chr5:87839404-87839345	mm	5qE1
A_55_P1962603	Cplx2	Mus musculus complexin 2 (Cp	6994.0545	3369.0235	P	P	-3.211064	0.4816982	NM_009946	NM_009946	NM_009946	NM_009946	Mm.268902	12890	ENSMUST00000026985	NP1216959	GO:0000149(SNARE binding)|GO:000	chr13:54481348-54481407	mm	13qB1
A_55_P2070766			7876.926	3702.01	P	P	-3.21375	0.4699816										chr13:060250825-060250884	mm	13qB2
A_55_P2035038			8123.7525	3784.526	P	P	-3.251051	0.4658593										chr15:054645465-054645406	mm	15qD1
A_51_P230734	Slc34a3	Mus musculus solute carrier fa	222.88265	68.63508	P	P	-3.269347	0.3079427	NM_080854	NM_080854	NM_080854	NM_080854	Mm.490356	142681	ENSMUST00000006638	TC1587588	GO:0005436(sodium:phosphate symp	chr2:25084497-25084438	mm	2qA3
A_65_P20654			57.351235	10.79069	P	A	-3.282425	0.188151	TC1678291							TC1678291		chr5:137339588-137339529	mm	5qG2
A_55_P2086954			7513.59	3472.9745	P	P	-3.284205	0.4622257										chr14:090900219-090900278	mm	14qE2.1
A_55_P2077522		Mus musculus adult male hippo	572.07355	236.33815	P	P	-3.284414	0.4131255	AK013651		AK013651	AK013651				TC1721473		chr8:040821891-040821832	mm	8qA4
A_55_P1981830			1821.8085	750.83195	P	P	-3.293323	0.4121355										chrY_random:036219774-036219715		
A_52_P189984	Lrrc1	Mus musculus leucine rich rep	69.88726	13.04283	P	A	-3.298423	0.1866267	NM_172528	NM_172528	NM_172528	NM_172528	Mm.28534	214345	ENSMUST00000113421	TC1591656	GO:0003674(molecular function)|GO:	chr9:77302974-77300479	mm	9qD
A_55_P2011410	Pdzk1	Mus musculus PDZ domain con	674.9447	277.57955	P	P	-3.301196	0.4112627	NM_021517	NM_021517	NM_021517	NM_021517	Mm.489677	59020	ENSMUST00000058865	TC1758289	GO:0005124(scavenger receptor bind	chr3:96674286-96674345	mm	3qF2.1
A_55_P2029902	Gab3	Mus musculus growth factor re	2883.5535	1357.95	P	P	-3.310986	0.4709294	NM_181584	NM_181584	NM_181584	NM_181584	Mm.267056	210710	ENSMUST00000129495	TC1588323	GO:0005515(protein binding)|GO:000	chrX:72245395-72245336	mm	XqA7.3
A_55_P2063505			6564.254	3090.8105	P	P	-3.311686	0.4708548										chr12:033682931-033682872	mm	12qA3
A_51_P333518	Sh3rf2	Mus musculus SH3 domain con	96.72717	17.851115	P	P	-3.320417	0.1845512	NM_172966	NM_172966	NM_172966	NM_172966	Mm.45472	269016	ENSMUST00000074679	TC1584677	GO:0004864(protein phosphatase inh	chr18:42317002-42317061	mm	18qB3
A_55_P1982227			7167.0125	3367.73	P	P	-3.320721	0.4698931										chr17:081734701-081734642	mm	17qE3
A_55_P1975565	Ttc34	Mus musculus tetratricopeptid	61.2292	11.29245	P	A	-3.321718	0.1844292	NM_172878	NM_172878	NM_172878	NM_172878	Mm.72944	242800	ENSMUST00000050220	NP1481750	GO:0003674(molecular function)|GO:	chr4:154241174-154241233	mm	4qE2
A_51_P120295	Cyp2d26	Mus musculus cytochrome P45	1176.3445	478.52205	P	P	-3.341827	0.4067873	NM_029562	NM_029562	NM_029562	NM_029562	Mm.439931	76279	ENSMUST00000006094	TC1771800	GO:0004497(monooxygenase activity	chr15:82620634-82620575	mm	15qE1
A_55_P2021398			7707.509	3504.515	P	P	-3.353818	0.4546884										chr1:012764615-012764674	mm	1qA3
A_52_P499907	Kcnj1	Mus musculus potassium inwar	1616.74	654.7974	P	P	-3.358079	0.4050109	NM_019659	NM_019659	NM_019659	NM_019659	Mm.390168	56379	ENSMUST00000172015	TC1585425	GO:0000166(nucleotide binding)|GO:(	chr9:32206612-32206671	mm	9qA4
A_55_P2101944			5300.426	2463.578	P	P	-3.368993	0.4647887										chr7:146146994-146146935	mm	7qF4
A_55_P2077497		predicted gene, 17678 [Source	6725.667	3092.91	P	P	-3.416044	0.4598667	ENSMUST00000167348		AK158706	AK158706	Mm.235238		ENSMUST00000167348	TC1682940		chr3:57850492-57850670	mm	3qD
A_55_P2112986	Klk1b22	Mus musculus kallikrein 1-rela	213.55	61.84073	P	P	-3.439919	0.2895843	NM_010114	NM_010114	NM_010114	NM_010114	Mm.5193	13646	ENSMUST00000077528	TC1583968	GO:0003824(catalytic activity)|GO:0	chr7:51371648-51371707	mm	7qB4
A_55_P2123486	Map3k10	Mus musculus mitogen-activat	57.309945	9.8883645	P	A	-3.452751	0.1725419	NM_001081292	NM_001081292	NM_001081292	NM_001081292	Mm.389883	269881	ENSMUST00000108341		GO:0000166(nucleotide binding)|GO:(	chr7:28442925-28442866	mm	7qA3
A_55_P2008403	Coq9	coenzyme Q9 homolog (yeast)	38.003965	6.55103	P	A	-3.454625	0.1723775	ENSMUST00000160364	XM_918040	XM_918040	XM_918040		67914	ENSMUST00000160364	TC1727960	GO:0003674(molecular function)|GO:	chr8:97374758-97374817	mm	8qD1
A_55_P1960479			5824.574	2640.1075	P	P	-3.479894	0.4532705										chr7:066891025-066891084	mm	7qC
A_55_P2019833			7047.6285	3190.0565	P	P	-3.486021	0.4526425										chr18:048518596-048518537	mm	18qC
A_55_P2139430			5316.9065	2406.166	P	P	-3.486924	0.45255										chr1:171821341-171821282	mm	1qH3
A_55_P1997595	Slc6a19	Mus musculus solute carrier fa	326.8997	93.06395	P	P	-3.487254	0.2846866	NM_028878	NM_028878	NM_028878	NM_028878	Mm.271635	74338	ENSMUST00000132085	TC1581859	GO:0003333(amino acid transmembr	chr13:73818777-73818718	mm	13qC1
A_55_P2040793			164.27735	46.71547	P	P	-3.490346	0.2843695										chrY_random:024673982-024673923		
A_55_P2041457			4292.777	1940.8065	P	P	-3.491225	0.4521098										chr5:053747039-053747098	mm	5qC1
A_55_P2077501			5673.099	2563.6745	P	P	-3.493275	0.4519002										chr1:186475396-186475455	mm	1qH5
A_65_P05872		AHNAK nucleoprotein 2 [Sourc	72.155395	12.167045	P	A	-3.497938	0.1686228	ENSMUST00000101010	XM_003945498	XM_003945498	XM_003945498	Mm.313169		ENSMUST00000101010	TC1583682		chr12:114012303-114012244	mm	12qF1
A_55_P1961761	Dcc	Mus musculus deleted in color	6397.4275	2883.5535	P	P	-3.504671	0.4507364	NM_007831	NM_007831	NM_007831	NM_007831	Mm.167882	13176	ENSMUST00000144892	TC1606562	GO:0001764(neuron migration)|GO:00	chr7:068417221-068417162	mm	7qC
A_55_P1984745	Pappa2	Mus musculus pappalysin 2 (Pa	49.779355	8.358434	P	A	-3.506273	0.1679096	NM_001085376	NM_001085376	NM_001085376	NM_001085376	Mm.458621	23850	ENSMUST00000159861	TC1636572	GO:0004222(metalloendopeptidase a	chr1:160641962-160641903	mm	1qH1
A_51_P410772	Rhcg	Mus musculus Rhesus blood gr	225.3396	63.34468	P	P	-3.522361	0.2811076	NM_019799	NM_019799	NM_019799	NM_019799	Mm.10909	56315	ENSMUST00000172788	TC1581472	GO:0005886(plasma membrane)|GO:(	chr7:86738337-86738278	mm	7qD3
A_55_P1961760			7436.1755	3326.946	P	P	-3.537505	0.4474001										chr7:068417161-068417102	mm	7qC
A_55_P2075731			6344.845	2835.5745	P	P	-3.542349	0.44691										chr17_random:000063672-000063613		
A_55_P2050988			610.4909	235.25655	P	P	-3.542809	0.3853564										chr9:053825164-053825223	mm	9qA5.3
A_55_P2064171			4660.2315	2081.92	P	P	-3.544013	0.4467418										chr6:142919207-142919148	mm	6qG3
A_55_P1960999	Pigr	Mus musculus polymeric immu	8847.996	3839.677	P	P	-3.55138	0.4339601	NM_011082	NM_011082	NM_011082	NM_011082	Mm.276414	18703	ENSMUST00000027675	NP1221957	GO:0005154(epidermal growth factor	chr14:070241981-070241922	mm	14qD2
A_55_P2020035			6299.3205	2808.6515	P	P	-3.552687	0.4458658										chr10:003971423-003971364	mm	10qA1
A_55_P2154107	Gcm1	Mus musculus glial cells missin	3992.4625	1778.116	P	P	-3.557622	0.4453682	NM_008103	NM_008103	NM_008103	NM_008103	Mm.1400	14531	ENSMUST00000151882	NP1221957	GO:0003677(DNA binding)|GO:00037	chr7:068290936-068290995	mm	7qC
A_55_P2068248			3626.9285	1612.485	P	P	-3.565382	0.4445869										chr14:084391942-084392001	mm	14qD3
A_55_P1962699		Mus musculus 3 days neonate	4271.189	1898.5325	P	P	-3.566271	0.4444974	AK080285		AK080285	AK080285				TC1776563		chr10:089001547-089001606	mm	10qC2
A_55_P1991806			90.54932	14.5687	P	P	-3.590232	0.1608924	NAP018083-001									chrX:083643602-083643543	mm	XqC1
A_55_P2333580	BC020402	Mus musculus cDNA sequence	53.96911	8.588218	P	A	-3.611868	0.1591321	NR_033219	NR_033219	NR_033219	NR_033219	Mm.393120	407824		TC1599218		chr10:7398826-7398769	mm	10qA1
A_51_P295034	Klk1b4	Mus musculus kallikrein 1-rela	411.09595	111.8765	P	P	-3.612309	0.2721421	NM_010915	NM_010915	NM_010915	NM_010915	Mm.440886	18048	ENSMUST00000077354	TC1590361	GO:0003824(catalytic activity)|GO:0	chr7:51466302-51466457	mm	7qB4
A_55_P2138739			498.56445	188.1826	P	P	-3.619802	0.3774489										chrY_random:022013721-022013780		
A_52_P75777	Lgr6	Mus musculus adult male aorta	44.696725	7.056707	P	A	-3.627408	0.1578797	AK040883		AK040883	AK040883	Mm.279594	329252		TC1598991	GO:0003674(molecular function)|GO:	chr1:136880597-136880538	mm	1qE4
A_55_P2096127			2930.7425	1283.8265	P	P	-3.630794	0.438055										chr13:102097488-102097429	mm	13qD1
A_55_P1967591			5048.56	2202.094	P	P	-3.649726	0.4361826										chr13:006310513-006310454	mm	13qA1
A_52_P185162	Slc26a3	solute carrier family 26, memb	45.66626	7.0448465	P	A	-3.672921	0.1542681	ENSMUST00000110854		BC037066	BC037066		13487	ENSMUST00000110854	TC1586988	GO:0005515(protein binding)|GO:000	chr12:32137633-32137692	mm	12qA3
A_55_P2082841			6289.372	2724.88	P	P	-3.679524	0.4332515										chr1:173964405-173964464	mm	1qH3
A_55_P2068247			4398.5265	1897.3595	P	P	-3.698835	0.4313625										chr15:013245897-013245838	mm	15qA1
A_55_P2058557	Dnajb5	DnaJ (Hsp40) homolog, subfam	61.219825	9.113193	P	A	-3.743102	0.1488602	ENSMUST00000084662		AK158972	AK158972	Mm.20437	56323	ENSMUST00000084662	TC1632135	GO:0000122(negative regulation of tr	chr4:42972193-42972252	mm	4qA5
A_52_P217796	Ift46	Mus musculus intraflagellar tra	98.57553	14.58711	P	P	-3.754778	0.147979	NM_023831	NM_023831	NM_023831	NM_023831	Mm.278597	76568	ENSMUST00000002099	NP731282	GO:0005515(protein binding)|GO:000	chr9:44600737-44600796	mm	9qA5.2
A_55_P1989419	AI987944	Mus musculus expressed sequ	150.14695	38.81479	P	P	-3.754895	0.258512	NM_183167	NM_183167	NM_183167	NM_183167	Mm.379390	233168	ENSMUST00000071804	TC1581553	GO:0003674(molecular function)|GO:	chr7:48629773-48629714	mm	7qB3
A_52_P185014	Lrrc3	Mus musculus leucine rich rep	56.70888	8.3782485	P	A	-3.757939	0.1477414	NM_145152	NM_145152	NM_145152	NM_145152	Mm.133301	237387	ENSMUST00000057608	TC1586488	GO:0003674(molecular function)|GO:	chr10:77363748-77363689	mm	10qC1
A_55_P2171788			5315.495	2257.3185	P	P	-3.767965	0.4246676	NAP072206-1									chr9:095434704-095434763	mm	9qE3.3
A_51_P155503	Klk1b8	Mus musculus kallikrein 1-rela	504.3144	129.22425	P	P	-3.779419	0.2562375	NM_008457	NM_008457	NM_008457	NM_008457	Mm.435488	16624	ENSMUST00000072204	TC1586404	GO:0003824(catalytic activity)|GO:0	chr7:51209619-51209678	mm	7qB4
A_55_P2148935			2887.512	1216.0615	P	P	-3.804776	0.4211451										chrY_random:056067404-056067345		
A_55_P1981829	Rhox8	Mus musculus reproductive ho	5294.8725	2216.162	P	P	-3.832107	0.4185487	NM_001004193	NM_001004193	NM_001004193	NM_001004193	Mm.336025	434768	ENSMUST00000071885	TC1596027	GO:0003677(DNA binding)|GO:00055	chrX:35231521-35231462	mm	XqA3.3
A_55_P1990221		lag69c05.y1 Colon epithelia pr	80.13385	11.39987	P	A	-3.832289	0.1422604	CF969522		CF969522	CF969522	Mm.418696			TC1700694		chr11:88854469-88854410	mm	11qC
A_52_P877015	Thrb	Mus musculus thyroid hormone	47.172755	6.598375	P	A	-3.86552	0.1398768	NM_009380	NM_009380	NM_009380	NM_009380	Mm.32563	21834	ENSMUST00000022304	TC1668793	GO:0000122(negative regulation of tr	chr14:18869432-18869491	mm	14qA2
A_55_P2114697	Gcm1	Mus musculus glial cells missin	6513.5105	2675.319	P	P	-3.915404	0.4107338	NM_008103	NM_008103	NM_008103	NM_008103	Mm.1400	14531	ENSMUST00000024104	TC1677874	GO:0003677(DNA binding)|GO:00037	chr7:123731626-123731567	mm	7qF1
A_51_P343350	Amn	Mus musculus amnionless (Am	152.69885	36.850505	P	P	-3.945775	0.241328	NM_033603	NM_033603	NM_033603	NM_033603	Mm.197639	93835	ENSMUST00000021707	TC1579838	GO:0007275(multicellular organismal	chr12:112514095-112514243	mm	12qF1
A_55_P2207498	Image:619641	BB161756 RIKEN full-length e	81.886585	10.665268	P	A	-4.005844	0.1302444	BB161756		BB161756	BB161756	Mm.354189	541456		TC1629577		chr3:130421199-130421140	mm	3qG3
A_55_P2180996	Rims3	Mus musculus regulating synap	62.66851	7.609758	P	A	-4.143681	0.1214287	NM_182929	NM_182929	NM_182929	NM_182929	Mm.192502	242662	ENSMUST00000171363	TC1604489	GO:0005623(cell)|GO:0006810(transp	chr4:120563927-120563986	mm	4qD2.2
A_51_P309488	1810058N15Rik	Mus musculus 10 day old male	63.080745	7.611853	P	A	-4.156034	0.1206684	AK007900		AK007900	AK007900		76513		TC1627128	GO:0003674(molecular function)|GO:	chr19:5561414-5561473	mm	19qA
A_52_P653825	Keg1	Mus musculus kidney expresse	248.1594	53.23709	P	P	-4.272443	0.2145278	NM_029550	NM_029550	NM_029550	NM_029550	Mm.218561	64697	ENSMUST00000025598	TC1574528	GO:0005737(cytoplasm)|GO:0005739	chr19:12794066-12794125	mm	19qA
A_55_P2042183		Mus musculus cDNA clone IMA	65.090115	7.3683615	P	A	-4.281645	0.1132025	BC024537		BC024537	BC024537	Mm.288669			TC1609759		chrX:155180671-155180732	mm	XqF4
A_66_P115938		predicted gene 9991 [Source:M	46.864465	5.2467525	P	A	-4.303422	0.1119559	ENSMUST00000070048	XR_106072	XR_106072	XR_106072	Mm.122412		ENSMUST00000070048	TC1594698		chr1:92576299-92576358	mm	1qD
A_51_P186547	Pah	Mus musculus phenylalanine h	600.38515	187.41765	P	P	-4.325036	0.3121624	NM_008777	NM_008777	NM_008777	NM_008777	Mm.263539	18478	ENSMUST00000020241	TC1778538	GO:0003824(catalytic activity)|GO:0	chr10:87046641-87046701	mm	10qC1
A_55_P2049216	Oxgr1	Mus musculus oxoglutarate (al	196.53885	41.187895	P	P	-4.337378	0.2095662	NM_001001490	NM_001001490	NM_001001490	NM_001001490	Mm.138520	239283	ENSMUST00000058213		GO:0001609(G-protein coupled aden	chr14:120419035-120418976	mm	14qE4
A_55_P2013236	S100g	Mus musculus S100 calcium bi	14053.646	5038.3655	P	P	-4.360079	0.3585095	NM_009789	NM_009789	NM_009789	NM_009789	Mm.6891	12309	ENSMUST00000038769	TC1580034	GO:0005499(vitamin D binding)|GO:0	chrX:159399983-159399924	mm	XqF4
A_55_P2027801	BC052040	Mus musculus cDNA sequence	89.250945	9.6572335	P	A	-4.370476	0.1082032	NM_001145898	NM_001145898	NM_001145898	NM_001145898	Mm.134929	399568	ENSMUST00000110918	TC1582857	GO:0003674(molecular function)|GO:	chr2:115604445-115604504	mm	2qE4
A_66_P130035	Klk1b24	Mus musculus kallikrein 1-rela	966.4025	296.61405	P	P	-4.387857	0.306926	NM_010643	NM_010643	NM_010643	NM_010643	Mm.483098	16617	ENSMUST00000073713	TC1589542	GO:0003824(catalytic activity)|GO:0	chr7:51447637-51447696	mm	7qB4
A_52_P426863	C230052I12Rik	Mus musculus RIKEN cDNA C2	52.66402	5.125543	P	A	-4.578852	0.0973253	NM_178643	NM_178643	NM_178643	NM_178643	Mm.389855	101831	ENSMUST00000154597	TC1578945	GO:0003677(DNA binding)|GO:00036	chr7:36180268-36180100	mm	7qB2
A_55_P2184177		Mus musculus visual cortex cD	56.06168	5.391784	P	A	-4.602216	0.0961759	AK158955		AK158955	AK158955						chr7:66486890-66486831	mm	7qC
A_55_P2333061	A230101C19Rik	Mus musculus adult male hypo	61.195915	5.6989475	P	A	-4.665589	0.0931263	AK020705		AK020705	AK020705	Mm.488092	77756				chr11:89500032-89499973	mm	11qC
A_55_P2111163	S100g	Mus musculus S100 calcium bi	17938.115	5930.553	P	P	-4.703083	0.3306118	NM_009789	NM_009789	NM_009789	NM_009789	Mm.6891	12309	ENSMUST00000038769	TC1593177	GO:0005499(vitamin D binding)|GO:0	chrX:159399997-159399938	mm	XqF4
A_66_P132695	1700015F17Rik	Mus musculus RIKEN cDNA 17	52.283005	4.366868	P	A	-4.879619	0.0835237	NM_001200025	NM_001200025	NM_001200025	NM_001200025	Mm.276086	381716	ENSMUST00000156282	TC1631570	GO:0003674(molecular function)|GO:	chr5:5437896-5437837	mm	5qA1
A_51_P337269	Aldob	Mus musculus aldolase B, fruc	4548.598	1465.8335	P	P	-4.9875	0.3222605	NM_144903	NM_144903	NM_144903	NM_144903	Mm.482116	230163	ENSMUST00000029987	TC1756303	GO:0003824(catalytic activity)|GO:0	chr4:49549467-49549408	mm	4qB1
A_55_P2132132	Gm5532	Mus musculus 17 days embryo	83.701125	6.592623	P	A	-4.995017	0.0787639	AK147021		AK147021	AK147021	Mm.359304	433367				chr1:157367363-157367304	mm	1qG3
A_55_P1985346		uy13d02.y1 McCarrey Eddy sp	65.46688	4.9723085	P	A	-5.066525	0.0759515	BG099879		BG099879	BG099879	Mm.410648					chr4:139952451-139952510	mm	4qD3
A_55_P2111322	Iglv1	Mus musculus clone 46s58 imr	1607.732	410.0971	P	P	-5.07499	0.255078	AY170578		AY170578	AY170578	Mm.326349	16142		TC1572776		chr16:19061964-19061905	mm	16qA3
A_55_P2068312	Gm17019	Mus musculus predicted gene	83.53773	5.6297445	P	A	-5.301693	0.0673916	NM_182957	NM_182957	NM_182957	NM_182957	Mm.368256	66773	ENSMUST00000179506	TC1661661		chr5:15031721-15031662	mm	5qA2
A_55_P2304614	C530044C16Rik	Mus musculus 12 days embryo	59.32794	3.770435	P	A	-5.417051	0.0635524	AK049716		AK049716	AK049716	Mm.131454	319981				chr6:50764973-50765032	mm	6qB2.3
A_51_P245414	Klk1	Mus musculus kallikrein 1 (Klk1	885.9825	196.49905	P	P	-5.594335	0.2217866	NM_010639	NM_010639	NM_010639	NM_010639	Mm.142722	16612	ENSMUST00000075162	TC1770892	GO:0003824(catalytic activity)|GO:0	chr7:51484170-51484325	mm	7qB4
A_51_P413147	Klk1b3	Mus musculus kallikrein 1-rela	1209.1565	242.08475	P	P	-5.974413	0.2002096	NM_008693	NM_008693	NM_008693	NM_008693	Mm.439740	18050	ENSMUST00000085450	TC1588577	GO:0003824(catalytic activity)|GO:0	chr7:51457064-51457122	mm	7qB4
A_51_P130905	Gcm1	Mus musculus glial cells missin	105.41562	3.470764	P	A	-6.710451	0.0329246	NM_008103	NM_008103	NM_008103	NM_008103	Mm.1400	14531	ENSMUST00000024104	TC1593178	GO:0003677(DNA binding)|GO:00037	chr9:77912984-77913043	mm	9qE1
A_55_P2074015	LOC101055740	PREDICTED: Mus musculus un	84.4861	2.6778995	P	A	-6.785221	0.0316963	XM_003945332	XM_003945332	XM_003945332	XM_003945332		101055740				chr2:30638865-30638924	mm	2qB
A_52_P311313	Hcn1	Mus musculus hyperpolarizatio	91.294815	2.83773	P	A	-6.823642	0.0310831	NM_010408	NM_010408	NM_010408	NM_010408	Mm.343429	15165	ENSMUST00000006991	TC1588931	GO:0000166(nucleotide binding)|GO:(	chr13:118765058-118765117	mm	13qD2.3
A_55_P1997574	Gm4850	Mus musculus predicted pseud	94.02789	2.6840725	P	A	-6.99114	0.0285455	NR_015347	NR_015347	NR_015347	NR_015347	Mm.25966	226957		TC1640895		chr1:31324718-31324777	mm	1qB
A_55_P2051239	Gpr142	Mus musculus G protein-coupl	101.24976	2.6716365	P	A	-7.145808	0.0263866	NM_181749	NM_181749	NM_181749	NM_181749	Mm.269127	217302	ENSMUST00000045319	NP1489884	GO:0004871(signal transducer activi	chr11:114668000-114668059	mm	11qE2
A_55_P2402434	1110015O18Rik	Mus musculus RIKEN cDNA 11	105.36202	2.530383	P	A	-7.330939	0.0240161	NR_045272	NR_045272	NR_045272	NR_045272	Mm.75172	100503859		TC1774892		chr3:4814566-4814625	mm	3qA1
A_55_P1961616		Mouse anti-DNA antibody kap	108.7768	2.422892	P	A	-7.479041	0.022274	L08209		L08209	L08209	Mm.424513			TC1706491		chr6:68784899-68784842	mm	6qC1
A_51_P428372	Ppbp	Mus musculus pro-platelet bas	2070.6165	2.8200855	P	A	-18.31268	0.001362	NM_023785	NM_023785	NM_023785	NM_023785	Mm.293614	57349	ENSMUST00000031319	TC1580384	GO:0003674(molecular function)|GO:	chr5:91198970-91199029	mm	5qE1
