# Supplementary material for: Ubiquitin-dependent proteolysis of CXCL7 leads to posterior longitudinal ligament ossification
Source: PLoS One. 2018 May 21;13(5):e0196204. doi: 10.1371/journal.pone.0196204 (PMC5962073; doi:10.1371/journal.pone.0196204)
Supplement: S2 Dataset — (PDF) [file pone.0196204.s012.pdf]

Supporting Information

Ubiquitin-dependent proteolysis of CXCL7 leads to posterior longitudinal ligament ossification

Michiyo Tsuru, Atsushi Ono, Hideaki Umeyama, Masahiro Takeuchi and Kensei Nagata

Supplementary dataset

Microarray data are available in the Gene Expression Omnibus database (http://www.ncbi.nlm.nih.gov/geo/) under the accession number GSE57592.

Supplementary Dataset GSE57592

systematic_name	Ctrl_signal	OPLL_signal	Ctrl_flag	OPLL_flag	compare1_ratio	active_sequence	chr	mirbase_accession_No	start	stop	strand
hsv1-miR-H6-3p	0.1	12.60343	Not-Detect	Detected	126.0343	GGGATGGAAGGACGG		MIMAT0008404			
hsa-miR-199b-5p	0.1	12.25099033	Not-Detect	Detected	122.5099	GAACAGATAGTCTAAACACTGG	chr9	MIMAT0000263	130046868	130046847	+
hsa-miR-139-5p	0.1	10.30805333	Not-Detect	Detected	103.08053	CTGGAGACACGTGCAC	chr11	MIMAT0000250	72326134	72326120	+
hsa-miR-4317	0.1	8.879873333	Not-Detect	Detected	88.798733	AAACTCCCTGGCAATG	chr18	MIMAT0016872	6374386	6374372	+
hsv1-miR-H18	0.1	7.9926	Not-Detect	Detected	79.926	GGTCCCGGCGTC		MIMAT0014696			
hsa-miR-1271	0.1	6.33481	Not-Detect	Detected	63.3481	TGAGTGCTTGCTAGGTG	chr5	MIMAT0005796	175794969	175794984	−
hsa-miR-1246	0.1	5.785483333	Not-Detect	Detected	57.854833	CCTGCTCCAAAAATCC	chr2	MIMAT0005898	177465736	177465722	+
hsa-miR-1281	0.1	5.652183333	Not-Detect	Detected	56.521833	GGGAGAGGAGGAGG	chr22	MIMAT0005939	41488553	41488565	−
hsa-miR-191*	0.1	5.565563333	Not-Detect	Detected	55.655633	GGGGACGAAATCCAAG	chr3	MIMAT0001618	49058129	49058115	+
hsa-miR-1234	0.1	5.495356667	Not-Detect	Detected	54.953567	GTGGGGTGGGT	chr8	MIMAT0005589	145625557	145625548	+
hsa-miR-769-5p	0.1	5.4569	Not-Detect	Detected	54.569	AGCTCAGAACCCAGAGGTC	chr19	MIMAT0003886	46522223	46522240	−
hsa-miR-1825	0.1	5.331313333	Not-Detect	Detected	53.313133	GGAGAGGAGGGCAC	chr20	MIMAT0006765	30825638	30825650	−
hsa-miR-148b*	0.1	4.643136667	Not-Detect	Detected	46.431367	GCCTGAGTGTATAACAGAA	chr12	MIMAT0004699	54731028	54731045	−
hsa-miR-624*	0.1	4.477343333	Not-Detect	Detected	44.773433	TGAACACAAGGTACTGGTAC	chr14	MIMAT0003293	30553639	30553620	+
hsa-miR-491-5p	0.1	4.39433	Not-Detect	Detected	43.9433	CCTCATGGAAGGGTTC	chr9	MIMAT0002807	20716126	20716140	−
hsa-miR-335*	0.1	4.22969	Not-Detect	Detected	42.2969	GGTCAGGAGCAATAATGAAAAA	chr7	MIMAT0004703	130136004	130136024	−
hsa-miR-625*	0.1	4.080473333	Not-Detect	Detected	40.804733	TGAGGGGGAAAGTTCTATA	chr14	MIMAT0004808	65937875	65937892	−
hsa-miR-550a	0.1	4.019856667	Not-Detect	Detected	40.198567	GGGCTCTTACTCCCT	chr7	MIMAT0004800	30329439	30329453	−
hsa-miR-933	0.1	3.882966667	Not-Detect	Detected	38.829667	GGGAGAGGTCTCCCT	chr2	MIMAT0004976	176032428	176032415	+
hsa-miR-3653	16.477783	114.2250313	Detected	Detected	6.9320629	CTTCAGTCAACTTCTTAG	chr22	MIMAT0018073	29729238	29729222	+
hsa-miR-338-3p	3.01419	16.72975	Detected	Detected	5.5503303	CAACAAAATCACTGATGCTGG	chr17	MIMAT0000763	79099745	79099726	+
hsa-miR-146b-5p	2.7749067	15.37398	Detected	Detected	5.5403593	AGCCTATGGAATTCAGTTC	chr10	MIMAT0002809	104186270	104186288	−
hsa-miR-155	5.5655633	27.20819	Detected	Detected	4.8886678	ACCCCTATCACGATTAG	chr21	MIMAT0000646	26946302	26946317	−
hsa-miR-1228	2.7994167	13.44440333	Detected	Detected	4.8025732	GGGGGGCGAGG	chr12	MIMAT0005583	57588348	57588357	−
hsa-miR-181a-2*	1.67184	7.616886667	Detected	Detected	4.5559902	GGTACAGTCAACGGTCA	chr9	MIMAT0004558	127454803	127454818	−
hsa-miR-629*	2.3175867	9.772357	Detected	Detected	4.2166091	GCTGGGCTTACGTTGG	chr15	MIMAT0003298	68158846	68158831	+
hsa-miR-26b*	1.79513	7.2449	Detected	Detected	4.0358637	GAGCCAAGTAATGGAGAACA	chr2	MIMAT0004500	219267418	219267436	−
hsa-miR-30e*	16.874763	67.177034	Detected	Detected	3.9809171	GCTGTAAACATCCGACTG	chr1	MIMAT0000693	40992676	40992693	−
hsa-miR-142-3p	371.28853	1380.493033	Detected	Detected	3.7181138	TCCATAAAGTAGGAAACACTACA	chr17	MIMAT0000434	56408666	56408645	+
hsa-miR-22*	3.0598633	11.31408	Detected	Detected	3.6975769	TAAAGCTTGCCACTGAAG	chr17	MIMAT0004495	1617232	1617216	+
hsa-miR-99a	3.04066	10.99070967	Detected	Detected	3.6145803	CACAAGATCGGATCTACGG	chr21	MIMAT0000097	17911425	17911442	−
hsa-miR-3651	15.24837	53.38944	Detected	Detected	3.5013211	TCATGTACCAGCGACC	chr9	MIMAT0018071	95054826	95054812	+
hsa-miR-7-1*	5.8974	20.64399667	Detected	Detected	3.5005251	TATGGCAGACTGTGATTTG	chr9	MIMAT0004553	86584749	86584732	+
hsa-miR-15b*	24.234596	71.7979	Detected	Detected	2.96262	TAGAGCAGCAAATAATGATTCG	chr3	MIMAT0004586	160122434	160122454	−
hsa-miR-340*	34.922714	103.4310017	Detected	Detected	2.9617114	GCTATAAAGTAACTGAGACGG	chr5	MIMAT0000750	179442381	179442362	+
hsa-miR-30a	16.974657	48.653809	Detected	Detected	2.8662618	CTTCCAGTCGAGGATG	chr6	MIMAT0000087	72113280	72113265	+
hsa-miR-125a-5p	13.31482	37.52035	Detected	Detected	2.817939	TCACAGGTTAAAGGGTCTC	chr19	MIMAT0000443	52196527	52196544	−
hsa-miR-361-5p	45.151885	125.4357983	Detected	Detected	2.7780855	GTACCCCTGGAGATTC	chrX	MIMAT0000703	85045323	85045308	+
hsa-miR-132	6.4784733	17.90044667	Detected	Detected	2.7630656	CGACCATGGCTGTAGA	chr17	MIMAT0000426	1953281	1953267	+
hsv2-miR-H24	4.4218033	11.58098	Detected	Detected	2.6190627	AGGGCGGGGGGA		MIMAT0014707			
hsa-miR-363	420.25467	1085.326667	Detected	Detected	2.5825452	TACAGATGGATACCGTGCA	chrX	MIMAT0000707	133303478	133303461	+
hsa-miR-378*	24.79881	63.69096667	Detected	Detected	2.5683074	ACACAGGACCTGGAGTCA	chr5	MIMAT0000731	149092589	149092606	−
hsa-miR-27b	4.4773433	11.22994	Detected	Detected	2.5081704	GCAGAACTTAGCCACTGT	chr9	MIMAT0000419	97847791	97847807	−
hsa-miR-151-3p	99.280733	246.1382033	Detected	Detected	2.4792142	CCTCAAGGAGCTTCAGT	chr8	MIMAT0000757	141742729	141742713	+
hsa-miR-28-5p	14.249183	33.960081	Detected	Detected	2.3833002	CTCAATAGACTGTGAGCTCC	chr3	MIMAT0000085	188406584	188406603	−
hsa-miR-505	26.868797	63.20132	Detected	Detected	2.3522199	AGGAAACCAGCAAGTGTTG	chrX	MIMAT0002876	139006378	139006361	+
hsa-miR-191	10.469624	24.234596	Detected	Detected	2.3147533	CAGCTGCTTTTGGGA	chr3	MIMAT0000440	49058088	49058075	+
hsa-miR-502-5p	5.9682733	13.79809683	Detected	Detected	2.3119077	TAGCACCCAGATAGCAAGG	chrX	MIMAT0002873	49665963	49665981	−
hsa-miR-3200-5p	10.308053	23.69670933	Detected	Detected	2.298854	ACCTTGTGCGCCTTC	chr22	MIMAT0017392	31127564	31127577	−
hsa-miR-27a	27.20819	61.07559333	Detected	Detected	2.2447503	GCGGAACTTAGCCACTG	chr19	MIMAT0000084	13947324	13947309	+
hsa-miR-29c*	68.557165	153.3149667	Detected	Detected	2.2363084	GAACACCAGGAGAAATCGGT	chr1	MIMAT0004673	207975233	207975215	+
hsa-let-7d*	7.6168867	16.87476333	Detected	Detected	2.215441	AGAAAGGCAGCAGGTCGT	chr9	MIMAT0004484	96941182	96941198	−
hsa-miR-146a	23.31294	48.44695267	Detected	Detected	2.0781142	AACCCATGGAATTCAGTTC	chr5	MIMAT0000449	159912383	159912400	−
hsa-miR-145	42.065686	86.27669867	Detected	Detected	2.0509994	AGGGATTCCTGGGAAAAC	chr5	MIMAT0000437	148810230	148810246	−
hsa-miR-365	49.603074	99.28073333	Detected	Detected	2.0015036	ATAAGGATTTTTAGGGGCATTA	chr16	MIMAT0000710	14403197	14403218	−
hsa-miR-423-3p	11.928523	23.31294033	Detected	Detected	1.9543861	ACTGAGGGGCCTCA	chr17	MIMAT0001340	28444159	28444171	−
hsa-miR-151-5p	809.65432	1571.288967	Detected	Detected	1.9406911	ACTAGACTGTGAGCTCC	chr8	MIMAT0004697	141742693	141742678	+
hsa-miR-342-3p	446.77447	857.4416833	Detected	Detected	1.9191823	ACGGGTGCGATTTCTG	chr14	MIMAT0000753	100576060	100576074	−
hsa-miR-4284	123.92654	235.4955967	Detected	Detected	1.9002839	ATGGGGTGATGTGAGC	chr7	MIMAT0016915	73125660	73125674	−
hsa-miR-4323	8.2505833	15.51047667	Detected	Detected	1.8799249	TCTGAGGCTGTGGGG	chr19	MIMAT0016875	42637655	42637642	+
hsa-miR-1275	4.39433	8.250583333	Detected	Detected	1.8775521	GACAGCCTCTCCCC	chr6	MIMAT0005929	33967782	33967770	+
hsa-miR-23a	967.55235	1812.929333	Detected	Detected	1.8737274	GGAAATCCCTGGCAATGT	chr19	MIMAT0000078	13947465	13947449	+
hsa-miR-454*	10.56302	19.7279	Detected	Detected	1.8676382	GCAGAGACAATATTGATAGGGT	chr17	MIMAT0003884	54569945	54569924	+
hsa-miR-195	10.99071	20.18733	Detected	Detected	1.8367631	GCCAATATTTCTGTGCTGC	chr17	MIMAT0000461	6920968	6920951	+
hsa-miR-128	126.01693	230.65727	Detected	Detected	1.8303673	AAAGAGACCGGTTCACTGT	chr2	MIMAT0000424	136423018	136423036	−
hsa-miR-328	16.057947	29.36293333	Detected	Detected	1.8285609	ACGGAAGGGCAGAGAGGG	chr16	MIMAT0000752	67236292	67236276	+
hsa-miR-874	2.85254	5.18813	Detected	Detected	1.8187755	TCGGTCCCTCGGG	chr5	MIMAT0004911	136983328	136983317	+
hsa-miR-454	12.60343	22.91401333	Detected	Detected	1.8180776	ACCCTATAAGCAATATTGCAC	chr17	MIMAT0003885	57215204	57215185	+
hsa-miR-1285	8.8798733	16.05794667	Detected	Detected	1.8083531	AGGTCTCACTTTGTTGC	chr2	MIMAT0005876	70480122	70480106	+
hsa-miR-186	286.36663	516.42115	Detected	Detected	1.8033566	AGCCCAAAAGGAGAATTCTTT	chr1	MIMAT0000456	71533349	71533330	+
hsa-miR-374b	27.771433	50.042302	Detected	Detected	1.8019344	CACTTAGCAGGTTGTATTA	chrX	MIMAT0004955	73438413	73438396	+
hsa-miR-24	190.19717	338.9052667	Detected	Detected	1.7818629	CTGTTCCTGCTGAACTGA	chr9	MIMAT0000080	97848350	97848367	−
hsa-miR-26b	106.79123	190.1971667	Detected	Detected	1.7810185	ACCTATCCTGAATTACTTG	chr2	MIMAT0000083	219267382	219267400	−
hsa-miR-10a	4.93335	8.669816667	Detected	Detected	1.7573893	CACAAATTCGGATCTACAGGG	chr17	MIMAT0000253	46657243	46657224	+
hsa-miR-130b	63.690967	106.7912347	Detected	Detected	1.6767093	ATGCCCTTTCATCATTGC	chr22	MIMAT0000691	22007648	22007664	−
hsa-miR-362-3p	4.6431367	7.778796667	Detected	Detected	1.6753323	TGAATCCTTGAATAGGTGTG	chrX	MIMAT0004683	49773616	49773634	−
hsa-miR-130b*	6.1877233	10.11970333	Detected	Detected	1.6354486	GTAGTGCAACAGGGAAAGA	chr22	MIMAT0004680	22007608	22007625	−
hsa-miR-98	8.8144533	14.24918333	Detected	Detected	1.6165703	AACAATACAACTTACTACCTC	chrX	MIMAT0000096	53583226	53583207	+
hsa-miR-500a	17.188814	27.77143333	Detected	Detected	1.615669	TCTCACCCAGGTAG	chrX	MIMAT0004773	49773060	49773073	−
hsa-miR-940	16.72975	26.868797	Detected	Detected	1.6060489	GGGGAGCGGGGG	chr16	MIMAT0004983	2321817	2321827	−
hsa-miR-342-5p	32.2485	51.06933333	Detected	Detected	1.5836189	TCAATCACAGATAGCACCC	chr14	MIMAT0004694	100576013	100576030	−
hsa-miR-378	10.119703	15.89344	Detected	Detected	1.5705441	CCTTCTGACTCCA	chr5	MIMAT0000732	149112439	149112450	−
hsa-miR-130a	93.506699	144.68593	Detected	Detected	1.5473322	ATGCCCTTTTAACATTGCA	chr11	MIMAT0000425	57408729	57408746	−
hsa-miR-3200-3p	3.8829667	5.968273333	Detected	Detected	1.5370395	CAGACCTGAGTAGCGC	chr22	MIMAT0015085	31127603	31127618	−
hsa-miR-942	36.94219	56.51066567	Detected	Detected	1.5297054	CACATGGCAAAACAGAGAA	chr1	MIMAT0004985	117637280	117637298	−
hsa-miR-766	31.200547	47.706554	Detected	Detected	1.5290294	GCTGAGGCTGTGGGGCT	chrX	MIMAT0003888	118780786	118780771	+
hsa-miR-501-5p	30.908047	46.83227333	Detected	Detected	1.515213	TCTCACCCAGGGACAAAG	chrX	MIMAT0002872	49661087	49661104	−
hsa-miR-1280	5.9452333	8.99957	Detected	Detected	1.5137455	GGGTGGCAGCGG	chr3	MIMAT0005946	128081018	128081028	−
hsa-miR-505*	39.394613	59.16407433	Detected	Detected	1.5018316	ACATCAATACTTCCTGGCTC	chrX	MIMAT0004776	139006342	139006324	+
hsa-miR-93*	48.446953	72.13608133	Detected	Detected	1.4889705	CGGGAAGTGCTAGCTC	chr7	MIMAT0004509	99691461	99691447	+
hsa-miR-574-3p	29.362933	43.390201	Detected	Detected	1.4777202	TGTGGGTGTGTGCATG	chr4	MIMAT0003239	38869720	38869734	−
hsa-miR-23b	144.68593	212.3189333	Detected	Detected	1.467447	GGTAATCCCTGGCAATG	chr9	MIMAT0000418	97847552	97847567	−
hsa-miR-215	235.4956	343.9077833	Detected	Detected	1.4603576	GTCTGTCAATTCATAGGTCAT	chr1	MIMAT0000272	220291241	220291222	+
hsa-miR-1260b	19.397323	28.1485	Detected	Detected	1.4511539	ATGGTGGCAGTGGTG	chr11	MIMAT0015041	96074616	96074629	−
hsa-miR-324-5p	81.94324	118.1663983	Detected	Detected	1.4420518	ACACCAATGCCCTAGGG	chr17	MIMAT0000761	7126653	7126638	+
hsa-miR-500b	9.4125563	13.31482	Detected	Detected	1.4145806	ACCCAGGTAGCAAGGA	chrX	MIMAT0016925	49775293	49775307	−
hsa-miR-30b	2930.1483	4137.064333	Detected	Detected	1.4118959	AGCTGAGTGTAGGATGTT	chr8	MIMAT0000420	135812800	135812784	+
hsa-miR-326	36.81878	51.55732	Detected	Detected	1.4002995	CTGGAGGAAGGGCCCAG	chr11	MIMAT0000756	75046214	75046199	+
hsa-miR-150	2352.7873	3293.595667	Detected	Detected	1.3998697	CACTGGTACAAGGGTTGG	chr19	MIMAT0000451	50004078	50004062	+
hsa-miR-222	28.1485	38.84292733	Detected	Detected	1.3799289	ACCCAGTAGCCAG	chrX	MIMAT0000279	45606509	45606498	+
hsa-miR-4318	22.66427	30.90804667	Detected	Detected	1.3637345	AGCATGTACCCACAGTG	chr18	MIMAT0016869	35237153	35237168	−
hsa-miR-199a-5p	67.177034	91.58993333	Detected	Detected	1.3634114	GAACAGGTAGTCTGAACAC	chr1	MIMAT0000231	170380350	170380332	+
hsa-miR-1260	75.2672	100.583275	Detected	Detected	1.3363494	TGGTGGCAGAGGTGG	chr14	MIMAT0005911	77732578	77732591	−
hsa-miR-296-5p	8.99957	11.92852317	Detected	Detected	1.3254548	ACAGGATTGAGGGGG	chr20	MIMAT0000690	56826098	56826083	+
hsa-miR-664	59.164074	77.85423333	Detected	Detected	1.3159039	TGTAGGCTGGGGATAAA	chr1	MIMAT0005949	220373950	220373935	+
hsa-miR-182	100.58328	130.7217333	Detected	Detected	1.2996369	AGTGTGAGTTCTACCAT	chr7	MIMAT0000259	129410268	129410253	+
hsa-miR-144*	41.726493	54.01116233	Detected	Detected	1.2944093	CTTACAGTATATGATGATATCC	chr17	MIMAT0004600	27188586	27188566	+
hsa-miR-625	62.032868	80.09745	Detected	Detected	1.2912099	GGACTATAGAACTTTCCCC	chr14	MIMAT0003294	65937837	65937854	−

hsa-miR-26a	489.418	628.60168	Detected	Detected	1.2843861	AGCCTATCCTGGATT	chr3	MIMAT0000082	38010911	38010925	-
hsa-miR-3198	11.58098	14.69671333	Detected	Detected	1.2690388	TCTCCATTCCCCAGG	chr22	MIMAT0015083	18247015	18247002	+
hsa-let-7d	611.18266	764.3796333	Detected	Detected	1.2506566	AACTATGCAACCTACTACC	chr9	MIMAT0000065	96941127	96941144	-
hsa-miR-720	579.402	716.3933333	Detected	Detected	1.2364357	TGGAGGCCCCAGC	chr3	MIMAT0005954	164059160	164059171	-
hsa-miR-362-5p	61.075593	75.2672	Detected	Detected	1.2323613	ACTCACACCTAGGTTCC	chrX	MIMAT0000705	49660323	49660339	-
hsa-miR-192	400.72374	489.418	Detected	Detected	1.2213352	GGCTGTCAATTCATAGGTC	chr11	MIMAT0000222	64658652	64658635	+
hsa-let-7f	838.78233	1011.26462	Detected	Detected	1.2056341	AACTATACAATCTACTACCTC	chr9	MIMAT0000067	96938636	96938656	-
hsa-miR-1305	12.739113	15.24837	Detected	Detected	1.1969726	TCTCTCCCATTAGAGTTGA	chr4	MIMAT0005893	183090500	183090517	-
hsa-miR-148b	45.570757	54.53382567	Detected	Detected	1.1966847	ACAAAGTTCTGTGATGCAC	chr12	MIMAT0000759	54731066	54731083	-
hsa-miR-185	1571.289	1875.6813	Detected	Detected	1.1937214	TCAGGAACTGCCTTTCT	chr22	MIMAT0000455	20020682	20020697	-
hsa-let-7a	1812.9293	2150.648333	Detected	Detected	1.1862836	AACTATACAACCTACTACCT	chr9	MIMAT0000062	96938246	96938265	-
hsa-miR-532-3p	338.90527	400.7237367	Detected	Detected	1.1824063	TGCAAGCCTTGGGTG	chrX	MIMAT0004780	49767818	49767831	-
hsa-miR-331-3p	2150.6483	2539.366	Detected	Detected	1.1807444	TTCTAGGATAGGCCCAGGG	chr12	MIMAT0000760	94226389	94226407	-
hsa-miR-361-3p	153.31497	177.9172667	Detected	Detected	1.160469	AAATCAGAATCACACCTGGG	chrX	MIMAT0004682	85158707	85158689	+
hsa-miR-140-5p	14.696713	16.9746565	Detected	Detected	1.1549968	CTACCATAGGGTAAAACCACT	chr16	MIMAT0000431	68524508	68524528	-
hsa-miR-30c	2671.0677	3077.077333	Detected	Detected	1.1520028	GCTGAGAGTGTAGGATGT	chr1	MIMAT0000244	41222977	41222994	-
hsa-miR-19a	118.1664	134.6015667	Detected	Detected	1.139085	TCAGTTTTGCATAGATTTGCA	chr13	MIMAT0000073	92003196	92003215	-
hsa-miR-194	264.92796	300.18206	Detected	Detected	1.1330705	TCCACATGGAGTTGCT	chr1	MIMAT0000460	220291534	220291519	+
hsa-miR-197	125.4358	141.5951333	Detected	Detected	1.1288255	GCTGGGTGGAGAAGGTG	chr1	MIMAT0000227	110141568	110141583	-
hsa-miR-374a	15.37398	17.1888135	Detected	Detected	1.1180458	CACTTATCAGGTTGTATTATAA	chrX	MIMAT0000727	73423878	73423857	+
hsa-miR-1202	21.454293	23.879626	Detected	Detected	1.1130465	CTCCCCCACTGCA	chr6	MIMAT0005865	156267952	156267963	-
hsa-miR-30e	97.500634	107.743032	Detected	Detected	1.1050496	CTTCCAGTCAAGGATGT	chr1	MIMAT0000692	41220049	41220064	-
hsa-miR-484	902.09865	987.9723333	Detected	Detected	1.0951932	ATCGGGAGGGGACTGA	chr16	MIMAT0002174	15737165	15737179	-
hsa-miR-4286	63.20132	68.55716533	Detected	Detected	1.0847426	GGTACCAGGAGTGGG	chr8	MIMAT0016916	10524501	10524514	-
hsa-miR-501-3p	22.914013	24.79881	Detected	Detected	1.0822552	AGAATCCTTGCCCGGG	chrX	MIMAT0004774	49774387	49774401	-
hsa-let-7g	343.90778	371.2885333	Detected	Detected	1.0796165	AACTGTACAAACTACTACCTC	chr3	MIMAT0000414	52302319	52302300	+
hsa-miR-21	246.1382	264.9279633	Detected	Detected	1.0763383	TCAACATCAGTCTGATAAGC	chr17	MIMAT0000076	57918637	57918655	-
hsa-miR-1274a	7.1139533	7.583083	Detected	Detected	1.065945	TGGCGCCTGAACAG	chr5	MIMAT0005927	41475752	41475764	-
hsa-miR-30d	857.44168	902.0986533	Detected	Detected	1.0520816	CTTCCAGTCGGGGA	chr8	MIMAT0000245	135817145	135817133	+
hsa-miR-142-5p	33.960081	35.68391667	Detected	Detected	1.0507606	AGTAGTGCTTTCTACTTTA	chr17	MIMAT0000433	56408628	56408611	+
hsa-miR-762	5.18813	5.396133333	Detected	Detected	1.0400922	GCTCGGCCCCGG	chr16	MIMAT0010313	30905283	30905293	-
hsa-miR-550a*	278.04571	286.3666333	Detected	Detected	1.0299265	ATGTGCCTGAGGGAGTAA	chr7	MIMAT0003257	30295999	30296016	-
hsa-miR-4281	107.74303	110.889922	Detected	Detected	1.0292074	CCCCCCTCCCCG	chr5	MIMAT0016907	176056491	176056481	+
hsa-miR-19b	2539.366	2573.067	Detected	Detected	1.0132714	TCAGTTTTGCATGGATTTGC	chr13	MIMAT0000074	92003502	92003521	-
hsa-miR-744	25.641337	25.75154	Detected	Detected	1.0042979	TGCTGTTAGCCCTA	chr17	MIMAT0004945	11985235	11985247	-
hsa-let-7g*	0.1	0.1	Not-Detect	Not-Detect	1	GCAAGGCAGTGGCCT	chr3	MIMAT0004584	52302375	52302362	+
hsa-miR-486-3p	0.1	0.1	Not-Detect	Not-Detect	1	ATCCTGTACTGAGCTGC	chr8	MIMAT0004762	41518024	41518009	+
hsa-miR-30d*	0.1	0.1	Not-Detect	Not-Detect	1	GCAGCAAACATCTGACTGA	chr8	MIMAT0004551	135817185	135817168	+
hsa-miR-136*	0.1	0.1	Not-Detect	Not-Detect	1	AGACTCATTTGAGACGATGAT	chr14	MIMAT0004606	101351089	101351108	-
hsa-miR-572	0.1	0.1	Not-Detect	Not-Detect	1	TGGGCCACCGCCG	chr4	MIMAT0003237	11370519	11370530	-
hsa-miR-887	0.1	0.1	Not-Detect	Not-Detect	1	CCTCGGGATGGCGC	chr5	MIMAT0004951	15935347	15935359	-
hsa-miR-3935	0.1	0.1	Not-Detect	Not-Detect	1	GTGGCTGGTGCTCG	chr16	MIMAT0018350	56279498	56279510	-
hsa-miR-3140	0.1	0.1	Not-Detect	Not-Detect	1	ACTACCTGAATTCCCAAA	chr4	MIMAT0015008	153410559	153410543	+
hsa-miR-599	0.1	0.1	Not-Detect	Not-Detect	1	GTTTGATAAACTGACACAA	chr8	MIMAT0003267	100548943	100548926	+
hsa-miR-3620	0.1	0.1	Not-Detect	Not-Detect	1	CTGGGTGCGGGAT	chr1	MIMAT0018001	228285031	228285042	-
hsa-miR-943	0.1	0.1	Not-Detect	Not-Detect	1	CTGGAGGACGGCAA	chr4	MIMAT0004986	1988185	1988173	+
hsa-miR-2114	0.1	0.1	Not-Detect	Not-Detect	1	GACCGCTTCAAGGAA	chrX	MIMAT0011156	149396259	149396272	-
hsa-miR-4258	0.1	0.1	Not-Detect	Not-Detect	1	CCAAGGCGGTGGC	chr1	MIMAT0016879	154948184	154948195	-
hsa-miR-1247	0.1	0.1	Not-Detect	Not-Detect	1	TCCGGGGACGAACG	chr14	MIMAT0005899	102026684	102026672	+
hsa-miR-518c*	0.1	0.1	Not-Detect	Not-Detect	1	CAGAAAGTGCTTCCCTC	chr19	MIMAT0002847	54209525	54209541	-
hsa-miR-3170	0.1	0.1	Not-Detect	Not-Detect	1	ACTGTCTGTCTCAGAACC	chr13	MIMAT0015045	98860792	98860808	-
hsa-miR-3666	0.1	0.1	Not-Detect	Not-Detect	1	TCGGCATCTACACTTGC	chr7	MIMAT0018088	114293433	114293448	-
ebv-miR-BART19-5p	0.1	0.1	Not-Detect	Not-Detect	1	CATGTCATGTTTGCGGG		MIMAT0004836			
hsv1-miR-H7*	0.1	0.1	Not-Detect	Not-Detect	1	GAAGAGGGGTCGGG		MIMAT0012596			
hsa-miR-2277-5p	0.1	0.1	Not-Detect	Not-Detect	1	GACTGGCAGCGCT	chr5	MIMAT0017352	92956442	92956431	+
hsa-miR-429	0.1	0.1	Not-Detect	Not-Detect	1	ACGGTTTTACCAGACAGTA	chr1	MIMAT0001536	1104439	1104456	-
hsa-miR-127-5p	0.1	0.1	Not-Detect	Not-Detect	1	ATCAGAGCCCTCTGA	chr14	MIMAT0004604	101349346	101349359	-
hsa-miR-541*	0.1	0.1	Not-Detect	Not-Detect	1	AGTGGGACCGACAGCAG	chr14	MIMAT0004919	101530850	101530865	-
hsa-miR-3689a-5p	0.1	0.1	Not-Detect	Not-Detect	1	TCCCAGGAACCATGAT	chr9	MIMAT0018117	137741363	137741348	+
hcmv-miR-UL22A*	0.1	0.1	Not-Detect	Not-Detect	1	CTACAAACTAGCATTCTGGT		MIMAT0001575			
kshv-miR-K12-12	0.1	0.1	Not-Detect	Not-Detect	1	TCAACCAGGGCACC		MIMAT0015238			
hsa-miR-299-5p	0.1	0.1	Not-Detect	Not-Detect	1	ATGTATGTGGGACGGTAAAC	chr14	MIMAT0002890	101490140	101490158	-
hsa-miR-1293	0.1	0.1	Not-Detect	Not-Detect	1	GCACAAATCTCCAGACC	chr12	MIMAT0005883	50627955	50627940	+
hsa-miR-548u	0.1	0.1	Not-Detect	Not-Detect	1	CGCAAAAGTAATTGCAG	chr6	MIMAT0015013	57254985	57255000	-
hsa-miR-7-2*	0.1	0.1	Not-Detect	Not-Detect	1	TTAGGTAGACTGGGATTTG	chr15	MIMAT0004554	89155131	89155148	-
hsa-miR-379	0.1	0.1	Not-Detect	Not-Detect	1	CCTACGTTCCATAGTC	chr14	MIMAT0000733	101488414	101488428	-
hsa-miR-744*	0.1	0.1	Not-Detect	Not-Detect	1	AGGTTGAGGTTAGTGGCA	chr17	MIMAT0004946	11985288	11985304	-
hsa-miR-384	0.1	0.1	Not-Detect	Not-Detect	1	TATGAACAATTTCTAGGAAT	chrX	MIMAT0001075	76139773	76139755	+
hsa-miR-101*	0.1	0.1	Not-Detect	Not-Detect	1	AGCATCAGCACTGTGATA	chr1	MIMAT0004513	65524148	65524132	+
hsa-miR-490-3p	0.1	0.1	Not-Detect	Not-Detect	1	CAGCATGGAGTCCTCCA	chr7	MIMAT0002806	136238534	136238550	-
hsa-miR-3117	0.1	0.1	Not-Detect	Not-Detect	1	CTGGCACTATATGAGTCC	chr1	MIMAT0014979	67094172	67094188	-
hsa-miR-577	0.1	0.1	Not-Detect	Not-Detect	1	CAGGTACCAATATTTTATCTA	chr4	MIMAT0003242	115577931	115577950	-
hsa-miR-34c-3p	0.1	0.1	Not-Detect	Not-Detect	1	CCTGGCCGTGTGG	chr11	MIMAT0004677	111384219	111384230	-
hsa-miR-892a	0.1	0.1	Not-Detect	Not-Detect	1	CTACGCAGAAAGGACACAGT	chrX	MIMAT0004907	145078251	145078233	+
hsa-miR-200b	0.1	0.1	Not-Detect	Not-Detect	1	TCATCATTACCAGGCAG	chr1	MIMAT0000318	1102546	1102561	-
hsa-miR-3942	0.1	0.1	Not-Detect	Not-Detect	1	ATTTCAGGTAACAGTATTGC	chr15	MIMAT0018358	35664500	35664482	+
hsa-miR-1208	0.1	0.1	Not-Detect	Not-Detect	1	TCCGCCTGTCTGAAC	chr8	MIMAT0005873	129162379	129162392	-
hsa-miR-509-5p	0.1	0.1	Not-Detect	Not-Detect	1	TGATTGCCACTGTCTGC	chrX	MIMAT0004779	146340317	146340301	+
hsa-miR-3146	0.1	0.1	Not-Detect	Not-Detect	1	CCATTCTTTCTATCCTAGC	chr7	MIMAT0015018	19745051	19745034	+
hsa-miR-152	0.1	0.1	Not-Detect	Not-Detect	1	CCAAGTTCTGTCATGC	chr17	MIMAT0000438	46114600	46114586	+
hsa-miR-606	0.1	0.1	Not-Detect	Not-Detect	1	ATCTTTGATTTTCAGTAGTTT	chr10	MIMAT0003274	77312277	77312296	-
hsa-miR-363*	0.1	0.1	Not-Detect	Not-Detect	1	AAATTGCATCGTGATCCAC	chrX	MIMAT0003385	133303435	133303418	+
hsa-miR-99a*	0.1	0.1	Not-Detect	Not-Detect	1	CAGACCCATAGAAGCGA	chr21	MIMAT0004511	17911464	17911479	-
hsa-miR-212	0.1	0.1	Not-Detect	Not-Detect	1	GGCCGTGACTGGAGAC	chr17	MIMAT0000269	1953655	1953641	+
hsa-miR-4265	0.1	0.1	Not-Detect	Not-Detect	1	CCCAGAGCTGAGCC	chr2	MIMAT0016891	109758034	109758022	+
hsa-miR-1254	0.1	0.1	Not-Detect	Not-Detect	1	ACTGCAGGCTCCAGC	chr10	MIMAT0005905	70519102	70519115	-
hsa-miR-519c-3p	0.1	0.1	Not-Detect	Not-Detect	1	ATCCTCTAAAAAGATGCACTT	chr19	MIMAT0002832	54189777	54189797	-
hsa-miR-3178	0.1	0.1	Not-Detect	Not-Detect	1	CGATCCGGCCGC	chr16	MIMAT0015055	2581949	2581939	+
hsa-miR-632	0.1	0.1	Not-Detect	Not-Detect	1	TCCCACAGGAAGCAGAC	chr17	MIMAT0003302	30677191	30677206	-
hsa-miR-3670	0.1	0.1	Not-Detect	Not-Detect	1	TAGAGAAGGACAGCTGTG	chr16	MIMAT0018093	15001619	15001636	-
ebv-miR-BART22	0.1	0.1	Not-Detect	Not-Detect	1	ACTACTAGACCATGACTTTG		MIMAT0010132			
hsv2-miR-H2	0.1	0.1	Not-Detect	Not-Detect	1	TCGCGCATGACCC		MIMAT0010205			
hsa-miR-23b*	0.1	0.1	Not-Detect	Not-Detect	1	AAATCAGCATGCCAGGAACC	chr9	MIMAT0004587	97847512	97847530	-
hsa-miR-4296	0.1	0.1	Not-Detect	Not-Detect	1	TGAGCCTGAGCCCA	chr10	MIMAT0016845	126721378	126721366	+
hsa-miR-1273e	0.1	0.1	Not-Detect	Not-Detect	1	TCCACTTCCTGGGTTC		MIMAT0018079			
hsa-miR-545*	0.1	0.1	Not-Detect	Not-Detect	1	TCATCTAATAAACATTTACTGA	chrX	MIMAT0004785	73506984	73506964	+
hsa-miR-3692*	0.1	0.1	Not-Detect	Not-Detect	1	CAGTATCCACTCCTGAC	chr6	MIMAT0018121	157950177	157950192	-
hcmv-miR-US25-2-3p	0.1	0.1	Not-Detect	Not-Detect	1	CCGCGGGAGCTC		MIMAT0001583			
hsa-miR-4329	0.1	0.1	Not-Detect	Not-Detect	1	GTGGAACTAGGGTCTC	chrX	MIMAT0016923	112024006	112023992	+
kshv-miR-K12-4-5p	0.1	0.1	Not-Detect	Not-Detect	1	CCTAGAGTACTGCGGTT		MIMAT0002191			
hsa-miR-1301	0.1	0.1	Not-Detect	Not-Detect	1	GAAGTCACTCCCAGG	chr2	MIMAT0005797	25551579	25551566	+
hsa-miR-765	0.1	0.1	Not-Detect	Not-Detect	1	CATCACCTTCCTTCTCCT	chr1	MIMAT0003945	156906011	156905995	+
hsa-miR-193b	0.1	0.1	Not-Detect	Not-Detect	1	AGCGGGACTTTGAGGG	chr16	MIMAT0002819	14397881	14397895	-
hsa-miR-3913	0.1	0.1	Not-Detect	Not-Detect	1	AGACATCAAGATCAGTCC	chr12	MIMAT0018187	69978528	69978545	-
hsa-miR-3124	0.1	0.1	Not-Detect	Not-Detect	1	GACTTTGCCTTCGCC	chr1	MIMAT0014986	249120589	249120602	-
hsa-miR-143*	0.1	0.1	Not-Detect	Not-Detect	1	ACCAGAGATGCAGCACT	chr5	MIMAT0004599	148808513	148808528	-
hsa-miR-583	0.1	0.1	Not-Detect	Not-Detect	1	GTAATGGGACCTTCCTCT	chr5	MIMAT0003248	95414861	95414877	-
hsa-miR-3609	0.1	0.1	Not-Detect	Not-Detect	1	CAGCCAGTATTACTCATCA	chr7	MIMAT0017986	98479329	98479346	-
hsa-miR-924	0.1	0.1	Not-Detect	Not-Detect	1	GCAAGACATCACAAGACTCT	chr18	MIMAT0004974	37202109	37202091	+
hsa-miR-204	0.1	0.1	Not-Detect	Not-Detect	1	AGGCATAGGATGACAAAGGG	chr9	MIMAT0000265	73424944	73424926	+

hsa-miR-411	0.1	0.1	Not-Detect	Not-Detect	1	CGTACGCTATACGGTCTA	chr14	MIMAT0003329	101489681	101489697	−
hsa-miR-1226	0.1	0.1	Not-Detect	Not-Detect	1	CTAGGGAACACAGGG	chr3	MIMAT0005577	47891106	47891119	−
hsa-miR-513b	0.1	0.1	Not-Detect	Not-Detect	1	ATAAATGACACCTCCTTGT	chrX	MIMAT0005788	146280596	146280579	+
hsa-miR-3152	0.1	0.1	Not-Detect	Not-Detect	1	TTATTGCCCCTATTCTAACA	chr9	MIMAT0015025	18573351	18573369	−
hsa-miR-613	0.1	0.1	Not-Detect	Not-Detect	1	GGCAAAGAAGGAACAT	chr12	MIMAT0003281	12917648	12917662	−
hsa-miR-365*	0.1	0.1	Not-Detect	Not-Detect	1	ACAGCTGCCCCTGA	chr17	MIMAT0009199	29902467	29902479	−
ebv-miR-BART1-3p	0.1	0.1	Not-Detect	Not-Detect	1	GACATAGTGGATAGCGGTG		MIMAT0003390			
hsv1-miR-H13	0.1	0.1	Not-Detect	Not-Detect	1	CCAGTGCTCGCACT		MIMAT0014690			
hsa-miR-218	0.1	0.1	Not-Detect	Not-Detect	1	ACATGGTTAGATCAAGCACA	chr4	MIMAT0000275	20529923	20529942	−
hsa-miR-4272	0.1	0.1	Not-Detect	Not-Detect	1	ACAATCACTAGTTGAATG	chr3	MIMAT0016902	67275925	67275941	−
hsa-miR-520c-3p	0.1	0.1	Not-Detect	Not-Detect	1	ACCCTCTAAAAGGAAGCACT	chr19	MIMAT0002846	54210763	54210781	−
hsa-miR-3183	0.1	0.1	Not-Detect	Not-Detect	1	TCCGAGCGACTCCG	chr17	MIMAT0015063	925746	925734	+
hsa-miR-639	0.1	0.1	Not-Detect	Not-Detect	1	ACAGCGCTCGCAACCGC	chr19	MIMAT0003309	14640422	14640437	−
hsa-miR-3676	0.1	0.1	Not-Detect	Not-Detect	1	AAAGCGTGGGGGAA	chr17	MIMAT0018100	8090559	8090571	−
ebv-miR-BART6-3p	0.1	0.1	Not-Detect	Not-Detect	1	TCTAAGGCTAGTCCGAT		MIMAT0003415			
hsv2-miR-H25	0.1	0.1	Not-Detect	Not-Detect	1	GTCCCGGTCTCCG		MIMAT0014708			
hsa-miR-4303	0.1	0.1	Not-Detect	Not-Detect	1	CTGTCCTCAGCTCAGA	chr12	MIMAT0016856	98389187	98389173	+
hsa-miR-1279	0.1	0.1	Not-Detect	Not-Detect	1	AGAAAGAAGCAATATGA	chr12	MIMAT0005937	69666967	69666952	+
hsa-miR-548c-5p	0.1	0.1	Not-Detect	Not-Detect	1	GGCAAAAACCGCAATTACTTTT	chr12	MIMAT0004806	65016314	65016334	−
hsa-miR-660	47.239033	47.23903333	Detected	Detected	1	CAACTCCGATATGCAATGG	chrX	MIMAT0003338	49777868	49777885	−
hsa-miR-373	0.1	0.1	Not-Detect	Not-Detect	1	ACACCCCAAAATCGAAGC	chr19	MIMAT0000726	54292008	54292024	−
hiv1-miR-H1	0.1	0.1	Not-Detect	Not-Detect	1	GCCCAGGCACG		MIMAT0004480			
hsa-miR-449c	0.1	0.1	Not-Detect	Not-Detect	1	ACAGCCGCTAGCAATA	chr5	MIMAT0010251	54468130	54468116	+
kshv-miR-K12-8	0.1	0.1	Not-Detect	Not-Detect	1	TGCTCTCTCAGTCGC		MIMAT0002186			
hsa-miR-302b*	0.1	0.1	Not-Detect	Not-Detect	1	GAAAGCACTTCCATGTTAAAG	chr4	MIMAT0000714	113569672	113569653	+
hsa-miR-553	0.1	0.1	Not-Detect	Not-Detect	1	AAAACAAAATCTCACCGTT	chr1	MIMAT0003216	100746815	100746832	−
hsa-miR-330-5p	0.1	0.1	Not-Detect	Not-Detect	1	GCCTAAGACACAGGCCC	chr19	MIMAT0004693	46142290	46142275	+
hsa-miR-1322	0.1	0.1	Not-Detect	Not-Detect	1	CAGCATCAGCAGCATC	chr8	MIMAT0005953	10682951	10682937	+
hsa-miR-559	0.1	0.1	Not-Detect	Not-Detect	1	TTTTGGTGCATATTTACTTTA	chr2	MIMAT0003223	47604830	47604849	−
hsa-miR-802	0.1	0.1	Not-Detect	Not-Detect	1	ACAAGGATGAATCTTTGTTA	chr21	MIMAT0004185	37093034	37093052	−
hsa-miR-196a*	0.1	0.1	Not-Detect	Not-Detect	1	CTCAGGCAGTTTCTT	chr12	MIMAT0004562	54385591	54385604	−
hsa-miR-3920	0.1	0.1	Not-Detect	Not-Detect	1	TCAGAGAGTTAAGATAATCAGT	chr11	MIMAT0018195	101390622	101390602	+
hsa-miR-1178	0.1	0.1	Not-Detect	Not-Detect	1	CTAGGGAAGAACAGTGA	chr12	MIMAT0005823	120151514	120151499	+
hsa-miR-499-5p	0.1	0.1	Not-Detect	Not-Detect	1	AAACATCACTGCAAGTCTTAA	chr20	MIMAT0002870	33578212	33578231	−
hsa-miR-3130-3p	0.1	0.1	Not-Detect	Not-Detect	1	TTACCCAGTCTCCGG	chr2	MIMAT0014994	207648007	207648021	−
hsa-miR-589*	0.1	0.1	Not-Detect	Not-Detect	1	TCTGGGAACCGGCAT	chr7	MIMAT0003256	5502059	5502045	+
hsa-miR-3613-5p	0.1	0.1	Not-Detect	Not-Detect	1	GAACAAAAAAAAAAGTACAAC	chr13	MIMAT0017990	50570587	50570568	+
hsa-miR-208b	0.1	0.1	Not-Detect	Not-Detect	1	ACAAACCTTTTGTTCGTCTTA	chr14	MIMAT0004960	23887262	23887243	+
hsa-miR-1233	0.1	0.1	Not-Detect	Not-Detect	1	CTGCGGGAGGAC	chr15	MIMAT0005588	34674351	34674340	+
hsa-miR-516a-3p	0.1	0.1	Not-Detect	Not-Detect	1	ACCCTCTGAAAGGAAGCA	chr19	MIMAT0006778	54228751	54228768	−
hsa-miR-3159	0.1	0.1	Not-Detect	Not-Detect	1	GTGGCCGACACTTG	chr11	MIMAT0015033	18409352	18409364	−
hsa-miR-618	0.1	0.1	Not-Detect	Not-Detect	1	ACTCAGAAGGACAAGTAG	chr12	MIMAT0003287	81329552	81329536	+
hsv1-miR-H2	0.1	0.1	Not-Detect	Not-Detect	1	AGTCGCACTCGTCC		MIMAT0008399			
hsa-miR-4279	0.1	0.1	Not-Detect	Not-Detect	1	GAAGCCGGGAGGA	chr5	MIMAT0016909	31936255	31936244	+
hsa-miR-522	0.1	0.1	Not-Detect	Not-Detect	1	ACACTCTAAAGGGAACCATTT	chr19	MIMAT0002868	54254520	54254539	−
hsa-miR-3189	0.1	0.1	Not-Detect	Not-Detect	1	CTACCCCATCAGACCC	chr19	MIMAT0015071	18497423	18497437	−
hsa-miR-645	0.1	0.1	Not-Detect	Not-Detect	1	TCAGCAGTACCAGCCTAG	chr20	MIMAT0003315	49202385	49202401	−
hsa-miR-3680*	0.1	0.1	Not-Detect	Not-Detect	1	CCTACTCCCAGGGTC	chr16	MIMAT0018107	21517441	21517427	+
ebv-miR-BART9*	0.1	0.1	Not-Detect	Not-Detect	1	GTTTCCAATTCAGGGTC		MIMAT0004816			
hsv2-miR-H7-3p	0.1	0.1	Not-Detect	Not-Detect	1	GACGAGGGGTCGG		MIMAT0015375			
hsa-miR-431	0.1	0.1	Not-Detect	Not-Detect	1	TGCATGACGGCCTGC	chr14	MIMAT0001625	101347370	101347383	−
hsa-miR-548i	0.1	0.1	Not-Detect	Not-Detect	1	GGCAAAATCCGCAATT	chr3	MIMAT0005935	125509306	125509291	+
hsa-miR-665	0.1	0.1	Not-Detect	Not-Detect	1	AGGGGCCTCAGCCT	chr14	MIMAT0004952	101341419	101341431	−
hsa-miR-375	0.1	0.1	Not-Detect	Not-Detect	1	TCACGCGAGCCGAAC	chr2	MIMAT0000728	219866427	219866414	+
hsa-let-7b*	0.1	0.1	Not-Detect	Not-Detect	1	GGGAAGGCAGTAGGTT	chr22	MIMAT0004482	46509632	46509646	−
hsa-miR-452*	0.1	0.1	Not-Detect	Not-Detect	1	CACTTACTTCTTTGCAGA	chrX	MIMAT0001636	151128178	151128162	+
hsa-miR-3065-3p	0.1	0.1	Not-Detect	Not-Detect	1	CTCCAACAATATCCTGGT	chr17	MIMAT0015378	79099732	79099748	−
hsa-miR-483-5p	0.1	0.1	Not-Detect	Not-Detect	1	CTCCCTTCTTTCCTC	chr11	MIMAT0004761	2155392	2155379	+
hsa-miR-135a*	0.1	0.1	Not-Detect	Not-Detect	1	CGCCACGGCTCCA	chr3	MIMAT0004595	52328311	52328300	+
hsa-miR-568	0.1	0.1	Not-Detect	Not-Detect	1	GTGTGTATACATTTATACAT	chr3	MIMAT0003232	114035356	114035338	+
hsa-miR-33b*	0.1	0.1	Not-Detect	Not-Detect	1	GGGCTGCACTGCCG	chr17	MIMAT0004811	17717224	17717212	+
hsa-miR-877	0.1	0.1	Not-Detect	Not-Detect	1	CCCTGCGCCATCT	chr6	MIMAT0004949	30552117	30552128	−
hsa-miR-198	0.1	0.1	Not-Detect	Not-Detect	1	GAACCTATCTCCCCTC	chr3	MIMAT0000228	120114541	120114527	+
hsa-miR-3927	0.1	0.1	Not-Detect	Not-Detect	1	ATGCCTATCAAATATCTACCT	chr9	MIMAT0018202	112273820	112273801	+
hsa-miR-1185	0.1	0.1	Not-Detect	Not-Detect	1	AACATACAAAGGGTATCCT	chr14	MIMAT0005798	101509330	101509348	−
hsa-miR-3136	0.1	0.1	Not-Detect	Not-Detect	1	AATGACCCTACCTATTCAG	chr3	MIMAT0015003	69098140	69098123	+
hsa-miR-147b	0.1	0.1	Not-Detect	Not-Detect	1	TAGCAGAAGCATTTCCGC	chr15	MIMAT0004928	45725301	45725317	−
hsa-miR-3618	0.1	0.1	Not-Detect	Not-Detect	1	GCTCTTTTCATTAATGTAGAC	chr22	MIMAT0017998	20073322	20073341	−
hsa-miR-4254	0.1	0.1	Not-Detect	Not-Detect	1	GAGATGGTGGAGTAGC	chr1	MIMAT0016884	32224326	32224312	+
hsa-miR-1243	0.1	0.1	Not-Detect	Not-Detect	1	CACTCCTATAATTGATCCAG	chr4	MIMAT0005894	114028026	114028044	−
hsa-miR-518a-3p	0.1	0.1	Not-Detect	Not-Detect	1	TCCAGCAAAGGGAAGCGC	chr19	MIMAT0002863	54234314	54234331	−
hsa-miR-3166	0.1	0.1	Not-Detect	Not-Detect	1	TAGGCCAGTAGGCATTG	chr11	MIMAT0015040	87909736	87909751	−
hsa-miR-3663-3p	0.1	0.1	Not-Detect	Not-Detect	1	GCGCCCGGCCT	chr10	MIMAT0018085	118927268	118927259	+
ebv-miR-BART17-5p	0.1	0.1	Not-Detect	Not-Detect	1	CTTGTATGCCTGCGT		MIMAT0003715			
hsv1-miR-H5-5p	0.1	0.1	Not-Detect	Not-Detect	1	GTAGAGATGCCCGAAC		MIMAT0015280			
hsa-miR-224	0.1	0.1	Not-Detect	Not-Detect	1	AACGGAACCACTAGTGACTT	chrX	MIMAT0000281	151127077	151127059	+
hsa-miR-1267	0.1	0.1	Not-Detect	Not-Detect	1	TGGGGATTACACTTCAAC	chr13	MIMAT0005921	108183550	108183534	+
hsa-miR-16-1*	0.1	0.1	Not-Detect	Not-Detect	1	TCAGCAGCACAGTTA	chr13	MIMAT0004489	50623185	50623172	+
hsa-miR-3686	0.1	0.1	Not-Detect	Not-Detect	1	TCATTTACTTTCTCTTACAGA	chr8	MIMAT0018114	130496378	130496359	+
hbv-miR-B4	0.1	0.1	Not-Detect	Not-Detect	1	CGCCCACCAACCA		MIMAT0012171			
kshv-miR-K12-10a*	0.1	0.1	Not-Detect	Not-Detect	1	AGTGGTGGTATCGCC		MIMAT0015212			
hsa-miR-4315	0.1	0.1	Not-Detect	Not-Detect	1	GTCCAGCTCAGAAAGC	chr17	MIMAT0016866	43552756	43552741	+
hsa-miR-129-5p	0.1	0.1	Not-Detect	Not-Detect	1	GCAAGCCCAGACCGC	chr7	MIMAT0000242	127635171	127635185	−
hsa-miR-548p	0.1	0.1	Not-Detect	Not-Detect	1	AAAGTAACTGCAGTTTTTGC	chr5	MIMAT0005934	100152253	100152235	+
hsa-miR-3201	0.1	0.1	Not-Detect	Not-Detect	1	ATTTTTCTTCATATCCC	chr22	MIMAT0015086	48670177	48670192	−
hsa-miR-676	0.1	0.1	Not-Detect	Not-Detect	1	AACTCAACAACCTTAGGA	chrX	MIMAT0018204	69242753	69242769	−
hsa-miR-1909	0.1	0.1	Not-Detect	Not-Detect	1	CGGTGAGCACCCG	chr19	MIMAT0007883	1816227	1816216	+
hsa-miR-1	0.1	0.1	Not-Detect	Not-Detect	1	ATACATACTTCTTTACATTCC	chr18	MIMAT0000416	19409038	19409018	+
hsa-miR-487b	0.1	0.1	Not-Detect	Not-Detect	1	AAGTGGATGACCCTGTAC	chr14	MIMAT0003180	101512847	101512863	−
hsa-miR-31	0.1	0.1	Not-Detect	Not-Detect	1	AGCTATGCCAGCATCTT	chr9	MIMAT0000089	21512141	21512126	+
hsa-miR-138-1*	0.1	0.1	Not-Detect	Not-Detect	1	GGCCCTGGTGTTG	chr3	MIMAT0004607	44155776	44155787	−
hsa-miR-34a	0.1	0.1	Not-Detect	Not-Detect	1	ACAACCAGCTAAGACACTGC	chr1	MIMAT0000255	9211769	9211751	+
hsa-miR-889	0.1	0.1	Not-Detect	Not-Detect	1	ACAATGGTTGTCCGATATTA	chr14	MIMAT0004921	101514288	101514306	−
hsa-miR-19b-1*	0.1	0.1	Not-Detect	Not-Detect	1	GCTGGATGCAAACCTGC	chr13	MIMAT0004491	92003468	92003483	−
hsa-miR-3938	0.1	0.1	Not-Detect	Not-Detect	1	CCGGGTTATCTACAAGG	chr3	MIMAT0018353	55886599	55886584	+
hsa-miR-1205	0.1	0.1	Not-Detect	Not-Detect	1	CTCAAAGCAAACCCTG	chr8	MIMAT0005869	128972891	128972905	−
hsa-miR-508-3p	0.1	0.1	Not-Detect	Not-Detect	1	TCTACTCCAAAAGGCTAC	chrX	MIMAT0002880	146126205	146126188	+
hsa-miR-3143	0.1	0.1	Not-Detect	Not-Detect	1	CGAAAGAAGCGCTTTA	chr6	MIMAT0015012	27115418	27115432	−
hsa-miR-602	0.1	0.1	Not-Detect	Not-Detect	1	GGGCCGCAGCTGTCG	chr9	MIMAT0003270	140732895	140732908	−
hsa-miR-3622a-5p	0.1	0.1	Not-Detect	Not-Detect	1	CTCACCTGAGCTCCC	chr8	MIMAT0018003	27559215	27559228	−
hsa-miR-2115*	0.1	0.1	Not-Detect	Not-Detect	1	CTAGCCTCCATGAATTCT	chr3	MIMAT0011159	48357928	48357912	+
hsa-miR-4261	0.1	0.1	Not-Detect	Not-Detect	1	TGGGTCCCTGTTTCC	chr2	MIMAT0016890	10332787	10332774	+
hsa-miR-1250	0.1	0.1	Not-Detect	Not-Detect	1	AAAGGCCACATCCAGC	chr17	MIMAT0005902	79107039	79107025	+
hsa-miR-518e*	0.1	0.1	Not-Detect	Not-Detect	1	CAGAAAGCGCTTCCCTCTA	chr19	MIMAT0005450	54189741	54189759	−
hsa-miR-3174	0.1	0.1	Not-Detect	Not-Detect	1	GGCTCTGCATCTCTAAC	chr15	MIMAT0015051	90550003	90550018	−
hsa-miR-3668	0.1	0.1	Not-Detect	Not-Detect	1	ATTTTGATCAATCTCTACATT	chr6	MIMAT0018091	140526398	140526417	−
ebv-miR-BART20-3p	0.1	0.1	Not-Detect	Not-Detect	1	GGTAACAGGCTGTGCC		MIMAT0003720			
hsa-miR-2355-5p	0.1	0.1	Not-Detect	Not-Detect	1	TTGTCCATTGTATCTGGG	chr2	MIMAT0016895	207974741	207974724	+
hsa-miR-4292	0.1	0.1	Not-Detect	Not-Detect	1	CCAAGGCCGGCC	chr9	MIMAT0016919	139725455	139725465	−
hsa-miR-1272	0.1	0.1	Not-Detect	Not-Detect	1	TTTCAGAATTTGCTGCCA	chr15	MIMAT0005925	65054630	65054614	+

hsa-miR-543	0.1	0.1	Not-Detect	Not-Detect	1	AAGAAGTGCACCGCGAATG	chr14	MIMAT0004954	101498374	101498391	-
hsa-miR-369-5p	0.1	0.1	Not-Detect	Not-Detect	1	GCGAATATAACACGGTCGAT	chr14	MIMAT0001621	101531946	101531964	-
hcmv-miR-UL70-3p	0.1	0.1	Not-Detect	Not-Detect	1	CCGCGCGCCAGC		MIMAT0003343			
hsa-miR-4325	0.1	0.1	Not-Detect	Not-Detect	1	TCACTGAGACAAGTGCA	chr20	MIMAT0016887	55896585	55896570	+
kshv-miR-K12-2*	0.1	0.1	Not-Detect	Not-Detect	1	CAGCTCTAGCCCTGG		MIMAT0015215			
hsa-miR-29b	25.118747	25.11874667	Detected	Detected	1	AACACTGATTTCAAATGGTGC	chr1	MIMAT0000100	207975861	207975841	+
hsa-miR-1296	0.1	0.1	Not-Detect	Not-Detect	1	GGAGATGGAGCCAGG	chr10	MIMAT0005794	65132753	65132740	+
hsa-miR-548x	0.1	0.1	Not-Detect	Not-Detect	1	TGAAAGTAATTGCAGTTTTTA	chr21	MIMAT0015081	20058473	20058454	+
hsa-miR-323-3p	0.1	0.1	Not-Detect	Not-Detect	1	AGAGGTCGACCGTGTAATGT	chr14	MIMAT0000755	100561873	100561892	-
hsa-miR-711	0.1	0.1	Not-Detect	Not-Detect	1	CTTACGTCTCTCCCTG	chr3	MIMAT0012734	48616398	48616384	+
hsa-miR-1911*	0.1	0.1	Not-Detect	Not-Detect	1	GGAGACCACAATGCC	chrX	MIMAT0007886	113997798	113997811	-
hsa-miR-380*	0.1	0.1	Not-Detect	Not-Detect	1	GCGCATGTTCTATGGTC	chr14	MIMAT0000734	100561116	100561132	-
hsa-miR-760	0.1	0.1	Not-Detect	Not-Detect	1	TCCCCACAGACCCA	chr1	MIMAT0004957	94312443	94312455	-
hsa-miR-3909	0.1	0.1	Not-Detect	Not-Detect	1	AGACTGCAGGCCCT	chr22	MIMAT0018183	35731712	35731724	-
hsa-miR-103-as	0.1	0.1	Not-Detect	Not-Detect	1	AGCAGCATTGTACAGGG	chr5	MIMAT0007402	167987915	167987931	-
hsa-miR-3120	0.1	0.1	Not-Detect	Not-Detect	1	TGCCTGTCTACACTTGC	chr1	MIMAT0014982	172108003	172108018	-
hsa-miR-141*	0.1	0.1	Not-Detect	Not-Detect	1	TCCAACACTGTACTGGAA	chr12	MIMAT0004598	7073281	7073297	-
hsa-miR-580	0.1	0.1	Not-Detect	Not-Detect	1	CCTAATGATTCATCATTCTC	chr5	MIMAT0003245	36148075	36148057	+
hsa-miR-3605-5p	0.1	0.1	Not-Detect	Not-Detect	1	GGCTTCCTTGCTATCC	chr1	MIMAT0017981	33798037	33798023	+
hsa-miR-9*	0.1	0.1	Not-Detect	Not-Detect	1	ACTTTCGGTTATCTAGCTT	chr1	MIMAT0000442	154656832	154656814	+
hsa-miR-200c*	0.1	0.1	Not-Detect	Not-Detect	1	CCAAACACTGCTGGGTA	chr12	MIMAT0004657	7072872	7072887	-
hsa-miR-3945	0.1	0.1	Not-Detect	Not-Detect	1	ATATCAACCCTCTCCTATGC	chr4	MIMAT0018361	185772211	185772193	+
hsa-miR-1224-3p	0.1	0.1	Not-Detect	Not-Detect	1	CTGAGGAGAGAGGAGG	chr3	MIMAT0005459	183959263	183959277	-
hsa-miR-512-3p	0.1	0.1	Not-Detect	Not-Detect	1	GACCTCAGCTATGACAGCA	chr19	MIMAT0002823	54169986	54170004	-
hsa-miR-3149	0.1	0.1	Not-Detect	Not-Detect	1	ATACACACACATATCCATACA	chr8	MIMAT0015022	77879076	77879057	+
hsa-miR-1538	0.1	0.1	Not-Detect	Not-Detect	1	AGGAACAGCAGCAGC	chr16	MIMAT0007400	69599771	69599758	+
hsa-miR-609	0.1	0.1	Not-Detect	Not-Detect	1	AGAGATGAGAGAAACACCC	chr10	MIMAT0003277	105978581	105978564	+
hsa-miR-3647-5p	0.1	0.1	Not-Detect	Not-Detect	1	ATGAATGTGAATCATCACTTC	chr16	MIMAT0018066	70563419	70563438	-
hsv1-miR-H1	0.1	0.1	Not-Detect	Not-Detect	1	TCCACTTCCCGTCC		MIMAT0003744			
hsa-miR-4268	0.1	0.1	Not-Detect	Not-Detect	1	CACATCCTGAGAGGAG	chr2	MIMAT0016896	220771276	220771262	+
hsa-miR-1256	0.1	0.1	Not-Detect	Not-Detect	1	AGCTAGTGAGAAGTCAATG	chr1	MIMAT0005907	21314862	21314845	+
hsa-miR-519e*	0.1	0.1	Not-Detect	Not-Detect	1	GAAAGTGCTCCCTTTTG	chr19	MIMAT0002828	54183213	54183228	-
hsa-miR-635	0.1	0.1	Not-Detect	Not-Detect	1	GGACATTGTTTCAGTGCCCA	chr17	MIMAT0003305	66420629	66420611	+
hsa-miR-3673	0.1	0.1	Not-Detect	Not-Detect	1	TATTCCGTATATACATTCCAT	chr8	MIMAT0018096	130508118	130508098	+
ebv-miR-BART4	0.1	0.1	Not-Detect	Not-Detect	1	AGCACACCAGCAGCA		MIMAT0003412			
hsv2-miR-H22	0.1	0.1	Not-Detect	Not-Detect	1	GCCCACCCACGTC		MIMAT0014704			
hsa-miR-24-1*	0.1	0.1	Not-Detect	Not-Detect	1	ACTGATATCAGCTCAGTAGGC	chr9	MIMAT0000079	96888131	96888151	-
hsa-miR-548aa	0.1	0.1	Not-Detect	Not-Detect	1	TGGTGCAAAAGTAATTGTG	chr8	MIMAT0018447	124360334	124360352	-
hsa-miR-656	0.1	0.1	Not-Detect	Not-Detect	1	AGAGGTTGACTGTATAATATT	chr14	MIMAT0003332	101533104	101533123	-
hsa-miR-1827	0.1	0.1	Not-Detect	Not-Detect	1	ATTCAATCTACTGCCTCA	chr12	MIMAT0006767	100583711	100583727	-
hsa-miR-371-5p	0.1	0.1	Not-Detect	Not-Detect	1	AGTGCCCCCACAG	chr19	MIMAT0004687	54290942	54290953	-
hcmv-miR-US33-5p	0.1	0.1	Not-Detect	Not-Detect	1	CGCCCACGGTCC		MIMAT0001584			
hsa-miR-448	0.1	0.1	Not-Detect	Not-Detect	1	ATGGGACATCCTACATATGCA	chrX	MIMAT0001532	114058089	114058108	-
kshv-miR-K12-6-3p	0.1	0.1	Not-Detect	Not-Detect	1	CTCAACAGCCCGAA		MIMAT0002189			
hsa-miR-301b	0.1	0.1	Not-Detect	Not-Detect	1	GCTTTGACAATATCATTGCAC	chr22	MIMAT0004958	22007317	22007336	-
hsa-miR-1304	0.1	0.1	Not-Detect	Not-Detect	1	CACATCTCACTGTAGCC	chr11	MIMAT0005892	93466880	93466865	+
hsa-miR-551a	0.1	0.1	Not-Detect	Not-Detect	1	TGGAAACCAAGAGTGGG	chr1	MIMAT0003214	3477339	3477324	+
hsa-miR-556-3p	0.1	0.1	Not-Detect	Not-Detect	1	AAAGATGAGCTAATGGTAATAT	chr1	MIMAT0004793	162312391	162312411	-
hsa-miR-335	0.1	0.1	Not-Detect	Not-Detect	1	ACATTTTTCGTTATTGCTC	chr7	MIMAT0000765	130135972	130135989	-
hsa-miR-767-5p	0.1	0.1	Not-Detect	Not-Detect	1	CATGCTCAGACAACCAT	chrX	MIMAT0003882	151561941	151561926	+
hsa-miR-194*	0.1	0.1	Not-Detect	Not-Detect	1	CAGATAACAGCAGCCCC	chr11	MIMAT0004671	64658898	64658883	+
hsa-miR-3916	0.1	0.1	Not-Detect	Not-Detect	1	CTGAGAACCAGCCATT	chr1	MIMAT0018190	247365313	247365299	+
hsa-miR-497	0.1	0.1	Not-Detect	Not-Detect	1	ACAAACCACAGTGTGCTG	chr17	MIMAT0002820	6921273	6921257	+
hsa-miR-3126-5p	0.1	0.1	Not-Detect	Not-Detect	1	TGCTTCTGGCATCTGT	chr2	MIMAT0014989	69330830	69330844	-
hsa-miR-586	0.1	0.1	Not-Detect	Not-Detect	1	GGACCTAAAAATACAATGCA	chr6	MIMAT0003252	45165447	45165429	+
hsa-miR-3610	0.1	0.1	Not-Detect	Not-Detect	1	CGGCGCCTCCTT	chr8	MIMAT0017987	117887027	117887017	+
hsa-miR-92a-2*	0.1	0.1	Not-Detect	Not-Detect	1	GTAATGCAACAAATCCCCAC	chrX	MIMAT0004508	133303597	133303579	+
hsa-miR-2052	0.1	0.1	Not-Detect	Not-Detect	1	ACATTACTGTTATCAAAACA	chr8	MIMAT0009977	75617930	75617948	-
hsa-miR-421	0.1	0.1	Not-Detect	Not-Detect	1	GCGCCCAATTAATGTCTG	chrX	MIMAT0003339	73438281	73438265	+
hsa-miR-514b-3p	0.1	0.1	Not-Detect	Not-Detect	1	TCCACTCACAGAGGTG	chrX	MIMAT0015088	146331738	146331724	+
hsa-miR-3155	0.1	0.1	Not-Detect	Not-Detect	1	AGTTCCCACTGCAGAG	chr10	MIMAT0015029	6194216	6194230	-
hsa-miR-15a*	0.1	0.1	Not-Detect	Not-Detect	1	TGAGGCAGCACAATATGGC	chr13	MIMAT0004488	50623326	50623309	+
hsa-miR-615-5p	0.1	0.1	Not-Detect	Not-Detect	1	GATCCGAGCACCGGG	chr12	MIMAT0004804	54427759	54427772	-
ebv-miR-BART10*	0.1	0.1	Not-Detect	Not-Detect	1	TGTACAGAACCAAAGAGGTGGC		MIMAT0004817			
hsv1-miR-H15	0.1	0.1	Not-Detect	Not-Detect	1	CGTGGCGGCCC		MIMAT0014693			
hsa-miR-219-1-3p	0.1	0.1	Not-Detect	Not-Detect	1	CGGGACGTCCAGAC	chr6	MIMAT0004567	33175682	33175694	-
hsa-miR-4275	0.1	0.1	Not-Detect	Not-Detect	1	AAAGAAGTGGTAATTGG	chr4	MIMAT0016905	28821215	28821230	-
hsa-miR-125b-2*	0.1	0.1	Not-Detect	Not-Detect	1	GTCCCAAGAGCCTGA	chr21	MIMAT0004603	17962618	17962631	-
hsa-miR-520f	0.1	0.1	Not-Detect	Not-Detect	1	AACCCTCTAAAAGGAAGCACT	chr19	MIMAT0002830	54185468	54185488	-
hsa-miR-3186-3p	0.1	0.1	Not-Detect	Not-Detect	1	CAAAGCCATCTCTCCG	chr17	MIMAT0015068	79418202	79418188	+
hsa-miR-642a	0.1	0.1	Not-Detect	Not-Detect	1	CAAGACACATTTGGAGAG	chr19	MIMAT0003312	46178205	46178222	-
hsa-miR-3678-5p	0.1	0.1	Not-Detect	Not-Detect	1	CACAGCAGAGTTTGTAC	chr17	MIMAT0018102	73402162	73402177	-
ebv-miR-BART7*	0.1	0.1	Not-Detect	Not-Detect	1	TGTTTCATAGTCAAGGTCC		MIMAT0004815			
hsv2-miR-H4-5p	0.1	0.1	Not-Detect	Not-Detect	1	GCATGCGTGCCGA		MIMAT0010203			
hsa-miR-548e	0.1	0.1	Not-Detect	Not-Detect	1	TGCAAAAGTAGTCTCAGTT	chr10	MIMAT0005874	112748740	112748757	-
hsa-miR-663	0.1	0.1	Not-Detect	Not-Detect	1	GCGGTCCCGCGGC	chr20	MIMAT0003326	26188857	26188846	+
hsa-miR-186*	0.1	0.1	Not-Detect	Not-Detect	1	CCCAAAAAATTCACCTTTGGGC	chr1	MIMAT0004612	71533388	71533368	+
hsa-miR-374a*	0.1	0.1	Not-Detect	Not-Detect	1	AATTACAATACAATCTGATAAG	chrX	MIMAT0004688	73507183	73507163	+
hiv1-miR-TAR-5p	0.1	0.1	Not-Detect	Not-Detect	1	TCAGATCTGGTCTAACCA		MIMAT0006016			
hsa-miR-450b-3p	0.1	0.1	Not-Detect	Not-Detect	1	TATGGATGCAAAATGATCCCAA	chrX	MIMAT0004910	133674283	133674263	+
kshv-miR-K12-9*	0.1	0.1	Not-Detect	Not-Detect	1	AGCGGGGTTTACGCA		MIMAT0002184			
hsa-miR-302d	0.1	0.1	Not-Detect	Not-Detect	1	ACACTCAAACATGGAAGCA	chr4	MIMAT0000718	113569225	113569208	+
hsa-miR-133a	0.1	0.1	Not-Detect	Not-Detect	1	CAGCTGGTTGAAGGGGA	chr18	MIMAT0000427	19405732	19405716	+
hsa-miR-563	0.1	0.1	Not-Detect	Not-Detect	1	GGGAAACGTATGTCAAC	chr3	MIMAT0003227	15915332	15915347	-
hsa-miR-339-5p	0.1	0.1	Not-Detect	Not-Detect	1	CGTGAGCTCCTGGA	chr7	MIMAT0000764	1062605	1062593	+
hsa-miR-875-3p	0.1	0.1	Not-Detect	Not-Detect	1	CACAACCTCAGTGTTTC	chr8	MIMAT0004923	100549078	100549063	+
hsa-miR-3923	0.1	0.1	Not-Detect	Not-Detect	1	CCCTAATCCAACATTACTAG	chr3	MIMAT0018198	79557090	79557108	-
hsa-miR-1181	0.1	0.1	Not-Detect	Not-Detect	1	CGGCTCGGGTGG	chr19	MIMAT0005826	10514167	10514157	+
hsa-miR-3132	0.1	0.1	Not-Detect	Not-Detect	1	TCCTCTGAGCTCCTTC	chr2	MIMAT0014997	220413825	220413811	+
hsa-miR-591	0.1	0.1	Not-Detect	Not-Detect	1	ACAATGAGAACCCATGGT	chr7	MIMAT0003259	95849008	95848992	+
hsa-miR-3615	0.1	0.1	Not-Detect	Not-Detect	1	GAGCCGCGAGGAG	chr17	MIMAT0017994	72744811	72744822	-
hsa-miR-935	0.1	0.1	Not-Detect	Not-Detect	1	GCGGTAGCGGAAGCGG	chr19	MIMAT0004978	54485624	54485638	-
hsa-miR-425*	0.1	0.1	Not-Detect	Not-Detect	1	GGGCGGACACGAC	chr3	MIMAT0001343	49057656	49057645	+
hsa-miR-1237	0.1	0.1	Not-Detect	Not-Detect	1	CTGGGGGACGG	chr11	MIMAT0005592	64136166	64136175	-
hsa-miR-517*	0.1	0.1	Not-Detect	Not-Detect	1	AGACAGTGCTTCCATCT	chr19	MIMAT0002851	54215541	54215557	-
hsa-miR-621	0.1	0.1	Not-Detect	Not-Detect	1	AGGTAAGCGCTGTTGC	chr13	MIMAT0003290	41384968	41384982	-
hsa-miR-3659	0.1	0.1	Not-Detect	Not-Detect	1	TGCCCTCGTAGACAAC	chr1	MIMAT0018080	38554967	38554981	-
ebv-miR-BART14*	0.1	0.1	Not-Detect	Not-Detect	1	TGTAAATCGGCAGCGTA		MIMAT0003425			
hsv1-miR-H3*	0.1	0.1	Not-Detect	Not-Detect	1	ACTCGGAACCCGCG		MIMAT0015279			
hsa-miR-4282	0.1	0.1	Not-Detect	Not-Detect	1	TCCTGGATGCAAATTTTA	chr6	MIMAT0016912	73677466	73677450	+
hsa-miR-1263	0.1	0.1	Not-Detect	Not-Detect	1	ACTCAGTATGCCAGGG	chr3	MIMAT0005915	163889292	163889278	+
hsa-miR-525-3p	0.1	0.1	Not-Detect	Not-Detect	1	CGCTCTAAAGGGAAGCGC	chr19	MIMAT0002839	54200843	54200859	-
hsa-miR-3192	0.1	0.1	Not-Detect	Not-Detect	1	TTCCACTGCTACAACCT	chr20	MIMAT0015076	18451275	18451290	-
hsa-miR-648	0.1	0.1	Not-Detect	Not-Detect	1	ACCAGTGCCCTGCACA	chr22	MIMAT0003318	18463667	18463653	+
hsa-miR-3682	0.1	0.1	Not-Detect	Not-Detect	1	CTACCTCCACCTGTATC	chr2	MIMAT0018110	54076328	54076313	+
ebv-miR-BHRF1-2*	0.1	0.1	Not-Detect	Not-Detect	1	GCTATCTGCTGCAACAG		MIMAT0000996			
hsa-miR-4316	0.1	0.1	Not-Detect	Not-Detect	1	CACCAGCTAGCCTCA	chr17	MIMAT0016867	75393092	75393079	+
hsv2-miR-H9-5p	0.1	0.1	Not-Detect	Not-Detect	1	ACCGCGACTCCACC		MIMAT0015376			
hsa-miR-4311	0.1	0.1	Not-Detect	Not-Detect	1	CACACTCAGCTCTCTTT	chr15	MIMAT0016863	66332583	66332598	-
hsa-miR-1288	0.1	0.1	Not-Detect	Not-Detect	1	TCTCCAGATCAGGGCA	chr17	MIMAT0005942	16185378	16185392	-

hsa-miR-548l	0.1	0.1	Not-Detect	Not-Detect	1	GACAAAACCCGCAAA	chr11	MIMAT0005889	94199696	94199683	+
hsa-miR-32	0.1	0.1	Not-Detect	Not-Detect	1	TGCAACTTAGTAATGTGCAA	chr9	MIMAT0000090	111808535	111808517	+
hsa-miR-671-3p	0.1	0.1	Not-Detect	Not-Detect	1	GGTGGAGCCCTGA	chr7	MIMAT0004819	150935583	150935594	-
hsa-miR-376b	0.1	0.1	Not-Detect	Not-Detect	1	AACATGGATTTTCCTCTATG	chr14	MIMAT0002172	101506837	101506855	-
hsa-miR-455-3p	0.1	0.1	Not-Detect	Not-Detect	1	GTGTATATGCCCATGGA	chr9	MIMAT0004784	116971772	116971787	-
hsa-miR-485-5p	0.1	0.1	Not-Detect	Not-Detect	1	GAATTCATCACGGCCAGC	chr14	MIMAT0002175	101521769	101521785	-
hsa-miR-136	0.1	0.1	Not-Detect	Not-Detect	1	TCCATCATCAAAACAAATGGAGT	chr14	MIMAT0000448	101351054	101351075	-
hsa-miR-571	0.1	0.1	Not-Detect	Not-Detect	1	CTCACTCAGATGGCCAAC	chr4	MIMAT0003236	344010	344026	-
hsa-miR-885-5p	0.1	0.1	Not-Detect	Not-Detect	1	AGAGGCAGGGTAGTGTA	chr3	MIMAT0004947	10436204	10436189	+
hsa-miR-3934	0.1	0.1	Not-Detect	Not-Detect	1	CTGCCTCAGTTTCCA	chr6	MIMAT0018349	33665937	33665950	-
hsa-miR-1200	0.1	0.1	Not-Detect	Not-Detect	1	GAGGCTCAGAATGGC	chr7	MIMAT0005863	36958991	36958978	+
hsa-miR-3139	0.1	0.1	Not-Detect	Not-Detect	1	AACAGGCATCTGTTGAG	chr4	MIMAT0015007	144264629	144264644	-
hsa-miR-598	0.1	0.1	Not-Detect	Not-Detect	1	TGACGATGACAACGATGAC	chr8	MIMAT0003266	10892797	10892780	+
hsa-miR-2113	0.1	0.1	Not-Detect	Not-Detect	1	GTGACAGAGCCAAGC	chr6	MIMAT0009206	98472469	98472482	-
hsa-miR-4257	0.1	0.1	Not-Detect	Not-Detect	1	CTCAGTCCCCACCT	chr1	MIMAT0016878	150524468	150524480	-
hsa-miR-518c	0.1	0.1	Not-Detect	Not-Detect	1	ACACTCTAAAGAGAAGCGCTT	chr19	MIMAT0002848	54212053	54212072	-
hsa-miR-3169	0.1	0.1	Not-Detect	Not-Detect	1	CTATGTGCCAAGCACA	chr13	MIMAT0015044	61773964	61773950	+
hsa-miR-626	0.1	0.1	Not-Detect	Not-Detect	1	AAGACATTTTCAGACAGCT	chr15	MIMAT0003295	41983844	41983861	-
ebv-miR-BART19-3p	0.1	0.1	Not-Detect	Not-Detect	1	AGCATTCCCAAGCAAACA		MIMAT0003718			
hsv1-miR-H7	0.1	0.1	Not-Detect	Not-Detect	1	CCTTTGGTTGCAGACC		MIMAT0012595			
hsa-miR-2277-3p	0.1	0.1	Not-Detect	Not-Detect	1	GAGCCAGGCAGGG	chr5	MIMAT0011777	92956480	92956468	+
hsa-miR-4289	0.1	0.1	Not-Detect	Not-Detect	1	TGATAGCCCTGCACAA	chr9	MIMAT0016920	91360779	91360765	+
hsa-miR-127-3p	0.1	0.1	Not-Detect	Not-Detect	1	AGCCAAGCTCAGACGGAT	chr14	MIMAT0000446	100419129	100419146	-
hsa-miR-541	0.1	0.1	Not-Detect	Not-Detect	1	AGTCCAGATTCTGTGCCC	chr14	MIMAT0004920	101530890	101530906	-
hsa-miR-3689a-3p	0.1	0.1	Not-Detect	Not-Detect	1	ACCACGATATCACACCT	chr9	MIMAT0018118	137741403	137741388	+
hcmv-miR-UL22A	0.1	0.1	Not-Detect	Not-Detect	1	TCTCACGGGAAGGCT		MIMAT0001574			
hsa-miR-4321	0.1	0.1	Not-Detect	Not-Detect	1	CGCAGGGCGGTC	chr19	MIMAT0016874	2250697	2250707	-
kshv-miR-K12-11*	0.1	0.1	Not-Detect	Not-Detect	1	CCTAGAAATGTTTAAGCTGT		MIMAT0015213			
hsa-miR-299-3p	0.1	0.1	Not-Detect	Not-Detect	1	AAGCGGTTTACCATCCCA	chr14	MIMAT0000687	101490174	101490190	-
hsa-miR-1292	0.1	0.1	Not-Detect	Not-Detect	1	CAGCGTCTGCCGG	chr20	MIMAT0005943	2633438	2633449	-
hsa-miR-548t	0.1	0.1	Not-Detect	Not-Detect	1	CAAAAACCACGATCACT	chr4	MIMAT0015009	174189325	174189340	-
hsa-miR-378c	0.1	0.1	Not-Detect	Not-Detect	1	CCACTCTTCTGACTCC	chr10	MIMAT0016847	132760885	132760871	+
hsa-miR-1914	0.1	0.1	Not-Detect	Not-Detect	1	CAGAAGTGGGCCGG	chr20	MIMAT0007889	62572851	62572839	+
hsa-miR-383	0.1	0.1	Not-Detect	Not-Detect	1	AGCCACAATCACCTTCT	chr8	MIMAT0000738	14710974	14710959	+
hsa-miR-489	0.1	0.1	Not-Detect	Not-Detect	1	GCTGCCGTATATGTGATG	chr7	MIMAT0002805	92951256	92951239	+
hsa-miR-3116	0.1	0.1	Not-Detect	Not-Detect	1	AGTCCCTACTATGTTCCA	chr1	MIMAT0014978	62544506	62544523	-
hsa-miR-576-5p	0.1	0.1	Not-Detect	Not-Detect	1	AAAGACGTGGAGAAATTAGAAT	chr4	MIMAT0003241	110409870	110409890	-
hsa-miR-34b*	0.1	0.1	Not-Detect	Not-Detect	1	CAATCAGCTAATGACACTGCCT	chr11	MIMAT0000685	110888886	110888907	-
hsa-miR-891b	0.1	0.1	Not-Detect	Not-Detect	1	TCAATGACTCAGGTAAGTTGC	chrX	MIMAT0004913	145082602	145082583	+
hsa-miR-200a*	0.1	0.1	Not-Detect	Not-Detect	1	TCCAGCACTGTCCGGT	chr1	MIMAT0001620	1103265	1103279	-
hsa-miR-3941	0.1	0.1	Not-Detect	Not-Detect	1	TATGATCCTCAGTTGTGTG	chr10	MIMAT0018357	124176544	124176561	-
hsa-miR-509-3p	0.1	0.1	Not-Detect	Not-Detect	1	CTACCCACAGACGTAC	chrX	MIMAT0002881	146340353	146340338	+
hsa-miR-3145	0.1	0.1	Not-Detect	Not-Detect	1	CAATTCCAAACACTCAAA	chr6	MIMAT0015016	138756421	138756405	+
hsa-miR-605	0.1	0.1	Not-Detect	Not-Detect	1	AGGAGAAGGCACCATGG	chr10	MIMAT0003273	53059355	53059370	-
hsa-miR-2117	0.1	0.1	Not-Detect	Not-Detect	1	CTGTCCTTGGCAAAGA	chr17	MIMAT0011162	41522230	41522244	-
hsa-miR-4264	0.1	0.1	Not-Detect	Not-Detect	1	AATGACCATGACTGAGT	chr2	MIMAT0016899	79876446	79876431	+
hsa-miR-1253	0.1	0.1	Not-Detect	Not-Detect	1	TGCAGGCTGATCTTCTT	chr17	MIMAT0005904	2651415	2651400	+
hsa-miR-519b-3p	0.1	0.1	Not-Detect	Not-Detect	1	AACCTCTAAAAGGATGCACTT	chr19	MIMAT0002837	54198519	54198538	-
hsa-miR-3177	0.1	0.1	Not-Detect	Not-Detect	1	ACGTGTCCCCAGTGC	chr16	MIMAT0015054	1785046	1785059	-
hsa-miR-631	0.1	0.1	Not-Detect	Not-Detect	1	GCTGAGGTCTGGGCC	chr15	MIMAT0003300	75645987	75645974	+
hsa-miR-367*	0.1	0.1	Not-Detect	Not-Detect	1	AGAGTTGCATATTAGCAACAGT	chr4	MIMAT0004686	113569056	113569036	+
ebv-miR-BART21-5p	0.1	0.1	Not-Detect	Not-Detect	1	GTTAGTTGCCTTCACTAG		MIMAT0010130			
hsv2-miR-H19	0.1	0.1	Not-Detect	Not-Detect	1	CAGTCCGTGCTTGC		MIMAT0014701			
hsa-miR-4295	0.1	0.1	Not-Detect	Not-Detect	1	AAGGAAAACATTGCACTG	chr10	MIMAT0016844	114393940	114393956	-
hsa-miR-1273d	0.1	0.1	Not-Detect	Not-Detect	1	ACTGCAGCCTCAACC	chr1	MIMAT0015090	10287796	10287809	-
hsa-miR-545	0.1	0.1	Not-Detect	Not-Detect	1	GCACACAATAAATGTTTGCTGA	chrX	MIMAT0003165	73507022	73507002	+
hsa-miR-3692	0.1	0.1	Not-Detect	Not-Detect	1	ACTTCTGCAGTGTCAGT	chr6	MIMAT0018122	157950215	157950230	-
hcmv-miR-US25-1*	0.1	0.1	Not-Detect	Not-Detect	1	GAGAACCGACCTAGCGT		MIMAT0004755			
hsa-miR-4328	0.1	0.1	Not-Detect	Not-Detect	1	AATCCTGGGAAAACTGG	chrX	MIMAT0016926	78156736	78156721	+
kshv-miR-K12-4-3p	0.1	0.1	Not-Detect	Not-Detect	1	TCAGCTAGGCCTCAGT		MIMAT0002192			
hsa-miR-1299	0.1	0.1	Not-Detect	Not-Detect	1	TCCCTCACACAGAATTC	chr9	MIMAT0005887	69002321	69002306	+
hsa-miR-549	0.1	0.1	Not-Detect	Not-Detect	1	AGAGCTCATCCATAGTTGTC	chr15	MIMAT0003333	81134399	81134381	+
hsa-miR-323b-5p	0.1	0.1	Not-Detect	Not-Detect	1	TGCGAACTCACCACGGA	chr14	MIMAT0001630	101522576	101522592	-
hsa-miR-764	0.1	0.1	Not-Detect	Not-Detect	1	AGGAGGACAAGTGAGC	chrX	MIMAT0010367	113873935	113873949	-
hsa-miR-193a-5p	0.1	0.1	Not-Detect	Not-Detect	1	TCATCTCGCCCGC	chr17	MIMAT0004614	29887045	29887056	-
hsa-miR-3912	0.1	0.1	Not-Detect	Not-Detect	1	ACATGTCCATATTATGCGT	chr5	MIMAT0018186	170813742	170813725	+
hsa-miR-106a*	0.1	0.1	Not-Detect	Not-Detect	1	GTAAGAAGTGCTTACATTGCAG	chrX	MIMAT0004517	133304298	133304278	+
hsa-miR-493*	0.1	0.1	Not-Detect	Not-Detect	1	AATGAAAGCCTACCATGTAC	chr14	MIMAT0002813	100405167	100405186	-
hsa-miR-3123	0.1	0.1	Not-Detect	Not-Detect	1	GATTAAACAATTCTCTG	chr1	MIMAT0014985	241295621	241295636	-
hsa-miR-143	0.1	0.1	Not-Detect	Not-Detect	1	GAGCTACAGTGCTTC	chr5	MIMAT0000435	148808548	148808561	-
hsa-miR-582-5p	0.1	0.1	Not-Detect	Not-Detect	1	AGTAACTGGTTGAACAACTGTA	chr5	MIMAT0003247	58999469	58999448	+
hsa-miR-3607-5p	0.1	0.1	Not-Detect	Not-Detect	1	ACTGATTTGCTTCATCACA	chr5	MIMAT0017984	85916329	85916346	-
hsa-miR-922	0.1	0.1	Not-Detect	Not-Detect	1	GACGTAGTCCTATTCTC	chr3	MIMAT0004972	197401445	197401430	+
hsa-miR-203	0.1	0.1	Not-Detect	Not-Detect	1	CTAGTGGTCCTAAACATT	chr14	MIMAT0000264	104583811	104583827	-
hsa-miR-410	0.1	0.1	Not-Detect	Not-Detect	1	ACAGGCCATCTGTGTTATA	chr14	MIMAT0002171	101532301	101532318	-
hsa-miR-1225-5p	58.108268	58.10826767	Detected	Detected	1	CCCCCCACTGG	chr16	MIMAT0005572	2140217	2140208	+
hsa-miR-513a-5p	0.1	0.1	Not-Detect	Not-Detect	1	ATGACACCTCCCTGTG	chrX	MIMAT0002877	146295034	146295019	+
hsa-miR-3151	0.1	0.1	Not-Detect	Not-Detect	1	ACCTGATCCCATTGCC	chr8	MIMAT0015024	104166857	104166871	-
hsa-miR-154*	0.1	0.1	Not-Detect	Not-Detect	1	AATAGGTCAACCGTGTATGA	chr14	MIMAT0000453	101526145	101526163	-
hsa-miR-612	0.1	0.1	Not-Detect	Not-Detect	1	AAGGAGCTCAGAAGCCC	chr11	MIMAT0003280	65211953	65211968	-
bkv-miR-B1-5p	0.1	0.1	Not-Detect	Not-Detect	1	ATGCTCTTCCCAAGTCT		MIMAT0009149			
hsv1-miR-H12	0.1	0.1	Not-Detect	Not-Detect	1	AAGCGTTCGCACTTC		MIMAT0014689			
hsa-miR-217	0.1	0.1	Not-Detect	Not-Detect	1	TCCAATCAGTTCCTGAT	chr2	MIMAT0000274	56210158	56210143	+
hsa-miR-125a-3p	0.1	0.1	Not-Detect	Not-Detect	1	GGCTCCCAAGAACCTCA	chr19	MIMAT0004602	52196565	52196580	-
hsa-miR-520b	0.1	0.1	Not-Detect	Not-Detect	1	CCCTCTAAAAGGAAGCACT	chr19	MIMAT0002843	54204524	54204541	-
hsa-miR-3182	0.1	0.1	Not-Detect	Not-Detect	1	GACTACACTACAGAAGC	chr16	MIMAT0015062	83541955	83541970	-
hsa-miR-3675-5p	0.1	0.1	Not-Detect	Not-Detect	1	GAAATCTCTACAGAAGCC	chr1	MIMAT0018098	17185477	17185461	+
ebv-miR-BART5*	0.1	0.1	Not-Detect	Not-Detect	1	AGGTGAACAGCGGC		MIMAT0009205			
hsa-miR-25*	0.1	0.1	Not-Detect	Not-Detect	1	CAATTGCCCAAGTCTCC	chr7	MIMAT0004498	99691216	99691201	+
hsa-miR-4302	0.1	0.1	Not-Detect	Not-Detect	1	CTCGCTGAGCCACA	chr12	MIMAT0016855	26026980	26026968	+
hsa-miR-1278	0.1	0.1	Not-Detect	Not-Detect	1	ATAGATGATATGCACAGTACT	chr1	MIMAT0005936	193105684	193105703	-
hsa-miR-548c-3p	0.1	0.1	Not-Detect	Not-Detect	1	GCAAAAGTAATTGAGATTTTTG	chr12	MIMAT0003285	63302616	63302637	-
hsa-miR-659	0.1	0.1	Not-Detect	Not-Detect	1	TGGGGACCCTCCCT	chr22	MIMAT0003337	38243766	38243754	+
hsa-miR-184	0.1	0.1	Not-Detect	Not-Detect	1	ACCCTTATCAGTTCTCCGTCCA	chr15	MIMAT0000454	79502183	79502203	-
hsa-miR-372	0.1	0.1	Not-Detect	Not-Detect	1	ACGCTCAAATGTCGCAGC	chr19	MIMAT0000724	54291191	54291207	-
hcmv-miR-US5-2	0.1	0.1	Not-Detect	Not-Detect	1	GACATCGTCACACCTA		MIMAT0001580			
hsa-miR-449b*	0.1	0.1	Not-Detect	Not-Detect	1	AGTGGCAGGGTAGTTG	chr5	MIMAT0009203	54466549	54466535	+
kshv-miR-K12-7*	0.1	0.1	Not-Detect	Not-Detect	1	CATAAATCCCCGTCCG		MIMAT0015217			
hsa-miR-302b	0.1	0.1	Not-Detect	Not-Detect	1	CTACTAAAACATGGAAGCACT	chr4	MIMAT0000715	113569709	113569690	+
hsa-miR-552	0.1	0.1	Not-Detect	Not-Detect	1	TTGTCTAACCAGTCACCTGT	chr1	MIMAT0003215	35135280	35135262	+
hsa-miR-330-3p	0.1	0.1	Not-Detect	Not-Detect	1	TCTCTGCAGGCCGTG	chr19	MIMAT0000751	46142330	46142316	+
hsa-miR-1321	0.1	0.1	Not-Detect	Not-Detect	1	ATCACATTCACCTCCCT	chrX	MIMAT0005952	85090839	85090854	-
hsa-miR-558	0.1	0.1	Not-Detect	Not-Detect	1	ATTTTGGTACAGCAGCTC	chr2	MIMAT0003222	32757282	32757298	-
hsa-miR-337-5p	0.1	0.1	Not-Detect	Not-Detect	1	AACTCCTGTATGAAGCCG	chr14	MIMAT0004695	101340856	101340872	-
hsa-miR-770-5p	0.1	0.1	Not-Detect	Not-Detect	1	TGGCCCTGACACGTGGT	chr14	MIMAT0003948	101318753	101318768	-
hsa-miR-196a	0.1	0.1	Not-Detect	Not-Detect	1	CCCAACAACATGAAACTACC	chr12	MIMAT0000226	54385548	54385567	-
hsa-miR-3919	0.1	0.1	Not-Detect	Not-Detect	1	ACTGAGTCCTTTGTTCTC	chr3	MIMAT0018193	159000493	159000509	-
hsa-miR-10b*	0.1	0.1	Not-Detect	Not-Detect	1	ATTCCCCTAGAATCGAATC	chr2	MIMAT0004556	177015100	177015117	-
hsa-miR-499-3p	0.1	0.1	Not-Detect	Not-Detect	1	AGCACAGACTTGCTGTGA	chr20	MIMAT0004772	33578253	33578269	-
hsa-miR-3129	0.1	0.1	Not-Detect	Not-Detect	1	AAACCAATCTCTACACTACT	chr2	MIMAT0014992	189997791	189997773	+

hsa-miR-1469	0.1	0.1	Not-Detect	Not-Detect	1	GGAGCCCGCGC	chr15	MIMAT0007347	96876502	96876511	-
hsa-miR-589	0.1	0.1	Not-Detect	Not-Detect	1	CTCAGAGCAGACGTGG	chr7	MIMAT0004799	5535494	5535480	+
hsa-miR-3613-3p	0.1	0.1	Not-Detect	Not-Detect	1	GAAGGGTTGGGCTTT	chr13	MIMAT0017991	50570626	50570613	+
hsa-miR-208a	0.1	0.1	Not-Detect	Not-Detect	1	ACAAGCTTTTTGCTCGTCTT	chr14	MIMAT0000241	22927709	22927690	+
hsa-miR-1231	0.1	0.1	Not-Detect	Not-Detect	1	GCAGCTGTCCG	chr1	MIMAT0005586	201777753	201777762	-
hsa-miR-515-5p	0.1	0.1	Not-Detect	Not-Detect	1	CAGAAAGTGCTTTCTTTTGGAGA	chr19	MIMAT0002826	54182271	54182293	-
hsa-miR-3158	0.1	0.1	Not-Detect	Not-Detect	1	GTCCTGCAGAGAGGA	chr10	MIMAT0015032	103361230	103361244	-
hsa-miR-617	0.1	0.1	Not-Detect	Not-Detect	1	GCCACCTTCAAATGGGA	chr12	MIMAT0003286	81226348	81226333	+
hsa-miR-3655	0.1	0.1	Not-Detect	Not-Detect	1	AGCAACACCGCAGC	chr5	MIMAT0018075	140027437	140027449	-
hsa-miR-4278	0.1	0.1	Not-Detect	Not-Detect	1	CAAGGGCAAACCCC	chr5	MIMAT0016910	6828024	6828012	+
hsa-miR-521	0.1	0.1	Not-Detect	Not-Detect	1	ACACTCTAAAGGGAAGTGCG	chr19	MIMAT0002854	54219903	54219922	-
hsa-miR-644	0.1	0.1	Not-Detect	Not-Detect	1	GCTCTAAGAAAGCCACA	chr20	MIMAT0003314	33054193	33054208	-
hsa-miR-3680	0.1	0.1	Not-Detect	Not-Detect	1	TGCACAATCCTGTGAGT	chr16	MIMAT0018106	21517404	21517388	+
ebv-miR-BART9	0.1	0.1	Not-Detect	Not-Detect	1	ACTACGGGACCCATGAA		MIMAT0003419			
hsv2-miR-H6*	0.1	0.1	Not-Detect	Not-Detect	1	AGGATGGAAGGGCAG		MIMAT0015652			
hsa-miR-27a*	0.1	0.1	Not-Detect	Not-Detect	1	TGCTCACAAGCAGCTA	chr19	MIMAT0004501	13947284	13947270	+
hsa-miR-4309	0.1	0.1	Not-Detect	Not-Detect	1	TGGAATCCTAGACTCCA	chr14	MIMAT0016859	103005993	103006008	-
hsa-miR-1284	0.1	0.1	Not-Detect	Not-Detect	1	GAAAAGCCAGGGTCTG	chr3	MIMAT0005941	71591171	71591157	+
hsa-miR-548h	0.1	0.1	Not-Detect	Not-Detect	1	GACAAAAACCGCGATT	chr8	MIMAT0005928	26906407	26906392	+
hsa-miR-664*	0.1	0.1	Not-Detect	Not-Detect	1	ATCCAATCATTTTCCCTAGC	chr1	MIMAT0005948	220373913	220373895	+
hsa-miR-188-3p	0.1	0.1	Not-Detect	Not-Detect	1	TGCAAACCCTGCATGTG	chrX	MIMAT0004613	49768167	49768182	-
hsa-miR-374c	0.1	0.1	Not-Detect	Not-Detect	1	AGCACTTAGCAGGTTGT	chrX	MIMAT0018443	73438402	73438417	-
hsa-miR-452	0.1	0.1	Not-Detect	Not-Detect	1	TCAGTTTCCTCTGCAAA	chrX	MIMAT0001635	151128134	151128119	+
hsa-miR-302f	0.1	0.1	Not-Detect	Not-Detect	1	AAACATGGAAGCAATTA	chr18	MIMAT0005932	27878903	27878918	-
hsa-miR-30b*	0.1	0.1	Not-Detect	Not-Detect	1	GAAGTAAACATCCACCTCC	chr8	MIMAT0004589	135812838	135812821	+
hsa-miR-135a	0.1	0.1	Not-Detect	Not-Detect	1	TCACATAGGAATAAAAAGCCATA	chr3	MIMAT0000428	52328273	52328251	+
hsa-miR-567	0.1	0.1	Not-Detect	Not-Detect	1	GTTCTGTCCTGGAAGAACA	chr3	MIMAT0003231	111831668	111831685	-
hsa-miR-33b	0.1	0.1	Not-Detect	Not-Detect	1	GCAATGCAACAGCAATGCAC	chr17	MIMAT0003301	17717184	17717166	+
hsa-miR-876-5p	0.1	0.1	Not-Detect	Not-Detect	1	TGGTGATTCACAAAGAAATCCA	chr9	MIMAT0004924	28863655	28863635	+
hsa-miR-1976	0.1	0.1	Not-Detect	Not-Detect	1	ACAGCAAGGAGGGCA	chr1	MIMAT0009451	26881071	26881084	-
hsa-miR-3926	0.1	0.1	Not-Detect	Not-Detect	1	TCTCTGCCTGCTTTTTG	chr8	MIMAT0018201	12584755	12584771	-
hsa-miR-1184	0.1	0.1	Not-Detect	Not-Detect	1	GGAAGCCATCAAGTCG	chrX	MIMAT0005829	154687245	154687260	-
hsa-miR-3135	0.1	0.1	Not-Detect	Not-Detect	1	CACTGCAGTCTCAGC	chr3	MIMAT0015001	20179074	20179087	-
hsa-miR-1471	0.1	0.1	Not-Detect	Not-Detect	1	ACACCTGGCTCCACA	chr2	MIMAT0007349	232756973	232756960	+
hsa-miR-593*	0.1	0.1	Not-Detect	Not-Detect	1	GCTGAGCAATGCCTG	chr7	MIMAT0003261	127509174	127509188	-
hsa-miR-3617	0.1	0.1	Not-Detect	Not-Detect	1	CCCATCTTGCAACTATG	chr20	MIMAT0017997	44333771	44333756	+
hsa-miR-938	0.1	0.1	Not-Detect	Not-Detect	1	ACTGGGTTCACCTTTA	chr10	MIMAT0004981	29891228	29891214	+
hsa-miR-21*	0.1	0.1	Not-Detect	Not-Detect	1	ACAGCCCATCGACTG	chr17	MIMAT0004494	57918679	57918692	-
hsa-miR-4253	0.1	0.1	Not-Detect	Not-Detect	1	ACCCCCTGGACATGC	chr1	MIMAT0016882	23189709	23189696	+
hsa-miR-124*	0.1	0.1	Not-Detect	Not-Detect	1	ATCAAGGTCCGCTGTGA	chr8	MIMAT0004591	65291735	65291751	-
hsa-miR-517c	0.1	0.1	Not-Detect	Not-Detect	1	ACACTCTAAAAGGATGCAC	chr19	MIMAT0002866	54244626	54244644	-
hsa-miR-3165	0.1	0.1	Not-Detect	Not-Detect	1	TGAGGTCACATTGCATC	chr11	MIMAT0015039	71783304	71783289	+
hsa-miR-624	0.1	0.1	Not-Detect	Not-Detect	1	AGGTAATACCAATACCTTGTG	chr14	MIMAT0004807	31483925	31483906	+
hsa-miR-3662	0.1	0.1	Not-Detect	Not-Detect	1	CATCAGTCACTACTCATCA	chr6	MIMAT0018083	135300553	135300536	+
ebv-miR-BART17-3p	0.1	0.1	Not-Detect	Not-Detect	1	ACTAAGGGGACACCAGGC		MIMAT0003716			
hsv1-miR-H5-3p	0.1	0.1	Not-Detect	Not-Detect	1	CCGGAGGGTTTGGA		MIMAT0008403			
hsa-miR-223*	0.1	0.1	Not-Detect	Not-Detect	1	AACTCAGCTTGTCAAATACACG	chrX	MIMAT0004570	65238738	65238758	-
hsa-miR-4285	0.1	0.1	Not-Detect	Not-Detect	1	ATGAGTCGGACTCGC	chr7	MIMAT0016913	101936383	101936396	-
hsa-miR-1266	0.1	0.1	Not-Detect	Not-Detect	1	AGCCCTGTTCTACAGC	chr15	MIMAT0005920	52569348	52569334	+
hsa-miR-526b*	0.1	0.1	Not-Detect	Not-Detect	1	GCCTCTAAAAGGAAGCAC	chr19	MIMAT0002836	54197701	54197718	-
hsa-miR-16	4674.1999	4674.199867	Detected	Detected	1	CGCCAATATTTACGTGCTG	chr3	MIMAT0000069	160122545	160122563	-
hsa-miR-651	0.1	0.1	Not-Detect	Not-Detect	1	CAAAAGTCAAGCTTATCCT	chrX	MIMAT0003321	8095025	8095042	-
hsa-miR-3685	0.1	0.1	Not-Detect	Not-Detect	1	AGTCTTCAGGTAGGGTAG	chr12	MIMAT0018113	95703709	95703725	-
hbv-miR-B2RC	0.1	0.1	Not-Detect	Not-Detect	1	CTCTCCGCGCCC		MIMAT0012170			
hsa-miR-4319	0.1	0.1	Not-Detect	Not-Detect	1	GTGGCTTTGCTCAGG	chr18	MIMAT0016870	42550073	42550060	+
kshv-miR-K12-1*	0.1	0.1	Not-Detect	Not-Detect	1	GGTTGCAGGAAACAGG		MIMAT0015214			
hsa-miR-296-3p	0.1	0.1	Not-Detect	Not-Detect	1	GGAGAGCCTCCAC	chr20	MIMAT0004679	57392738	57392727	+
hsa-miR-4314	0.1	0.1	Not-Detect	Not-Detect	1	CTGTCCCATTTCCCA	chr17	MIMAT0016868	7991388	7991401	-
hsa-miR-548o	0.1	0.1	Not-Detect	Not-Detect	1	GCAAAAGTAACTGCAGT	chr7	MIMAT0005919	102046296	102046281	+
hsa-miR-675*	0.1	0.1	Not-Detect	Not-Detect	1	TGAGCGGTGAGGGC	chr11	MIMAT0006790	2018052	2018040	+
hsa-miR-1908	0.1	0.1	Not-Detect	Not-Detect	1	GACCAATCGCCGTC	chr11	MIMAT0007881	61582664	61582652	+
hsa-miR-377*	0.1	0.1	Not-Detect	Not-Detect	1	GAATTCACCAAGGGCAACC	chr14	MIMAT0004689	101528397	101528414	-
hsa-let-7e*	0.1	0.1	Not-Detect	Not-Detect	1	GGAAAGCTAGGAGGC	chr11	MIMAT0004485	122017300	122017286	+
hsa-miR-483-3p	0.1	0.1	Not-Detect	Not-Detect	1	AAGACGGGAGGAGAGGAG	chr11	MIMAT0002173	2155431	2155415	+
hsa-let-7i*	0.1	0.1	Not-Detect	Not-Detect	1	AGCAAGGCAGTAGCTTG	chr12	MIMAT0004585	62997533	62997548	-
hsa-miR-487a	0.1	0.1	Not-Detect	Not-Detect	1	AACTGGATGTCCCTGT	chr14	MIMAT0002178	101518838	101518852	-
hsa-miR-138	0.1	0.1	Not-Detect	Not-Detect	1	CGGCCTGATTCACA	chr3	MIMAT0000430	44155735	44155748	-
hsa-miR-346	0.1	0.1	Not-Detect	Not-Detect	1	AGAGGCAGGCATGCGGG	chr10	MIMAT0000773	88024492	88024477	+
hsa-miR-888*	0.1	0.1	Not-Detect	Not-Detect	1	TTCACCCAAAGAGGTGTC	chrX	MIMAT0004917	145076368	145076352	+
hsa-miR-3937	0.1	0.1	Not-Detect	Not-Detect	1	CCCCCATTGCTACAG	chrX	MIMAT0018352	39520539	39520552	-
hsa-miR-1204	0.1	0.1	Not-Detect	Not-Detect	1	ATAATGGAGACCAGGCC	chr8	MIMAT0005868	128808216	128808231	-
hsa-miR-507	0.1	0.1	Not-Detect	Not-Detect	1	TTCACTCCAAAAGGTGCAA	chrX	MIMAT0002879	146312577	146312560	+
hsa-miR-3142	0.1	0.1	Not-Detect	Not-Detect	1	TCTGAAGGTTCAGAAAGG	chr5	MIMAT0015011	159901419	159901436	-
hsa-miR-149*	0.1	0.1	Not-Detect	Not-Detect	1	GCACAGCCCCCGT	chr2	MIMAT0004609	241395482	241395493	-
hsa-miR-601	0.1	0.1	Not-Detect	Not-Detect	1	CTCCTCCAACAATCCTAGA	chr9	MIMAT0003269	126164840	126164823	+
hsa-miR-3622a-3p	0.1	0.1	Not-Detect	Not-Detect	1	ACAGGCATGGGAGGT	chr8	MIMAT0018004	27559251	27559264	-
hsa-miR-95	0.1	0.1	Not-Detect	Not-Detect	1	TGCTCAATAAATACCCGTTG	chr4	MIMAT0000094	8007097	8007079	+
hsa-miR-2115	0.1	0.1	Not-Detect	Not-Detect	1	TCCATCAGGAGTCATGG	chr3	MIMAT0011158	48357891	48357876	+
hsa-miR-4260	0.1	0.1	Not-Detect	Not-Detect	1	TGGGACTCCATGCCC	chr1	MIMAT0016881	209796817	209796804	+
hsa-miR-1249	0.1	0.1	Not-Detect	Not-Detect	1	TGAAGAAGGGGGGGA	chr22	MIMAT0005901	45596896	45596883	+
hsa-miR-518e	0.1	0.1	Not-Detect	Not-Detect	1	CACTCTGAAGGGAAGCGC	chr19	MIMAT0002861	54233149	54233165	-
hsa-miR-3173	0.1	0.1	Not-Detect	Not-Detect	1	TGGCCTGCCTATTTCC	chr14	MIMAT0015048	95604319	95604305	+
hsa-miR-628-5p	0.1	0.1	Not-Detect	Not-Detect	1	CCTCTAGTAAATATGTCAGCA	chr15	MIMAT0004809	55665181	55665162	+
hsa-miR-3667-5p	0.1	0.1	Not-Detect	Not-Detect	1	ACCTTCTCCTCAATGGG	chr22	MIMAT0018089	49937070	49937055	+
ebv-miR-BART2-5p	0.1	0.1	Not-Detect	Not-Detect	1	GCAAGGGCGAATGCA		MIMAT0001000			
hsv1-miR-H8*	0.1	0.1	Not-Detect	Not-Detect	1	TATATACAGGGACCGGG		MIMAT0012598			
hsa-miR-2355-3p	0.1	0.1	Not-Detect	Not-Detect	1	ATCTCCAAACAGCAAGG	chr2	MIMAT0017950	207974785	207974770	+
hsa-miR-4291	0.1	0.1	Not-Detect	Not-Detect	1	AGCTGTTCCTGCTGAA	chr9	MIMAT0016922	96581650	96581664	-
hsa-miR-542-5p	0.1	0.1	Not-Detect	Not-Detect	1	TCTCGTGACATGATGATCCC	chrX	MIMAT0003340	133675408	133675390	+
hsa-miR-369-3p	0.1	0.1	Not-Detect	Not-Detect	1	AAAGATCAACCATGTATTATT	chr14	MIMAT0000721	101531979	101531998	-
hcmv-miR-UL36*	0.1	0.1	Not-Detect	Not-Detect	1	GCACGTTGAAAACACCTGGA		MIMAT0004754			
hsa-miR-4324	0.1	0.1	Not-Detect	Not-Detect	1	TTAAGGTTAGGGTCTCAGG	chr19	MIMAT0016876	49812115	49812098	+
kshv-miR-K12-2	0.1	0.1	Not-Detect	Not-Detect	1	CAGATCGACCCGGAC		MIMAT0002183			
hsa-miR-29a*	0.1	0.1	Not-Detect	Not-Detect	1	CTGAACACCAAAAGAAATCAGT	chr7	MIMAT0004503	130561530	130561510	+
hsa-miR-1295	0.1	0.1	Not-Detect	Not-Detect	1	TCACCCAGATCTGCG	chr1	MIMAT0005885	171070937	171070924	+
hsa-miR-548w	0.1	0.1	Not-Detect	Not-Detect	1	AGGCAAAAACCGCAG	chr16	MIMAT0015060	26036576	26036589	-
hsa-miR-708*	0.1	0.1	Not-Detect	Not-Detect	1	CTAGAAGCTCACAGTCTA	chr11	MIMAT0004927	79113143	79113127	+
hsa-miR-1911	0.1	0.1	Not-Detect	Not-Detect	1	CCCAACAGACATGGC	chrX	MIMAT0007885	113997764	113997777	-
hsa-miR-380	0.1	0.1	Not-Detect	Not-Detect	1	AAGATGTGGACCATATTACATA	chr14	MIMAT0000735	100561146	100561167	-
hsa-miR-759	0.1	0.1	Not-Detect	Not-Detect	1	GTCAAAATTGTTTGCACT	chr13	MIMAT0010497	53384213	53384229	-
hsa-miR-1915*	0.1	0.1	Not-Detect	Not-Detect	1	GGCCCGGGCAGC	chr10	MIMAT0007891	21785523	21785513	+
hsa-miR-3908	0.1	0.1	Not-Detect	Not-Detect	1	AAACAGTCTACCTACATTGC	chr12	MIMAT0018182	124020963	124020981	-
hsa-miR-103-2*	0.1	0.1	Not-Detect	Not-Detect	1	CAAGGCAGCACTGTAA	chr20	MIMAT0009196	3898159	3898173	-
hsa-miR-491-3p	0.1	0.1	Not-Detect	Not-Detect	1	GTAGAAGGGAATCTTGCATA	chr9	MIMAT0004765	20716156	20716174	-
hsa-miR-3119	0.1	0.1	Not-Detect	Not-Detect	1	GCCATCAAAGTTAAAAGC	chr1	MIMAT0014981	170120529	170120546	-
hsa-miR-141	0.1	0.1	Not-Detect	Not-Detect	1	CCATCTTTACCAGACAG	chr12	MIMAT0000432	7073324	7073339	-
hsa-miR-579	0.1	0.1	Not-Detect	Not-Detect	1	AATCGCGGTTTATACCAAATG	chr5	MIMAT0003244	32394567	32394548	+
hsa-miR-3605-3p	0.1	0.1	Not-Detect	Not-Detect	1	CTAGAGGACAGGTAACAC	chr1	MIMAT0017982	33798081	33798065	+
hsa-miR-9	0.1	0.1	Not-Detect	Not-Detect	1	TCATACAGCTAGATAACCA	chr1	MIMAT0000441	156390170	156390152	+
hsa-miR-200c	0.1	0.1	Not-Detect	Not-Detect	1	TCCATCATTACCCGG	chr12	MIMAT0000617	7072914	7072927	-

hsa-miR-3944	0.1	0.1	Not-Detect	Not-Detect	1	CCGGAGCAGCAGG	chr10	MIMAT0018360	135185144	135185133	+
hsa-miR-122*	0.1	0.1	Not-Detect	Not-Detect	1	TATTTAGTGTGATAATGGCGTT	chr18	MIMAT0004590	56118357	56118377	−
hsa-miR-511	0.1	0.1	Not-Detect	Not-Detect	1	TGACTGCAGAGCAAAAGA	chr10	MIMAT0002808	17887125	17887142	−
hsa-miR-3148	0.1	0.1	Not-Detect	Not-Detect	1	AAGCACACACCAGTTTT	chr8	MIMAT0015021	29814818	29814803	+
hsa-miR-1537	0.1	0.1	Not-Detect	Not-Detect	1	ACAACTGTAACTAGACGG	chr1	MIMAT0007399	236016360	236016344	+
hsa-miR-608	0.1	0.1	Not-Detect	Not-Detect	1	ACGGAGCTGTCCCAAC	chr10	MIMAT0003276	102734767	102734781	−
hsa-miR-3647-3p	0.1	0.1	Not-Detect	Not-Detect	1	GATCAGACACACAAAAATT	chr16	MIMAT0018067	70563468	70563485	−
hsa-miR-214*	0.1	0.1	Not-Detect	Not-Detect	1	GCACAGCAAGTGTAGACA	chr1	MIMAT0004564	172107988	172107972	+
hsa-miR-4267	0.1	0.1	Not-Detect	Not-Detect	1	GTGCCACCGAGCT	chr2	MIMAT0016893	110827563	110827552	+
hsa-miR-1255b	0.1	0.1	Not-Detect	Not-Detect	1	AACCACTTTCTTTGCTCA	chr1	MIMAT0005945	167967907	167967924	−
hsa-miR-519e	0.1	0.1	Not-Detect	Not-Detect	1	AACACTCTAAAAGGAGGCACTT	chr19	MIMAT0002829	54183246	54183266	−
hsa-miR-3180	0.1	0.1	Not-Detect	Not-Detect	1	CTCCGGAAGCTCCG	chr16	MIMAT0018178	2186031	2186018	+
hsa-miR-634	0.1	0.1	Not-Detect	Not-Detect	1	GTCCAAAGTTGGGGTGCT	chr17	MIMAT0003304	64783255	64783271	−
hsa-miR-3672	0.1	0.1	Not-Detect	Not-Detect	1	AAGATGTTTTACATGAGTCTC	chrX	MIMAT0018095	120504843	120504862	−
ebv-miR-BART3*	0.1	0.1	Not-Detect	Not-Detect	1	AGCACAACACTAACACTA		MIMAT0003410			
hsv2-miR-H21	0.1	0.1	Not-Detect	Not-Detect	1	CCGTAGACAGCATGAC		MIMAT0014703			
hsa-miR-4298	0.1	0.1	Not-Detect	Not-Detect	1	CTGCCTCCTCCTCC	chr11	MIMAT0016852	1880725	1880713	+
hsa-miR-548a-5p	0.1	0.1	Not-Detect	Not-Detect	1	GGTAAAACTCGCAATTACTTTT	chr8	MIMAT0004803	105496642	105496622	+
hsa-miR-655	0.1	0.1	Not-Detect	Not-Detect	1	AAAGAGGTTAACCATGTATTA	chr14	MIMAT0003331	101515949	101515968	−
hsa-miR-371-3p	0.1	0.1	Not-Detect	Not-Detect	1	ACACTCAAAAGATGGCGGC	chr19	MIMAT0000723	54290974	54290992	−
hcmv-miR-US33-3p	0.1	0.1	Not-Detect	Not-Detect	1	TGGATGTGCTCGGACC		MIMAT0004756			
hsa-miR-4330	0.1	0.1	Not-Detect	Not-Detect	1	GCAAGGCTCTGATCTG	chrX	MIMAT0016924	150336774	150336788	−
kshv-miR-K12-5*	0.1	0.1	Not-Detect	Not-Detect	1	CCTTAGGGCACCAGG		MIMAT0015218			
hsa-miR-301a	0.1	0.1	Not-Detect	Not-Detect	1	GCTTTGACAATACTATTGCAC	chr17	MIMAT0000688	54583351	54583331	+
hsa-miR-1303	0.1	0.1	Not-Detect	Not-Detect	1	AGAGCAAGACCCCGT	chr5	MIMAT0005891	154065395	154065408	−
hsa-miR-550b	0.1	0.1	Not-Detect	Not-Detect	1	CAGTGCCTGAGGGA	chr7	MIMAT0018445	30329486	30329473	+
hsa-miR-325	0.1	0.1	Not-Detect	Not-Detect	1	ACACTTACTGGACACCTAC	chrX	MIMAT0000771	76225866	76225849	+
hsa-miR-331-5p	0.1	0.1	Not-Detect	Not-Detect	1	GGATCCCTGGGACC	chr12	MIMAT0004700	95702230	95702242	−
hsa-miR-767-3p	0.1	0.1	Not-Detect	Not-Detect	1	AGAAACCATGGGGTATGA	chrX	MIMAT0003883	151561975	151561959	+
hsa-miR-3915	0.1	0.1	Not-Detect	Not-Detect	1	AATAAGACCATCTTTTCCTC	chrX	MIMAT0018189	32601814	32601796	+
hsa-miR-496	0.1	0.1	Not-Detect	Not-Detect	1	GAGATTGGCCATGTAAT	chr14	MIMAT0002818	100596723	100596739	−
hsa-miR-3126-3p	0.1	0.1	Not-Detect	Not-Detect	1	TCTGTGTGACGGATGC	chr2	MIMAT0015377	69330866	69330880	−
hsa-miR-585	0.1	0.1	Not-Detect	Not-Detect	1	TAGCATACAGATACGCCCA	chr5	MIMAT0003250	168690683	168690666	+
hsa-miR-92a-1*	0.1	0.1	Not-Detect	Not-Detect	1	AGCATTGCAACCGATCCC	chr13	MIMAT0004507	92003584	92003600	−
hsa-miR-205*	0.1	0.1	Not-Detect	Not-Detect	1	GAACTTCACTCCACTGA	chr1	MIMAT0009197	209605553	209605568	−
hsa-miR-412	0.1	0.1	Not-Detect	Not-Detect	1	ACGGCTAGTGGACCAG	chr14	MIMAT0002170	101531845	101531859	−
hsa-miR-1227	0.1	0.1	Not-Detect	Not-Detect	1	CTGGGGAAAAGGGTGG	chr19	MIMAT0005580	2234148	2234134	+
hsa-miR-514	0.1	0.1	Not-Detect	Not-Detect	1	TCTACTCACAGAAGTGTC	chrX	MIMAT0002883	146360843	146360826	+
hsa-miR-3154	0.1	0.1	Not-Detect	Not-Detect	1	TCTGCTCCCAACTCC	chr9	MIMAT0015028	131007300	131007287	+
hsa-miR-615-3p	0.1	0.1	Not-Detect	Not-Detect	1	AAGAGGGAGACCCAGGC	chr12	MIMAT0003283	54427800	54427815	−
ebv-miR-BART10	0.1	0.1	Not-Detect	Not-Detect	1	ACAGCCAACTCCATGG		MIMAT0003420			
hsv1-miR-H14-5p	0.1	0.1	Not-Detect	Not-Detect	1	CCTGAGCCAGGGAC		MIMAT0014691			
hsa-miR-218-2*	0.1	0.1	Not-Detect	Not-Detect	1	CGCGGTGCTTGACA	chr5	MIMAT0004566	168195238	168195226	+
hsa-miR-4274	0.1	0.1	Not-Detect	Not-Detect	1	CAGGGGGAGGGAC	chr4	MIMAT0016906	7461824	7461835	−
hsa-miR-125b-1*	0.1	0.1	Not-Detect	Not-Detect	1	AGCTCCCAAGAGCCT	chr11	MIMAT0004592	121970540	121970527	+
hsa-miR-520e	0.1	0.1	Not-Detect	Not-Detect	1	CCCTCAAAAAGGAAGCACT	chr19	MIMAT0002825	54179021	54179038	−
hsa-miR-3185	0.1	0.1	Not-Detect	Not-Detect	1	CCGCAGACCGACC	chr17	MIMAT0015065	46801799	46801788	+
hsa-miR-641	0.1	0.1	Not-Detect	Not-Detect	1	GAGGTGACTCTATCCTATG	chr19	MIMAT0003311	40788488	40788471	+
hsa-miR-3678-3p	0.1	0.1	Not-Detect	Not-Detect	1	CCGGTCCGTACAAA	chr17	MIMAT0018103	73402227	73402239	−
ebv-miR-BART7	0.1	0.1	Not-Detect	Not-Detect	1	CCCTGGACACTGGAC		MIMAT0003416			
hsv2-miR-H4-3p	0.1	0.1	Not-Detect	Not-Detect	1	GAGTTCGCTAGGCAAG		MIMAT0010204			
hsa-miR-26a-2*	0.1	0.1	Not-Detect	Not-Detect	1	GAAACAAGTAATCAAGAATAGG	chr12	MIMAT0004681	58218464	58218444	+
hsa-miR-4305	0.1	0.1	Not-Detect	Not-Detect	1	GAACTGGAGGTGTCTAG	chr13	MIMAT0016857	40238198	40238183	+
hsa-miR-548d-5p	0.1	0.1	Not-Detect	Not-Detect	1	GGCAAAAACCACAATTACTTTT	chr8	MIMAT0004812	124360319	124360298	+
hsa-miR-662	0.1	0.1	Not-Detect	Not-Detect	1	CTGCTGGGCCACAACG	chr16	MIMAT0003325	820249	820263	−
hiv1-miR-TAR-3p	0.1	0.1	Not-Detect	Not-Detect	1	TGGGTTCCCTAGTTAGC		MIMAT0006017			
hsa-miR-450a	0.1	0.1	Not-Detect	Not-Detect	1	ATATTAGGAACACATCGCAA	chrX	MIMAT0001545	133674409	133674390	+
kshv-miR-K12-9	0.1	0.1	Not-Detect	Not-Detect	1	TTACGCAGCTGCGTA		MIMAT0002185			
hsa-miR-302c*	0.1	0.1	Not-Detect	Not-Detect	1	CAGCAGGTACCCCC	chr4	MIMAT0000716	113569547	113569535	+
hsa-miR-555	0.1	0.1	Not-Detect	Not-Detect	1	ATCAGAGGTTCAGCTTAC	chr1	MIMAT0003219	155316221	155316205	+
hsa-miR-1324	0.1	0.1	Not-Detect	Not-Detect	1	GAAAGTGCATAGAATTCTGT	chr3	MIMAT0005956	75679979	75679997	−
hsa-miR-562	0.1	0.1	Not-Detect	Not-Detect	1	GCAAATGGTACAGCTACT	chr2	MIMAT0003226	233037426	233037442	−
hsa-miR-339-3p	0.1	0.1	Not-Detect	Not-Detect	1	CGGCTCTGTCGTCG	chr7	MIMAT0004702	1062640	1062628	+
hsa-miR-196b*	0.1	0.1	Not-Detect	Not-Detect	1	GAAGGCAGTGTCGTG	chr7	MIMAT0009201	27209169	27209156	+
hsa-miR-3922	0.1	0.1	Not-Detect	Not-Detect	1	AAAGAGTCAAGTCAAGGC	chr12	MIMAT0018197	104985477	104985493	−
hsa-miR-3131	0.1	0.1	Not-Detect	Not-Detect	1	AAGGCCCTTCCACC	chr2	MIMAT0014996	219923435	219923423	+
hsa-miR-146b-3p	0.1	0.1	Not-Detect	Not-Detect	1	CCAGAACTGAGTCCAC	chr10	MIMAT0004766	104196320	104196334	−
hsa-miR-3614-5p	0.1	0.1	Not-Detect	Not-Detect	1	GGGCAGCCTTCAGA	chr17	MIMAT0017992	54968667	54968655	+
hsa-miR-934	0.1	0.1	Not-Detect	Not-Detect	1	CCAGTGTCTCCAG	chrX	MIMAT0004977	135633061	135633072	−
hsa-miR-20a*	0.1	0.1	Not-Detect	Not-Detect	1	CTTTAAGTGCTCATAATGCAG	chr13	MIMAT0004493	92003364	92003383	−
hsa-miR-1236	0.1	0.1	Not-Detect	Not-Detect	1	CTGGAGAGACAAGGGG	chr6	MIMAT0005591	31924717	31924703	+
hsa-miR-516b	0.1	0.1	Not-Detect	Not-Detect	1	AAAGTGCTTCTTACCTCCA	chr19	MIMAT0002859	54228714	54228732	−
hsa-miR-3161	0.1	0.1	Not-Detect	Not-Detect	1	ATCTGGGCCTCTGTTC	chr11	MIMAT0015035	48118351	48118365	−
hsa-miR-620	0.1	0.1	Not-Detect	Not-Detect	1	ATTTCTATATCTATCTCCAT	chr12	MIMAT0003289	116586444	116586426	+
hsa-miR-3658	0.1	0.1	Not-Detect	Not-Detect	1	ATCTCCATGGTGTTTTCT	chr1	MIMAT0018078	165877165	165877181	−
ebv-miR-BART14	0.1	0.1	Not-Detect	Not-Detect	1	ATCCCTACTACTGCAGCA		MIMAT0003426			
hsv1-miR-H3	0.1	0.1	Not-Detect	Not-Detect	1	GTCCCAACCGCAC		MIMAT0008400			
hsa-miR-221*	0.1	0.1	Not-Detect	Not-Detect	1	AAATCTACATTGTATGCCAGG	chrX	MIMAT0004568	45605630	45605611	+
hsa-miR-1262	0.1	0.1	Not-Detect	Not-Detect	1	ATCCTTCTACAAATTCACCC	chr1	MIMAT0005914	68649235	68649217	+
hsa-miR-524-3p	0.1	0.1	Not-Detect	Not-Detect	1	ACTCCAAAGGGAAGCGCC	chr19	MIMAT0002850	54214311	54214328	−
hsa-miR-3191	0.1	0.1	Not-Detect	Not-Detect	1	CTGTCTGGCCAGCT	chr19	MIMAT0015075	47730268	47730256	+
hsa-miR-647	0.1	0.1	Not-Detect	Not-Detect	1	GAAGGAAGTGAGTGCAG	chr20	MIMAT0003317	62574019	62574004	+
hsa-miR-3681*	0.1	0.1	Not-Detect	Not-Detect	1	AGTAGTGGATGAAGCACT	chr2	MIMAT0018109	12339305	12339321	−
ebv-miR-BHRF1-2	0.1	0.1	Not-Detect	Not-Detect	1	TCAATTTCTGCCGCAA		MIMAT0000997			
hsv2-miR-H9-3p	0.1	0.1	Not-Detect	Not-Detect	1	GAGGAGGAGACCGAG		MIMAT0014352			
hsa-miR-28-3p	0.1	0.1	Not-Detect	Not-Detect	1	TCCAGGAGCTCACA	chr3	MIMAT0004502	188406631	188406643	−
hsa-miR-4310	0.1	0.1	Not-Detect	Not-Detect	1	GGGACATGAATGCTGC	chr15	MIMAT0016862	42158739	42158725	+
hsa-miR-1287	0.1	0.1	Not-Detect	Not-Detect	1	GACTCGAACCACTGAT	chr10	MIMAT0005878	100155011	100154997	+
hsa-miR-548k	0.1	0.1	Not-Detect	Not-Detect	1	AGCAAAATCCGCAAGTA	chr11	MIMAT0005882	70130098	70130113	−
hsa-miR-3199	0.1	0.1	Not-Detect	Not-Detect	1	AACTTTCTCCTAAGGCAG	chr22	MIMAT0015084	28316528	28316545	−
hsa-miR-18a*	0.1	0.1	Not-Detect	Not-Detect	1	CCAGAAGGAGCACTTA	chr13	MIMAT0002891	92003059	92003073	−
hsa-miR-376a*	0.1	0.1	Not-Detect	Not-Detect	1	TACTCATAGAAGGAGAATCTAC	chr14	MIMAT0003386	101507126	101507146	−
hsa-let-7c*	0.1	0.1	Not-Detect	Not-Detect	1	TAACTCCCAGGGTGTA	chr21	MIMAT0004483	17912187	17912201	−
hsa-miR-3074	0.1	0.1	Not-Detect	Not-Detect	1	CGGTGCCTACTGAGC	chr9	MIMAT0015027	97848366	97848353	+
hsa-miR-30c-2*	0.1	0.1	Not-Detect	Not-Detect	1	AGAGTAAACAGCCTTCTCC	chr6	MIMAT0004550	72086730	72086713	+
hsa-miR-135b*	0.1	0.1	Not-Detect	Not-Detect	1	CCCATGGCTTTTAGC	chr1	MIMAT0004698	205417505	205417492	+
hsa-miR-570	0.1	0.1	Not-Detect	Not-Detect	1	GCAAAGGTAATTGCTGTTTT	chr3	MIMAT0003235	195426334	195426352	−
hsa-miR-885-3p	0.1	0.1	Not-Detect	Not-Detect	1	TATCCACTACACCCCG	chr3	MIMAT0004948	10436236	10436222	+
hsa-miR-3929	0.1	0.1	Not-Detect	Not-Detect	1	AGTGGTCTACTCACATCA	chr18	MIMAT0018206	33514105	33514089	+
hsa-miR-1197	0.1	0.1	Not-Detect	Not-Detect	1	AGAAGTAGACCATGTGTC	chr14	MIMAT0005955	101491961	101491977	−
hsa-miR-504	0.1	0.1	Not-Detect	Not-Detect	1	GATAGAGTGCAGACCAG	chrX	MIMAT0002875	137749905	137749890	+
hsa-miR-3138	0.1	0.1	Not-Detect	Not-Detect	1	ACTCCCTCTACCTCACT	chr4	MIMAT0015006	10080305	10080290	+
hsa-miR-148a*	0.1	0.1	Not-Detect	Not-Detect	1	AGTCGGAGTGTCTCAG	chr7	MIMAT0004549	25989565	25989551	+
hsa-miR-597	0.1	0.1	Not-Detect	Not-Detect	1	ACAGTGGTCATCGAGTG	chr8	MIMAT0003265	9599203	9599218	−
hsa-miR-941	0.1	0.1	Not-Detect	Not-Detect	1	GCACATGTGCACACAG	chr20	MIMAT0004984	62550855	62550870	−
hsa-miR-2110	0.1	0.1	Not-Detect	Not-Detect	1	CACTCAGCGGCCG	chr10	MIMAT0010133	115933892	115933881	+
hsa-miR-4256	0.1	0.1	Not-Detect	Not-Detect	1	ACCTTCATCAGGTCAGA	chr1	MIMAT0016877	113004419	113004404	+
hsa-miR-1245	0.1	0.1	Not-Detect	Not-Detect	1	ATGTAGGCCTTTAGATCAC	chr2	MIMAT0005897	189842865	189842882	−
hsa-miR-518b	0.1	0.1	Not-Detect	Not-Detect	1	ACCTCTAAAGGGGAGCG	chr19	MIMAT0002844	54206047	54206062	−
hsa-miR-3168	0.1	0.1	Not-Detect	Not-Detect	1	GTCTGACTGTAGAACTC	chr13	MIMAT0015043	41675179	41675164	+

hsa-miR-3664	0.1	0.1	Not-Detect	Not-Detect	1	ACTCATGAGTGAAGACAG	chr11	MIMAT0018086	70718416	70718400	+
ebv-miR-BART18-5p	0.1	0.1	Not-Detect	Not-Detect	1	TGTATAGGAAGTGCGAACTTG		MIMAT0003717			
hsa-miR-2276	0.1	0.1	Not-Detect	Not-Detect	1	CCTCGCCTCTGACA	chr13	MIMAT0011775	24736617	24736629	-
hsa-miR-4288	0.1	0.1	Not-Detect	Not-Detect	1	GGAAACTCAGCAGACA	chr8	MIMAT0016918	28362689	28362675	+
hsa-miR-1269	0.1	0.1	Not-Detect	Not-Detect	1	CCAGTAGCACGGCT	chr4	MIMAT0005923	67142617	67142629	-
hsa-miR-539	0.1	0.1	Not-Detect	Not-Detect	1	ACACACCAAGGATAATTTCTCC	chr14	MIMAT0003163	101513667	101513687	-
hsa-miR-3688	0.1	0.1	Not-Detect	Not-Detect	1	AGAGTGGCAAAGTCTTTC	chr4	MIMAT0018116	160050034	160050018	+
hcmv-miR-UL148D	0.1	0.1	Not-Detect	Not-Detect	1	CGGTGAAGAAGGGGA		MIMAT0001578			
hsa-miR-4320	0.1	0.1	Not-Detect	Not-Detect	1	AGGAAGCTACAGAATCCC	chr18	MIMAT0016871	47652923	47652907	+
kshv-miR-K12-11	0.1	0.1	Not-Detect	Not-Detect	1	TCGGACACAGGCTAA		MIMAT0002181			
hsa-miR-298	0.1	0.1	Not-Detect	Not-Detect	1	TGGGAGAACCTCCCTG	chr20	MIMAT0004901	57393314	57393300	+
hsa-miR-1291	0.1	0.1	Not-Detect	Not-Detect	1	ACTGCTGGTCTTCAGTC	chr12	MIMAT0005881	49048263	49048248	+
hsa-miR-548s	0.1	0.1	Not-Detect	Not-Detect	1	AAAATAACTGCAGTTTTGGC	chr2	MIMAT0014987	11907625	11907643	-
hsa-miR-190b	0.1	0.1	Not-Detect	Not-Detect	1	AACCCAATATCAAACATATCA	chr1	MIMAT0004929	154166171	154166152	+
hsa-miR-378b	0.1	0.1	Not-Detect	Not-Detect	1	TTCTGCCTCCAAGTCC	chr3	MIMAT0014999	10371950	10371964	-
hsa-let-7f-2*	0.1	0.1	Not-Detect	Not-Detect	1	GGAAAGACAGTAGACTGTATAG	chrX	MIMAT0004487	53584231	53584211	+
hsa-miR-100*	0.1	0.1	Not-Detect	Not-Detect	1	CATACCTATAGATACAAGCTT	chr11	MIMAT0004512	122023005	122022986	+
hsa-miR-488*	0.1	0.1	Not-Detect	Not-Detect	1	TTGAGAGTGCCATTATCTGG	chr1	MIMAT0002804	175265155	175265136	+
hsa-miR-3115	0.1	0.1	Not-Detect	Not-Detect	1	ACCAACTAGTAAACCCATAT	chr1	MIMAT0014977	23370804	23370822	-
hsa-miR-139-3p	0.1	0.1	Not-Detect	Not-Detect	1	ACTCCAACAGGGCCG	chr11	MIMAT0004552	72326171	72326158	+
hsa-miR-576-3p	0.1	0.1	Not-Detect	Not-Detect	1	GATTCCAATTTTTCCACAT	chr4	MIMAT0004796	110409912	110409929	-
hsa-miR-34b	0.1	0.1	Not-Detect	Not-Detect	1	ATGGCAGTGGAGTTAGT	chr11	MIMAT0004676	111383718	111383733	-
hsa-miR-891a	0.1	0.1	Not-Detect	Not-Detect	1	TCAGTGGCTCAGGT	chrX	MIMAT0004902	145109343	145109331	+
hsa-miR-200a	0.1	0.1	Not-Detect	Not-Detect	1	ACATCGTTACCAGACAGT	chr1	MIMAT0000682	1103301	1103317	-
hsa-miR-3940	0.1	0.1	Not-Detect	Not-Detect	1	AAGTGGGCTGGGATC	chr19	MIMAT0018356	6416498	6416485	+
hsa-miR-1207-3p	0.1	0.1	Not-Detect	Not-Detect	1	GAAATGAGGGCCAGC	chr8	MIMAT0005872	129061453	129061466	-
hsa-miR-509-3-5p	0.1	0.1	Not-Detect	Not-Detect	1	CATGATTGCCACGTCTG	chrX	MIMAT0004975	146341200	146341185	+
hsa-miR-3144-5p	0.1	0.1	Not-Detect	Not-Detect	1	CTATATATCTCTTTGGTCCC	chr6	MIMAT0015014	120336340	120336358	-
hsa-miR-604	0.1	0.1	Not-Detect	Not-Detect	1	GTCCTGAATTCCGCAGC	chr10	MIMAT0003272	29834011	29833996	+
hsa-miR-3622b-5p	0.1	0.1	Not-Detect	Not-Detect	1	TCACCTGACCTCCCA	chr8	MIMAT0018005	27559231	27559218	+
hsa-miR-2116*	0.1	0.1	Not-Detect	Not-Detect	1	GGGAGTTCTTGGCATG	chr15	MIMAT0011161	59463452	59463438	+
hsa-miR-4263	0.1	0.1	Not-Detect	Not-Detect	1	GGCCAAGGCACTTAG	chr2	MIMAT0016898	28219293	28219306	-
hsa-miR-1252	0.1	0.1	Not-Detect	Not-Detect	1	TAAATGAATTCAATTTCCTTCT	chr12	MIMAT0005944	79813042	79813062	-
hsa-miR-518f*	0.1	0.1	Not-Detect	Not-Detect	1	GAGAAAGTGCTTCCCT	chr19	MIMAT0002841	54203290	54203305	-
hsa-miR-3176	0.1	0.1	Not-Detect	Not-Detect	1	CCGGTAGTCCCAGG	chr16	MIMAT0015053	593342	593354	-
hsa-miR-630	0.1	0.1	Not-Detect	Not-Detect	1	ACCTTCCCTGGTACAGA	chr15	MIMAT0003299	72879624	72879639	-
hsa-miR-367	0.1	0.1	Not-Detect	Not-Detect	1	TCACCATTGCTAAAGTGCAAT	chr4	MIMAT0000719	113569094	113569075	+
ebv-miR-BART21-3p	0.1	0.1	Not-Detect	Not-Detect	1	AAACACCAGTGGGCA		MIMAT0010131			
hsv2-miR-H13	0.1	0.1	Not-Detect	Not-Detect	1	CAGTGCTCGCACTTT		MIMAT0014700			
hsa-miR-23a*	0.1	0.1	Not-Detect	Not-Detect	1	AAATCCCATCCCCAG	chr19	MIMAT0004496	13947430	13947417	+
hsa-miR-4294	0.1	0.1	Not-Detect	Not-Detect	1	CCCTGCTGTAGACTCC	chr10	MIMAT0016849	50193583	50193569	+
hsa-miR-1273c	0.1	0.1	Not-Detect	Not-Detect	1	GACAGGGTCTCGTTTT	chr6	MIMAT0015017	155174510	155174524	-
hsa-miR-544b	0.1	0.1	Not-Detect	Not-Detect	1	TTAGAAATGCACAACCTCA	chr3	MIMAT0015004	124451337	124451354	-
hsa-miR-181c*	0.1	0.1	Not-Detect	Not-Detect	1	GTCCACTCAACGGTCG	chr19	MIMAT0004559	13985584	13985598	-
hsa-miR-3691	0.1	0.1	Not-Detect	Not-Detect	1	GTACCGAGTCTCCATC	chr6	MIMAT0018120	5148503	5148489	+
hcmv-miR-US25-1	0.1	0.1	Not-Detect	Not-Detect	1	GGTCCGAGCCACTG		MIMAT0001581			
hsa-miR-4327	0.1	0.1	Not-Detect	Not-Detect	1	CCAGTCCCCCATGC	chr21	MIMAT0016889	31747640	31747628	+
kshv-miR-K12-3*	0.1	0.1	Not-Detect	Not-Detect	1	TGTCACATTCTGTGACCG		MIMAT0002194			
hsa-miR-29b-2*	0.1	0.1	Not-Detect	Not-Detect	1	CTAAGCCACCATGTGA	chr1	MIMAT0004515	207975819	207975805	+
hsa-miR-1298	0.1	0.1	Not-Detect	Not-Detect	1	TACATCTGGACAGCCG	chrX	MIMAT0005800	113949674	113949688	-
hsa-miR-548z	0.1	0.1	Not-Detect	Not-Detect	1	TGCAAAAGTAATTGCGG	chr12	MIMAT0018446	65016364	65016349	+
hsa-miR-323b-3p	0.1	0.1	Not-Detect	Not-Detect	1	AAGAGGTCGACCGTG	chr14	MIMAT0015050	101522614	101522627	-
hsa-miR-1913	0.1	0.1	Not-Detect	Not-Detect	1	TGGCAGCAGCGGA	chr6	MIMAT0007888	166922911	166922900	+
hsa-miR-193a-3p	0.1	0.1	Not-Detect	Not-Detect	1	ACTGGGACTTTGTAGGC	chr17	MIMAT0000459	29887075	29887090	-
hsa-miR-3911	0.1	0.1	Not-Detect	Not-Detect	1	TGCCTCCTCCAGGA	chr9	MIMAT0018185	130452998	130452986	+
hsa-miR-105*	0.1	0.1	Not-Detect	Not-Detect	1	TAGCACATGCTCAAACATCC	chrX	MIMAT0004516	151560762	151560743	+
hsa-miR-493	0.1	0.1	Not-Detect	Not-Detect	1	CCTGGCACACAGTAG	chr14	MIMAT0003161	101335461	101335474	-
hsa-miR-3122	0.1	0.1	Not-Detect	Not-Detect	1	AAGACCGTCCTCTTGT	chr1	MIMAT0014984	212250971	212250985	-
hsa-miR-582-3p	0.1	0.1	Not-Detect	Not-Detect	1	GGTTCAGTTGTTCAACC	chr5	MIMAT0004797	58999505	58999490	+
hsa-miR-3607-3p	0.1	0.1	Not-Detect	Not-Detect	1	CATCAGAAAGCGTTTACA	chr5	MIMAT0017985	85916367	85916383	-
hsa-miR-921	0.1	0.1	Not-Detect	Not-Detect	1	GAATCCTGGTTCTGTCC	chr1	MIMAT0004971	166124005	166123990	+
hsa-miR-202*	0.1	0.1	Not-Detect	Not-Detect	1	CAAAGAAGTATATGCATAGGA	chr10	MIMAT0002810	135061063	135061044	+
hsa-miR-409-5p	0.1	0.1	Not-Detect	Not-Detect	1	ATGCAAAGTTGCTCGGGTA	chr14	MIMAT0001638	101531656	101531673	-
hsa-miR-1225-3p	0.1	0.1	Not-Detect	Not-Detect	1	CTGGGGGCGGC	chr16	MIMAT0005573	2140285	2140276	+
hsa-miR-513a-3p	0.1	0.1	Not-Detect	Not-Detect	1	CCTTCTCAGAAAGGTGAA	chrX	MIMAT0004777	146295074	146295057	+
hsa-miR-3150b	0.1	0.1	Not-Detect	Not-Detect	1	CCAACCTCGACGATC	chr8	MIMAT0018194	96085211	96085198	+
hsa-miR-154	0.1	0.1	Not-Detect	Not-Detect	1	CGAAGGCAACACGGATAAC	chr14	MIMAT0000452	101526110	101526127	-
hsa-miR-611	0.1	0.1	Not-Detect	Not-Detect	1	GTCAGACCCCGAGGG	chr11	MIMAT0003279	61560028	61560015	+
hsa-miR-3649	0.1	0.1	Not-Detect	Not-Detect	1	CTTAGACACTCAGGTCC	chr12	MIMAT0018069	1769545	1769530	+
bkv-miR-B1-3p	0.1	0.1	Not-Detect	Not-Detect	1	GACTCTGGACATGGATC		MIMAT0009150			
hsv1-miR-H11	0.1	0.1	Not-Detect	Not-Detect	1	GCGTTCGCACTTTGT		MIMAT0014688			
hsa-miR-216b	0.1	0.1	Not-Detect	Not-Detect	1	TCACATTTGCCTGCAG	chr2	MIMAT0004959	56227880	56227866	+
hsa-miR-1258	0.1	0.1	Not-Detect	Not-Detect	1	TTCCACGACCTAATCCT	chr2	MIMAT0005909	180725626	180725611	+
hsa-miR-520a-5p	0.1	0.1	Not-Detect	Not-Detect	1	AGAAAGTACTTCCCTCTGG	chr19	MIMAT0002833	54194151	54194169	-
hsa-miR-3181	0.1	0.1	Not-Detect	Not-Detect	1	CCGGCGCCGAG	chr16	MIMAT0015061	50776233	50776242	-
hsa-miR-637	0.1	0.1	Not-Detect	Not-Detect	1	ACGCAGAGCCCGAAAGC	chr19	MIMAT0003307	3961495	3961480	+
hsa-miR-3675-3p	0.1	0.1	Not-Detect	Not-Detect	1	TTGGGGGAGTTCCTTA	chr1	MIMAT0018099	17185507	17185493	+
ebv-miR-BART5	0.1	0.1	Not-Detect	Not-Detect	1	CGATGGGCAGCTATA		MIMAT0003413			
hsv2-miR-H23*	0.1	0.1	Not-Detect	Not-Detect	1	GAGTAGTACCGCGAAG		MIMAT0014706			
hsa-miR-25	7231.2865	7231.2865	Detected	Detected	1	TCAGACCGAGACAAGTGC	chr7	MIMAT0000081	99691255	99691239	+
hsa-miR-4301	0.1	0.1	Not-Detect	Not-Detect	1	TCACAAGTGAAGTAGTGG	chr11	MIMAT0016850	113320774	113320758	+
hsa-miR-1277	0.1	0.1	Not-Detect	Not-Detect	1	AAAATACATATATATCTACGTA	chrX	MIMAT0005933	117520404	117520424	-
hsa-miR-548b-5p	0.1	0.1	Not-Detect	Not-Detect	1	GGCCAAAACCACAATTACTTT	chr6	MIMAT0004798	119390257	119390238	+
hsa-miR-658	0.1	0.1	Not-Detect	Not-Detect	1	ACCAACGGACCTACTTCCCT	chr22	MIMAT0003336	38240363	38240345	+
hsa-miR-3714	0.1	0.1	Not-Detect	Not-Detect	1	ACAGGGGAGCACTGC	chr3	MIMAT0018165	16974696	16974709	-
hcmv-miR-US5-1	0.1	0.1	Not-Detect	Not-Detect	1	ACGCTCTCGTCAGGC		MIMAT0001579			
hsa-miR-449b	0.1	0.1	Not-Detect	Not-Detect	1	GCCAGCTAACAATACACTG	chr5	MIMAT0003327	54466510	54466493	+
kshv-miR-K12-7	0.1	0.1	Not-Detect	Not-Detect	1	GCGCCAGCAACATG		MIMAT0002187			
hsa-miR-302a*	0.1	0.1	Not-Detect	Not-Detect	1	AGCAAGTACATCCACGTTT	chr4	MIMAT0000683	113569366	113569349	+
hsa-miR-551b*	0.1	0.1	Not-Detect	Not-Detect	1	GGTCTCACCCACG	chr3	MIMAT0004794	168269673	168269684	-
hsa-miR-329	0.1	0.1	Not-Detect	Not-Detect	1	AAAGAGGTTAACCAGGTGTG	chr14	MIMAT0001629	101493173	101493192	-
hsa-miR-195*	0.1	0.1	Not-Detect	Not-Detect	1	GGAGCAGCACAGCCA	chr17	MIMAT0004615	6921007	6920994	+
hsa-miR-3918	0.1	0.1	Not-Detect	Not-Detect	1	AGTCTCCATCTGCGG	chr6	MIMAT0018192	159185731	159185718	+
hsa-miR-10b	0.1	0.1	Not-Detect	Not-Detect	1	CACAAATTCGGTTCTACAGGG	chr2	MIMAT0000254	177015060	177015079	-
hsa-miR-498	0.1	0.1	Not-Detect	Not-Detect	1	GAAAAACGCCCCCTGGC	chr19	MIMAT0002824	54177491	54177506	-
hsa-miR-3128	0.1	0.1	Not-Detect	Not-Detect	1	ATGAGAGTTTTTTACTTGCC	chr2	MIMAT0014991	178120699	178120681	+
hsa-miR-1468	0.1	0.1	Not-Detect	Not-Detect	1	CAGCGAAACAGGCA	chrX	MIMAT0006789	63005914	63005902	+
hsa-miR-588	0.1	0.1	Not-Detect	Not-Detect	1	GTTCTAACCCATTGTGGC	chr6	MIMAT0003255	126805796	126805812	-
hsa-miR-3612	0.1	0.1	Not-Detect	Not-Detect	1	TCCATTTCTCAAGATGCC	chr12	MIMAT0017989	128778655	128778671	-
hsa-miR-92b*	0.1	0.1	Not-Detect	Not-Detect	1	CACTGCACCGCGTC	chr1	MIMAT0004792	155164996	155165008	-
hsa-miR-1229	0.1	0.1	Not-Detect	Not-Detect	1	CTGTGGGAGGGC	chr5	MIMAT0005584	179225346	179225336	+
hsa-miR-515-3p	0.1	0.1	Not-Detect	Not-Detect	1	AACGCTCCAAAAGAAGGCACT	chr19	MIMAT0002827	54182308	54182328	-
hsa-miR-3157	0.1	0.1	Not-Detect	Not-Detect	1	AGACTGCACTAGCCTG	chr10	MIMAT0015031	97824102	97824088	+
hsa-miR-616*	0.1	0.1	Not-Detect	Not-Detect	1	AAGTCACTGAAGGGTTT	chr12	MIMAT0003284	56199249	56199233	+
hsa-miR-3654	0.1	0.1	Not-Detect	Not-Detect	1	TTCCTCAGCTTGTCCA	chr7	MIMAT0018074	132719675	132719661	+
ebv-miR-BART11-5p	0.1	0.1	Not-Detect	Not-Detect	1	CAACTAGCGCACCAAACT		MIMAT0003421			
hsv1-miR-H17	0.1	0.1	Not-Detect	Not-Detect	1	CCGCCTCGCGC		MIMAT0014695			
hsa-miR-219-5p	0.1	0.1	Not-Detect	Not-Detect	1	AGAATTGCGTTTGGACAATCA	chr6	MIMAT0000276	33175632	33175652	-
hsa-miR-4277	0.1	0.1	Not-Detect	Not-Detect	1	GTGTACTGTGCTCAGAA	chr5	MIMAT0016908	1708930	1708915	+
hsa-miR-126*	0.1	0.1	Not-Detect	Not-Detect	1	CGCGTACCAAAAGTAATAATG	chr9	MIMAT0000444	139565069	139565088	-

hsa-miR-520h	0.1	0.1	Not-Detect	Not-Detect	1	ACTCTAAAGGGAAGCACTTTG	chr19	MIMAT0002867	54245822	54245841	-
hsa-miR-3187	0.1	0.1	Not-Detect	Not-Detect	1	CCGCGCAGCCC	chr19	MIMAT0015069	813637	813646	-
hsa-miR-643	0.1	0.1	Not-Detect	Not-Detect	1	CTACCTGAGCTAGCATAC	chr19	MIMAT0003313	52785115	52785131	-
ebv-miR-BART8*	0.1	0.1	Not-Detect	Not-Detect	1	TCTACGACCCCATAG		MIMAT0003418			
hsv2-miR-H6	0.1	0.1	Not-Detect	Not-Detect	1	GCATCCCCTCGCC		MIMAT0015651			
hsa-miR-4308	0.1	0.1	Not-Detect	Not-Detect	1	AAGAAGAAACTCCAGGG	chr14	MIMAT0016861	55344901	55344886	+
hsa-miR-1283	0.1	0.1	Not-Detect	Not-Detect	1	AGAAAGCGCTTTCCTTT	chr19	MIMAT0005799	54191753	54191769	-
hsa-miR-548g	0.1	0.1	Not-Detect	Not-Detect	1	GTACAAAAGTAATTACAGTTTT	chr4	MIMAT0005912	148265855	148265835	+
hsa-miR-187*	0.1	0.1	Not-Detect	Not-Detect	1	GCCCGGGTCCTGT	chr18	MIMAT0004561	33484836	33484825	+
hsa-miR-374b*	0.1	0.1	Not-Detect	Not-Detect	1	AATGATAATACAACCTGCTAAG	chrX	MIMAT0004956	73438443	73438423	+
hsa-let-7a*	0.1	0.1	Not-Detect	Not-Detect	1	GAAAGACAGTAGATTGTATAG	chr9	MIMAT0004481	96938295	96938315	-
hsa-miR-451	169956.7	169956.6967	Detected	Detected	1	AACTCAGTAATGGTAACGGTTT	chr17	MIMAT0001631	27188424	27188404	+
hsa-miR-302e	0.1	0.1	Not-Detect	Not-Detect	1	AAGCATGGAAGCACTTA	chr11	MIMAT0005931	7256003	7256018	-
hsa-miR-132*	0.1	0.1	Not-Detect	Not-Detect	1	AGTAACAATCGAAAGCCACG	chr17	MIMAT0004594	1953245	1953227	+
hsa-miR-134	0.1	0.1	Not-Detect	Not-Detect	1	CCCCTCTGGTCAA	chr14	MIMAT0000447	101521041	101521052	-
hsa-miR-566	0.1	0.1	Not-Detect	Not-Detect	1	GTTGGGATCACAGGCGCCC	chr3	MIMAT0003230	50210775	50210792	-
hsa-miR-33a*	0.1	0.1	Not-Detect	Not-Detect	1	GTGATGCACTGTGGAA	chr22	MIMAT0004506	42297000	42297014	-
hsa-miR-876-3p	0.1	0.1	Not-Detect	Not-Detect	1	TGAATTACTTTGTAAACCACCA	chr9	MIMAT0004925	28863694	28863674	+
hsa-miR-1973	0.1	0.1	Not-Detect	Not-Detect	1	TATGCTACCTTTGCACG	chr4	MIMAT0009448	117220909	117220924	-
hsa-miR-3925	0.1	0.1	Not-Detect	Not-Detect	1	AGGCTCCACTTTCAGT	chr6	MIMAT0018200	36590245	36590231	+
hsa-miR-1183	0.1	0.1	Not-Detect	Not-Detect	1	TGCCCACTCTCACCA	chr7	MIMAT0005828	21510736	21510749	-
hsa-miR-3134	0.1	0.1	Not-Detect	Not-Detect	1	AATATGTAGTCTTTTATCCATCA	chr3	MIMAT0015000	15738871	15738850	+
hsa-miR-1470	0.1	0.1	Not-Detect	Not-Detect	1	CGGGGTGCACGG	chr19	MIMAT0007348	15560369	15560379	-
hsa-miR-593	0.1	0.1	Not-Detect	Not-Detect	1	AGAAACCCCAGCAGAGA	chr7	MIMAT0004802	127721994	127722009	-
hsa-miR-3616-5p	0.1	0.1	Not-Detect	Not-Detect	1	ACATATCATGAGTGCACTT	chr20	MIMAT0017995	45795631	45795648	-
hsa-miR-937	0.1	0.1	Not-Detect	Not-Detect	1	GGCAGAGAGTCAGAGC	chr8	MIMAT0004980	144895198	144895184	+
hsa-miR-4252	0.1	0.1	Not-Detect	Not-Detect	1	TGGTGCTGACTCAGTG	chr1	MIMAT0016886	6489946	6489932	+
hsa-miR-124	0.1	0.1	Not-Detect	Not-Detect	1	GGCATTCACCGCGTGC	chr8	MIMAT0000422	65291771	65291786	-
hsa-miR-517b	0.1	0.1	Not-Detect	Not-Detect	1	AACACTCTAAAGGGATGCACG	chr19	MIMAT0002857	54224374	54224394	-
hsa-miR-3164	0.1	0.1	Not-Detect	Not-Detect	1	CGCCATTTCCCTTAAA	chr11	MIMAT0015038	68850660	68850674	-
hsa-miR-3661	0.1	0.1	Not-Detect	Not-Detect	1	CAGCTGTCCGAGTCC	chr5	MIMAT0018082	133561476	133561489	-
hsv1-miR-H4*	0.1	0.1	Not-Detect	Not-Detect	1	ACTAGCGAGTTAGACAGG		MIMAT0008402			
hsa-miR-223	15182.923	15182.92333	Detected	Detected	1	TGGGGTATTTGACAAACTGAC	chrX	MIMAT0000280	65238781	65238800	-
hsa-miR-1265	0.1	0.1	Not-Detect	Not-Detect	1	AACAACACTTGACCACA	chr10	MIMAT0005918	14478594	14478609	-
hsa-miR-526b	0.1	0.1	Not-Detect	Not-Detect	1	ACAGAAAGTGCTTCCCTC	chr19	MIMAT0002835	54197666	54197682	-
hsa-miR-3194	0.1	0.1	Not-Detect	Not-Detect	1	CAGCCCTCCTGGTG	chr20	MIMAT0015078	50069471	50069459	+
hsa-miR-650	0.1	0.1	Not-Detect	Not-Detect	1	GTCCTGAGAGCGCTGC	chr22	MIMAT0003320	23165291	23165305	-
hsa-miR-3684	0.1	0.1	Not-Detect	Not-Detect	1	AAGGACGTGTACTAGGT	chr4	MIMAT0018112	99918590	99918605	-
hbv-miR-B20	0.1	0.1	Not-Detect	Not-Detect	1	ACGCGCTCGCCA		MIMAT0012172			
kshv-miR-K12-1	0.1	0.1	Not-Detect	Not-Detect	1	GCTTACACCCAGTTTCC		MIMAT0002182			
hsa-miR-2909	0.1	0.1	Not-Detect	Not-Detect	1	CCAAGAGATGTTGGCC	chr17	MIMAT0013863	35391051	35391065	-
hsa-miR-4313	0.1	0.1	Not-Detect	Not-Detect	1	GGGTTTGGGGCCA	chr15	MIMAT0016865	76054585	76054574	+
hsa-miR-129*	0.1	0.1	Not-Detect	Not-Detect	1	ATACTTTTTGGGGTAAGGG	chr7	MIMAT0004548	127847977	127847994	-
hsa-miR-548n	0.1	0.1	Not-Detect	Not-Detect	1	ACAAAATCCACAATTACTTTT	chr7	MIMAT0005916	34980402	34980383	+
hsa-miR-675	0.1	0.1	Not-Detect	Not-Detect	1	CACTGTGGGCCCTC	chr11	MIMAT0004284	2018020	2018008	+
hsa-miR-190	0.1	0.1	Not-Detect	Not-Detect	1	ACCTAATATATCAAACATATCA	chr15	MIMAT0000458	63116171	63116191	-
hsa-miR-377	0.1	0.1	Not-Detect	Not-Detect	1	ACAAAAGTTGCCTTTGTGTG	chr14	MIMAT0000730	101528434	101528452	-
hsa-let-7e	0.1	0.1	Not-Detect	Not-Detect	1	AACTATACAACCTCCTACC	chr19	MIMAT0000066	52196050	52196067	-
hsa-miR-466	0.1	0.1	Not-Detect	Not-Detect	1	ATGTGTGTTGCGTGTATG	chr3	MIMAT0015002	31203269	31203253	+
hsa-miR-486-5p	82079.27	82079.27	Detected	Detected	1	CTCGGGGCAGCTCA	chr8	MIMAT0002177	41637127	41637140	-
hsa-miR-137	0.1	0.1	Not-Detect	Not-Detect	1	CTACGCGTATTCTTAAGCAA	chr1	MIMAT0000429	98284294	98284275	+
hsa-miR-573	0.1	0.1	Not-Detect	Not-Detect	1	CTGATCAGTTACACATCAC	chr4	MIMAT0003238	24521853	24521836	+
hsa-miR-345	0.1	0.1	Not-Detect	Not-Detect	1	GAGCCCTGGACTAG	chr14	MIMAT0000772	100774222	100774234	-
hsa-miR-888	0.1	0.1	Not-Detect	Not-Detect	1	TGACTGACAGCTTTTTGAG	chrX	MIMAT0004916	145076332	145076315	+
hsa-miR-19a*	0.1	0.1	Not-Detect	Not-Detect	1	TGTAGTGCAACTATGCAAAAC	chr13	MIMAT0004490	92003160	92003179	-
hsa-miR-3936	0.1	0.1	Not-Detect	Not-Detect	1	TGCATCTGCCATACACC	chr5	MIMAT0018351	131701268	131701253	+
hsa-miR-1203	0.1	0.1	Not-Detect	Not-Detect	1	GAGCTGCATCCTGGC	chr17	MIMAT0005866	46233814	46233801	+
hsa-miR-506	0.1	0.1	Not-Detect	Not-Detect	1	TCTACTCAGAAGGGTGC	chrX	MIMAT0002878	146312328	146312313	+
hsa-miR-3141	0.1	0.1	Not-Detect	Not-Detect	1	TCCTCCTCCACCCG	chr5	MIMAT0015010	153975599	153975587	+
hsa-miR-149	0.1	0.1	Not-Detect	Not-Detect	1	GGGAGTGAAGACACGGAG	chr2	MIMAT0000450	241395438	241395454	-
hsa-miR-600	0.1	0.1	Not-Detect	Not-Detect	1	GAGCAAGGCTCTTGTC	chr9	MIMAT0003268	125873907	125873893	+
hsa-miR-3621	0.1	0.1	Not-Detect	Not-Detect	1	CCTGCAGACCCCG	chr9	MIMAT0018002	140063672	140063661	+
hsa-miR-944	0.1	0.1	Not-Detect	Not-Detect	1	CTCATCCGATGTACAATAAT	chr3	MIMAT0004987	189547767	189547785	-
hsa-miR-2114*	0.1	0.1	Not-Detect	Not-Detect	1	AAGTCCCTTGCTTGAGG	chrX	MIMAT0011157	149396292	149396307	-
hsa-miR-4259	0.1	0.1	Not-Detect	Not-Detect	1	TCCTGACCCCTAGACC	chr1	MIMAT0016880	159869859	159869845	+
hsa-miR-1248	0.1	0.1	Not-Detect	Not-Detect	1	TTTAGCACAGTGCTTATACA	chr3	MIMAT0005900	186504472	186504490	-
hsa-miR-518d-3p	0.1	0.1	Not-Detect	Not-Detect	1	GCTCCAAAGGGAAGCGCT	chr19	MIMAT0002864	54238186	54238203	-
hsa-miR-3171	0.1	0.1	Not-Detect	Not-Detect	1	GATATATACAGATTCCATACATC	chr14	MIMAT0015046	28102443	28102422	+
hsa-miR-628-3p	0.1	0.1	Not-Detect	Not-Detect	1	TCGACTGCCACTCTT	chr15	MIMAT0003297	55665218	55665205	+
hsa-miR-3667-3p	0.1	0.1	Not-Detect	Not-Detect	1	AAAGACCCATGGAGAGG	chr22	MIMAT0018090	49937106	49937091	+
ebv-miR-BART2-3p	0.1	0.1	Not-Detect	Not-Detect	1	TTTATTTTCTCCAAATCGCTC		MIMAT0004744			
hsv1-miR-H8	0.1	0.1	Not-Detect	Not-Detect	1	GAACCCCCTGACCC		MIMAT0012597			
hsa-miR-2278	0.1	0.1	Not-Detect	Not-Detect	1	CCAGGCAACACACAC	chr9	MIMAT0011778	97572267	97572280	-
hsa-miR-4290	0.1	0.1	Not-Detect	Not-Detect	1	GAGGGAAGAAAGGAGG	chr9	MIMAT0016921	92785807	92785793	+
hsa-miR-1270	0.1	0.1	Not-Detect	Not-Detect	1	ACACAGCTCTTCCATATC	chr19	MIMAT0005924	20510115	20510098	+
hsa-miR-542-3p	0.1	0.1	Not-Detect	Not-Detect	1	TTTCAGTTATCAATCTGTCACA	chrX	MIMAT0003389	133675444	133675424	+
hsa-miR-181a*	0.1	0.1	Not-Detect	Not-Detect	1	GGTACAATCAACGGTCGA	chr1	MIMAT0000270	198828257	198828241	+
hsa-miR-3689b*	0.1	0.1	Not-Detect	Not-Detect	1	ACCACAATATCACACCTC	chr9	MIMAT0018181	137742077	137742061	+
hcmv-miR-UL36	0.1	0.1	Not-Detect	Not-Detect	1	TCTTTCCAGGTGTCTTC		MIMAT0001576			
kshv-miR-K12-12*	0.1	0.1	Not-Detect	Not-Detect	1	CGGAGAGGAATGGTG		MIMAT0003712			
hsa-miR-1294	0.1	0.1	Not-Detect	Not-Detect	1	AGACAACAATGCCAACC	chr5	MIMAT0005884	153726719	153726734	-
hsa-miR-548v	0.1	0.1	Not-Detect	Not-Detect	1	TGGTGCAAAAGTAACTGT	chr8	MIMAT0015020	17539156	17539140	+
hsa-miR-708	0.1	0.1	Not-Detect	Not-Detect	1	CCCAGCTAGATTGTAA	chr11	MIMAT0004926	79113098	79113084	+
hsa-miR-1910	0.1	0.1	Not-Detect	Not-Detect	1	AGGCGGCAGGCAC	chr16	MIMAT0007884	85775258	85775247	+
hsa-miR-379*	0.1	0.1	Not-Detect	Not-Detect	1	AGTTAGTGGACCATGTTA	chr14	MIMAT0004690	101488451	101488467	-
hsa-miR-758	0.1	0.1	Not-Detect	Not-Detect	1	GGTTAGTGGACCAGGTCAC	chr14	MIMAT0003879	101492412	101492429	-
hsa-miR-3907	0.1	0.1	Not-Detect	Not-Detect	1	TGTGAGCCAGCCTGG	chr7	MIMAT0018179	151130688	151130675	+
hsa-miR-490-5p	0.1	0.1	Not-Detect	Not-Detect	1	ACCCACCTGGAGATC	chr7	MIMAT0004764	136587958	136587971	-
hsa-miR-3118	0.1	0.1	Not-Detect	Not-Detect	1	AGAATTTTCATAATGCAGTCA	chr1	MIMAT0014980	142667334	142667354	-
hsa-miR-578	0.1	0.1	Not-Detect	Not-Detect	1	ACAATCCTAGAGCACAAG	chr4	MIMAT0003243	166307458	166307474	-
hsa-miR-34c-5p	0.1	0.1	Not-Detect	Not-Detect	1	GCAATCAGCTAACTACACTG	chr11	MIMAT0000686	110889389	110889408	-
hsa-miR-892b	0.1	0.1	Not-Detect	Not-Detect	1	TCTACCCAGAAAGGAGCCA	chrX	MIMAT0004918	145078782	145078765	+
hsa-miR-200b*	0.1	0.1	Not-Detect	Not-Detect	1	TCCAATGCTGCCCAG	chr1	MIMAT0004571	1102512	1102525	-
hsa-miR-3943	0.1	0.1	Not-Detect	Not-Detect	1	CGCCAAGTGAAGCC	chr7	MIMAT0018359	43190526	43190538	-
hsa-miR-122	0.1	0.1	Not-Detect	Not-Detect	1	CAAACACCATTGTCACACT	chr18	MIMAT0000421	56118324	56118341	-
hsa-miR-510	0.1	0.1	Not-Detect	Not-Detect	1	GTGATTGCCACTCTCC	chrX	MIMAT0002882	146353883	146353869	+
hsa-miR-3147	0.1	0.1	Not-Detect	Not-Detect	1	TCACACCCTCCTCAC	chr7	MIMAT0015019	57472746	57472759	-
hsa-miR-153	0.1	0.1	Not-Detect	Not-Detect	1	GATCACTTTTGTGACTATGC	chr2	MIMAT0000439	220158907	220158888	+
hsa-miR-607	0.1	0.1	Not-Detect	Not-Detect	1	GTTATAGATCTGGATTTGAAC	chr10	MIMAT0003275	98588506	98588487	+
hsa-miR-3646	0.1	0.1	Not-Detect	Not-Detect	1	TGGGCTGGGCTCA	chr20	MIMAT0018065	43036827	43036838	-
hsa-miR-99b	0.1	0.1	Not-Detect	Not-Detect	1	CGCAAGGTCGGTTCTA	chr19	MIMAT0000689	52195878	52195892	-
hsa-miR-214	0.1	0.1	Not-Detect	Not-Detect	1	ACTGCCTGTCTGT	chr1	MIMAT0000271	172108029	172108018	+
hsa-miR-4266	0.1	0.1	Not-Detect	Not-Detect	1	GGCCAAGGCCTCC	chr2	MIMAT0016892	109930071	109930060	+
hsa-miR-1255a	0.1	0.1	Not-Detect	Not-Detect	1	AATCTACTTTCTTTGCTCATC	chr4	MIMAT0005906	102251508	102251489	+
hsa-miR-519d	0.1	0.1	Not-Detect	Not-Detect	1	CACTCTAAAGGGAGGCACT	chr19	MIMAT0002853	54216658	54216675	-
hsa-miR-3179	0.1	0.1	Not-Detect	Not-Detect	1	ACGTTTAAATTTCACCCC	chr16	MIMAT0015056	14995420	14995437	-
hsa-miR-633	0.1	0.1	Not-Detect	Not-Detect	1	TTTATTGTGGTAGATACTATTAG	chr17	MIMAT0003303	61021637	61021658	-
hsa-miR-3671	0.1	0.1	Not-Detect	Not-Detect	1	TGCAGACTAGTCCTTATTTG	chr1	MIMAT0018094	65523518	65523500	+
ebv-miR-BART3	0.1	0.1	Not-Detect	Not-Detect	1	ACACCTGGTGACTAGTG		MIMAT0003411			
hsv2-miR-H20	0.1	0.1	Not-Detect	Not-Detect	1	AGAAAGGGGTCCGTAA		MIMAT0014702			

hsa-miR-23c	0.1	0.1	Not-Detect	Not-Detect	1	GGGTAATCACTGGCAA	chrX	MIMAT0018000	20035292	20035278	+
hsa-miR-4297	0.1	0.1	Not-Detect	Not-Detect	1	CACAGACAGGAAGGC	chr10	MIMAT0016846	131641588	131641575	+
hsa-miR-548a-3p	0.1	0.1	Not-Detect	Not-Detect	1	GCAAAAGTAATTGCCAGTT	chr6	MIMAT0003251	18572078	18572096	−
hsa-miR-654-5p	0.1	0.1	Not-Detect	Not-Detect	1	GCACATGTTCTGCGGCCCA	chr14	MIMAT0003330	100576327	100576345	−
hsa-miR-182*	0.1	0.1	Not-Detect	Not-Detect	1	TAGTTGGCAAGTCTAGAACC	chr7	MIMAT0000260	129410309	129410291	+
hsa-miR-370	0.1	0.1	Not-Detect	Not-Detect	1	ACCAGGTTCCACCC	chr14	MIMAT0000722	101377532	101377544	−
hcmv-miR-US25-2-5p	0.1	0.1	Not-Detect	Not-Detect	1	TCATCCACCTGAACAGA		MIMAT0001582			
hsa-miR-433	0.1	0.1	Not-Detect	Not-Detect	1	ACACCGAGGAGCCC	chr14	MIMAT0001627	101348295	101348307	−
kshv-miR-K12-5	0.1	0.1	Not-Detect	Not-Detect	1	ACCGGCAAGTTCCAG		MIMAT0002190			
hsa-miR-300	0.1	0.1	Not-Detect	Not-Detect	1	AGAGAGAGTCTGCCCT	chr14	MIMAT0004903	101507758	101507772	−
hsa-miR-1302	0.1	0.1	Not-Detect	Not-Detect	1	TTTAGCATAAGTATGTCCCA	chr1	MIMAT0005890	30439	30458	−
hsa-miR-193b*	0.1	0.1	Not-Detect	Not-Detect	1	TCATCTCGCCCTC	chr16	MIMAT0004767	14397847	14397858	−
hsa-miR-3914	0.1	0.1	Not-Detect	Not-Detect	1	ACTTCTCATTTTCTGGTTC	chr7	MIMAT0018188	70772723	70772741	−
hsa-miR-106b*	0.1	0.1	Not-Detect	Not-Detect	1	GCAGCAAGTACCCAC	chr7	MIMAT0004672	99691688	99691675	+
hsa-miR-495	0.1	0.1	Not-Detect	Not-Detect	1	AAGAAGTGCACCATGTTTGT	chr14	MIMAT0002817	101500144	101500162	−
hsa-miR-3125	0.1	0.1	Not-Detect	Not-Detect	1	TCTCTCCACAGCTTCC	chr2	MIMAT0014988	12877507	12877521	−
hsa-miR-92a	33642.35	33642.35	Detected	Detected	1	ACAGGCCGGGACAAGT	chr13	MIMAT0000092	92003621	92003636	−
hsa-miR-205	0.1	0.1	Not-Detect	Not-Detect	1	CAGACTCCGGTGGAAT	chr1	MIMAT0000266	209605518	209605532	−
hsa-miR-411*	0.1	0.1	Not-Detect	Not-Detect	1	GGTTAGTGGACCGTGT	chr14	MIMAT0004813	101489719	101489733	−
hsa-miR-1226*	0.1	0.1	Not-Detect	Not-Detect	1	CCCCATCCAGG	chr3	MIMAT0005576	47891061	47891070	−
hsa-miR-513c	0.1	0.1	Not-Detect	Not-Detect	1	ATAAACGACACCTCCTTGA	chrX	MIMAT0005789	146271256	146271239	+
hsa-miR-3153	0.1	0.1	Not-Detect	Not-Detect	1	AAATGTCCCTACTCGCT	chr9	MIMAT0015026	91927196	91927211	−
hsa-miR-155*	0.1	0.1	Not-Detect	Not-Detect	1	TGTTAATGCTAATATGTAGGAG	chr21	MIMAT0004658	26946335	26946355	−
hsa-miR-614	0.1	0.1	Not-Detect	Not-Detect	1	CCACCTGGCAAGAACAG	chr12	MIMAT0003282	13068823	13068838	−
hsa-miR-3650	0.1	0.1	Not-Detect	Not-Detect	1	GGACTCTACAGACACAC	chr5	MIMAT0018070	38557625	38557610	+
ebv-miR-BART1-5p	0.1	0.1	Not-Detect	Not-Detect	1	CACAGCACGTCACTTC		MIMAT0000999			
hsv1-miR-H14-3p	0.1	0.1	Not-Detect	Not-Detect	1	GTCGCACGCGCC		MIMAT0014692			
hsa-miR-218-1*	0.1	0.1	Not-Detect	Not-Detect	1	CCATGGTGCTTGACG	chr4	MIMAT0004565	20529973	20529986	−
hsa-miR-4273	0.1	0.1	Not-Detect	Not-Detect	1	CTGTCCATCAGAGAACA	chr3	MIMAT0016903	75787443	75787458	−
hsa-miR-3184	0.1	0.1	Not-Detect	Not-Detect	1	AAAAGCTCGGTCTGAGG	chr17	MIMAT0015064	28444136	28444121	+
hsa-miR-640	0.1	0.1	Not-Detect	Not-Detect	1	AGAGGCAGGTTCCTGGA	chr19	MIMAT0003310	19545937	19545952	−
hsa-miR-3677	0.1	0.1	Not-Detect	Not-Detect	1	GGCCGTGGCCAG	chr16	MIMAT0018101	2320763	2320773	−
ebv-miR-BART6-5p	0.1	0.1	Not-Detect	Not-Detect	1	CCTATGGATTGGACCAA		MIMAT0003414			
hsv2-miR-H3	0.1	0.1	Not-Detect	Not-Detect	1	CTCCCAACCGCAGA		MIMAT0010202			
hsa-miR-26a-1*	0.1	0.1	Not-Detect	Not-Detect	1	CGTGCAAGTAACCAAGAATAG	chr3	MIMAT0004499	38010945	38010964	−
hsa-miR-4304	0.1	0.1	Not-Detect	Not-Detect	1	TGCCCTGGACATGC	chr12	MIMAT0016854	123495240	123495228	+
hsa-miR-548d-3p	0.1	0.1	Not-Detect	Not-Detect	1	GCAAAAGAAACTGTGGTT	chr8	MIMAT0003323	124360355	124360338	+
hsa-miR-661	0.1	0.1	Not-Detect	Not-Detect	1	ACGCGCAGGCCAGAGA	chr8	MIMAT0003324	145019432	145019418	+
hsa-miR-185*	0.1	0.1	Not-Detect	Not-Detect	1	GACCAGAGGAAAGCCAG	chr22	MIMAT0004611	20020717	20020732	−
hsa-miR-373*	0.1	0.1	Not-Detect	Not-Detect	1	GGAAAGCGCCCCC	chr19	MIMAT0000725	54291974	54291985	−
hiv1-miR-N367	0.1	0.1	Not-Detect	Not-Detect	1	TTGAAGCACCATCCAAAGG		MIMAT0004478			
hsa-miR-449c*	0.1	0.1	Not-Detect	Not-Detect	1	ACAGAGAGGAGTGCAAC	chr5	MIMAT0013771	54468168	54468153	+
kshv-miR-K12-8*	0.1	0.1	Not-Detect	Not-Detect	1	AGCGGGGCGTTAGT		MIMAT0015216			
hsa-miR-302c	0.1	0.1	Not-Detect	Not-Detect	1	CCACTGAAACATGGAAGCAC	chr4	MIMAT0000717	113569583	113569565	+
hsa-miR-130a*	0.1	0.1	Not-Detect	Not-Detect	1	GCAGACAGTAGCACAATG	chr11	MIMAT0004593	57408696	57408712	−
hsa-miR-554	0.1	0.1	Not-Detect	Not-Detect	1	ACTGGCTGAGTCAGGA	chr1	MIMAT0003217	151518293	151518307	−
hsa-miR-1323	0.1	0.1	Not-Detect	Not-Detect	1	AGAAAATGCCCCTCAGT	chr19	MIMAT0005795	54175238	54175253	−
hsa-miR-561	0.1	0.1	Not-Detect	Not-Detect	1	ACTTCAAGGATCTTAAACTT	chr2	MIMAT0003225	189162282	189162300	−
hsa-miR-338-5p	0.1	0.1	Not-Detect	Not-Detect	1	CACTCAGCACCAGGA	chr17	MIMAT0004701	79099709	79099696	+
hsa-miR-873	0.1	0.1	Not-Detect	Not-Detect	1	AGGAGACTCACAAGTTCCTG	chr9	MIMAT0004953	28888907	28888889	+
hsa-miR-3921	0.1	0.1	Not-Detect	Not-Detect	1	ACAAGGCATATGGTACTC	chr3	MIMAT0018196	99683231	99683215	+
hsa-miR-1179	0.1	0.1	Not-Detect	Not-Detect	1	CCAACCAATGAAAGAATG	chr15	MIMAT0005824	89151356	89151372	−
hsa-miR-3130-5p	0.1	0.1	Not-Detect	Not-Detect	1	GGCTGCACCGGAG	chr2	MIMAT0014995	207647978	207647990	−
hsa-miR-146a*	0.1	0.1	Not-Detect	Not-Detect	1	CTGAAGAACTGAATTTCAGAGG	chr5	MIMAT0004608	159912416	159912436	−
hsa-miR-590-3p	0.1	0.1	Not-Detect	Not-Detect	1	ACTAGCTTATACATAAAATTA	chr7	MIMAT0004801	73605584	73605603	−
hsa-miR-3614-3p	0.1	0.1	Not-Detect	Not-Detect	1	AAAACACCAAGATCTGAAG	chr17	MIMAT0017993	54968705	54968688	+
hsa-miR-424*	0.1	0.1	Not-Detect	Not-Detect	1	ATAGCAGCGCCTCA	chrX	MIMAT0004749	133680711	133680699	+
hsa-miR-516a-5p	0.1	0.1	Not-Detect	Not-Detect	1	GAAAGTGCTTCTTTCCTCG	chr19	MIMAT0004770	54260014	54260032	−
hsa-miR-3160	0.1	0.1	Not-Detect	Not-Detect	1	TGGGCTTTCTAGTCTCA	chr11	MIMAT0015034	46473413	46473429	−
hsa-miR-619	0.1	0.1	Not-Detect	Not-Detect	1	ACTGGGCACAAACATG	chr12	MIMAT0003288	109230767	109230753	+
hsa-miR-3657	0.1	0.1	Not-Detect	Not-Detect	1	AATCACCAATAATGGGACA	chr12	MIMAT0018077	112475491	112475474	+
ebv-miR-BART13*	0.1	0.1	Not-Detect	Not-Detect	1	CTGTACGAGCCACGA		MIMAT0004818			
hsv1-miR-H2*	0.1	0.1	Not-Detect	Not-Detect	1	AGTCTGTGCCGGGC		MIMAT0008398			
hsa-miR-4280	0.1	0.1	Not-Detect	Not-Detect	1	GCTCTGCTCAGAACTAC	chr5	MIMAT0016911	86410726	86410711	+
hsa-miR-1261	0.1	0.1	Not-Detect	Not-Detect	1	AAGCCAAAGCCTTATCC	chr11	MIMAT0005913	90602311	90602296	+
hsa-miR-523	0.1	0.1	Not-Detect	Not-Detect	1	ACCCTCTATAGGGAAGCG	chr19	MIMAT0002840	54201697	54201713	−
hsa-miR-3190	0.1	0.1	Not-Detect	Not-Detect	1	TCTCTGGCCGTCTACC	chr19	MIMAT0015073	47730252	47730267	−
hsa-miR-646	0.1	0.1	Not-Detect	Not-Detect	1	GCCTCAGAGGCAGCTGCT	chr20	MIMAT0003316	58883594	58883610	−
hsa-miR-3681	0.1	0.1	Not-Detect	Not-Detect	1	GCACAGAGTGCATCAT	chr2	MIMAT0018108	12339271	12339285	−
ebv-miR-BHRF1-1	0.1	0.1	Not-Detect	Not-Detect	1	AACTCCGGGGCTGA		MIMAT0000995			
hsv2-miR-H7-5p	0.1	0.1	Not-Detect	Not-Detect	1	CCTTTGGTTACGGACC		MIMAT0014351			
hsa-miR-27b*	0.1	0.1	Not-Detect	Not-Detect	1	GTTCACCAATCAGCTAA	chr9	MIMAT0004588	97847751	97847766	−
hsa-miR-431*	0.1	0.1	Not-Detect	Not-Detect	1	AGAAGCCCTGCAAGACG	chr14	MIMAT0004757	101347412	101347427	−
hsa-miR-1286	0.1	0.1	Not-Detect	Not-Detect	1	AGGGCTCATCTTGGTC	chr22	MIMAT0005877	20236723	20236709	+
hsa-miR-548j	0.1	0.1	Not-Detect	Not-Detect	1	ACCAAAGACCGCAATTA	chr22	MIMAT0005875	26951227	26951212	+
hsa-miR-668	0.1	0.1	Not-Detect	Not-Detect	1	GTAGTGGGCCGAGCCGA	chr14	MIMAT0003881	101521644	101521659	−
hsa-miR-376a	0.1	0.1	Not-Detect	Not-Detect	1	ACGTGGATTTTCCTCTATG	chr14	MIMAT0000729	101506457	101506475	−
hsa-miR-3065-5p	0.1	0.1	Not-Detect	Not-Detect	1	TCCAGCATCAGTGATTTT	chr17	MIMAT0015066	79099692	79099708	−
hsa-miR-135b	0.1	0.1	Not-Detect	Not-Detect	1	TCACATAGGAATGAAAAGCCATA	chr1	MIMAT0000758	205417467	205417446	+
hsa-miR-569	0.1	0.1	Not-Detect	Not-Detect	1	ACTTTCCAGGATTCATTAAC	chr3	MIMAT0003234	170824533	170824515	+
hsa-miR-340	0.1	0.1	Not-Detect	Not-Detect	1	AATCAGTCTCATTGCTTTA	chr5	MIMAT0004692	179442339	179442322	+
hsa-miR-877*	0.1	0.1	Not-Detect	Not-Detect	1	CTGGGAGGAGGGAG	chr6	MIMAT0004950	30552182	30552194	−
hsa-miR-199a-3p	0.1	0.1	Not-Detect	Not-Detect	1	TAACCAATGTGCAGACTACT	chr1	MIMAT0000232	172113765	172113746	+
hsa-miR-3928	0.1	0.1	Not-Detect	Not-Detect	1	GCCGAAGCTCCAAG	chr22	MIMAT0018205	31556105	31556093	+
hsa-miR-1193	0.1	0.1	Not-Detect	Not-Detect	1	GCACGTCACCGGT	chr14	MIMAT0015049	101496409	101496420	−
hsa-miR-503	0.1	0.1	Not-Detect	Not-Detect	1	CTGCAGAACTGTTCCCGC	chrX	MIMAT0002874	133680385	133680369	+
hsa-miR-3137	0.1	0.1	Not-Detect	Not-Detect	1	ACCCCATTGCTCCCA	chr3	MIMAT0015005	194855264	194855251	+
hsa-miR-596	0.1	0.1	Not-Detect	Not-Detect	1	CCCGAGGAGCCGG	chr8	MIMAT0003264	1765421	1765432	−
hsa-miR-3619	0.1	0.1	Not-Detect	Not-Detect	1	GCTGCACCAGCCT	chr22	MIMAT0017999	46486949	46486960	−
hsa-miR-211	0.1	0.1	Not-Detect	Not-Detect	1	AGGCGAAGGATGACAAAGGGA	chr15	MIMAT0000268	31357281	31357262	+
hsa-miR-4255	0.1	0.1	Not-Detect	Not-Detect	1	TCCATCTCTGAACACTG	chr1	MIMAT0016885	37627175	37627190	−
hsa-miR-1244	0.1	0.1	Not-Detect	Not-Detect	1	AACCATCTCATACAAACCA	chr2	MIMAT0005896	232578085	232578103	−
hsa-miR-518a-5p	0.1	0.1	Not-Detect	Not-Detect	1	GAAAGGGCTTCCCTT	chr19	MIMAT0005457	54223370	54223384	−
hsa-miR-3167	0.1	0.1	Not-Detect	Not-Detect	1	ACACCAGTATTTCTGAAATC	chr11	MIMAT0015042	126858428	126858410	+
ebv-miR-BART18-3p	0.1	0.1	Not-Detect	Not-Detect	1	GACGAAGCCCAAACTTCCG		MIMAT0004835			
hsa-miR-224*	0.1	0.1	Not-Detect	Not-Detect	1	TGTAGTCACTAGGGCAC	chrX	MIMAT0009198	151127124	151127109	+
hsa-miR-4287	0.1	0.1	Not-Detect	Not-Detect	1	AAAGTGCCCTCAAGGG	chr8	MIMAT0016917	27743584	27743570	+
hsa-miR-3197	0.1	0.1	Not-Detect	Not-Detect	1	CGCCTTTCCGAGC	chr21	MIMAT0015082	42539502	42539513	−
hsa-miR-653	0.1	0.1	Not-Detect	Not-Detect	1	CAGTAGAGATTGTTTCAACA	chr7	MIMAT0003328	93112104	93112086	+
hsa-miR-3687	0.1	0.1	Not-Detect	Not-Detect	1	ACGTCGCACGAACG	chr21	MIMAT0018115	9826248	9826260	−
hcmv-miR-UL112	0.1	0.1	Not-Detect	Not-Detect	1	AGCCTGGATCTCACCG		MIMAT0001577			
hsa-miR-432*	0.1	0.1	Not-Detect	Not-Detect	1	AGACATGGAGGAGCCAT	chr14	MIMAT0002815	101350886	101350901	−
kshv-miR-K12-10b	0.1	0.1	Not-Detect	Not-Detect	1	GCCACTCGGGGGG		MIMAT0002180			
hsa-miR-297	0.1	0.1	Not-Detect	Not-Detect	1	CATGCACATGCACACAT	chr4	MIMAT0004450	111781761	111781746	+
hsa-miR-1290	0.1	0.1	Not-Detect	Not-Detect	1	TCCCTGATCCAAAAATCC	chr1	MIMAT0005880	19223635	19223619	+
hsa-miR-548q	0.1	0.1	Not-Detect	Not-Detect	1	CCGCCATTACTTTTGC	chr10	MIMAT0011163	12767281	12767267	+
hsa-miR-3202	0.1	0.1	Not-Detect	Not-Detect	1	ATTAAAGCTCTTCTCCCTT	chrX	MIMAT0015089	153246559	153246577	−
hsa-miR-676*	0.1	0.1	Not-Detect	Not-Detect	1	TGCAAGTCCTGAGGTT	chrX	MIMAT0018203	69242720	69242734	−
hsa-miR-1909*	0.1	0.1	Not-Detect	Not-Detect	1	CAGGGCAGGCACC	chr19	MIMAT0007882	1816193	1816182	+
hsa-let-7f-1*	0.1	0.1	Not-Detect	Not-Detect	1	GGGAAGGCAATAGATTGTAT	chr9	MIMAT0004486	96938694	96938712	−

hsa-miR-488	0.1	0.1	Not-Detect	Not-Detect	1	GACCAAGAAATAGCCTTTCA	chr1	MIMAT0004763	176998570	176998552	+
hsa-miR-31*	0.1	0.1	Not-Detect	Not-Detect	1	ATGGCAATATGTTGGCATAG	chr9	MIMAT0004504	21512178	21512160	+
hsa-miR-138-2*	0.1	0.1	Not-Detect	Not-Detect	1	AACCCTGGTGTCGTGA	chr16	MIMAT0004596	56892493	56892507	-
hsa-miR-575	0.1	0.1	Not-Detect	Not-Detect	1	GCTCCTGTCCAACTGGCT	chr4	MIMAT0003240	83674568	83674552	+
hsa-miR-34a*	0.1	0.1	Not-Detect	Not-Detect	1	AGGGCAGTATACTTGCTG	chr1	MIMAT0004557	9211811	9211795	+
hsa-miR-890	0.1	0.1	Not-Detect	Not-Detect	1	CAACTGATGCCTTTCCA	chrX	MIMAT0004912	145075823	145075808	+
hsa-miR-19b-2*	0.1	0.1	Not-Detect	Not-Detect	1	TGAAATGCAAACCTGCAAAACT	chrX	MIMAT0004492	133303740	133303720	+
hsa-miR-3939	0.1	0.1	Not-Detect	Not-Detect	1	GACATCCTGTGGTCTG	chr6	MIMAT0018355	167411377	167411363	+
hsa-miR-1206	0.1	0.1	Not-Detect	Not-Detect	1	GCTTAAACATCTACATGAAC	chr8	MIMAT0005870	129021149	129021167	-
hsa-miR-508-5p	0.1	0.1	Not-Detect	Not-Detect	1	CATGAGTGACGCCCTC	chrX	MIMAT0004778	146318478	146318464	+
hsa-miR-3144-3p	0.1	0.1	Not-Detect	Not-Detect	1	TAAAGAGACCGAACAGG	chr6	MIMAT0015015	120336378	120336393	-
hsa-miR-150*	0.1	0.1	Not-Detect	Not-Detect	1	CTGTCCCCCAGGC	chr19	MIMAT0004610	50004113	50004102	+
hsa-miR-603	0.1	0.1	Not-Detect	Not-Detect	1	GCAAAAGTAATTGCAGTGT	chr10	MIMAT0003271	24564678	24564695	-
hsa-miR-3622b-3p	0.1	0.1	Not-Detect	Not-Detect	1	CAGGCACGGGAGC	chr8	MIMAT0018006	27559267	27559256	+
hsa-miR-96*	0.1	0.1	Not-Detect	Not-Detect	1	CATATTGGCACTGCACA	chr7	MIMAT0004510	129414604	129414589	+
hsa-miR-2116	0.1	0.1	Not-Detect	Not-Detect	1	AGACCTCCTATGCTAAGAA	chr15	MIMAT0011160	59463415	59463398	+
hsa-miR-4262	0.1	0.1	Not-Detect	Not-Detect	1	CAGGTAGTCTGAATGTC	chr2	MIMAT0016894	11977102	11977087	+
hsa-miR-1251	0.1	0.1	Not-Detect	Not-Detect	1	AGCGCCTTTGGCAG	chr12	MIMAT0005903	97885699	97885711	-
hsa-miR-518f	0.1	0.1	Not-Detect	Not-Detect	1	CCTCTAAAGAGAAGCGCTTT	chr19	MIMAT0002842	54203323	54203341	-
hsa-miR-3175	0.1	0.1	Not-Detect	Not-Detect	1	ACGTCACTGCGTTCT	chr15	MIMAT0015052	93447646	93447659	-
hsa-miR-3669	0.1	0.1	Not-Detect	Not-Detect	1	TATATTCCGTATACATATTCCG	chr8	MIMAT0018092	130509669	130509649	+
ebv-miR-BART20-5p	0.1	0.1	Not-Detect	Not-Detect	1	GGAATGAAGACATGCCT		MIMAT0003719			
hsv2-miR-H11*	0.1	0.1	Not-Detect	Not-Detect	1	TAGGACAAAGTGCGAAC		MIMAT0014697			
hsa-miR-4293	0.1	0.1	Not-Detect	Not-Detect	1	CTGTTCCTGTCAGGC	chr10	MIMAT0016848	14425266	14425253	+
hsa-miR-1273	0.1	0.1	Not-Detect	Not-Detect	1	AAGAAAGAGTCTTGCTTTG	chr8	MIMAT0005926	101036309	101036292	+
hsa-miR-544	0.1	0.1	Not-Detect	Not-Detect	1	GAACTTGCTAAAAATGCAGAAT	chr14	MIMAT0003164	101515050	101515070	-
hsa-miR-181c	0.1	0.1	Not-Detect	Not-Detect	1	ACTCACCGACAGGTTGAAT	chr19	MIMAT0000258	13985543	13985560	-
hsa-miR-3690	0.1	0.1	Not-Detect	Not-Detect	1	CTTTGTCTACGCTGGG	chrX	MIMAT0018119	1412828	1412842	-
hcmv-miR-UL70-5p	0.1	0.1	Not-Detect	Not-Detect	1	TCTGGACGAGGCCGA		MIMAT0003342			
hsa-miR-4326	0.1	0.1	Not-Detect	Not-Detect	1	GTCTGGGAGACAGAGG	chr20	MIMAT0016888	61918175	61918189	-
hsa-miR-29b-1*	0.1	0.1	Not-Detect	Not-Detect	1	TCTAAACCACCATATGAAACCAG	chr7	MIMAT0004514	130562250	130562229	+
hsa-miR-1297	0.1	0.1	Not-Detect	Not-Detect	1	CACCTGAATTACTTGAA	chr13	MIMAT0005886	54886174	54886159	+
hsa-miR-548y	0.1	0.1	Not-Detect	Not-Detect	1	GGCAAAAACAGTGATTAC	chr14	MIMAT0018354	48230241	48230225	+
hsa-miR-323-5p	0.1	0.1	Not-Detect	Not-Detect	1	GCGAACGCGCCACG	chr14	MIMAT0004696	101492093	101492105	-
hsa-miR-718	0.1	0.1	Not-Detect	Not-Detect	1	CGACGCCCGGC	chrX	MIMAT0012735	153285432	153285423	+
hsa-miR-1912	0.1	0.1	Not-Detect	Not-Detect	1	TTCACACTGCATGCTCT	chrX	MIMAT0007887	113886070	113886085	-
hsa-miR-381	0.1	0.1	Not-Detect	Not-Detect	1	ACAGAGAGCTTGCCCT	chr14	MIMAT0000736	101512312	101512326	-
hsa-miR-761	0.1	0.1	Not-Detect	Not-Detect	1	TGTGTCAGTTTCACCC	chr1	MIMAT0010364	52302043	52302029	+
hsa-miR-192*	0.1	0.1	Not-Detect	Not-Detect	1	CTGTGACCTATGGAATTGG	chr11	MIMAT0004543	64658696	64658679	+
hsa-miR-3910	0.1	0.1	Not-Detect	Not-Detect	1	TGTCTTGGTTTTATGCCT	chr9	MIMAT0018184	94398597	94398614	-
hsa-miR-105	0.1	0.1	Not-Detect	Not-Detect	1	ACCACAGGAGTCTGAGC	chrX	MIMAT0000102	151560725	151560709	+
hsa-miR-492	0.1	0.1	Not-Detect	Not-Detect	1	AAGAATCTTGTCCCGCAGG	chr12	MIMAT0002812	95228208	95228225	-
hsa-miR-3121	0.1	0.1	Not-Detect	Not-Detect	1	TGTCCTTTGCCTACTCT	chr1	MIMAT0014983	180407516	180407501	+
hsa-miR-581	0.1	0.1	Not-Detect	Not-Detect	1	ACTGATCTAGAGAACACAA	chr5	MIMAT0003246	53247369	53247352	+
hsa-miR-3606	0.1	0.1	Not-Detect	Not-Detect	1	AATTAAAATAGCCTTCACTAA	chr2	MIMAT0017983	189860372	189860391	-
hsa-miR-920	0.1	0.1	Not-Detect	Not-Detect	1	TACTGCTTCCACAGCTC	chr12	MIMAT0004970	24365409	24365424	-
hsa-miR-202	0.1	0.1	Not-Detect	Not-Detect	1	TTCCCATGCCCTATA	chr10	MIMAT0002811	135061097	135061084	+
hsa-miR-512-5p	0.1	0.1	Not-Detect	Not-Detect	1	GAAAGTGCCCTCAAGGCT	chr19	MIMAT0002822	54169951	54169968	-
hsa-miR-3150	0.1	0.1	Not-Detect	Not-Detect	1	CCAACCTCGAGGATC	chr8	MIMAT0015023	96085198	96085211	-
hsa-miR-1539	0.1	0.1	Not-Detect	Not-Detect	1	GGGCATCTGGGACG	chr18	MIMAT0007401	47013780	47013792	-
hsa-miR-610	0.1	0.1	Not-Detect	Not-Detect	1	TCCCAGCACACATTTAGC	chr11	MIMAT0003278	28078381	28078397	-
hsa-miR-3648	0.1	0.1	Not-Detect	Not-Detect	1	CCCTCGGCGATCC	chr21	MIMAT0018068	9825868	9825879	-
hsv1-miR-H1*	0.1	0.1	Not-Detect	Not-Detect	1	AGGGTGGAAGGCAGG		MIMAT0015220			
hsa-miR-216a	0.1	0.1	Not-Detect	Not-Detect	1	TCACAGTTGCCAGCTG	chr2	MIMAT0000273	56216124	56216110	+
hsa-miR-4269	0.1	0.1	Not-Detect	Not-Detect	1	GCCAGGGCTGTCTG	chr2	MIMAT0016897	240227175	240227187	-
hsa-miR-1257	0.1	0.1	Not-Detect	Not-Detect	1	GGTCAGAACCCATCAT	chr20	MIMAT0005908	60528643	60528629	+
hsa-miR-520a-3p	0.1	0.1	Not-Detect	Not-Detect	1	ACAGTCCAAAGGGAAGCACT	chr19	MIMAT0002834	54194189	54194208	-
hsa-miR-3180-5p	0.1	0.1	Not-Detect	Not-Detect	1	CGACGTGGGGCG	chr16	MIMAT0015057	15005107	15005118	-
hsa-miR-636	0.1	0.1	Not-Detect	Not-Detect	1	TGCGGGCGGGACG	chr17	MIMAT0003306	74732614	74732603	+
hsa-miR-3674	0.1	0.1	Not-Detect	Not-Detect	1	GGCCAATCTTAGGTTCT	chr8	MIMAT0018097	1749305	1749320	-
ebv-miR-BART4*	0.1	0.1	Not-Detect	Not-Detect	1	ACACCTGGTGCCTAC		MIMAT0009204			
hsv2-miR-H23	0.1	0.1	Not-Detect	Not-Detect	1	GCTGGCAAGCTCCA		MIMAT0014705			
hsa-miR-24-2*	0.1	0.1	Not-Detect	Not-Detect	1	CTGTGTTTCAGCTCA	chr19	MIMAT0004497	13947134	13947121	+
hsa-miR-4300	0.1	0.1	Not-Detect	Not-Detect	1	GAAGTAGTCCAGCTCC	chr11	MIMAT0016853	81601868	81601854	+
hsa-miR-1276	0.1	0.1	Not-Detect	Not-Detect	1	TGTCTCCACAGGGCT	chr15	MIMAT0005930	86313757	86313744	+
hsa-miR-548b-3p	0.1	0.1	Not-Detect	Not-Detect	1	ACAAAAGCAACTGAGGTTC	chr6	MIMAT0003254	119390293	119390275	+
hsa-miR-657	0.1	0.1	Not-Detect	Not-Detect	1	CCTAGAGAGGGTGAGA	chr17	MIMAT0003335	79099158	79099144	+
hsa-miR-3713	0.1	0.1	Not-Detect	Not-Detect	1	ACCATCCCCAAACGG	chr15	MIMAT0018164	76878994	76879007	-
hcmv-miR-US4	0.1	0.1	Not-Detect	Not-Detect	1	ATCCCCCTGCACGTC		MIMAT0003341			
hsa-miR-449a	0.1	0.1	Not-Detect	Not-Detect	1	ACCAGCTAACAATACACTGC	chr5	MIMAT0001541	54466396	54466377	+
kshv-miR-K12-6-5p	0.1	0.1	Not-Detect	Not-Detect	1	CCGATGGATTAGGTGCT		MIMAT0002188			
hsa-miR-302a	0.1	0.1	Not-Detect	Not-Detect	1	TCACCAAAACATGGAAGCAC	chr4	MIMAT0000684	113569404	113569386	+
hsa-miR-551b	0.1	0.1	Not-Detect	Not-Detect	1	CTGAAACCAAGTATGGGTCGC	chr3	MIMAT0003233	168269703	168269722	-
hsa-miR-556-5p	0.1	0.1	Not-Detect	Not-Detect	1	CTCATATTACAATGAGCTCAT	chr1	MIMAT0003220	162312353	162312372	-
hsa-miR-769-3p	0.1	0.1	Not-Detect	Not-Detect	1	AACCAAGACCCCGGAG	chr19	MIMAT0003887	46522266	46522280	-
hsa-miR-10a*	0.1	0.1	Not-Detect	Not-Detect	1	TATTCCCCTAGATACGAA	chr17	MIMAT0004555	46657283	46657267	+
hsa-miR-497*	0.1	0.1	Not-Detect	Not-Detect	1	TCTAACACCACAGTGTGG	chr17	MIMAT0004768	6921314	6921298	+
hsa-miR-3127	0.1	0.1	Not-Detect	Not-Detect	1	CTTCCCATTCCACAAG	chr2	MIMAT0014990	97464033	97464047	-
hsa-miR-145*	0.1	0.1	Not-Detect	Not-Detect	1	AGAACAGTATTTCCAGGAATC	chr5	MIMAT0004601	148810264	148810283	-
hsa-miR-587	0.1	0.1	Not-Detect	Not-Detect	1	GTGACTCATCACCTATGG	chr6	MIMAT0003253	107232019	107232035	-
hsa-miR-3611	0.1	0.1	Not-Detect	Not-Detect	1	TAAGAATTTCTTTCTTCACAA	chr10	MIMAT0017988	35368597	35368578	+
hsa-miR-92b	0.1	0.1	Not-Detect	Not-Detect	1	GGAGGCCGGGACG	chr1	MIMAT0003218	155165038	155165049	-
hsa-miR-2053	0.1	0.1	Not-Detect	Not-Detect	1	GTAAATAGAGGTTTAATTAACAC	chr8	MIMAT0009978	113655783	113655804	-
hsa-miR-422a	0.1	0.1	Not-Detect	Not-Detect	1	GCCTTCTGACCCTA	chr15	MIMAT0001339	64163159	64163147	+
hsa-miR-514b-5p	0.1	0.1	Not-Detect	Not-Detect	1	ATGATTGCCTCCCTCTT	chrX	MIMAT0015087	146331702	146331687	+
hsa-miR-3156	0.1	0.1	Not-Detect	Not-Detect	1	TGTCTCCCACTTCCAG	chr10	MIMAT0015030	45659475	45659490	-
hsa-miR-616	0.1	0.1	Not-Detect	Not-Detect	1	CTGCTCAAACCCTCC	chr12	MIMAT0004805	57913022	57913009	+
ebv-miR-BART11-3p	0.1	0.1	Not-Detect	Not-Detect	1	GGCAGTCAGCCTGG		MIMAT0003422			
hsv1-miR-H16	0.1	0.1	Not-Detect	Not-Detect	1	GCCTTCGATCCCAGC		MIMAT0014694			
hsa-miR-219-2-3p	0.1	0.1	Not-Detect	Not-Detect	1	ACAGATGTCCAGCCAC	chr9	MIMAT0004675	131154979	131154965	+
hsa-miR-4276	0.1	0.1	Not-Detect	Not-Detect	1	GCACATGAGTCACTGA	chr4	MIMAT0016904	175344958	175344972	-
hsa-miR-520g	0.1	0.1	Not-Detect	Not-Detect	1	ACACTCTAAAGGGAAGCACT	chr19	MIMAT0002858	54225479	54225497	-
hsa-miR-3186-5p	0.1	0.1	Not-Detect	Not-Detect	1	AAGCCACGTAGACAGA	chr17	MIMAT0015067	79418166	79418152	+
hsa-miR-642b	0.1	0.1	Not-Detect	Not-Detect	1	GGGTCCCTCTCCAA	chr19	MIMAT0018444	46178257	46178245	+
hsa-miR-3679-3p	0.1	0.1	Not-Detect	Not-Detect	1	GATGAAGATTACTGGGGG	chr2	MIMAT0018105	134884744	134884760	-
ebv-miR-BART8	0.1	0.1	Not-Detect	Not-Detect	1	CTGTACAATCTAGGAAACCG		MIMAT0003417			
hsv2-miR-H5	0.1	0.1	Not-Detect	Not-Detect	1	GGTCAGGTGGCCC		MIMAT0015650			
hsa-miR-4307	0.1	0.1	Not-Detect	Not-Detect	1	GGAAACAGGAAAAAACAT	chr14	MIMAT0016860	27377905	27377921	-
hsa-miR-1282	0.1	0.1	Not-Detect	Not-Detect	1	AAGCAGAAAAAGGCAAAC	chr15	MIMAT0005940	44085884	44085868	+
hsa-miR-548f	0.1	0.1	Not-Detect	Not-Detect	1	AAAAGTAATTACAGTTTTT	chr2	MIMAT0005895	213291063	213291045	+
hsa-miR-663b	0.1	0.1	Not-Detect	Not-Detect	1	CCTCAGGCACGGC	chr2	MIMAT0005867	133014649	133014638	+
hsa-miR-187	0.1	0.1	Not-Detect	Not-Detect	1	CCGGCTGCAACACA	chr18	MIMAT0000262	33484872	33484860	+
hsa-miR-450b-5p	0.1	0.1	Not-Detect	Not-Detect	1	TATTCAGGAACATATTGCAAAA	chrX	MIMAT0004909	133674246	133674226	+
hsa-miR-302d*	0.1	0.1	Not-Detect	Not-Detect	1	GCAAGTGCCTCCAT	chr4	MIMAT0004685	113569186	113569174	+
hsa-miR-133b	6.59395	6.59395	Detected	Detected	1	TAGCTGGTTGAAGGGGACC	chr6	MIMAT0000770	52013789	52013807	-
hsa-miR-564	0.1	0.1	Not-Detect	Not-Detect	1	GCCTGCTGACACCGT	chr3	MIMAT0003228	44903400	44903413	-
hsa-miR-33a	0.1	0.1	Not-Detect	Not-Detect	1	TGCAATGCAACTACAATGCAC	chr22	MIMAT0000091	42296954	42296973	-
hsa-miR-875-5p	0.1	0.1	Not-Detect	Not-Detect	1	CACCTGATAAAACTGAGGTATA	chr8	MIMAT0004922	100549045	100549025	+
hsa-miR-1972	0.1	0.1	Not-Detect	Not-Detect	1	TGAGCCACTGTGCC	chr16	MIMAT0009447	70064304	70064317	-
hsa-miR-3924	0.1	0.1	Not-Detect	Not-Detect	1	AGTAGCAGTCACATATACATAT	chr10	MIMAT0018199	59064309	59064289	+

hsa-miR-1182	0.1	0.1	Not-Detect	Not-Detect	1	GTCACATCCCTCCCA	chr1	MIMAT0005827	231155657	231155644	+
hsa-miR-3133	0.1	0.1	Not-Detect	Not-Detect	1	ATTGGGTTTTAAGAGTTCTT	chr2	MIMAT0014998	242417332	242417350	−
hsa-miR-147	0.1	0.1	Not-Detect	Not-Detect	1	GCAGAAGCATTTCCACAC	chr9	MIMAT0000251	123007322	123007306	+
hsa-miR-592	0.1	0.1	Not-Detect	Not-Detect	1	ACATCATCGCATATTGACAC	chr7	MIMAT0003260	126698178	126698160	+
hsa-miR-3616-3p	0.1	0.1	Not-Detect	Not-Detect	1	GCCTGCATCATGAAATG	chr20	MIMAT0017996	45795675	45795690	−
hsa-miR-936	0.1	0.1	Not-Detect	Not-Detect	1	CTGCGATTCCTCCCT	chr10	MIMAT0004979	105807882	105807869	+
hsa-miR-20b*	0.1	0.1	Not-Detect	Not-Detect	1	CTGGAAGTGCCCAT	chrX	MIMAT0004752	133303903	133303891	+
hsa-miR-4251	0.1	0.1	Not-Detect	Not-Detect	1	TTGGCCCTTTTCTCAG	chr1	MIMAT0016883	3044575	3044589	−
hsa-miR-1238	0.1	0.1	Not-Detect	Not-Detect	1	GGGGCAGACAGAC	chr19	MIMAT0005593	10662867	10662878	−
hsa-miR-517a	0.1	0.1	Not-Detect	Not-Detect	1	ACACTCTAAAGGGATGCAC	chr19	MIMAT0002852	54215579	54215596	−
hsa-miR-3163	0.1	0.1	Not-Detect	Not-Detect	1	GTCTTACTGCCCTCATT	chr11	MIMAT0015037	66701935	66701920	+
hsa-miR-622	0.1	0.1	Not-Detect	Not-Detect	1	GCTCCAACCTCAGCAGA	chr13	MIMAT0003291	90883501	90883516	−
hsa-miR-3660	0.1	0.1	Not-Detect	Not-Detect	1	TCAAAATGCTCTCCTGTC	chr5	MIMAT0018081	89312517	89312501	+
ebv-miR-BART15	0.1	0.1	Not-Detect	Not-Detect	1	TCAAGGAAACAAAACCACTGAC		MIMAT0003713			
hsv1-miR-H4	0.1	0.1	Not-Detect	Not-Detect	1	TGCTTGCCTGTCAAAC		MIMAT0008401			
hsa-miR-222*	0.1	0.1	Not-Detect	Not-Detect	1	AGGATCTACACTGGCTA	chrX	MIMAT0004569	45606472	45606457	+
hsa-miR-4283	0.1	0.1	Not-Detect	Not-Detect	1	AAACTCGCTGAGCCC	chr7	MIMAT0016914	63081480	63081494	−
hsa-miR-1264	0.1	0.1	Not-Detect	Not-Detect	1	AACAGGTGCTCAAATAAGA	chrX	MIMAT0005791	113887176	113887193	−
hsa-miR-525-5p	0.1	0.1	Not-Detect	Not-Detect	1	AGAAAGTGCATCCCTCTGG	chr19	MIMAT0002838	54200803	54200821	−
hsa-miR-3193	0.1	0.1	Not-Detect	Not-Detect	1	ACTCCTCAGATCCTACG	chr20	MIMAT0015077	30194995	30195010	−
hsa-miR-15b	9390.6537	9390.653667	Detected	Detected	1	TGTAAACCATGATGTGCTGC	chr3	MIMAT0000417	160122398	160122416	−
hsa-miR-649	0.1	0.1	Not-Detect	Not-Detect	1	GACTCTTGAACAACACAG	chr22	MIMAT0003319	21388546	21388530	+
hsa-miR-3683	0.1	0.1	Not-Detect	Not-Detect	1	TGATACTACTTCCAATGTCG	chr7	MIMAT0018111	7106630	7106612	+
ebv-miR-BHRF1-3	0.1	0.1	Not-Detect	Not-Detect	1	TGTGCTTACACACTTCCC		MIMAT0000998			
jcv-miR-J1-5p	0.1	0.1	Not-Detect	Not-Detect	1	ATGCTTTTCCCAGGTCT		MIMAT0009147			
hsa-miR-4312	0.1	0.1	Not-Detect	Not-Detect	1	TGGGGACAGGAACAA	chr15	MIMAT0016864	69094254	69094241	+
hsa-miR-1289	0.1	0.1	Not-Detect	Not-Detect	1	AAAATGCAGATTCCTGGA	chr5	MIMAT0005879	132763379	132763362	+
hsa-miR-548m	0.1	0.1	Not-Detect	Not-Detect	1	CAAAAACCACAAATACCTT	chrX	MIMAT0005917	94318174	94318157	+
hsa-miR-32*	0.1	0.1	Not-Detect	Not-Detect	1	AAATATCACACACACTAAATTG	chr9	MIMAT0004505	111808576	111808556	+
hsa-miR-18b*	0.1	0.1	Not-Detect	Not-Detect	1	GCCAGAAGGGGCAT	chrX	MIMAT0004751	133304140	133304128	+
hsa-miR-376c	0.1	0.1	Not-Detect	Not-Detect	1	ACGTGGAATTTCCTCTATG	chr14	MIMAT0000720	101506072	101506089	−
hsa-miR-455-5p	0.1	0.1	Not-Detect	Not-Detect	1	CGATGTAGTCCAAAGGCA	chr9	MIMAT0003150	116011554	116011571	−
hsa-miR-30a*	0.1	0.1	Not-Detect	Not-Detect	1	GCTGCAAACATCCGACT	chr6	MIMAT0000088	72113321	72113305	+
hsa-miR-7	25.75154	25.64133667	Detected	Detected	0.9957205	ACAACAAAATCACTAGTCTTCC	chr9	MIMAT0000252	86584708	86584687	+
hsa-miR-502-3p	54.011162	53.09176633	Detected	Detected	0.9829777	TGAATCCTTGCCCAGG	chrX	MIMAT0004775	49779264	49779278	−
hsa-miR-183	91.589933	89.8176	Detected	Detected	0.9806493	AGTGAATTCTACCAGTGCCA	chr7	MIMAT0000261	129414792	129414774	+
kshv-miR-K12-3	32.896317	32.2485	Detected	Detected	0.9803073	TCGCTGCCGTCC		MIMAT0002193			
hsa-miR-532-5p	47.706554	46.56506767	Detected	Detected	0.9760728	ACGGTCCTACACTCAAG	chrX	MIMAT0002888	49654518	49654534	−
hsa-miR-3195	5.4953567	5.314416667	Detected	Detected	0.967074	AACCCGGGCCCG	chr20	MIMAT0015079	60639874	60639884	−
hsa-miR-126	51.55732	49.235084	Detected	Detected	0.9549582	CGCATTATTACTCACGGT	chr9	MIMAT0000445	139565110	139565126	−
hsa-miR-144	23.879626	22.66426967	Detected	Detected	0.9491049	AGTACATCATCTATACTGTA	chr17	MIMAT0000436	27188621	27188603	+
hsa-miR-29a	130.72173	123.9265367	Detected	Detected	0.9480179	TAACCGATTTCAGATGGTGC	chr7	MIMAT0000086	130561568	130561550	+
hsa-miR-17*	11.22994	10.56302	Detected	Detected	0.9406123	CTACAAGTGCCTTCAC	chr13	MIMAT0000071	92002916	92002930	−
hsa-miR-500a*	48.653809	45.15188467	Detected	Detected	0.9280236	CAGAATCCTTGCCCAGGT	chrX	MIMAT0002871	49659834	49659851	−
hsa-miR-425	3293.5957	2930.148333	Detected	Detected	0.8896503	TCAACGGGAGTGATCGTG	chr3	MIMAT0003393	49032620	49032603	+
hsa-miR-22	3077.0773	2671.067667	Detected	Detected	0.8680535	ACAGTTCTTCAACTGGCAG	chr17	MIMAT0000077	1617270	1617253	+
hsa-miR-4306	4137.0643	3526.888833	Detected	Detected	0.85251	TACTGCCTTTTCTCTCCA	chr13	MIMAT0016858	100295378	100295393	−
hsa-miR-652	326.94853	278.0457067	Detected	Detected	0.8504265	CACAACCCTAGTGGC	chrX	MIMAT0003322	109298624	109298637	−
hsa-miR-210	74.164565	62.03286767	Detected	Detected	0.8364219	TCAGCCGCTGTCACAC	chr11	MIMAT0000267	568175	568161	+
hsa-miR-324-3p	987.97233	809.6543233	Detected	Detected	0.8195111	CCAGCAGCACCTGGGG	chr17	MIMAT0000762	7067411	7067396	+
hsa-miR-20b	114.22503	93.50669867	Detected	Detected	0.8186183	CTACCTGCACTATGAGCAC	chrX	MIMAT0001413	133303866	133303849	+
hsa-miR-3679-5p	7.9926	6.478473333	Detected	Detected	0.8105589	TCCCCTTCCCTGCC	chr2	MIMAT0018104	134884711	134884723	−
hsa-miR-148a	35.683917	28.813904	Detected	Detected	0.8074759	ACAAAGTTCTGTAGTGCACT	chr7	MIMAT0000243	25989603	25989585	+
hsa-miR-584	38.842927	31.20054667	Detected	Detected	0.8032491	CTCAGTCCCAGGCAAAC	chr5	MIMAT0003249	148441912	148441897	+
hsa-miR-1914*	20.82106	16.47778333	Detected	Detected	0.7913998	CCTCCCAGTGCGG	chr20	MIMAT0007890	62572888	62572877	+
hsa-miR-183*	7.7787967	5.945233333	Detected	Detected	0.764287	TTATGGCCCTTCGGT	chr7	MIMAT0004560	129414831	129414818	+
hsa-miR-1274b	165.43303	126.0169333	Detected	Detected	0.7617398	TGGCGCCCGAACA	chr19	MIMAT0005938	58024405	58024394	+
hsa-miR-181a	128.56187	97.50063367	Detected	Detected	0.7583946	ACTCACCGACAGCGT	chr1	MIMAT0000256	198828218	198828204	+
hsa-miR-17	230.65727	173.04701	Detected	Detected	0.7502344	CTACCTGCACTGTAAGC	chr13	MIMAT0000070	92002878	92002894	−
hsa-miR-423-5p	300.18206	223.2694333	Detected	Detected	0.7437801	AAAGTCTCGCTCTCTG	chr17	MIMAT0004748	28444121	28444135	−
hsa-miR-4299	223.26943	165.4330333	Detected	Detected	0.7409569	GCCTCTCATGTCACC	chr11	MIMAT0016851	11678259	11678246	+
hsa-let-7i	782.97319	579.402	Detected	Detected	0.7400024	AACAGCACAAACTACTACCTC	chr12	MIMAT0000415	62997473	62997492	−
hsa-miR-3180-3p	17.900447	12.73911333	Detected	Detected	0.7116646	GGCCTCCGGAAGC	chr16	MIMAT0015058	15005147	15005159	−
hsa-miR-103	1380.493	967.5523533	Detected	Detected	0.7008745	TCATAGCCCTGTACAATG	chr5	MIMAT0000101	167987970	167987953	+
hsa-miR-20a	185.3294	128.56187	Detected	Detected	0.6936939	CTACCTGCACTATAAGCAC	chr13	MIMAT0000075	92003331	92003348	−
hsa-miR-494	71.7979	49.60307433	Detected	Detected	0.6908708	GAGGTTTCCCGTGTA	chr14	MIMAT0002816	101496026	101496039	−
hsa-miR-107	1875.6813	1258.772	Detected	Detected	0.6711012	TGATAGCCCTGTACAATGCT	chr10	MIMAT0000104	91352575	91352557	+
hsa-miR-140-3p	3526.8888	2352.787333	Detected	Detected	0.6670999	CCGTGGTTCTACCCT	chr16	MIMAT0004597	69967052	69967065	−
hsa-miR-320d	1258.772	838.7823333	Detected	Detected	0.6663497	TCCTCTCAACCCAGC	chr13	MIMAT0006764	41302011	41301997	+
hsa-miR-3162	56.510666	36.81878	Detected	Detected	0.6515368	CTCCCCACCCTTCT	chr11	MIMAT0015036	59362581	59362569	+
hsa-miR-3665	46.832273	29.95506267	Detected	Detected	0.6396244	CGCCGCCCCG	chr13	MIMAT0018087	78272188	78272180	+
hsa-miR-320a	516.42115	326.9485333	Detected	Detected	0.6331045	TCGCCCTCTCAAC	chr8	MIMAT0000510	22102543	22102531	+
hsa-miR-93	320.75209	199.7150733	Detected	Detected	0.6226462	CTACCTGCACGAACAG	chr7	MIMAT0000093	99529359	99529344	+
hsa-miR-18b	7.2449	4.421803333	Detected	Detected	0.6103332	CTAACTGCACTAGATGCACC	chrX	MIMAT0001412	133304098	133304080	+
hsa-miR-320b	1011.2646	611.1826567	Detected	Detected	0.6043746	TTGCCCTCTCAACCC	chr1	MIMAT0005792	117214416	117214430	−
hsa-miR-106b	716.39333	420.2546667	Detected	Detected	0.5866256	ATCTGCACTGTCAGCAC	chr7	MIMAT0000680	99691647	99691632	+
hsa-miR-320c	764.37963	446.7744733	Detected	Detected	0.5844929	ACCCTCTCAACCCAG	chr18	MIMAT0005793	19263525	19263539	−
hsa-miR-638	38.047967	20.82106333	Detected	Detected	0.5472319	AGGCCGCCACCCGC	chr19	MIMAT0003308	10829107	10829119	−
hsa-miR-221	19.7279	10.37061	Detected	Detected	0.5256824	GAAACCCAGCAGACAATGT	chrX	MIMAT0000278	45605671	45605654	+
hsa-miR-29c	141.59513	74.16456533	Detected	Detected	0.5237791	TAACCGATTTCAAATGGTGCTA	chr1	MIMAT0000681	207975271	207975251	+
hsa-miR-125b	80.09745	41.72649333	Detected	Detected	0.5209466	TCACAAGTTAGGGTCTC	chr11	MIMAT0000423	121970500	121970484	+
hsa-miR-15a	628.60168	320.7520933	Detected	Detected	0.5102629	CACAAACCATTATGTGCTGCT	chr13	MIMAT0000068	50623289	50623270	+
hsa-miR-574-5p	20.643997	10.46962367	Detected	Detected	0.507151	ACACACTCACACACACAC	chr4	MIMAT0004795	38869683	38869699	−
hsa-miR-320e	1085.3267	549.8346067	Detected	Detected	0.5066075	CCTTCTCAACCCAGC	chr19	MIMAT0015072	47212601	47212588	+
hsa-miR-1915	43.390201	19.397323	Detected	Detected	0.4470439	CCCGCCGCGTC	chr10	MIMAT0007892	21785556	21785547	+
hsa-miR-96	86.276699	34.922714	Detected	Detected	0.4047757	AGCAAAAATGTGCTAGTGCCAA	chr7	MIMAT0000095	129414562	129414542	+
hsa-miR-16-2*	212.31893	81.94324	Detected	Detected	0.3859441	TAAAGCAGCACAGTAATATTGG	chr3	MIMAT0004518	160122586	160122606	−
hsa-miR-1207-5p	89.8176	32.89631667	Detected	Detected	0.3662569	CCCCTCCCAGCCT	chr8	MIMAT0005871	129061414	129061425	−
hsa-miR-2861	103.431	36.94213667	Detected	Detected	0.3571676	CCGCCCACCGC	chr9	MIMAT0013802	130548259	130548268	−
hsv2-miR-H10	134.60157	45.57075667	Detected	Detected	0.3385604	CCGCCCACCCC		MIMAT0014353			
hsa-let-7c	549.83461	185.3293967	Detected	Detected	0.3370639	AACCATACAACCTACTACC	chr21	MIMAT0000064	17912162	17912179	−
hsa-let-7b	2573.067	782.9731867	Detected	Detected	0.3042957	AACCACACAACCTACTACC	chr22	MIMAT0000063	46509575	46509592	−
hsa-miR-101	29.955063	8.814453333	Detected	Detected	0.2942559	TTCAGTTATCACAGTACTGT	chr1	MIMAT0000099	65524183	65524164	+
hsa-miR-1268	77.854233	21.45429333	Detected	Detected	0.27557	CCCCCACCACCAC	chr15	MIMAT0005922	22513250	22513239	+
hsa-miR-629	20.18733	4.93335	Detected	Detected	0.2443785	AGTTCTCCCAACGTAAAC	chr15	MIMAT0004810	70371752	70371736	+
hsa-miR-3656	173.04701	42.065686	Detected	Detected	0.2430882	CCACCCCCGCAC	chr11	MIMAT0018076	118889708	118889718	−
hsa-miR-181d	177.91727	38.04796667	Detected	Detected	0.213852	ACCCACCGACAACAATG	chr19	MIMAT0002821	13985731	13985746	−
hsa-miR-181b	199.71507	39.39461333	Detected	Detected	0.1972541	ACCCACCGACAGCA	chr1	MIMAT0000257	198828059	198828046	+
hsa-miR-595	49.235084	9.412556333	Detected	Detected	0.1911758	AGACACACCACGGCACA	chr7	MIMAT0003263	158325490	158325475	+
hsa-miR-4271	46.565068	7.113953333	Detected	Detected	0.1527745	CCCCACCTTTTCTTCC	chr3	MIMAT0016901	49311595	49311609	−
hsa-miR-939	51.069333	7.4282	Detected	Detected	0.1454532	CACCCCCAGAGCC	chr8	MIMAT0004982	145619401	145619390	+
hsa-miR-627	53.38944	6.187723333	Detected	Detected	0.1158979	TCCTCTTTTCTTAGAGACTCAC	chr15	MIMAT0003296	42491804	42491784	+
hsa-miR-30c-1*	1.5547933	0.1	Detected	Not-Detect	0.0643172	GGAGTAAACAACCCTCTCC	chr1	MIMAT0004674	41223015	41223032	−
hsa-miR-1307	110.88992	5.8974	Detected	Detected	0.0531825	CACGACCGACGCC	chr10	MIMAT0005951	105154110	105154099	+
hsa-miR-1228*	2.1246733	0.1	Detected	Not-Detect	0.0470661	CACACACCTGCC	chr12	MIMAT0005582	57588297	57588307	−
hsa-miR-337-3p	2.826	0.1	Detected	Not-Detect	0.0353857	GAAGAAAGGCATCATATAGGAG	chr14	MIMAT0000754	101340891	101340911	−
hsa-miR-129-3p	2.8731367	0.1	Detected	Not-Detect	0.0348052	ATGCTTTTTGGGGTAAGGG	chr11	MIMAT0004605	43603004	43603021	−
ebv-miR-BART13	2.91908	0.1	Detected	Not-Detect	0.0342574	TCAGCCGTCCCTGG		MIMAT0003424			
hsa-miR-196b	2.94726	0.1	Detected	Not-Detect	0.0339298	CCCAACAACAGGAAACTACC	chr7	MIMAT0001080	27209134	27209116	+
hsa-miR-670	3.1048067	0.1	Detected	Not-Detect	0.0322081	CACCACATACACTCAGG	chr11	MIMAT0010357	43581229	43581244	−

hsa-miR-424	3.1671033	0.1	Detected	Not-Detect	0.0315746	TTCAAAACATGAATTGCTGCTG	chrX	MIMAT0001341	133680675	133680655	+
hsa-miR-557	4.0198567	0.1	Detected	Not-Detect	0.0248765	AGACAAGGCCCACCCG	chr1	MIMAT0003221	168344830	168344844	−
hsa-miR-485-3p	4.0804733	0.1	Detected	Not-Detect	0.024507	AGAGAGGAGAGCCGTGT	chr14	MIMAT0002176	101521807	101521822	−
hsa-miR-520d-3p	4.22969	0.1	Detected	Not-Detect	0.0236424	ACCCACCAAAGAGAAGCAC	chr19	MIMAT0002856	54223407	54223424	−
hsa-miR-654-3p	5.3144167	0.1	Detected	Not-Detect	0.0188167	AAGGTGATGGTCAGCAGAC	chr14	MIMAT0004814	101506610	101506627	−
hsa-miR-382	5.3313133	0.1	Detected	Not-Detect	0.0187571	CGAATCCACCACGAAC	chr14	MIMAT0000737	101520660	101520674	−
hsa-miR-671-5p	5.3961333	0.1	Detected	Not-Detect	0.0185318	CTCCAGCCCCT	chr7	MIMAT0003880	150935548	150935557	−
hsa-miR-3196	5.4569	0.1	Detected	Not-Detect	0.0183254	GAGGCCCCTGCCG	chr20	MIMAT0015080	61870146	61870157	−
hsv1-miR-H6-5p	5.6521833	0.1	Detected	Not-Detect	0.0176923	TACACCCCCCTGCC		MIMAT0015281			
hsa-miR-206	5.7854833	0.1	Detected	Not-Detect	0.0172846	CCACACACTTCCTTAC	chr6	MIMAT0000462	52009206	52009220	−
hsa-miR-18a	6.33481	0.1	Detected	Not-Detect	0.0157858	CTATCTGCACTAGATGCA	chr13	MIMAT0000072	92003016	92003032	−
hsa-miR-3188	7.4282	0.1	Detected	Not-Detect	0.0134622	CCCCGTATCCGCA	chr19	MIMAT0015070	18392950	18392961	−
hsa-miR-4270	7.583083	0.1	Detected	Not-Detect	0.0131872	GCCCTCCCCTGAC	chr3	MIMAT0016900	15537775	15537764	+
hsa-miR-3652	8.6698167	0.1	Detected	Not-Detect	0.0115343	TCCTCACACCTCCAGC	chr12	MIMAT0018072	104324206	104324220	−
hsa-miR-590-5p	9.772357	0.1	Detected	Not-Detect	0.0102329	CTGCACTTTTATGAATAAGCTC	chr7	MIMAT0003258	73243479	73243500	−
hsa-miR-99b*	10.37061	0.1	Detected	Not-Detect	0.0096426	CGGACCCACAGAC	chr19	MIMAT0004678	52195919	52195930	−
hsa-miR-3917	11.31408	0.1	Detected	Not-Detect	0.0088385	CCCACCTGCTCAGT	chr1	MIMAT0018191	26232923	26232911	+
hsa-miR-409-3p	12.25099	0.1	Detected	Not-Detect	0.0081626	AGGGGTTCACCGAGCA	chr14	MIMAT0001639	100601442	100601457	−
hsa-miR-4322	13.444403	0.1	Detected	Not-Detect	0.007438	CCCCACGCGCTG	chr19	MIMAT0016873	10341141	10341151	−
ebv-miR-BART16	13.798097	0.1	Detected	Not-Detect	0.0072474	AGAGCACACACCCACT		MIMAT0003714			
ebv-miR-BART12	15.510477	0.1	Detected	Not-Detect	0.0064473	AACCACACCAAACACCACAG		MIMAT0003423			
hsa-miR-188-5p	15.89344	0.1	Detected	Not-Detect	0.0062919	CCCTCCACCATGC	chrX	MIMAT0000457	49768132	49768143	−
hsa-miR-1224-5p	23.696709	0.1	Detected	Not-Detect	0.00422	CCACCTCCCGA	chr3	MIMAT0005458	183959202	183959211	−
hsa-miR-1180	28.813904	0.1	Detected	Not-Detect	0.0034705	ACACACCCACGCG	chr17	MIMAT0005825	19247880	19247869	+
hsa-miR-3663-5p	37.52035	0.1	Detected	Not-Detect	0.0026652	CCGAGCACCACGC	chr10	MIMAT0018084	118927229	118927218	+
hsa-miR-432	50.042302	0.1	Detected	Not-Detect	0.0019983	CCACCCAATGACCTACTC	chr14	MIMAT0002814	101350839	101350855	−
hsa-miR-100	53.091766	0.1	Detected	Not-Detect	0.0018835	CACAAGTTCGGATCTACGG	chr11	MIMAT0000098	122022970	122022953	+
hsa-miR-1306	54.533826	0.1	Detected	Not-Detect	0.0018337	CACCACCAGAGCCA	chr22	MIMAT0005950	20073640	20073652	−
hsa-miR-623	72.136081	0.1	Detected	Not-Detect	0.0013863	ACCCAACAGCCCCTGC	chr13	MIMAT0003292	100008408	100008422	−
