# Supplementary material for: Ubiquitin-dependent proteolysis of CXCL7 leads to posterior longitudinal ligament ossification
Source: PLoS One. 2018 May 21;13(5):e0196204. doi: 10.1371/journal.pone.0196204 (PMC5962073; doi:10.1371/journal.pone.0196204)
Supplement: S1 Data — DOI; dx.doi.org/10.17504/protocols.io.j9gcr3w. (PDF) [file pone.0196204.s013.pdf]

Supporting Information

Ubiquitin-dependent proteolysis of CXCL7 leads to posterior longitudinal ligament ossification

Michiyo Tsuru, Atsushi Ono, Hideaki Umeyama, Masahiro Takeuchi and Kensei Nagata

Supplementary SNP data

DOI; dx.doi.org/10.17504/protocols.io.j9gcr3w

contig
Normal Exon1:
GTTTTTTTAGGCAAAGGTTTAATAGGAATTTGACCTGTCTGGTCTTTCTCTGG
GTTGGGCACAGCTTCAAATGCTTATGTGTGTATCACCACATACCCTCACTTCC
TCCTTTCCTACCTCTTCCTTCTTACTGGCTTTGAGAAAGAGCATATAAATGACA
TCTTCAGGGCATGAGAAGCCACTTATCTGCAGACTTGTAGGCAGCAACTCAC
CCTCACTCAGAGGTCTTCTGGTTCTGGAAACAACTCTAGCTCAGCCTTCTCC
ACCATGAGCCTCAGACTTGATACCACCCCTTCCTGTAACAGTGCGAGACCAC
TTCATGCCTTGCAGGTGCTGCTGCTTCTGTCATTGCTGCTGACTGCTCTGGCT
TCCTCCACCAAAGGACAAACTAAGAGAAACTTGGCGAAAGGCAAAGGTAGA
GGCCCTGCTTCTCTGCACTTGTTGCTGCTTCTGCTACACCTGTCTCTGGGGTA
AAGACTAGCTTGGTGCCTTTGGGGCTGGAGAGGGCCATTATACCAATAACTC
CAATTGGAGGAGACACACAGGGGGGTCACTTCTCACTTCTTGTGTGCTGGGC
AATCTTCTGGGCACTTTACTAAAGCGTTACAGATCAATATTCACAAACA

Normal Exon2:
TTTACTAAAAACGTTACAGATCATATTCACAATGGCTTTATGAGAGAGGTACA
ATTGCCCTCAATCTGCAAATAAGAGACCTGAGGAAAATATTCATGACCACCAA
TAGGTCACATTTTCTACCCTAGAGGAAAGTCTAGACAGTGACTTGTATGCTGA
ACTCCGCTGCATGTGTATAAAGACAACCTCTGGAATTCATCCCAAAAACATCC
AAAGTTTGGAAGTGATCGGGAAAGGAACCCATTGCAACCAAGTCGAAGTGA
TGTAAGTTGCTGTTTCTGTGCTATTGCCTTATCAGAGAAACCCTCTACCTCCAT
CCACATATGCACTCGTTTCCTCCAGTCTCATGGATTAGTTCTGATATTCAGATC
AGGACACCCACAGATAACCCTGTTCTCTTTTGCAGAGCCACACTGAAGGATG
GGAGGAAAATCTGCCTGGACCCAGATGCTCCCAGAATCAAGAAAATTGTACA
GAAAAAATTGGCAGGTGATGAATCTGCTGATTAATTTGTTCTGTTTCTGCCAA
ACTTCTTTAACTCCCA

Normal Exon3:
TTTCTGTGCTATTGCCTTATCAGAGAAACCCTCTACCTCCATCCACATATGCAC
TCGTTTCCTCCAGTCTCATGGATTAGTTCTGATATTCAGATCAGGACACCCAC
AGATAACCCTGTTCTCTTTTGCAGAGCCACACTGAAGGATGGGAGGAAAATC
TGCCTGGACCCAGATGCTCCCAGAATCAAGAAAATTGTACAGAAAAAATTGG
CAGGTGATGAATCTGCTGATTAATTTGTTCTGTTTCTGCCAAACTTCTTTAACT
CCCAGGAAGGGTAGAATTTTGAAACCTTGATTTTCTAGAGTTCTCATTTATTC
AGGATACCTATTCTTACTGTATTAAAATTTGGATATGTGTTTCATTCTGTCTCAA
AAATCACATTTTATTCTGAGAAGGTTGGTTAAAAGATGGCAGAAAGAAGATG
AAAATAAATAAGCCTGGTTTCAACCCTCTAATTCTTGCCTAAACATTGGACTG
TACTTTGCATTTTTTTCTTTAAAAATTTCTATTCTAACACAACTTGGTTGATTTT
TCCTGGTCTACTTTATGGTTATTAGACATACTCATGGGTATTATTAGATTTCATA
ATGGTCAATGTAAATAGGAATAA

OPLL Exon1:
TTTATTTAGCAAGTTAGTAGGAATTTGACCTGTCTGGTCTTTCTCTGGGTTGG
GCACAGCTTCAAATGCTTATGTGTGTATCACCACATACCCTCACTTCCTCCTTT
CCTACCTCTTCCTTCTTACTGGCTTTGAGAAAGAGCATATAAATGACATCTTC
AGGGCATGAGAAGCCACTTATCTGCAGACTTGTAGGCAGCAACTCACCCTCA

CTCAGAGGTCTTCTGGTTCTGGAAACAACTCTAGCTCAGCCTTCTCCACCAT
GAGCCTCAGACTTGATACCACCCCTTCCTGTAACAGTGCGAGACCACTTCAT
GCCTTGCAGGTGCTGCTGCTTCTGTCATTGCTGCTGACTGCTCTGGCTTCCTC
CACCAAAGGACAAACTAAGAGAAACTTGGCGAAAGGCAAAGGTAGAGGCC
CTGCTTCTCTGCACTTGTTGCTGCTTCTGCTACACCTGTCTCTGGGGTAAAGA
CTAGCTTGGTGCCTTTGGGGCTGGAGAGGGCCATTATACCAATAACTCCAATT
GGAGGAGACACACAGGGGGGTCACTTCTCACTTCTTGTGTGCTGGGCAATCT
TCTGGGCACTTTACTAAAGCGTTACAGATCATATTCACCAAAA

OPLL Exon2:
TTTACTAAAAGCGTTACAGATCATATTCACAATGGCTTTATGAGAGAGGTACA
ATTGCCCTCAATCTGCAAATAAGAGACCTGAGGAAAATATTCATGACCACCAA
TAGGTCACATTTTCTACCCTAGAGGAAAGTCTAGACAGTGACTTGTATGCTGA
ACTCCGCTGCATGTGTATAAAGACAACCTCTGGAATTCATCCCAAAAACATCC
AAAGTTTGGAAGTGATCGGGAAAGGAACCCATTGCAACCAAGTCGAAGTGA
TGTAAGTTGCTGTTTCTGTGCTATTGCCTTATCAGAGAAACCCTCTACCTCCAT
CCACATATGCACTCGTTTCCTCCAGTCTCATGGATTAGTTCTGATATTCAGATC
AGGACACCCACAGATAACCCTGTTCTCTTTTGCAGAGCCACACTGAAGGATG
GGAGGAAAATCTGCCTGGACCCAGATGCTCCCAGAATCAAGAAAATTGTACA
GAAAAAATTGGCAGGTGATGAATCTGCTGATTAATTTGTTCTGTTTCTGCCAA
ACTTCTTTAACTCCCA

OPLL Exon3:
TTTCTGTGCTATTGCCTTATCAGAGAAACCCTCTACCTCCATCCACATATGCAC
TCGTTTCCTCCAGTCTCATGGATTAGTTCTGATATTCAGATCAGGACACCCAC
AGATAACCCTGTTCTCTTTTGCAGAGCCACACTGAAGGATGGGAGGAAAATC
TGCCTGGACCCAGATGCTCCCAGAATCAAGAAAATTGTACAGAAAAAATTGG
CAGGTGATGAATCTGCTGATTAATTTGTTCTGTTTCTGCCAAACTTCTTTAACT
CCCAGGAAGGGTAGAATTTTGAAACCTTGATTTTCTAGAGTTCTCATTTATTC
AGGATACCTATTCTTACTGTATTAAAATTTGGATATGTGTTTCATTCTGTCTCAA
AAATCACATTTTATTCTGAGAAGGTTGGTTAAAAGATGGCAGAAAGAAGATG
AAAATAAATAAGCCTGGTTTCAACCCTCTAATTCTTGCCTAAACATTGGACTG
TACTTTGCATTTTTTTCTTTAAAAATTTCTATTCTAACACAACTTGGTTGATTTT
TCCTGGTCTACTTTATGGTTATTAGACATACTCATGGGTATTATTAGATTTCATA
ATGGTCAATGTAAATAGGAATAA

Exon-trimming
Normal Exon1:
AGAAGCCACTTATCTGCAGACTTGTAGGCAGCAACTCACCCTCACTCAGAGG
TCTTCTGGTTCTGGAAACAACTCTAGCTCAGCCTTCTCCACCATGAGCCTCAG
ACTTGATACCACCCCTTCCTGTAACAGTGCGAGACCACTTCATGCCTTGCAG
GTGCTGCTGCTTCTGTCATTGCTGCTGACTGCTCTGGCTTCCTCCACCAAAGG
ACAAACTAAGAGAAACTTGGCGAAAGGCAAAG

Normal Exon2:
AGGAAAGTCTAGACAGTGACTTGTATGCTGAACTCCGCTGCATGTGTATAAA
GACAACCTCTGGAATTCATCCCAAAAACATCCAAAGTTTGGAAGTGATCGGG

AAAGGAACCCATTGCAACCAAGTCGAAGTGAT

Normal Exon3:
AGCCACACTGAAGGATGGGAGGAAAATCTGCCTGGACCCAGATGCTCCCAG
AATCAAGAAAATTGTACAGAAAAAATTGGCAGGTGATGAATCTGCTGATTAA
TTTGTTCTGTTTCTGCCAAACTTCTTTAACTCCCAGGAAGGGTAGAATTTTGA
AACCTTGATTTTCTAGAGTTCTCATTTATTCAGGATACCTATTCTTACTGTATTA
AAATTTGGATATGTGTTTCATTCTGTCTCAAAAATCACATTTTATTCTGAGAAG
GTTGGTTAAAAGATGGCAGAAAGAAGATGAAAATAAATAAGCCTGGTTTCAA
CCCTCTAATTCTTGCCTAAA

OPLL Exon1:
AGAAGCCACTTATCTGCAGACTTGTAGGCAGCAACTCACCCTCACTCAGAGG
TCTTCTGGTTCTGGAAACAACTCTAGCTCAGCCTTCTCCACCATGAGCCTCAG
ACTTGATACCACCCCTTCCTGTAACAGTGCGAGACCACTTCATGCCTTGCAG
GTGCTGCTGCTTCTGTCATTGCTGCTGACTGCTCTGGCTTCCTCCACCAAAGG
ACAAACTAAGAGAAACTTGGCGAAAGGCAAAG

OPLL Exon2:
AGGAAAGTCTAGACAGTGACTTGTATGCTGAACTCCGCTGCATGTGTATAAA
GACAACCTCTGGAATTCATCCCAAAAACATCCAAAGTTTGGAAGTGATCGGG
AAAGGAACCCATTGCAACCAAGTCGAAGTGAT

OPLL Exon3:
AGCCACACTGAAGGATGGGAGGAAAATCTGCCTGGACCCAGATGCTCCCAG
AATCAAGAAAATTGTACAGAAAAAATTGGCAGGTGATGAATCTGCTGATTAA
TTTGTTCTGTTTCTGCCAAACTTCTTTAACTCCCAGGAAGGGTAGAATTTTGA
AACCTTGATTTTCTAGAGTTCTCATTTATTCAGGATACCTATTCTTACTGTATTA
AAATTTGGATATGTGTTTCATTCTGTCTCAAAAATCACATTTTATTCTGAGAAG
GTTGGTTAAAAGATGGCAGAAAGAAGATGAAAATAAATAAGCCTGGTTTCAA
CCCTCTAATTCTTGCCTAAA

Seq-data
Normal Exon1 Fw:
CGTGACAATCTCTGGGTGAGCAAGCTTCAATGCTTATGTGTGTATCACCACAT
ACCCTCACTTCCTCCTTTCCTACCTCTTCCTTCTTACTGGCTTTGAGAAAGAG
CATATAAATGACATCTTCAGGGCATGAGAAGCCACTTATCTGCAGACTTGTAG
GCAGCAACTCACCCTCACTCAGAGGTCTTCTGGTTCTGGAAACAACTCTAGC
TCAGCCTTCTCCACCATGAGCCTCAGACTTGATACCACCCCTTCCTGTAACAG
TGCGAGACCACTTCATGCCTTGCAGGTGCTGCTGCTTCTGTCATTGCTGCTGA
CTGCTCTGGCTTCCTCCACCAAAGGACAAACTAAGAGAAACTTGGCGAAAG
GCAAAGGTAGAGGCCCTGCTTCTCTGCACTTGTTGCTGCTTCTGCTACACCT
GTCTCTGGGGTAAAGACTAGCTTGGTGCCTTTGGGGCTGGAGAGGGCCATTA
TACCAATAACTCCAATTGGAGGAGACACACAGGGGGGTCACTTCTCACTTCT
TGTGTGCTGGGCAATCTTCTGGGCACTTTACTAAAGCGTTACAGATCAATATT
CACAAACA

Normal Exon1 Rv:
TGTCAGTTGCATTGCCCAGCACACAAGAAGTGAGAAGTGACCCCCCTGTGT
GTCTCCTCCAATTGGAGTTATTGGTATAATGGCCCTCTCCAGCCCCAAAGGCA
CCAAGCTAGTCTTTACCCCAGAGACAGGTGTAGCAGAAGCAGCAACAAGTG
CAGAGAAGCAGGGCCTCTACCTTTGCCTTTCGCCAAGTTTCTCTTAGTTTGTC
CTTTGGTGGAGGAAGCCAGAGCAGTCAGCAGCAATGACAGAAGCAGCAGC
ACCTGCAAGGCATGAAGTGGTCTCGCACTGTTACAGGAAGGGGTGGTATCAA
GTCTGAGGCTCATGGTGGAGAAGGCTGAGCTAGAGTTGTTTCCAGAACCAG
AAGACCTCTGAGTGAGGGTGAGTTGCTGCCTACAAGTCTGCAGATAAGTGGC
TTCTCATGCCCTGAAGATGTCATTTATATGCTCTTTCTCAAAGCCAGTAAGAA
GGAAGAGGTAGGAAAGGAGGAAGTGAGGGTATGTGGTGATACACACATAAG
CATTTGAAGCTGTGCCCAACCCAGAGAAAGACCAGACAGGTCAAATTCCTAT
TAAACCTTTGCCTAAAAAAAC

Normal Exon2 Fw:
ATTGGCTTTTGAGAGAGGTACAATTGCCCTCAATCTGCAAATAAGAGACCTG
AGGAAAATATTCATGACCACCAATAGGTCACATTTTCTACCCTAGAGGAAAGT
CTAGACAGTGACTTGTATGCTGAACTCCGCTGCATGTGTATAAAGACAACCTC
TGGAATTCATCCCAAAAACATCCAAAGTTTGGAAGTGATCGGGAAAGGAAC
CCATTGCAACCAAGTCGAAGTGATGTAAGTTGCTGTTTCTGTGCTATTGCCTT
ATCAGAGAAACCCTCTACCTCCATCCACATATGCACTCGTTTCCTCCAGTCTC
ATGGATTAGTTCTGATATTCAGATCAGGACACCCACAGATAACCCTGTTCTCT
TTTGCAGAGCCACACTGAAGGATGGGAGGAAAATCTGCCTGGACCCAGATG
CTCCCAGAATCAAGAAAATTGTACAGAAAAAATTGGCAGGTGATGAATCTGC
TGATTAATTTGTTCTGTTTCTGCCAAACTTCTTTTAACTCCCAA

Normal Exon2 Rv:
CATTCTTTAATCAGCAGATTCATCACCTGCCAATTTTTTCTGTACAATTTTCTT
GATTCTGGGAGCATCTGGGTCCAGGCAGATTTTCCTCCCATCCTTCAGTGTGG
CTCTGCAAAAGAGAACAGGGTTATCTGTGGGTGTCCTGATCTGAATATCAGA
ACTAATCCATGAGACTGGAGGAAACGAGTGCATATGTGGATGGAGGTAGAGG
GTTTCTCTGATAAGGCAATAGCACAGAAACAGCAACTTACATCACTTCGACTT
GGTTGCAATGGGTTCCTTTCCCGATCACTTCCAAACTTTGGATGTTTTTGGGA
TGAATTCCAGAGGTTGTCTTTATACACATGCAGCGGAGTTCAGCATACAAGTC
ACTGTCTAGACTTTCCTCTAGGGTAGAAAATGTGACCTATTGGTGGTCATGAA
TATTTTCCTCAGGTCTCTTATTTGCAGATTGAGGGCAATTGTACCTCTCTCATA
AAGCCATTGTGAATATGATCTGTAACGTTTTTAGTAAAA

Normal Expn3 Fw:
TAAGGCTAGCGGCTCTCATCACATATGCACTCGTTTCCTCCAGTCTCATGGATT
AGTTCTGATATTCAGATCAGGACACCCACAGATAACCCTGTTCTCTTTTGCAG
AGCCACACTGAAGGATGGGAGGAAAATCTGCCTGGACCCAGATGCTCCCAG
AATCAAGAAAATTGTACAGAAAAAATTGGCAGGTGATGAATCTGCTGATTAA
TTTGTTCTGTTTCTGCCAAACTTCTTTAACTCCCAGGAAGGGTAGAATTTTGA
AACCTTGATTTTCTAGAGTTCTCATTTATTCAGGATACCTATTCTTACTGTATTA
AAATTTGGATATGTGTTTCATTCTGTCTCAAAAATCACATTTTATTCTGAGAAG
GTTGGTTAAAAGATGGCAGAAAGAAGATGAAAATAAATAAGCCTGGTTTCAA

CCCTCTAATTCTTGCCTAAACATTGGACTGTACTTTGCATTTTTTTCTTTAAAA
ATTTCTATTCTAACACAACTTGGTTGATTTTTCCTGGTCTACTTTATGGTTATTA
GACATACTCATGGGTATTATTAGATTTCATAATGGTCAATGATAATAGGAAT

Normal Exon3 Rv:
TTTTATCCCATGAGTATGTCTAATAACCATAAAGTAGACCAGGAAAAATCAAC
CAAGTTGTGTTAGAATAGAAATTTTTAAAGAAAAAAATGCAAAGTACAGTCC
AATGTTTAGGCAAGAATTAGAGGGTTGAAACCAGGCTTATTTATTTTCATCTT
CTTTCTGCCATCTTTTAACCAACCTTCTCAGAATAAAATGTGATTTTTGAGAC
AGAATGAAACACATATCCAAATTTTAATACAGTAAGAATAGGTATCCTGAATA
AATGAGAACTCTAGAAAATCAAGGTTTCAAAATTCTACCCTTCCTGGGAGTT
AAAGAAGTTTGGCAGAAACAGAACAAATTAATCAGCAGATTCATCACCTGCC
AATTTTTTCTGTACAATTTTCTTGATTCTGGGAGCATCTGGGTCCAGGCAGATT
TTCCTCCCATCCTTCAGTGTGGCTCTGCAAAAGAGAACAGGGTTATCTGTGG
GTGTCCTGATCTGAATATCAGAACTAATCCATGAGACTGGAGGAAACGAGTG
CATATGTGGATGGAGGTAGAGGGTTTCTCTGATAAGGCAATAAGCACAGAAA

OPLL Exon1 Fw:
ACCGTTTTCTTTCCTGGATGGGCAAGCTTCAATGCTTATGTGTGTATCACCAC
ATACCCTCACTTCCTCCTTTCCTACCTCTTCCTTCTTACTGGCTTTGAGAAAGA
GCATATAAATGACATCTTCAGGGCATGAGAAGCCACTTATCTGCAGACTTGTA
GGCAGCAACTCACCCTCACTCAGAGGTCTTCTGGTTCTGGAAACAACTCTAG
CTCAGCCTTCTCCACCATGAGCCTCAGACTTGATACCACCCCTTCCTGTAACA
GTGCGAGACCACTTCATGCCTTGCAGGTGCTGCTGCTTCTGTCATTGCTGCTG
ACTGCTCTGGCTTCCTCCACCAAAGGACAAACTAAGAGAAACTTGGCGAAA
GGCAAAGGTAGAGGCCCTGCTTCTCTGCACTTGTTGCTGCTTCTGCTACACC
TGTCTCTGGGGTAAAGACTAGCTTGGTGCCTTTGGGGCTGGAGAGGGCCATT
ATACCAATAACTCCAATTGGAGGAGACACACAGGGGGGTCACTTCTCACTTC
TTGTGTGCTGGGCAATCTTCTGGGCACTTTACTAAAGCGTTACAGATCATATT
CACCAAAA

OPLL Exon1 Rv:
CTCTGTCAGTGCATGCTCCTCTCTCTAGAGTGAGAAGTGACCCCCCTGTGTGT
CTCCTCCAATTGGAGTTATTGGTATAATGGCCCTCTCCAGCCCCAAAGGCACC
AAGCTAGTCTTTACCCCAGAGACAGGTGTAGCAGAAGCAGCAACAAGTGCA
GAGAAGCAGGGCCTCTACCTTTGCCTTTCGCCAAGTTTCTCTTAGTTTGTCCT
TTGGTGGAGGAAGCCAGAGCAGTCAGCAGCAATGACAGAAGCAGCAGCAC
CTGCAAGGCATGAAGTGGTCTCGCACTGTTACAGGAAGGGGTGGTATCAAGT
CTGAGGCTCATGGTGGAGAAGGCTGAGCTAGAGTTGTTTCCAGAACCAGAA
GACCTCTGAGTGAGGGTGAGTTGCTGCCTACAAGTCTGCAGATAAGTGGCTT
CTCATGCCCTGAAGATGTCATTTATATGCTCTTTCTCAAAGCCAGTAAGAAGG
AAGAGGTAGGAAAGGAGGAAGTGAGGGTATGTGGTGATACACACATAAGCA
TTTGAAGCTGTGCCCAACCCAGAGAAAGACCAGACAGGTCAAATTCCTACTA
ACTTGCTAAATAAA

OPLL Exon2 Fw:

CATGTGCATTATGAGAGAGGTACATTGCCCTCATCTGCAAATAAGAGACCTGA
GGAAAATATTCATGACCACCAATAGGTCACATTTTCTACCCTAGAGGAAAGTC
TAGACAGTGACTTGTATGCTGAACTCCGCTGCATGTGTATAAAGACAACCTCT
GGAATTCATCCCAAAAACATCCAAAGTTTGGAAGTGATCGGGAAAGGAACC
CATTGCAACCAAGTCGAAGTGATGTAAGTTGCTGTTTCTGTGCTATTGCCTTA
TCAGAGAAACCCTCTACCTCCATCCACATATGCACTCGTTTCCTCCAGTCTCA
TGGATTAGTTCTGATATTCAGATCAGGACACCCACAGATAACCCTGTTCTCTT
TTGCAGAGCCACACTGAAGGATGGGAGGAAAATCTGCCTGGACCCAGATGC
TCCCAGAATCAAGAAAATTGTACAGAAAAAATTGGCAGGTGATGAATCTGCT
GATTAATTTGTTCTGTTTCTGCCAAACTTCTTTAACTCCCA

OPLL Exon2 Rv:
CGATACCGTTATTCAGCAGATTCATCACCTGCCAATTTTTTCTGTACAATTTTC
TTGATTCTGGGAGCATCTGGGTCCAGGCAGATTTTCCTCCCATCCTTCAGTGT
GGCTCTGCAAAAGAGAACAGGGTTATCTGTGGGTGTCCTGATCTGAATATCA
GAACTAATCCATGAGACTGGAGGAAACGAGTGCATATGTGGATGGAGGTAGA
GGGTTTCTCTGATAAGGCAATAGCACAGAAACAGCAACTTACATCACTTCGA
CTTGGTTGCAATGGGTTCCTTTCCCGATCACTTCCAAACTTTGGATGTTTTTG
GGATGAATTCCAGAGGTTGTCTTTATACACATGCAGCGGAGTTCAGCATACAA
GTCACTGTCTAGACTTTCCTCTAGGGTAGAAAATGTGACCTATTGGTGGTCAT
GAATATTTTCCTCAGGTCTCTTATTTGCAGATTGAGGGCAATTGTACCTCTCTC
ATAAAGCCATTGTGAATATGATCTGTAACGCTTTTAGTAAA

OPLL Exon3 Fw:
GCTTAGCGGTCACTCATCCACATATGCACTCGTTTCCTCCAGTCTCATGGATTA
GTTCTGATATTCAGATCAGGACACCCACAGATAACCCTGTTCTCTTTTGCAGA
GCCACACTGAAGGATGGGAGGAAAATCTGCCTGGACCCAGATGCTCCCAGA
ATCAAGAAAATTGTACAGAAAAAATTGGCAGGTGATGAATCTGCTGATTAATT
TGTTCTGTTTCTGCCAAACTTCTTTAACTCCCAGGAAGGGTAGAATTTTGAAA
CCTTGATTTTCTAGAGTTCTCATTTATTCAGGATACCTATTCTTACTGTATTAAA
ATTTGGATATGTGTTTCATTCTGTCTCAAAAATCACATTTTATTCTGAGAAGGT
TGGTTAAAAGATGGCAGAAAGAAGATGAAAATAAATAAGCCTGGTTTCAACC
CTCTAATTCTTGCCTAAACATTGGACTGTACTTTGCATTTTTTTCTTTAAAAAT
TTCTATTCTAACACAACTTGGTTGATTTTTCCTGGTCTACTTTATGGTTATTAGA
CATACTCATGGGTATTATTAGATTTCATAATGGTCAATGTAAATAGGAATAA

OPLL Exon3 Rv:
TTATTATTCCCCATTGAAGTATGTCTAATAACCATAAAGTAGACCAGGAAAAAT
CAACCAAGTTGTGTTAGAATAGAAATTTTTAAAGAAAAAAATGCAAAGTACA
GTCCAATGTTTAGGCAAGAATTAGAGGGTTGAAACCAGGCTTATTTATTTTCA
TCTTCTTTCTGCCATCTTTTAACCAACCTTCTCAGAATAAAATGTGATTTTTGA
GACAGAATGAAACACATATCCAAATTTTAATACAGTAAGAATAGGTATCCTGA
ATAAATGAGAACTCTAGAAAATCAAGGTTTCAAAATTCTACCCTTCCTGGGA
GTTAAAGAAGTTTGGCAGAAACAGAACAAATTAATCAGCAGATTCATCACCT
GCCAATTTTTTCTGTACAATTTTCTTGATTCTGGGAGCATCTGGGTCCAGGCA
GATTTTCCTCCCATCCTTCAGTGTGGCTCTGCAAAAGAGAACAGGGTTATCTG
TGGGTGTCCTGATCTGAATATCAGAACTAATCCATGAGACTGGAGGAAACGA

GTGCATATGTGGATGGAGGTAGAGGGTTTCTCTGATAAGGCAATAGCACAGA
AA

Exon 1

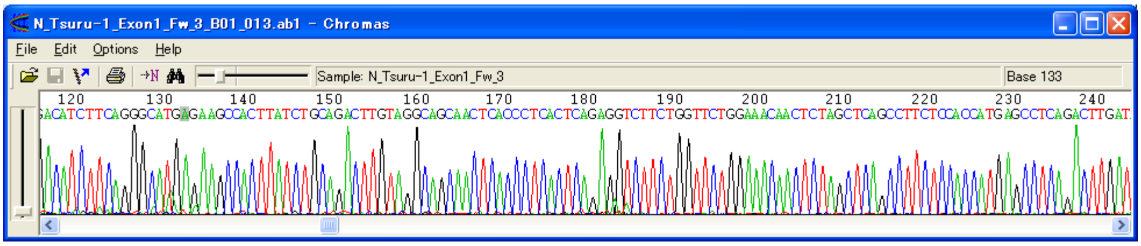

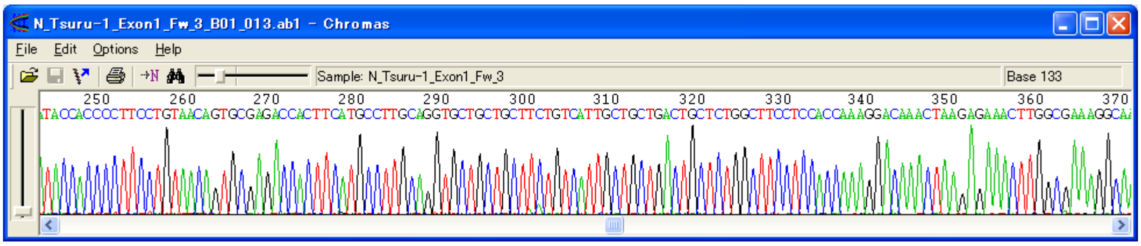

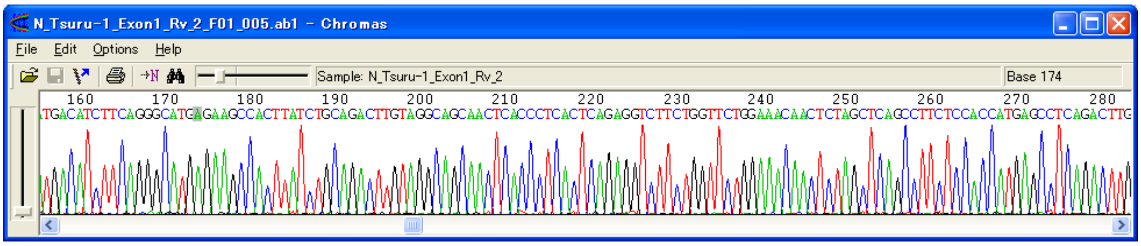

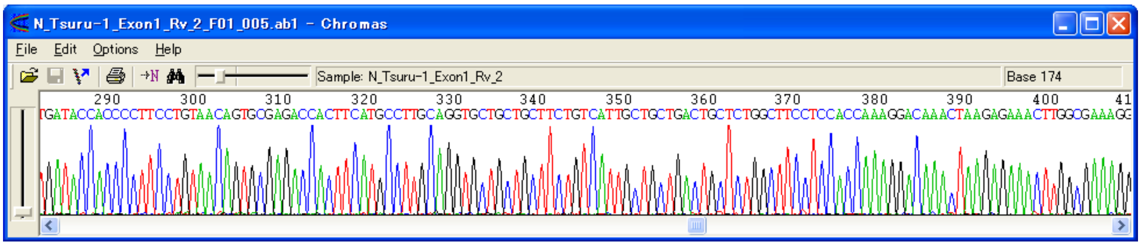

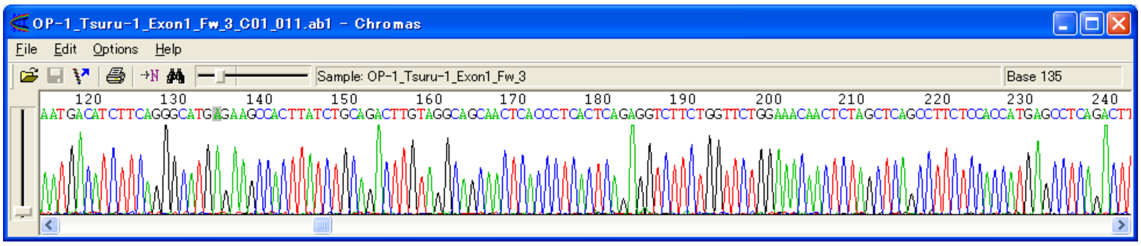

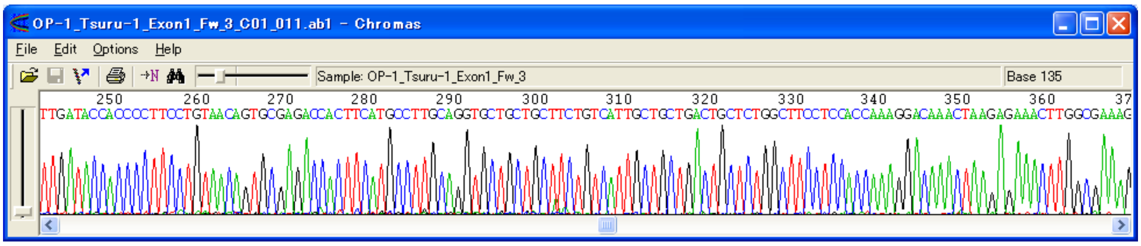
OP-1_Tsuru-1_Exon1_Fw_3_C01_011.ab1 - Chromas
File Edit Options Help
Sample: OP-1_Tsuru-1_Exon1_Fw_3
Base 135

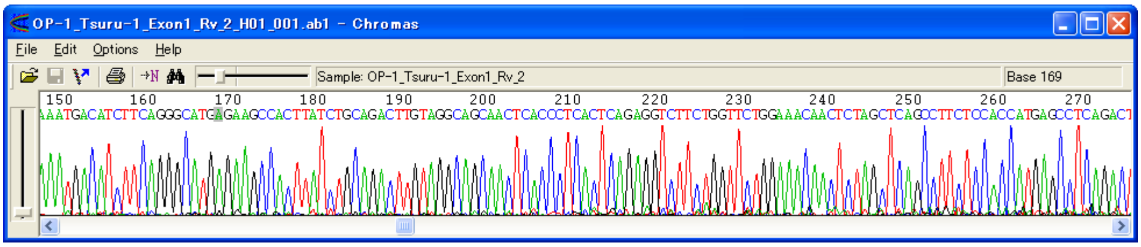
OP-1_Tsuru-1_Exon1_Rv_2_H01_001.ab1 - Chromas
File Edit Options Help
Sample: OP-1_Tsuru-1_Exon1_Rv_2
Base 169

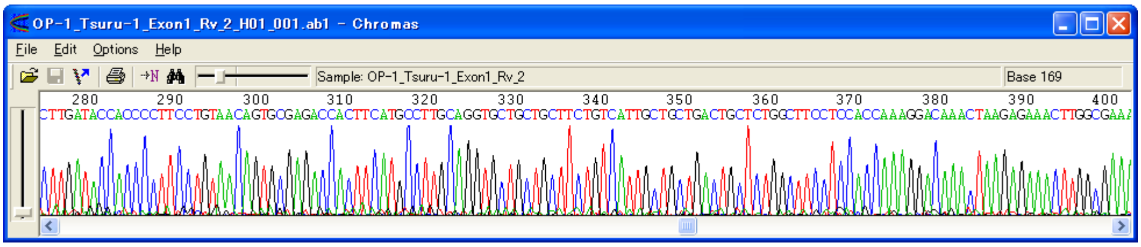
OP-1_Tsuru-1_Exon1_Rv_2_H01_001.ab1 - Chromas
File Edit Options Help
Sample: OP-1_Tsuru-1_Exon1_Rv_2
Base 169

Exon 2

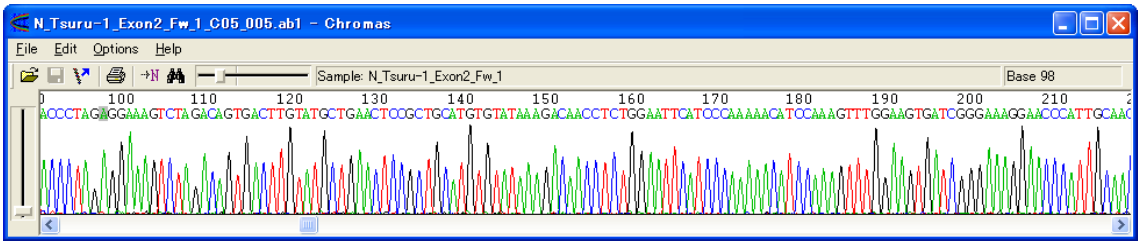
N_Tsuru-1_Exon2_Fw_1_C05_005.ab1 - Chromas
File Edit Options Help
Sample: N_Tsuru-1_Exon2_Fw_1
Base 98

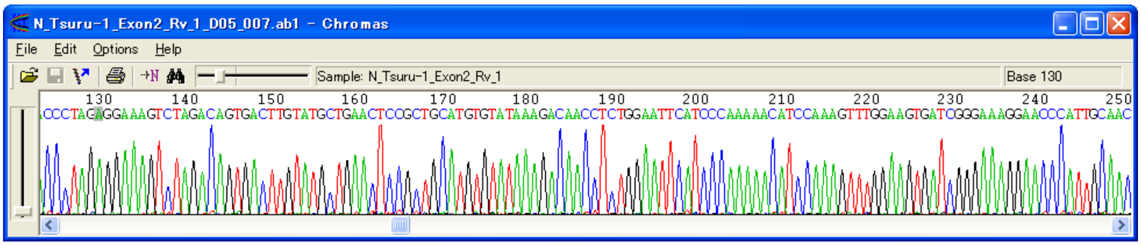
N_Tsuru-1_Exon2_Rv_1_D05_007.ab1 - Chromas
File Edit Options Help
Sample: N_Tsuru-1_Exon2_Rv_1
Base 130

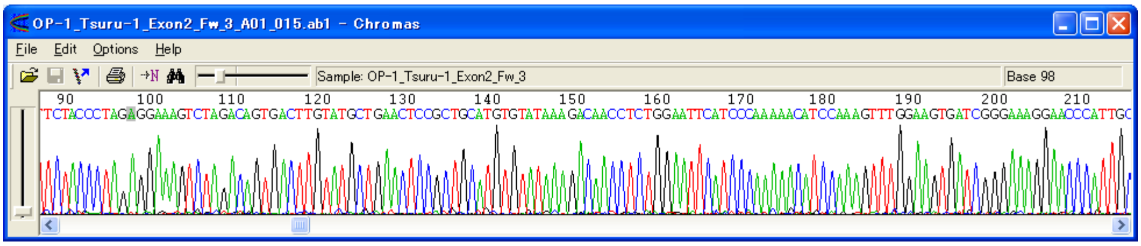
OP-1_Tsuru-1_Exon2_Fw_3_A01_015.ab1 - Chromas
File Edit Options Help
Sample: OP-1_Tsuru-1_Exon2_Fw_3
Base 98

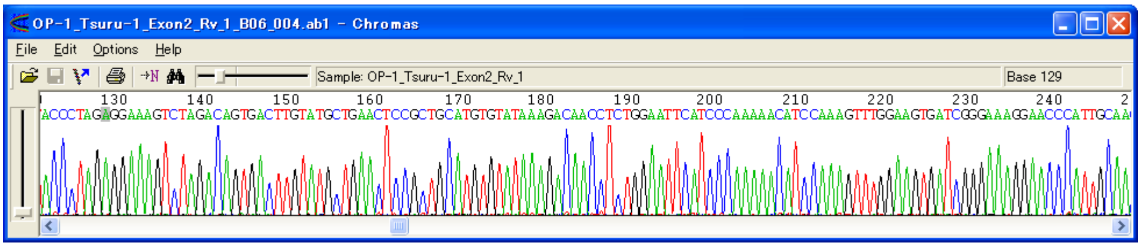
OP-1_Tsuru-1_Exon2_Rv_1_B06_004.ab1 - Chromas
File Edit Options Help
Sample: OP-1_Tsuru-1_Exon2_Rv_1
Base 129

Exon 3

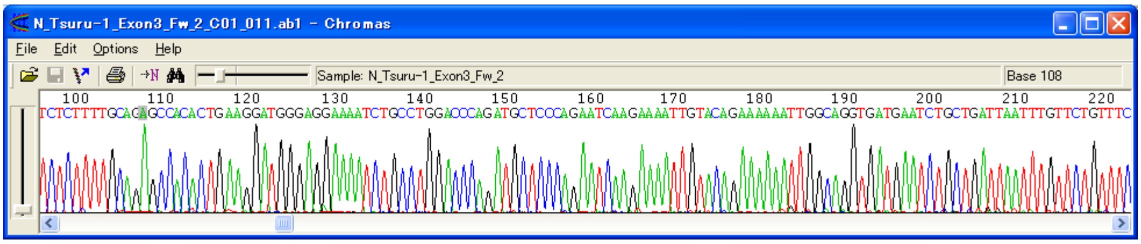
N_Tsuru-1_Exon3_Fw_2_C01_011.ab1 - Chromas
File Edit Options Help
Sample: N_Tsuru-1_Exon3_Fw_2
Base 108

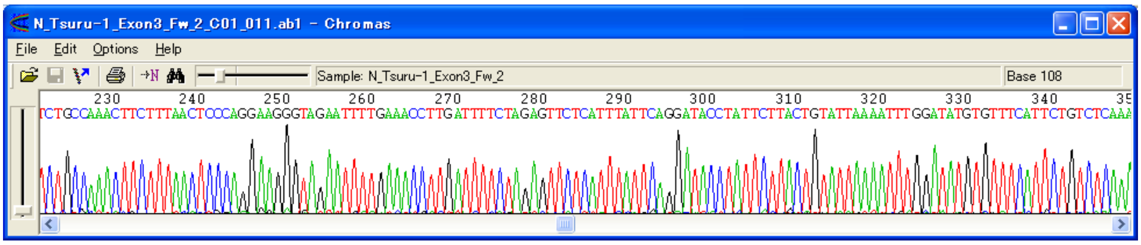
N_Tsuru-1_Exon3_Fw_2_C01_011.ab1 - Chromas
File Edit Options Help
Sample: N_Tsuru-1_Exon3_Fw_2
Base 108

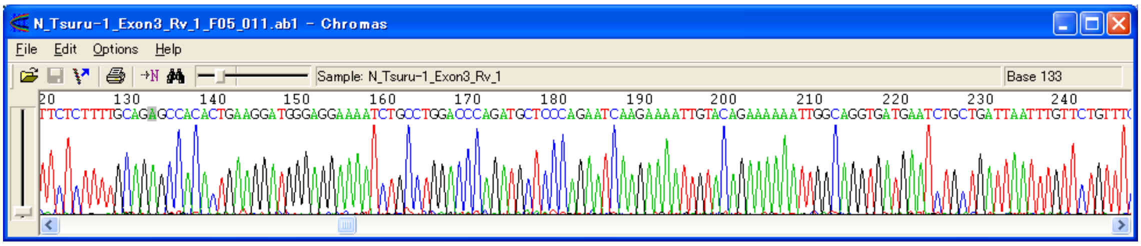
N_Tsuru-1_Exon3_Rv_1_F05_011.ab1 - Chromas
File Edit Options Help
Sample: N_Tsuru-1_Exon3_Rv_1
Base 133

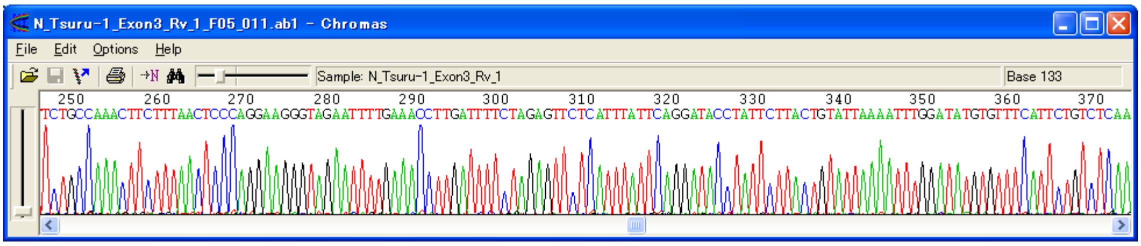
N_Tsuru-1_Exon3_Rv_1_F05_011.ab1 - Chromas
File Edit Options Help
Sample: N_Tsuru-1_Exon3_Rv_1
Base 133

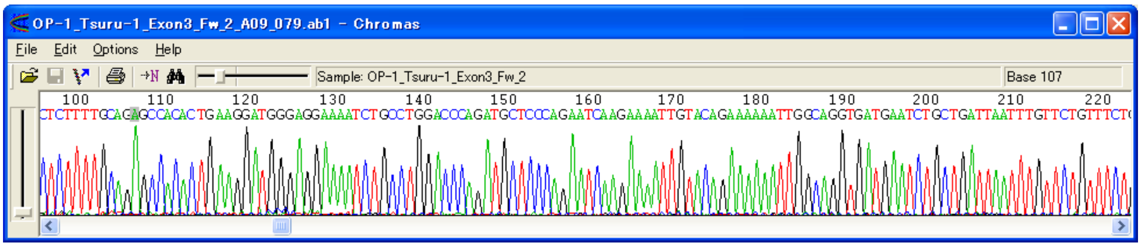
OP-1_Tsuru-1_Exon3_Fw_2_A09_079.ab1 - Chromas
File Edit Options Help
Sample: OP-1_Tsuru-1_Exon3_Fw_2
Base 107

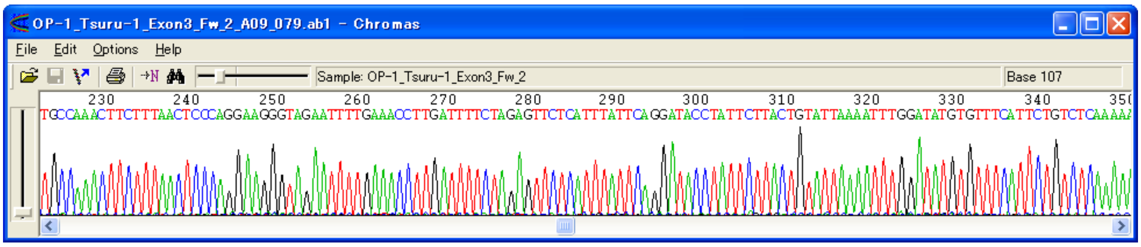
OP-1_Tsuru-1_Exon3_Fw_2_A09_079.ab1 - Chromas
File Edit Options Help
Sample: OP-1_Tsuru-1_Exon3_Fw_2
Base 107

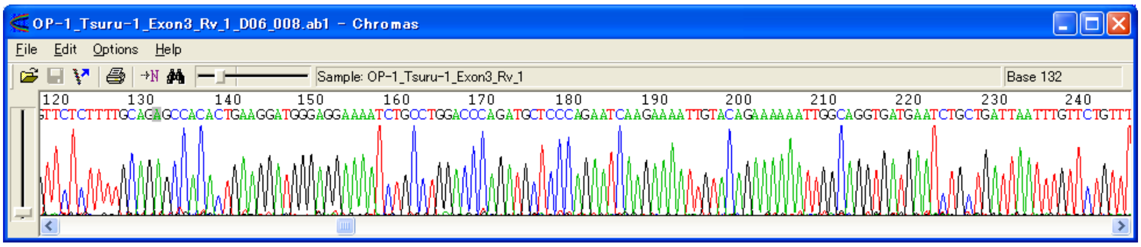
OP-1_Tsuru-1_Exon3_Rv_1_D06_008.ab1 - Chromas
File Edit Options Help
Sample: OP-1_Tsuru-1_Exon3_Rv_1
Base 132

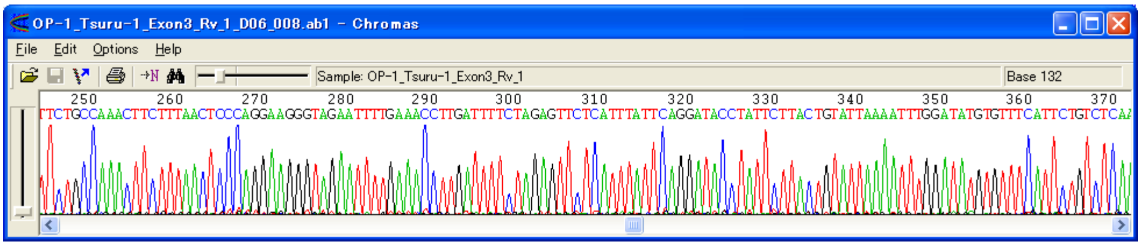
OP-1_Tsuru-1_Exon3_Rv_1_D06_008.ab1 - Chromas
File Edit Options Help
Sample: OP-1_Tsuru-1_Exon3_Rv_1
Base 132
