# Supplementary material for: Ubiquitin-dependent proteolysis of CXCL7 leads to posterior longitudinal ligament ossification
Source: PLoS One. 2018 May 21;13(5):e0196204. doi: 10.1371/journal.pone.0196204 (PMC5962073; doi:10.1371/journal.pone.0196204)

Supporting Information

Ubiquitin-dependent proteolysis of CXCL7 leads to posterior longitudinal ligament ossification

Michiyo Tsuru, Atsushi Ono, Hideaki Umeyama, Masahiro Takeuchi and Kensei Nagata

Supplementary conditional *Ppbp*$^{dE2E3/+}$ knockout mouse data.

Ppbp cKO F5, F6 Exon KO mouse PCR analysis

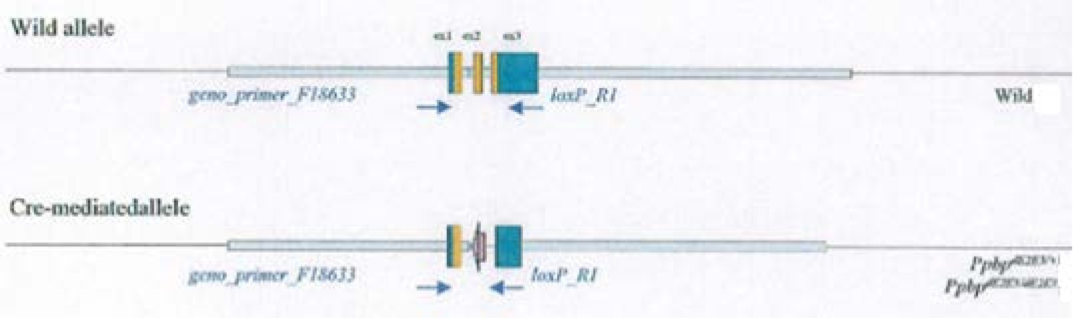

	Primer name	Primer sequence	Length
PCR primers	*geno_primer_ F18633*	GGCTTCAGACTCAGACCTACATCG	24
	LoxP_rl	AATGCAGGACATGTCAGAATGC	22

PCR conditions	PCR enzyme	KOD FX (TOYOBO, Japan)					
	Thermocycler	Gene Amp PCR System 9700 (Applied Bio Systems)					
	Component	Volume	Cycling condition 3-step				
	template	1.0 μL	Predenature	:	94.0 °C	2 mon	
	2x FX buffer	12.5 μL	Denature	:	98.0 °C	10 sec	
	2.0 mM dNTPs each	5.0 μL	Annealing	:	62.5 °C	30 sec	
	Forward primer (10 μM)	0.75 μL	Extention	:	68.0 °C	2 min	
	Reverse primer (10μM)	0.75 μL	Final extension	:	68.0°C	5 min	
	KOD FX	0.5 μL	Hold	:	4°C	∝	
	ddw	4.5 μL	Number of cycles				35 cycles
	Total	25.0 μL	Size of PCR product	: Wild			1070 bp
			Size of PCR product	: Cre-mediated			296 bp

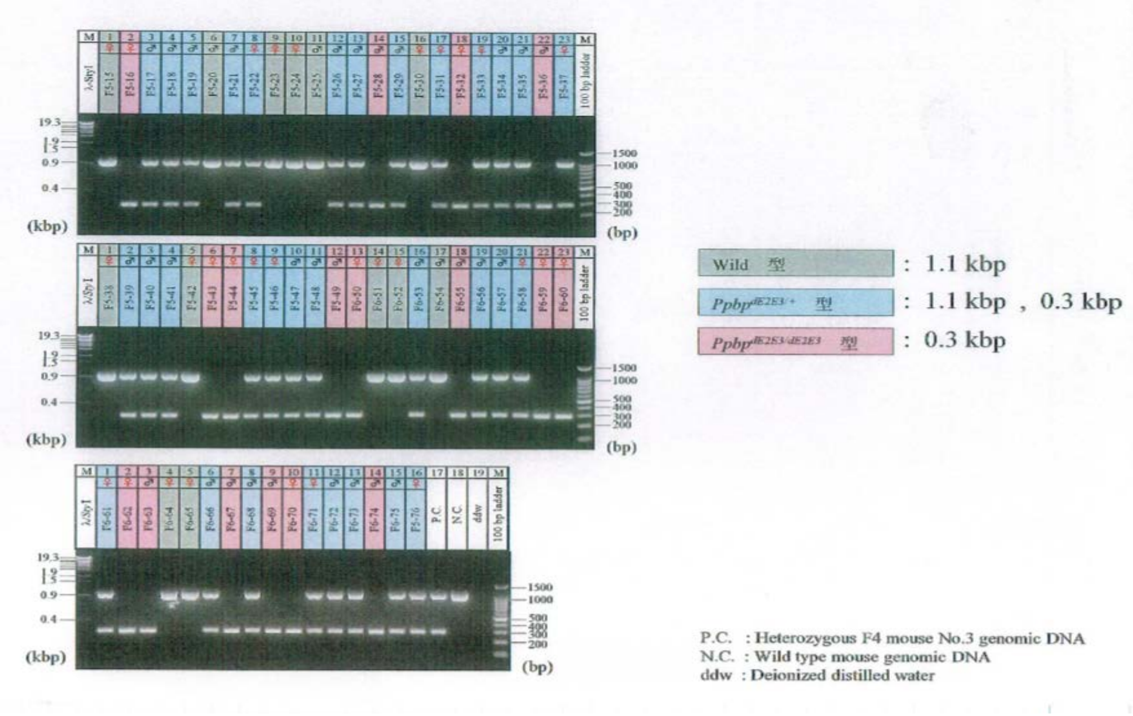

Supplement: S2 Data — (PDF) [file pone.0196204.s014.pdf]
